# Molecular Weight Control in Frontal Ring‐Opening Metathesis Polymerization

**DOI:** 10.1002/anie.202510071

**Published:** 2025-09-13

**Authors:** Kevin A. Stewart, Darya A. Ivannikava, Claire M. Massouh, Jacob J. Lessard

**Affiliations:** ^1^ Department of Chemistry University of Utah Salt Lake City UT 84112 USA

**Keywords:** Bulk, Controlled, Frontal polymerization, Reactive manufacturing, Ring opening metathesis polymerization

## Abstract

In this study, we push the limits of bulk frontal ring‐opening metathesis polymerization (FROMP) to control polymer molecular weight and dispersity without the need for solvents, deoxygenation, or post‐polymerization purification. By tuning inhibitor loadings and introducing inhibitory comonomers, we enable enhanced control over molecular weight (39–700 kg mol^−1^) and achieve dispersity as low as *Ð* = 1.07, surpassing previous limitations while preserving the thermal properties of the materials. This increased inhibition also leads to the first demonstration of closed‐mold, nonlinear propagation modes (i.e., spin modes) in FROMP of norbornene‐type monomers, where the reaction front spirals down the tube, revealing the intricate interplay between thermal transport, reaction kinetics, and gravitational effects. Furthermore, we constructed materials with gradient compositions and spatially defined variations in molecular weight, dispersity, composition, and patterning. These advances position controlled FROMP as a powerful tool for rapid, scalable, and self‐regulating material design, merging the fields of controlled polymerization and self‐propagating material manufacturing.

## Graphical abstract

Frontal ring‐opening metathesis polymerization (FROMP) of gradient thermoplastics with varying levels of control derived from both linear and nonlinear (spiral) fronts.

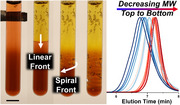

## Introduction

Controlled polymerization has advanced polymer science by enabling the meticulous manipulation of macromolecular architecture, composition, and functionality.^[^ ^1^, ^2^, ^3^, ^4^, ^5^, ^6^ ^]^ The ability to reliably target discrete chain lengths with uniform distributions has transformed polymer synthesis into a highly programmable discipline, driving innovation in material science that can meet the demands of modern technologies.^[^ ^7^, ^8^, ^9^, ^10^, ^11^ ^]^ However, despite its transformative impact, controlled polymerization techniques often suffer from long reaction times, reliance on solution‐based chemistry, and constraints imposed by heavily regulated environments, all of which slow forward progress and increase resource demands. Furthermore, extensive purification is frequently required for accurate characterization and final material manufacturing, further complicating the discovery and optimization of new polymer systems and realization of their potential applications.

Beyond these challenges, a wide range of polymer systems still adhere to a “polymerize then process” workflow, where synthesis and final material fabrication remain decoupled.^[^ ^12^, ^13^, ^14^ ^]^ This separation presents a significant hurdle, particularly within the constraints of controlled polymerizations, which have the potential to directly fabricate polymer parts with well‐defined molecular weights, low dispersity, and hierarchical morphologies.^[^ ^15^, ^16^, ^17^, ^18^ ^]^ While such precision is readily accessible in solution‐phase polymerization, integrating these advantages into direct manufacturing workflows remains an ongoing challenge in polymer science. Frontal polymerization (FP) is well poised to advance the integration of polymer synthesis and polymeric part manufacturing. By operating without solvents, requiring no external stimuli beyond a single low‐energy activation, and eliminating the need for purification, FP offers a direct route to producing functional polymer materials in their final form. This efficient and streamlined approach not only simplifies processing but also expands the possibilities for applying precision‐controlled polymerization methods to practical manufacturing.

FP is a bulk polymerization process that relies on heat generated from an exothermic polymerization to propagate an advancing reaction front for the production of polymer materials.^[^ ^19^, ^20^, ^21^, ^22^, ^23^ ^]^ This propagating front transforms neat monomer resin into a polymer material following thermochemical activation of the polymerization initiator from a local stimulus. A noteworthy FP mechanism is ring‐opening metathesis polymerization (ROMP), which relies on the opening of strained cyclic olefins to generate the requisite heat for a self‐sustaining propagation front.^[^ ^24^, ^25^, ^26^, ^27^, ^28^, ^29^, ^30^, ^31^, ^32^, ^33^, ^34^, ^35^, ^36^, ^37^, ^38^, ^39^, ^40^, ^41^ ^]^ ROMP in the context of solution‐based polymerization has been shown to enable precise control over polymer microstructure, chain length, and architecture under idealized conditions.^[^ ^42^, ^43^, ^44^, ^45^, ^46^, ^47^ ^]^ These conditions often include meticulously controlled air‐free environments, mild reaction temperatures, and (semi)dilute concentrations to regulate initiation, propagation, heat‐generation/dissipation, and reduce possible chain‐transfer events. Moving beyond these idealized conditions to bulk frontal ROMP (FROMP), we observe large temperature gradients (room temperature to 250 °C), codependent kinetic and thermal transport phenomena, and incredibly rapid reaction rates—all without requiring deoxygenation. Under these conditions, FP has only achieved an “acceptable” dispersity for controlled polymerization (*Ð* = *M̂*_weighted_/*M̂*_number _≤ 1.2) in a single specimen.^[^ ^48^ ^]^

**Figure 1:**
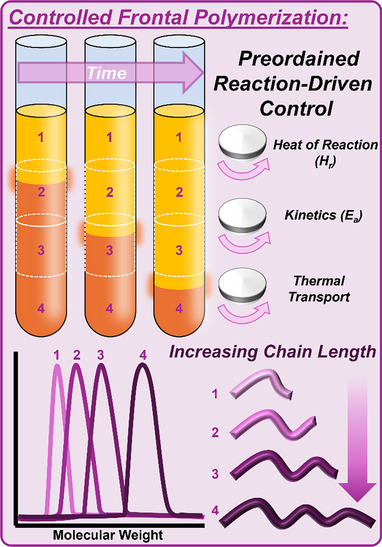
Cartoon depiction of frontal polymerization as a method for achieving preordained adaptations within polymer materials and the control parameters and size exclusion chromatography analysis depicting varying molecular weight in each region of a singular polymer part.

Our initial objectives for this work were twofold: first, to explore the limits of controlled frontal polymerization, and second, to use this controlled technique to modulate materials with preordained levels of control (Figure 1). Arising from these investigations, we discovered closed‐mold, nonlinear front propagation (i.e., spin modes) occurring both with and against the direction of gravity—a phenomenon that was previously unattainable with purely norbornene‐type monomers and has only recently been reported in FROMP resins.^[^ ^49^ ^]^ These spin modes not only pattern the material but have also been shown to augment mechanical properties of FP‐derived materials.^[^ ^49^, ^50^, ^51^, ^52^ ^]^ Finally, we utilized controlled FROMP to enable preordained adaptations within materials, providing not only global compositional control but also local variations in composition, patterning, and molecular weight distribution, paving the way for advanced and rapid material design and fabrication with precise regulation over both the macroscopic architecture and the internal composition of the constituent polymers.

## Results and Discussion

### Varying Loadings of DCPD‐H_2_

**Figure 2:**
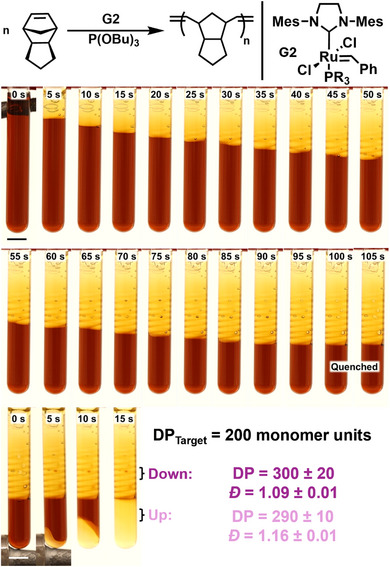
Timelapse of 200:1:10 DCPD‐H_2_:G2:TBP showing spin modes and quenching at 100 s then reinitiation from the bottom of the tube showing linear propagation (scale bars are 5 mm). Downward and upward have degree of polymerization (DP) within error yet different dispersity (*Ð*).

We began our study expanding on the previously reported controlled FROMP of dicyclopentadiene‐H_2_ (DCPD‐H_2_). DCPD‐H_2_ was synthesized in several hundred grams through hydrobromination of dicyclopentadiene to protect the norbornene olefin (90% yield), hydrogenation at ambient pressure to reduce the cyclopentene olefin (95% yield), followed by elimination to regenerate the norbornene olefin (84% yield, Figures S1–S13). FROMP resins were developed at monomer:initiator:inhibitor loadings of 4000:1:1, 2000:1:1, 1000:1:1, and 500:1:1 (Figures 2 and S14–S23). The front velocity of FROMP (*v*_f_) rose from 1.1 ± 0.2 mm s^−1^ at 4000:1:1 to 3.7 ± 0.4 mm s^−1^ at 500:1:1, attributable to elevated initiator concentration due to the bulk nature of these polymerizations. After FROMP, the resulting polymeric materials were characterized by size exclusion chromatography (SEC) showing lackluster performance in both achieving target molecular weights and maintaining low *Ð* (1.5–1.6 across all samples).

The poor control of both molecular weight and distribution is attributed to the slow initiation rate of Grubbs 2nd generation (G2) relative to propagation rate.^[^ ^53^, ^54^ ^]^ To remedy this, either a more active initiator can be used (e.g., Grubbs 3rd generation initiator) or the concentration of inhibitor can be increased to improve the initiation to propagation ratio (both rates are lowered but propagation is lowered significantly more). Under bulk conditions, a more active initiator would significantly accelerate resin activation, leading to premature gelation and spontaneous polymerization while mixing.^[^ ^55^ ^]^ Given that an increase in initiator activity is not a viable route, increasing inhibition becomes the most feasible approach. Increased inhibitor loadings should increase the control of ROMP while also aiding in longevity of the FROMP resins before activation.^[^ ^56^ ^]^

Thus, we chose to increase inhibitor loading to 10 equiv with respect to initiator for enhanced control and to identify the limits of this method. We prepared formulations with molar ratios of 4000, 2000, 1000, and 500 and—owing to increased solubility of the initiator in the resin at higher inhibitor loadings—200:1:10 monomer:initiator:inhibitor. Interestingly, with the decreased reaction kinetics leading to perturbations in thermal transport, all samples exhibited nonlinear front propagation (Figure 2), only recently shown for FROMP of cyclooctadiene and currently absent in close mold norbornene‐type monomers.^[^ ^49^ ^]^ The spinning nonlinear front propagation, also known as spin modes,^[^ ^50^ ^]^ observed was subtle for the low initiator loadings (e.g., 4000:1:10) and became increasingly more apparent at the higher loadings. The *v*_f_ increased from 0.39 ± 0.01 mm s^−1^ to 0.49 ± 0.02 mm s^−1^ for 4000:1:10 to 1000:1:10 before decreasing in rate down the tube to 0.28 ± 0.01 mm s^−1^ and 0.17 ± 0.01 mm s^−1^ for 500:1:10 and 200:1:10, respectively (Figures S24–S40 and Table S1). To explain this sudden inversion of *v*_f_ with increasing initiator concentration, we tracked the front across the tube following the spin modes and found the rates to be different with *v*_f_ of 0.35 ± 0.05, 0.6 ± 0.1, and 1.02 ± 0.09 mm s^−1^ for 1000, 500, and 200:1:10, respectively. This shows the *v*_f_ is indeed accelerating with increasing initiator loadings for the local fronts of these spin modes but is globally decreasing in the direction of gravity (Figure S41).

Interestingly, 200:1:10 quenched in the same location for all three trials and upon reinitiating from the bottom of the tube no patterns emerged when propagating against gravity. We then tested 4000, 1000, and 200:1:10 upward with *v*_f_ of 1.20 ± 0.01, 1.00 ± 0.04, and 0.97 ± 0.02 mm s^−1^, respectively. 1000:1:1 was also initiated upward to act as a control for nonlinear propagation, which eliminates spin modes as the cause for the increased upward front rate, resulting in a *v*_f_ of 3.7 ± 0.4 which was 1 mm s^−1^ faster than the downward propagation. We attribute this linear, more rapid front velocities when opposing gravity to convection—when the liquid is heated ahead of the front or during the initial activation, it becomes less dense and rises to the top causing mixing and increased initial temperature.

Cross sections of these samples were taken and then divided into three sections for analysis by SEC, NMR spectroscopy, and differential scanning calorimetry (DSC) (Figure S42). We observed a linear trend of dispersity at *x*:1:10 loadings, with *Ð* of 1.55 ± 0.01, 1.31 ± 0.01, 1.21 ± 0.02, 1.13 ± 0.01, and 1.09 ± 0.01 observed for 4000–200:1:10, respectively (Figures 3 and S43–S82; Tables S2 and S3). In hindsight, this observation is logical, as the relative concentration of inhibitor to the initiator becomes increasingly diluted at lower initiator loadings, despite maintaining the same molar equivalence. Consequently, the significance of the 10‐fold equivalence on control diminishes under conditions where the initiator is present in low concentrations (e.g., 4000:1:10). This trend was further reflected in *Ð* for the upward 10‐equiv inhibitor controls, eliminating the spin modes as the cause for this molecular weight distribution dependence.

**Figure 3:**
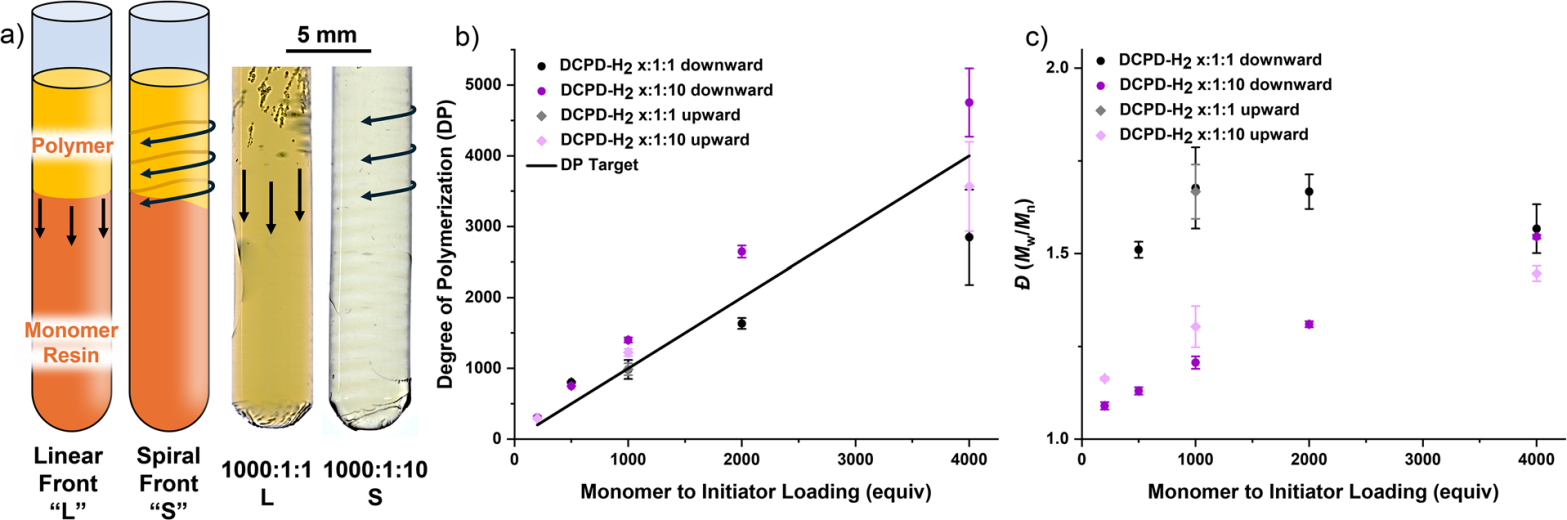
a) Cartoon depiction of front propagation for linear and spiral modes with 1000:1:1 and 1000:1:10 DCPD:G2:P(OBu)_3_ (monomer:initiator:inhibitor). b) Degree of polymerization (DP) and c) dispersity (*Ð* = *M̂*_weighted_/*M̂*_number_) for pDCPD‐H_2_ samples both down (with gravity) and upward (against gravity).

NMR spectroscopy of the resulting polymeric materials revealed near‐quantitative conversion and consistent *E*/*Z* configuration ratios (Figures S83–S97 and Table S4). The *E* configuration percentages for polymers were 63.2 ± 0.9% and 59.3 ± 0.5% across all loadings at 1 and 10 equiv inhibitor, respectively. These values are slightly higher than the *E* configurational percentages observed in solution‐state ROMP, where the *E* configuration percentage is approximately 50%, likely owing to the elevated temperatures promoting isomerization.^[^ ^48^ ^]^

DSC analysis was performed over two heating cycles to observe glass transition temperatures (*T*_g_, polymer glass to rubbery transition), exotherms from residual monomer consumption, or endotherms from small molecule boiling (Figures S98–S111; Tables S5, S6). The materials prepared using 1 equiv inhibitor loading displayed *T*_g_ values for the first heating cycle of 113 ± 7 °C across all loadings, consistent with FROMP‐based thermosets synthesized from *exo*‐DCPD.^[^ ^24^ ^]^ The exotherms from residual monomer conversion were minimal, with calculated conversions of 99 ± 1% for all loadings, agreeing with NMR spectroscopy. For the materials prepared using 10 equiv inhibitor loading, residual exotherms remained relatively low, with calculated conversions of 98.4 ± 0.3 to 94.7 ± 0.2% for 4000:1:10 and 200:1:10, respectively. However, the *T*_g_ values for the first heating cycle were notably lower, with values of 104 ± 5, 99 ± 2, 101 ± 1, 44 ± 1, and 41 ± 1 °C for 4000–200:1 monomer‐to‐initiator ratios. This dataset shows that high inhibitor loadings are detrimental to the thermal properties of the material, particularly in the lower DP samples demonstrating the highest level of control. This significant plasticization remains consistent in upward, unpatterned controls, with *T*_g_ values of 106 ± 3, 76 ± 4, and 37 ± 1 °C for 4000, 1000, and 200:1:10. This large decrease in *T*_g_ can be attributed to a combination of unconverted monomer and increased concentration of inhibitor plasticizing the material—small molecules increasing free volume between polymer chains, making long‐range polymer motion easier resulting in a reduction of *T*_g_.

### Anchimeric Monomer Inhibition via Copolymerization

**Figure 4:**
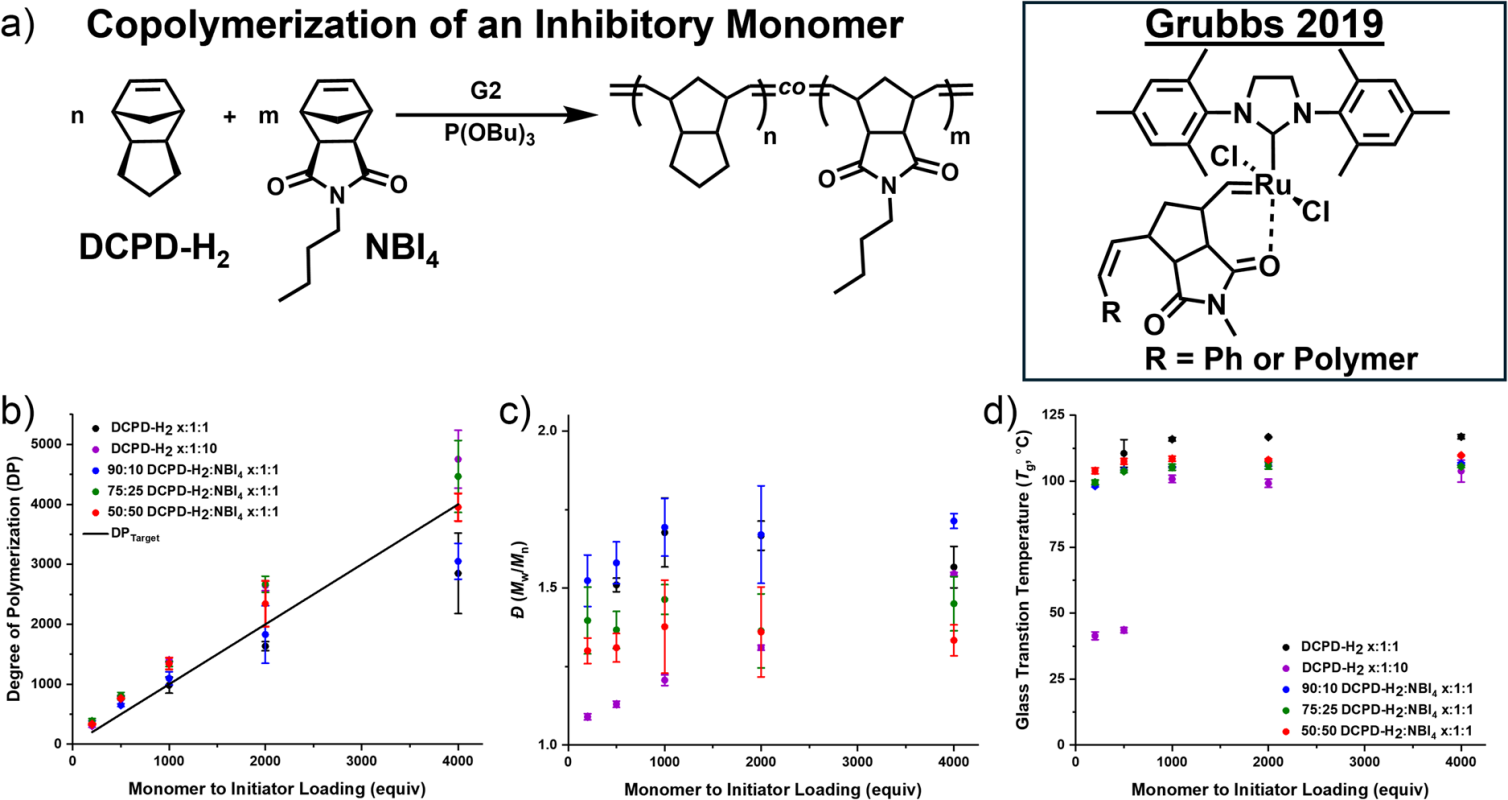
a) Reaction scheme for copolymerization of dicyclopentadiene‐H_2_ (DCPD‐H_2_) and exo‐butyl norbornene imide (NBI_4_) mixtures with Grubbs 2nd generation initiator (G2) and exogenous tributyl phosphite inhibitor (P(OBu)_3_) with inset showing previous work on anchimeric monomer inhibition of norbornene imides. b) Degree of polymerization (DP) and c) dispersity (*Ð* = *M̂*_weighted_/*M̂*_number_) for varying loadings of NBI_4_ downward. d) Glass transition temperature (*T*_g_) from DSC at varied loadings of monomer‐to‐initiator and loadings of NBI_4_.

With the excess phosphite inhibitor leading to plasticization of the materials, we sought a method to achieve inhibition and control independently within FROMP, thereby eliminating the need to compromise desirable material properties for polymerization control. Inspired by Grubbs’ investigations on terminal monomer chelation of the active Ru‐chain end,^[^ ^57^ ^]^ we hypothesized that incorporating a chelating monomer into the polymer could provide anchimeric (Greek “anchi” and “meros” referring to near/neighboring and part) inhibition,^[^ ^58^, ^59^, ^60^, ^61^ ^]^ reducing the reliance on small molecule plasticizers while maintaining similar levels of control. We synthesized *n*‐butyl‐*exo*‐norborneneimide (NBI_4_) in a single step (Figures S112–S115) with a yield of 96.8%. Moreover, NBI_4_ is a liquid, which facilitates straightforward mixing with DCPD‐H_2_, and has been shown to inhibit Grubbs‐type initiators within the context of solution‐based ROMP.^[^ ^57^, ^62^, ^63^, ^64^ ^]^ We targeted 10, 25, and 50 mol% NBI_4_ at 4000–200:1:1 monomer:initiator:exogenous inhibitor (P(OBu)_3_) (Figures 4a and S116–S163; Table S7), as loadings of 75 mol% NBI_4_ or greater did not yield a sustained reaction front.

Cure kinetics from DSC displayed a decrease in the heat of reaction (*H*_r_) from 372 ± 9 J g^−1^ to 273 ± 6 J g^−1^ for 1000:1:1 DCPD‐H_2_ and 50 mol% NBI_4_, respectively, with increasing loading of NBI_4_ due to the additional mass of the monomer (Figures S164–S186 and Table S8). Interestingly, no peak temperature change for the cure kinetics was observed from DCPD‐H_2_ at 1 equiv of P(OBu)_3_ (*T*_peak, 1000:1:1_ = 73 ± 1 °C) to 50 mol% NBI_4_ (*T*_peak, 1000:1:1_ = 72 ± 2 °C), whereas the 10 equiv P(OBu)_3_ loading in DCPD‐H_2_ showed a significant shift to higher temperature (*T*_peak, 1000:1:10_ = 86 ± 1 °C), which can typically be related to the level of inhibition of the FROMP resin. Nevertheless, we observed decreased *v*_f_ with increased loading of NBI_4_, from a *v*_f_ of 3.7 ± 0.4 mm s^−1^ for pure DCPD‐H_2_ at 500:1:1 to 0.7 ± 0.1 mm s^−1^ for 500:1:1 for 50 mol% NBI_4_, suggesting this monomer was indeed acting as a source of inhibition during the polymerization.

Excitingly, both the 25 and 50 mol% NBI_4_ displayed nonlinear front propagation, though quite subtle compared to the *x*:1:10 DCPD‐H_2_ examples (Video S1: 10, 25, and 50 mol% NBI_4_ at 500:1:1 monomer:initiator:inhibitor). The resulting polymers from 10, 25, and 50 mol% were characterized by SEC, NMR spectroscopy, and DSC (Figures S187–S284 and Tables S9–16). Molecular weight and dispersity control from SEC improved with loading of NBI_4_ across all monomer‐to‐initiator loadings (Figure 4b,c). Rather than changing in dispersity across monomer loadings observed in the DCPD 10 equiv samples, the *Ð* remained constant at each initiator loading due to the constant NBI_4_ concentration, which we attribute to the increase in inhibitory monomer content. The *Ð* for 10, 25, and 50 mol% NBI_4_ were 1.6 ± 0.1, 1.4 ± 0.1, and 1.3 ± 0.1 across all monomer‐to‐initiator loadings, improving with increasing NBI_4_ loading compared to the pure DCPD‐H_2_ at the same exogenous inhibitor loading (*Ð* = 1.6 ± 0.1). Gratifyingly, the consistent dispersity across loadings is another indication that this anchimeric inhibition is aiding in the control of FROMP without necessitating higher loadings of exogenous inhibitor, which in the 10 equiv example led to plasticization of the materials.

NMR spectroscopy of the resulting materials displayed reveals statistical incorporation of both monomers and considerable broadening of the olefin peaks, strongly indicating random distribution of the two monomers rather than compositional drift. DSC of these materials also displayed a single *T*_g_ for each sample of 115 ± 4, 104 ± 3, 104 ± 3, and 108 ± 2 °C for 0, 10, 25, and 50 mol% NBI_4_ across all monomer‐to‐initiator loadings for the first heating cycle (Figure 4d). Moreover, residual exotherms of monomer in the materials were conversions of 99 ± 1%, 98 ± 1%, and 98 ± 1% for 10, 25, and 50 mol% NBI_4_ across all monomer‐to‐initiator loadings, agreeing with the lack of residual monomer olefin peaks observed by NMR spectroscopy.

**Figure 5:**
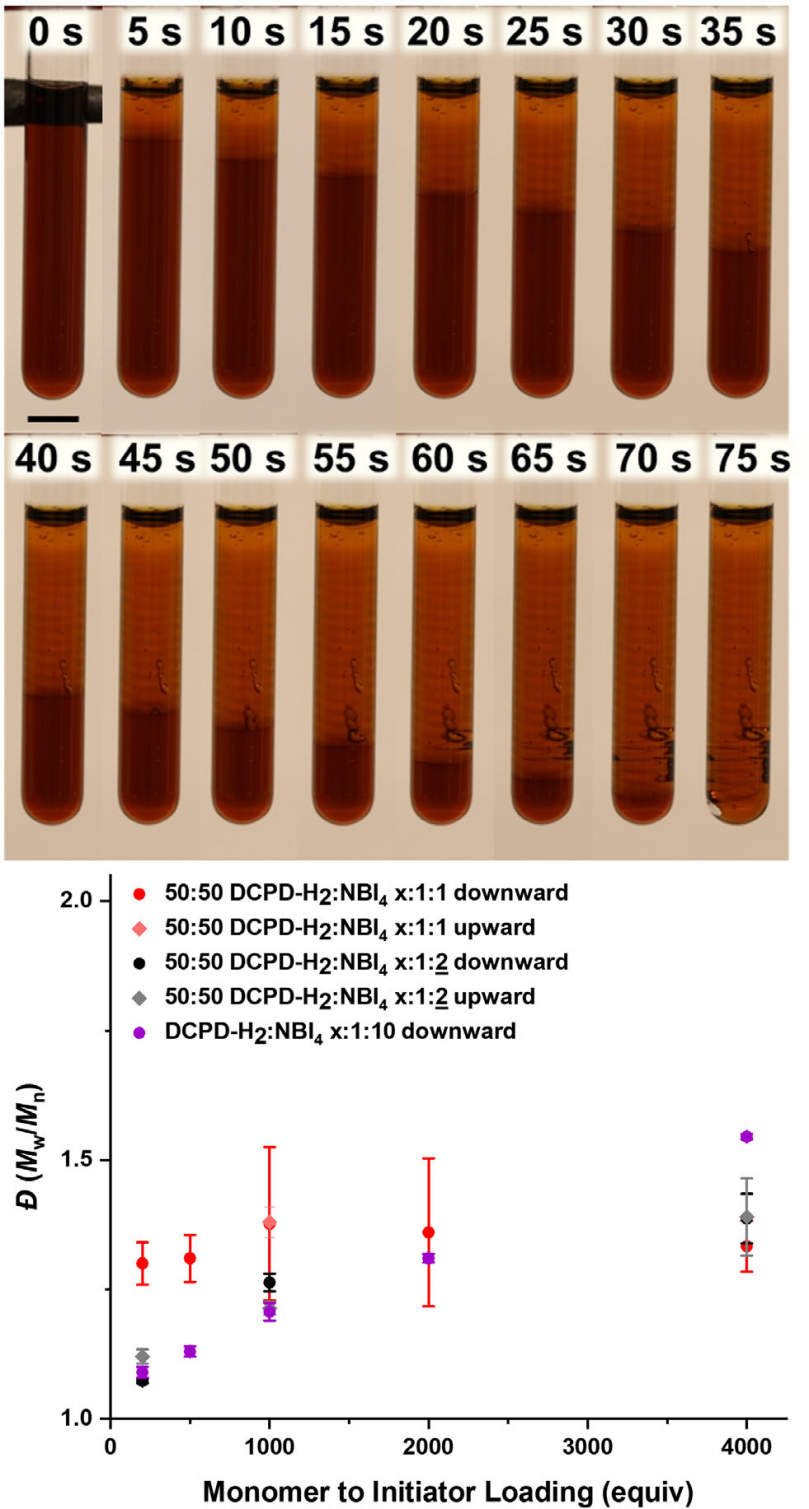
Timelapse of 50:50 200–1–2 showing spin modes (scale bar is 5 mm) and resulting dispersity (*Ð* = *M̂*_weighted_/*M̂*_number_) for varying loadings of NBI_4_ downward.

Unsatisfied with the lowest *Ð* achieved for our anchimeric inhibition sample set (*Ð =* 1.3 ± 0.1), we increased the loading of exogenous inhibitor to 2 equiv for 50 mol% NBI_4_ at 200:1, 1000:1, and 4000:1 monomer‐to‐initiator loadings. DSC of the monomer resin displayed a similar trend of *H*_r_ compared to the 1 equiv inhibitor loadings. However, similar to the 0% NBI_4_ at 10 equiv inhibitor, the peak temperature of the DSC exotherms increased from 73 ± 2 to 76 ± 1 °C for 1 and 2 equiv exogenous inhibitor, respectively, across all three loadings. Upon initiating the fronts of these resins, a significant increase in the breadth and prominence of the spin modes was observed (Figure 5) with *v*_f_ down the tube of 0.34 ± 0.09, 0.34 ± 0.05, 0.30 ± 0.02 mm s^−1^. This interesting constant front rate across monomer‐to‐initiator loadings is better understood when the rate of the front across the tube is considered with values of 0.50 ± 0.09 and 0.66 ± 0.06 mm s^−1^ for 1000:1:2 and 200:1:2, respectively. Like the DCPD‐H_2_ at 10 equiv exogenous inhibitor, the *v*_f_ is indeed increasing with the increasing loadings of initiator for the local fronts of these spin modes but in this case remaining the same in the direction of gravity. Cross sections of the 50 mol% NBI_4_ at 2 equiv exogenous inhibitor were taken and similarly analyzed by SEC, DSC, and NMR spectroscopy. Degree of polymerization (DP) from SEC of 4000, 1000, and 200:1:2 was 2900 ± 300, 1000 ± 100, and 305 ± 5, respectively. The *Ð* for the samples were 1.39 ± 0.05, 1.26 ± 0.01, and 1.07 ± 0.01 for 4000, 1000, and 200, showing again an increase in the dispersity of the samples with decreased initiator loading, but now the maximum dispersity is dictated by the anchimeric monomer inhibitor concentration. NMR spectroscopy of the samples shows a clear incorporation of each monomer into the polymer with little to no residual monomer, which is further confirmed through DSC.

As a control, we also ran 50 mol% NBI_4_ at 1000:1:1, 200:1:2, 1000:1:2, and 4000:1:2 in the upward direction. Excitingly, in all three trials, the 50 mol% at 1000:1:1 (Video S2: upward and downward spin modes of 50 mol% NBI_4_ at 1000:1:1 monomer:initiator:inhibitor) featured subtle spin modes upwards, which to the best of our knowledge has not been observed for any FP examples. We attribute this to the rapid activation time to induce a front with 1 equiv of inhibitor at 50 mol% NBI_4_ compared to the sluggish activation times of the 2 equiv inhibitor examples (6 s for initial polymer formation and ∼20 s until an appreciable front began, Video S3: upward and downward propagation of 50 mol% NBI_4_ at 1000:1:2 monomer:initiator:inhibitor). We believe this increased activation rate limits significant convective mixing and thus does not increase the initial temperature. Additionally, the rate at which the 1000:1:1 at 50 mol% NBI_4_ propagates upward with a *v*_f_ of 0.96 ± 0.07 mm s^−1^ dispels our preconceived notion that these spin modes must be in the direction of gravity and sluggish. Nevertheless, the resulting materials maintained similar thermal properties and control compared to their downward, patterned counterparts.

### Global Gradients

**Figure 6:**
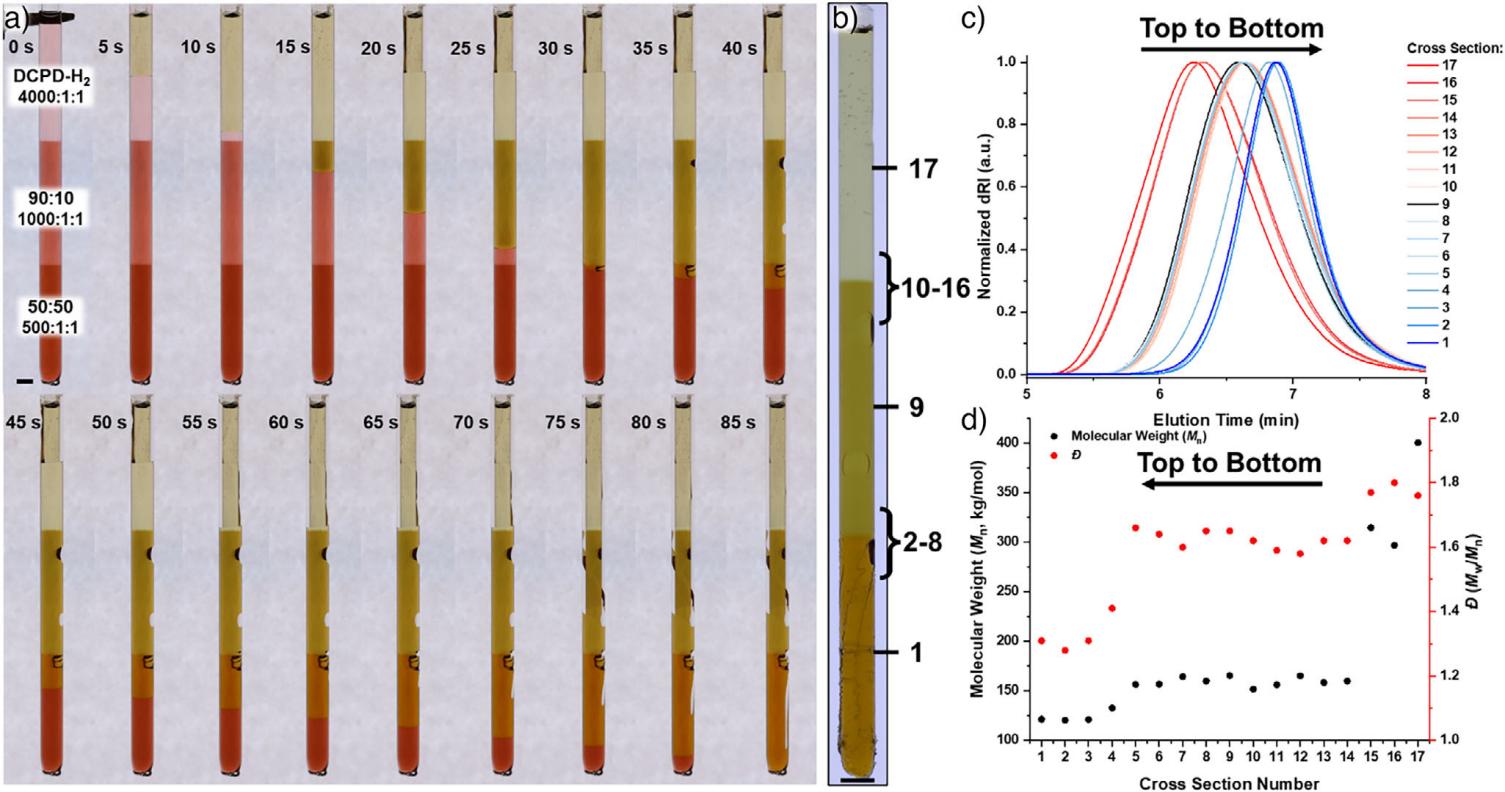
a) Timelapse of stacked DCPD‐H_2_ at 4000:1:1 monomer:initiator:inhibitor equivalent ratio (top), 10 mol% NBI_4_ resin at 1000:1:1 monomer:initiator:inhibitor equivalent ratio (middle), and 50 mol% NBI_4_ resin at 500:1:1 monomer:initiator:inhibitor equivalent ratio (bottom) with a sharp interface between each resin. b) Image of gradient material with approximate cross‐section location. c) Size‐exclusion chromatography of each cross section with d) sharp transitions of molecular weight and dispersity from the top of the tube to the bottom.

Building on the global optimization of molecular weight and *Ð* control in FROMP, we applied this knowledge to layered systems of FP to demonstrate its ability to adapt reactions and control polymerization within distinct components. By varying the polymerization within a single polymer monolith, we can achieve a wide array of pre‐ordained reaction adaptations. Specifically, we sought to control not only global compositions but also local variations in composition, patterning, *Ð*, and molecular weight. We began by layering different resin (co)monomer compositions and initiator ratios: 4000:1:1 at 0 mol% NBI_4_, 1000:1:1 at 10 mol% NBI_4_, and 500:1:1 at 50 mol% NBI_4_, arranged at the top, middle, and bottom, respectively. Importantly, the layers exhibited sharp interfaces, owing to the distinct resin densities between them. We activated the frontal polymerization from the top of the tube, transitioning from linear front propagation for 0 and 10 mol% NBI_4_ to nonlinear front propagation for the 50 mol% NBI_4_ (Figures 6a and S285; Video S4: stacked monomer resins with a sharp interface). After removing the material from the tube and preparing cross‐sections of the monolith for SEC, we observed sharp transitions of molecular weight and dispersity down the tube, with minimal intermediate compositions at the interfaces (Figures 6b–d and S286; Table S17).

**Figure 7:**
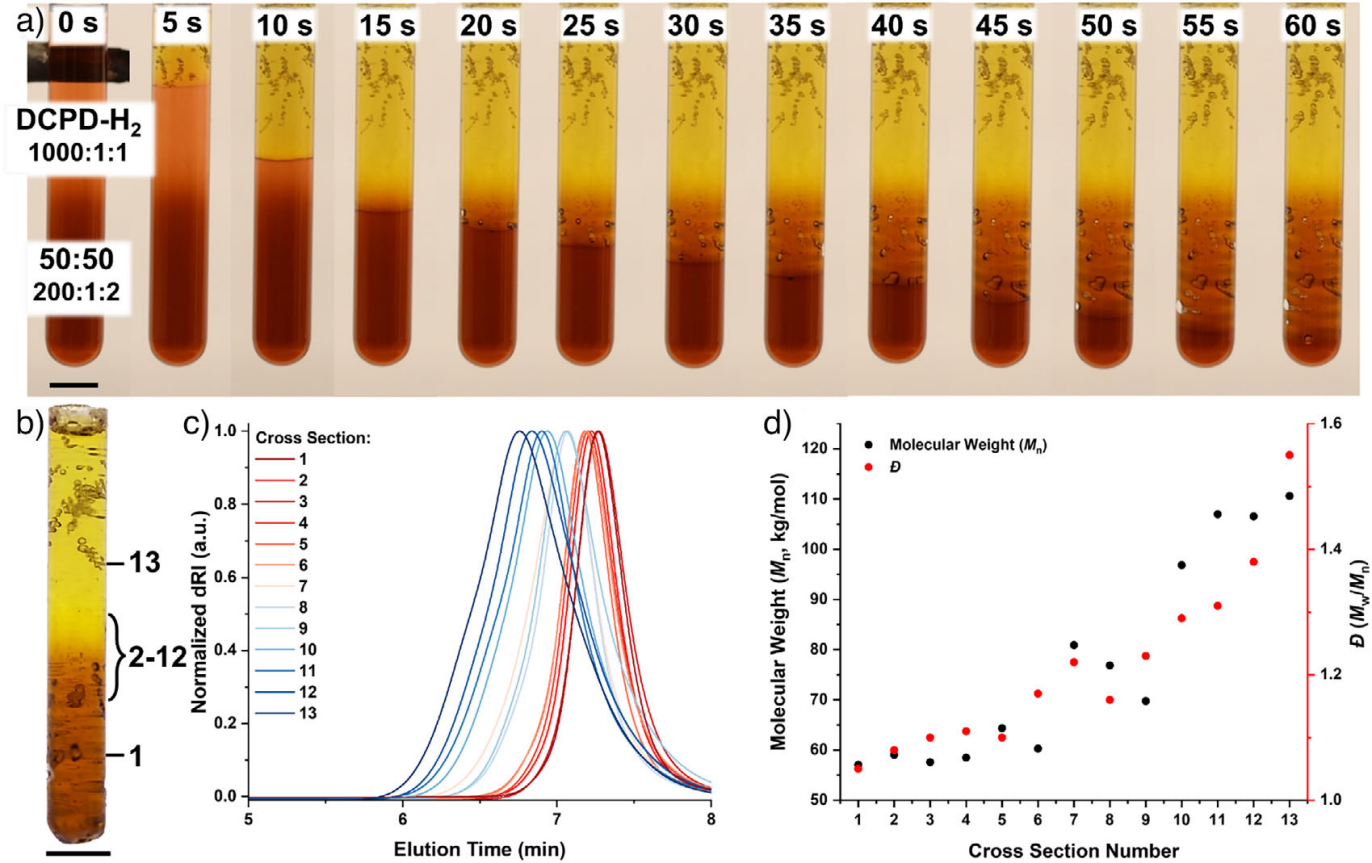
a) Timelapse of stacked DCPD‐H_2_ at 1000:1:1 monomer:initiator:inhibitor equivalent ratio (top) and 50 mol% NBI_4_ resin at 200:1:2 monomer:initiator:inhibitor equivalent ratio (bottom) with a gradient interface, showing transition from linear to nonlinear fronts within the same specimen. b) Image of gradient material with approximate cross‐section location. c) Size‐exclusion chromatography of each cross section with d) decreasing molecular weight and dispersity from the top of the tube to the bottom.

To explore more gradual transitions of molecular weight and *Ð*, we created a gradient interface by layering 1000:1:1 at 0 mol% NBI_4_ and 200:1:2 at 50 mol% NBI_4_, adding the less dense resin (0 mol% NBI_4_) first to promote mixing as the layers inverted (Figure S287). We activated a linear front for the 0 mol% NBI_4_ resin with a *v*_f_ of 1.7 mm s^−1^ before rapidly transitioning to a spin mode at the gradient interface, where *v*_f_ shifted to 0.3 and 1.8 mm s^−1^ downward and across the tube, respectively (Figures 7a and S288; Video S5: stacked monomer resins with a gradient interface). The resulting polymeric material was removed from the tube, with cross‐sections cut from the middle of each layer, while the interface was cut into 10 sections (∼0.5–1 mm thickness) to capture the gradient composition. Excitingly, we observed distinct molecular weights and *Ð* values across the interface, ranging from 111 kg mol^−1^ at a *Ð* of 1.55 at the top of the tube to 57 kg mol^−1^ at a *Ð* of 1.05 at the bottom (Figures 7b–d and S289; Table S18). This highlights how chain length control in FROMP enables the use of preordained mixed systems, where each slice of the resulting material is a unique reaction. Recreating such a material through the individual fabrication of each layer would be extremely laborious and impractical. While in this example we leveraged diffusion mixing to control compositional alteration, coupling controlled FP with technology‐guided mixing, systematic control over the material composition could be regulated more precisely, such as modulating monomer feed ratios for direct ink writing of FROMP materials.^[^ ^29^, ^33^ ^]^ Collectively, this strategy introduces a method for straightforward control over molecular weight, composition, and dispersity gradients within a single polymer material. By enabling such precision and adaptability, our approach opens new possibilities for rapidly designing materials with tailored mechanical and functional properties. For example, the stacking technique also allows effective material welding, with mechanical failure occurring within bulk domains rather than at the interface of the resins (Figures S290–S309; Tables S19–S21). This approach has potential for advancing the study of how these parameters affect the material properties of both thermosets and thermoplastics across various applications, as recently demonstrated by Anastasaki, Konkolewicz, and colleagues in adhesives and degradable networks.^[^ ^11^, ^65^ ^]^

## Conclusion

This study advances controlled FROMP, demonstrating significant strides in polymerization control and nonlinear front propagation. By systematically tuning inhibitor loadings and introducing anchimeric inhibitory monomers (monomers that inhibit upon incorporation), we achieved improved molecular weight control and dispersity, surpassing prior limitations. This control paves the way for developments in reactive manufacturing of copolymerization in both uncrosslinked and crosslinked systems.

Beyond improvements in control over polymerization, we harnessed FROMP to fabricate polymeric materials with both stepwise and gradient compositions, yielding spatially defined molecular weight distributions, patterning, and compositional variations—achievements that would be labor‐intensive or impossible via traditional polymer synthesis. This work positions FROMP as a powerful tool for next‐generation materials design, offering precise control without external intervention. These findings open new pathways for scalable, autonomous polymer manufacturing, merging the fields of controlled polymerization and chemically driven adaptive systems.

## Supporting Information

The authors have cited additional references within the Supporting Information.^[^ ^66^, ^67^ ^]^

## Conflict of Interests

The authors declare no conflict of interest.

## Supporting information



Supporting Information

Supporting Information

Supporting Information

Supporting Information

Supporting Information

Supporting Information

## Data Availability

The data that support the findings of this study are available in the Supporting Information of this article.
