# Supplementary material for: Molecular Weight Control in Frontal Ring‐Opening Metathesis Polymerization
Source: Angew Chem Int Ed Engl. 2025 Sep 13;64(44):e202510071. doi: 10.1002/anie.202510071 (PMC12559472; doi:10.1002/anie.202510071)
Supplement: Supplementary file 1 — Supporting Information [file ANIE-64-e202510071-s004.pdf]

Molecular Weight Control in Frontal Ring-Opening Metathesis Polymerization

Kevin A. Stewart, Darya A. Ivannikava, Claire M. Massouh, Jacob J. Lessard*

Department of Chemistry, University of Utah, Salt Lake City, Utah 84112, United States of America

E-mail: Jacob.lessard@utah.edu

Supporting Information:

Materials and Instrumentation
Experimental Procedures
FROMP of DCPD-H_2
Copolymerization
Gradient Materials
References

Materials and Instrumentation SI 3

Experimental Procedures SI 4

Nuclear Magnetic Resonance – SI 5

FROMP of DCPD-H_2 SI 14

Front Velocity and Timelapses – SI 16

Differential Scanning Calorimetry (DSC) Cure Kinetics – SI 31

Sample Images – SI 38

Size Exclusion Chromatography (SEC) Post-Cure – SI 42

NMR Spectroscopy Post-Cure – SI 50

DSC Post-Cure – SI 56

Copolymerization SI 66

Experimental Procedures – SI 66

Monomer Characterization – SI 66

Front Velocity and Timelapses – SI 72

DSC Cure Kinetics – SI 96

Sample Images – SI 108

SEC Post-Cure – SI 116

DSC Post-Cure – SI 130

NMR Post-Cure – SI 143

Gradient Polymer Materials SI 157

Dynamic Mechanical Analysis (DMA) Post-Cure SI 162

Tensile Testing SI 167

References SI 174

Materials:

Dicyclopentadiene (DCPD, 95%, Sigma), hydrobromic acid (48%, Sigma), cis-5-Norbornene-exo-2,3-dicarboxylic anhydride (98%, Oakwood), *n*-butylamine (MilliporeSigma), Grubbs' 2nd generation catalyst (G2, ChemScene), palladium on carbon (10% Pd, 50% Water, Accela) and tributyl phosphite (TBP, TCI chemicals, stored under N_2), diethyl ether (anhydrous, Fisher), tetrahydrofuran (THF, HPLC, OmniSolv), acetone (ACS, Fisher), methanol (ACS, Fisher), ethanol (200 proof, Decon), ethyl acetate (ACS, Fisher), and all other reagents were purchased commercially and used as received unless otherwise stated. Test tubes for frontal polymerizations were Fisherbrand 6x50 mm (inner diameter of ~4.75 mm, outer diameter ~6 mm) flint glass culture tubes or 7240 borosilicate self-fabricated tubes (inner diameter of ~5.0 mm, outer diameter ~7 mm) for the triple stacked sample.

Instrumentation:

Nuclear Magnetic Resonance (NMR):

Nuclear Magnetic Resonance (NMR) was preformed on a Bruker 500 with a cryoprobe or Bruker 300 MHz instrument using $CDCl_3$ as a solvent. NMR spectra were analyzed using MestreNova software.

Front Velocity Measurements:

Frontal polymerization was captured using a DSLR canon EOS R5 camera. The open-source physics (OSP) software package Tracker® was used to track the front location and calculate the average velocity.

Differential Scanning Calorimetry (DSC):

Differential scanning calorimetry (DSC) experiments were performed on a TA instruments Discovery DSC 250 instrument under nitrogen and analyzed using TA instruments Trios software. Cure kinetics were performed using 2-4 μL of reaction solution in sealed aluminum hermetic pans from 0 °C to 250 °C at a ramp rate of 10 °C min^{-1}. The exotherm values were obtained from integrating the peaks. Post cure experiments were run using 5-15 mg of sample in sealed aluminum pans from 0 °C to 250 °C at a ramp rate of 10 °C min^{-1}. Once at 250 °C, a 1 min isotherm was performed before the sample was cooled at 10 °C min^{-1} to 0 °C. The heating and cooling cycles were performed a total of 2 times. Glass transition temperatures were obtained as the mid-point in the depression and averaged over three separate samples.

Size Exclusion Chromatography (SEC):

Size exclusion chromatography (SEC) was performed on a Tosoh EcoSEC Elite equipped with temperature controlled dual pumps, 100 vial auto sampler, automated purge unit and degasser, column oven, 4.6x3.5 cm TSKgel guard column, 6x15 cm TSKgel SuperH-RC reference column, and 7.8x30 cm TSKgel GMH_{hr}-M in THF at 40 °C and a flow rate of 1 mL min^{-1} and 0.5 mL min^{-1} THF for the sample column and reference column, respectively. The system is equipped with temperature controlled refractive index, UV-8420 Detector, and LenS3 Multi-Angle Light Scattering Detector with measurement angles fixed at 10°, 90°, and 170°. Absolute molecular weight and dispersity (*Đ*) values were determined by multiangle light scattering from purified polymers refractive index increment (dn/dc) values assuming 100% mass recovery.

Density Measurements:

Density of polymer materials were determined using Mettler Toledo XPR105DR analytical balance coupled with a Mettler Toledo Archimedes kit using ethanol (**Figure S292**).

Dynamic Mechanical Analysis (DMA):

Dynamic mechanical analysis (DMA) was used to evaluate the thermomechanical properties of post-FROMP specimens. DMA experiments were performed on a TA Instruments Discovery DMA850 with a -90 °C chiller using supplied tensile grips under an inert atmosphere. Temperature ramp experiments were performed from 0 to 180 °C at a rate of 3 °C min^{-1} with a frequency of 1 Hz, an oscillating stress of 1000 Pa (0.001 MPa), and a preload force of 0.01 N. All experiments were analyzed using TA instruments Trios software.

Tensile Testing:

Tensile testing was performed to evaluate the mechanical properties of the post-FROMP specimens. Tensile experiments were performed on a ZwickRoelle Retroline load frame equipped with a 5 kN XforceP load cell, videoXtens 2-150 HP video extensometer, and 10 kN over-wedge grips (type 9304.03). All samples were prepared in a gasket U-mold with a 3 mm thickness at 45 °C in a vacuum oven then punched out using a Qualitest die (D-638-5-IMP-ASTM). Specimens were run with a starting preload force of 0.01 MPa and 5 mm/min at room temperature (~20 °C).

Experimental Procedures:

Synthesis of Hydrogenated Dicyclopentadiene (DCPD-H_2): Procedure Adapted from literature.[1]

Hydrobromination of Dicyclopentadiene (Norbornene Protection):

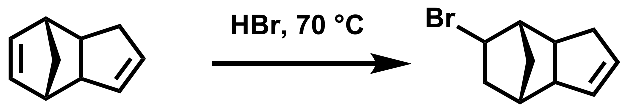

Dicyclopentadiene (DCPD; 326 g, 247 mmol, 1.00 equiv) was added to a 2000 mL round-bottomed flask. 500 mL of hydrobromic acid (HBr; 48% w/w; 518 mmol, 2.10 equiv) was added to the reaction vessel, a stir bar equipped, and the flask was sealed. The reaction flask was purged with N_2 for approximately 10 min. Then, the flask was placed in a preheated 70 °C oil bath and stirred for 22 h. The reaction mixture was then diluted with 1 L of deionized water and added to a 2 L separatory funnel. The aqueous mixture washed with diethyl ether (4 x 250 mL), and the combined organic layers were subsequently washed with saturated sodium bicarbonate (3 x 100 mL) and brine (2 x 100 mL). The organic layer was collected, dried with sodium sulfate, and concentrated via rotary evaporation. The crude oil was allowed to dry overnight under reduced pressure. The crude product was then vacuum distilled (600 mtorr, 125 °C) yielding a red, clear oil (476 g, 90.5% yield). **Preliminary optimization for subsequent steps were performed on the initial red colored DCPD-Br; however, a silica plug (100% hexanes mobile phase) should be performed on the distilled product prior to catalytic hydrogenation of DCPD-Br* (see **Figure S2**).

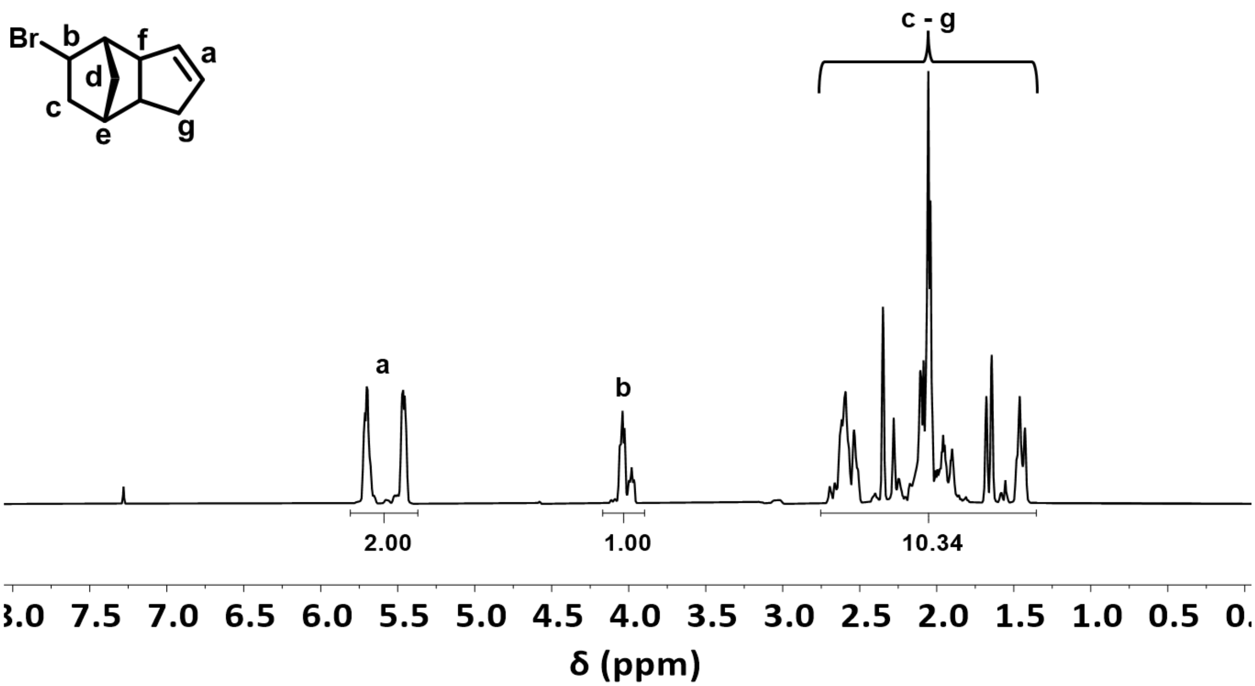

Figure S1: ^{1}H-NMR of bromo dicyclopentadiene (Br-DCPD).

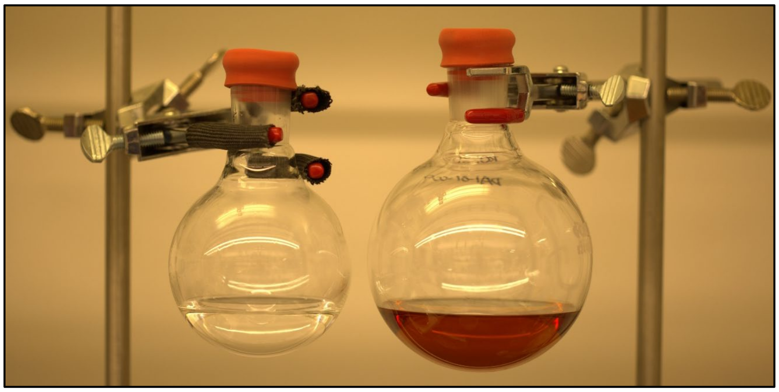

Figure S2: Image of bromo-DCPD before (right) and after (left) column chromatography. Little no to no change in NMR trace was observed, but the purified (left) drastically improved the efficiency next step (**Figure S5**).

Hydrogenation of Bromo-dicyclopentadiene (Br-DCPD):

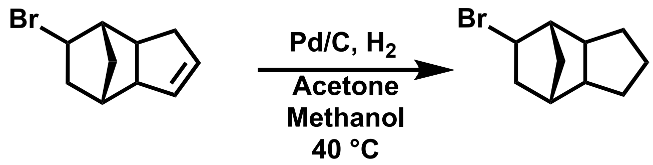

[50 g scale] Hydrogenation of Br-DCPD in 100% acetone (**no** silica purification)

Br-DCPD (50.1 g, 235 mmol) was added to a 250 round-bottomed flask and dispersed in 60 mL of acetone. 10% Pd/C (1.21 g) was added to the Br-DCPD solution, a stir bar was equipped, and the reaction vessel was sealed. While stirring, the reaction vessel was purged with N_2 for 1 min followed by vacuum until the solvent began to bubble (repeated 5 times). Next, three triple-wrapped balloons filled with H_2 were added and the reaction vessel was added to a 40 °C preheated oil bath for 72 h with H_2 balloons backfilled every 12 h. After 72 h, the slurry was vacuum eluted through a celite/silica plug and the plug was washed with acetone (8 x 50 mL). The filtrate was collected, transferred to a round-bottomed flask, and concentrated via rotary evaporation. The crude product was then purified with a silica plug (100% hexanes mobile phase). The eluent was concentrated via rotary evaporation and dried overnight under reduced pressure. ^{1}H NMR analysis indicated 71% conversion to the reduced product following one round of hydrogenation (**Figure S3**).

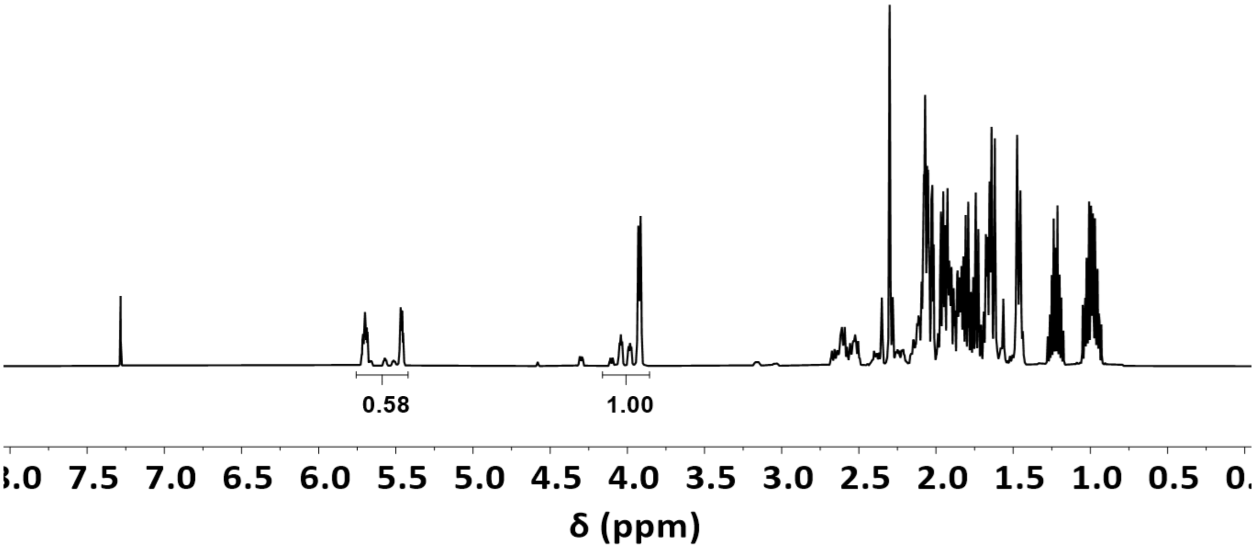

Figure S3: ^{1}H-NMR of partially hydrogenated bromo-dicyclopentadiene (Br-DCPD-H_2) performed at a 50 g scale following one round of catalytic hydrogenation in 100% acetone and no prior silica plug purification.

[50 g scale] Hydrogenation of Br-DCPD in 2:1 acetone/methanol (**no** silica purification)

The above procedure was performed at identical scale; however, a 2:1 acetone/methanol solvent mixture (40 mL and 20 mL, respectively) was utilized, yielding 91% conversion to the reduced product following one round of hydrogenation (**Figure S4**). The product mixture was subjected to a second round of hydrogenation following identical conditions, yielding 100% conversion of the double bond to hydrogenated bromo-dicyclopentadiene (Br-DCPD-H_2; 38.8 g, 76.8% yield).

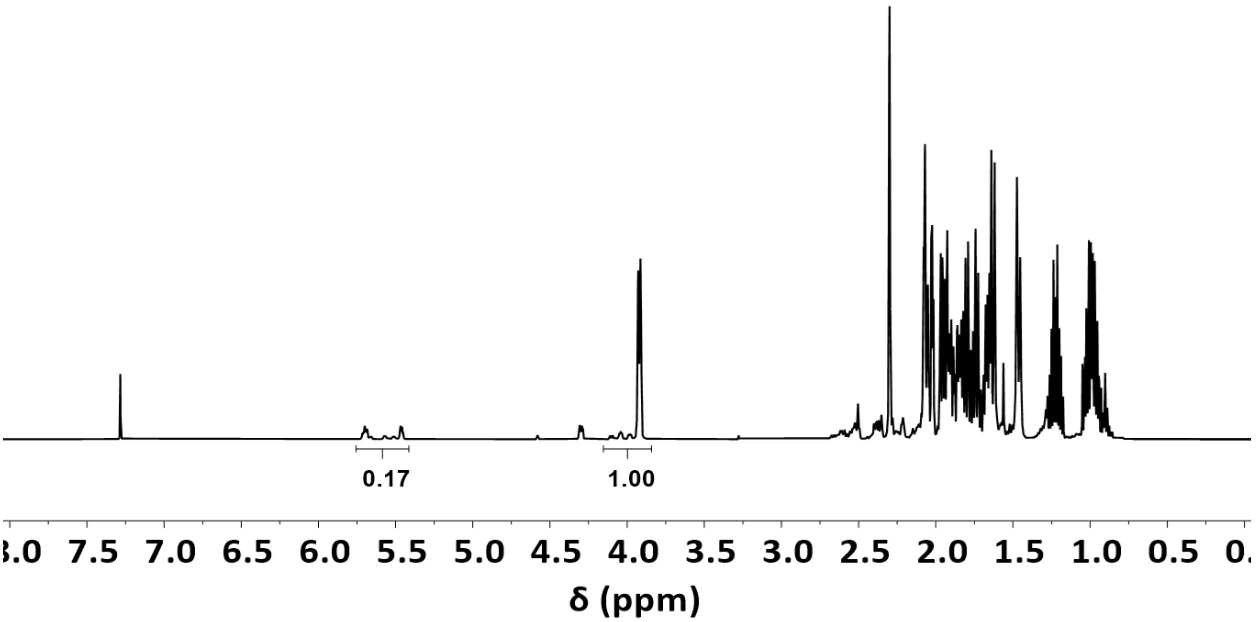

Figure S4: ^{1}H-NMR of partially hydrogenated bromo-dicyclopentadiene (Br-DCPD-H_2) performed at a 50 g scale following one round of catalytic hydrogenation in 2:1 acetone/methanol and no prior silica plug purification.

[50 g scale] Hydrogenation of Br-DCPD in 2:1 acetone/methanol (**with** silica purification)

The procedure was performed at identical scale and solvent parameters; however, the Br-DCPD precursor was purified with a silica plug (100% hexanes mobile phase) prior to catalytic hydrogenation. The modified conditions yielded 100% conversion to the reduced product following one round of hydrogenation (47.9 g, 95.0% yield) (**Figure S5**).

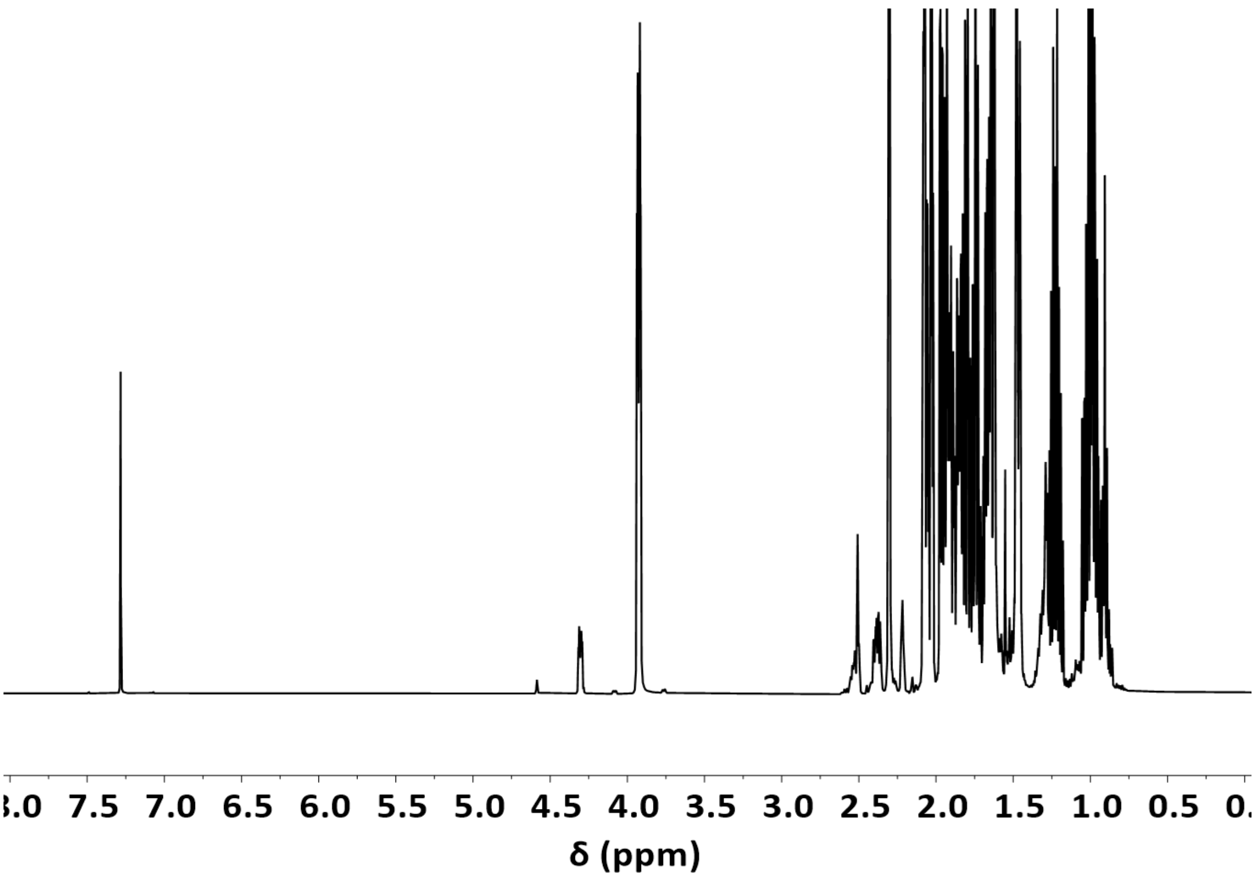

Figure S5: ^{1}H-NMR of fully hydrogenated bromo-dicyclopentadiene (Br-DCPD-H_2) performed at a 50 g scale following one round of catalytic hydrogenation in 2:1 acetone/methanol and prior silica plug purification.

[100 g scale] Hydrogenation of Br-DCPD in 2:1 acetone/methanol (**with** silica purification)

Upon identifying optimized conditions (*vide supra*), the hydrogenation was performed at twice the scale. Br-DCPD (100. g, 469 mmol) was added to a 500 mL round-bottomed flask and dispersed in 120 mL of 2:1 acetone/methanol solvent mixture. 10% Pd/C (2.25 g) was added to the Br-DCPD solution, a stir bar was equipped, and the reaction vessel was sealed. While stirring, the reaction vessel was purged with N_2 for 1 min followed by vacuum until the solvent began to bubble (repeated 5 times). Next, three triple-wrapped balloons filled with H_2 were added and the reaction vessel was added to a 40 °C preheated oil bath for 96 h with H_2 balloons backfilled every 12 h. After 96 h, the slurry was vacuum eluted through a celite/silica plug and the plug was washed with acetone (8 x 50 mL). The filtrate was collected, transferred to a round-bottomed flask, and concentrated via rotary evaporation. The crude product was then purified with a silica plug (100% hexanes mobile phase). The eluent was concentrated via rotary evaporation and dried overnight under reduced pressure. ^{1}H NMR analysis indicated 100% conversion to the reduced product following one round of hydrogenation (97.6 g, 96.3% yield) (**Figure S6**).

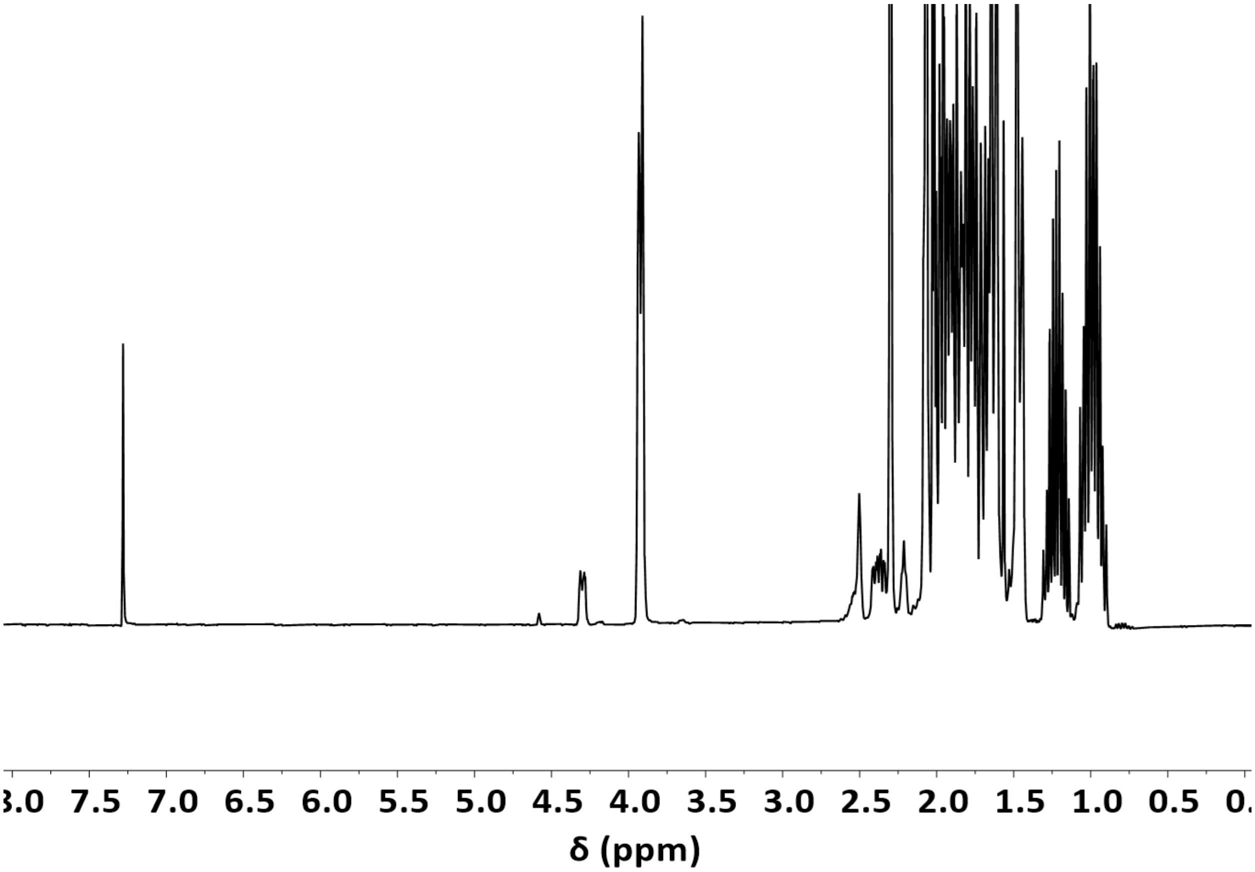

Figure S6: ^{1}H-NMR of fully hydrogenated bromo-dicyclopentadiene (Br-DCPD-H_2) performed at a 100 g scale following one round of catalytic hydrogenation in 2:1 acetone/methanol and prior silica plug purification.

[300 g scale] Hydrogenation of Br-DCPD in 2:1 acetone/methanol (**with** silica purification)

Br-DCPD (315. g, 1.48 mol) was added to a 1000 mL round-bottomed flask and dispersed in 375 mL of 2:1 acetone/methanol solvent mixture. 10% Pd/C (6.94 g) was added to the Br-DCPD solution, a stir bar was equipped, and the reaction vessel was sealed. While stirring, the reaction vessel was purged with N_2 for 1 min followed by vacuum until the solvent began to bubble (repeated 5 times). Next, four triple-wrapped balloons filled with H_2 were added and the reaction vessel was added to a 40 °C preheated oil bath for 96 h with H_2 balloons backfilled every 12 h. After 96 h, the slurry was vacuum eluted through a celite/silica plug and the plug was washed with acetone (8 x 50 mL). The filtrate was collected, transferred to a round-bottomed flask, and concentrated via rotary evaporation. The crude product was then purified with a silica plug (100% hexanes mobile phase). The eluent was concentrated via rotary evaporation and dried overnight under reduced pressure. ^{1}H NMR analysis indicated 99.0% conversion to the reduced product following one round of hydrogenation (**Figure S7**). The isolated Br-DCDPD-H_2 was resubjected to a second round of catalytic hydrogenation using the same volume of 2:1 acetone/methanol but a reduced amount of 10% Pd/C (3.210 g). Four triple-wrapped balloons filled with H_2 were added and the reaction vessel was added to a 40 °C preheated oil bath for 60 h with H_2 balloons backfilled every 12 h. Following and identical work-up and purification, ^{1}H NMR analysis indicated

100% conversion to the reduced product following one round of hydrogenation (302 g, 95.1% yield) (**Figure S8**).

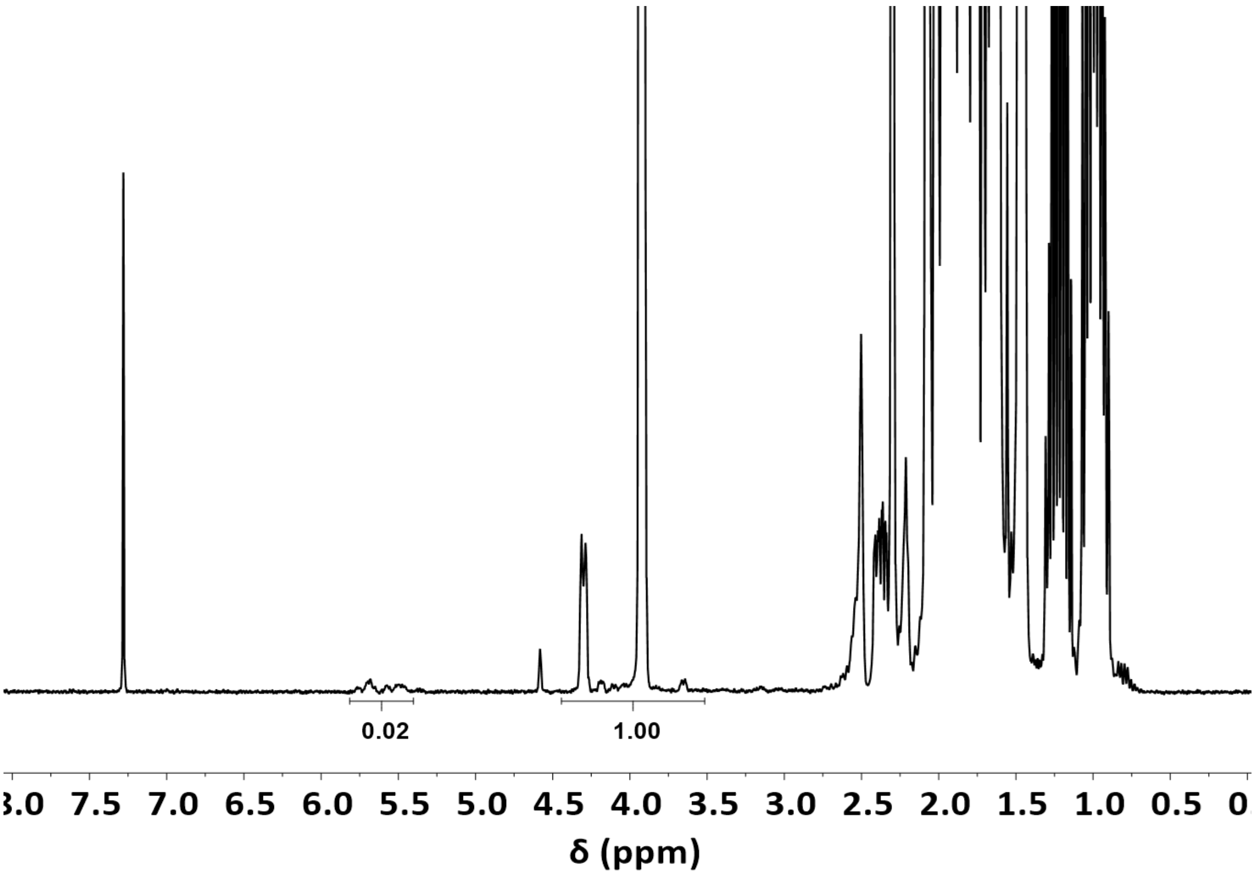

Figure S7: ^{1}H-NMR of near-quantitative hydrogenation of bromo-dicyclopentadiene (Br-DCPD-H_2) performed at a 300 g scale following one round of catalytic hydrogenation in 2:1 acetone/methanol and prior silica plug purification.

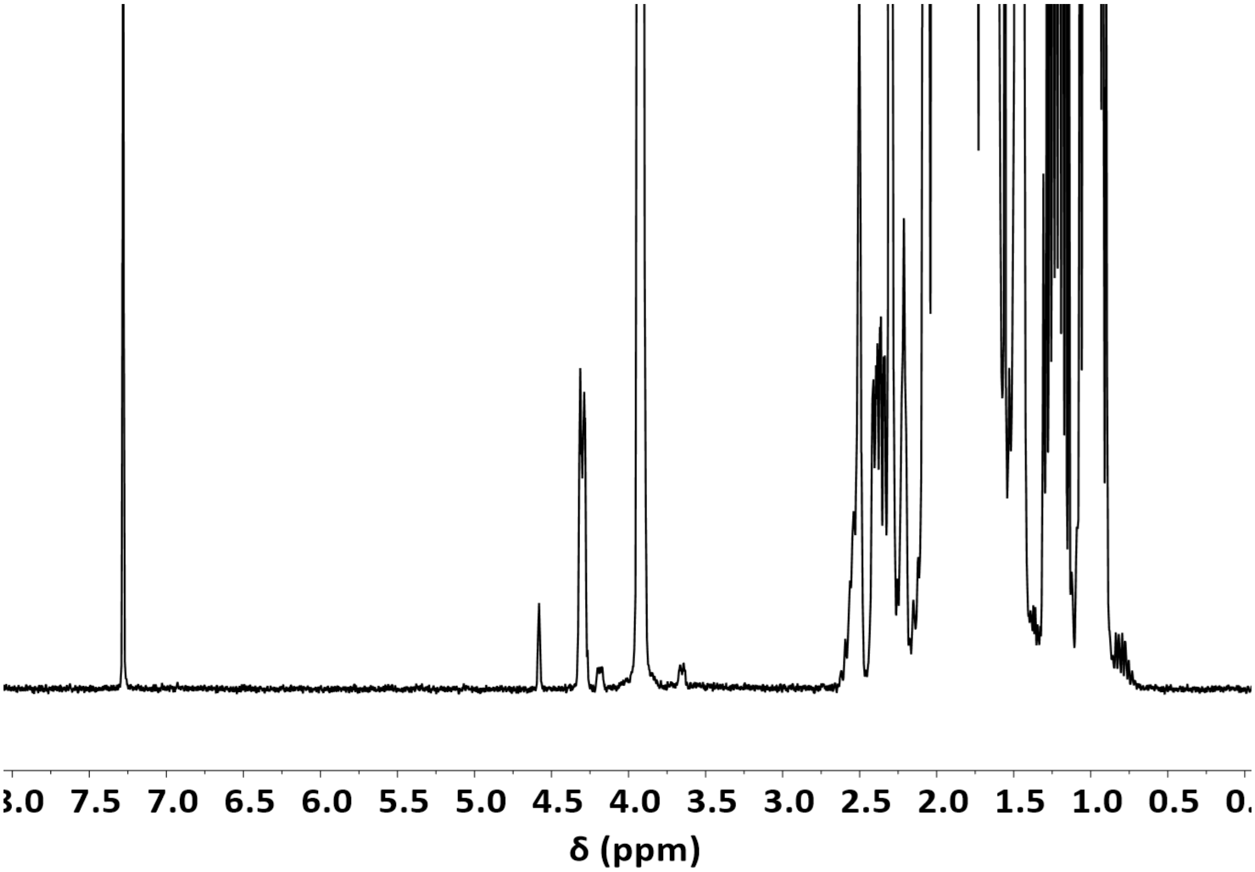

Figure S8: ^{1}H-NMR of quantitative hydrogenation of bromo-dicyclopentadiene (Br-DCPD-H_2) performed at a 300 g scale following a second round of catalytic hydrogenation in 2:1 acetone/methanol and prior silica plug purification.

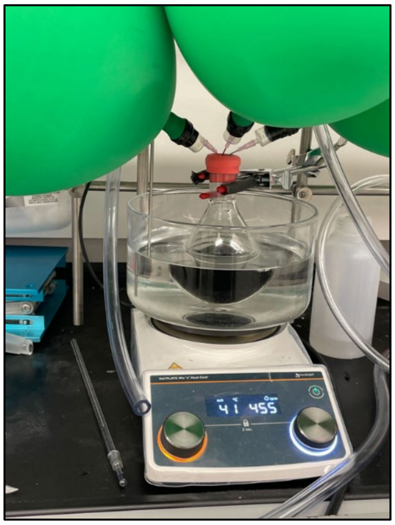

Figure S9: Image of Br-DCPD hydrogenation setup (50 g scale).

Elimination of Hydrogenated Bromo-dicyclopentadiene (Br-DCPD-H_2):

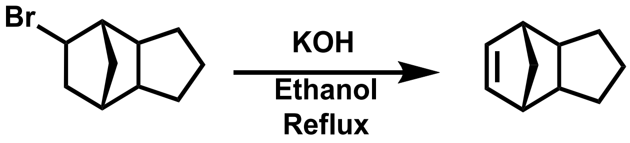

Hydrogenated bromo-dicyclopentadiene (Br-DCPD-H_2; 302 g, 1.41 mol, 1.00 equiv) in a 2 L round-bottomed flask was dispersed in 900 mL of absolute (200 proof) ethanol. Next, potassium hydroxide pellets (206 g, 3.65 mol, 2.60 equiv) was added to the Br-DCPD-H_2 solution. A stir bar and reflux condenser was equipped, and the reaction mixture was stirred in a 100 °C preheated oil bath for 36 h. The reaction vessel was then allowed to cool to room temperature and was diluted with 1 L of deionized water. In two separate batches, the diluted solution was added to a 2 L separatory funnel and extracted with diethyl ether (3 x 300 mL). The two separate organic washes were combined, added to the separatory funnel, and washed with deionized water (2 x 500 mL) and brine (2 x 500 mL). The organic layer was collected, dried with sodium sulfate, concentrated via rotary evaporation, and dried overnight under reduced pressure to give a clear, bright yellow oil. This crude product was vacuum distilled (40 °C, 600 mtorr) yielding a clear, colorless oil (158 g, 84.0% yield).

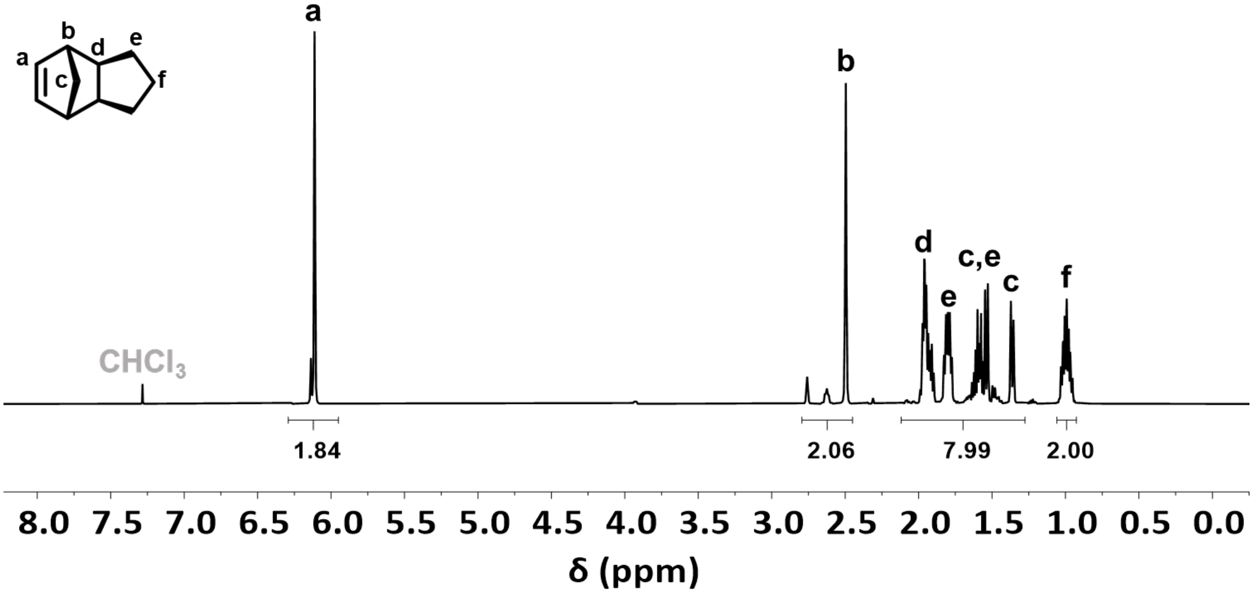

Figure S10: ^{1}H-NMR of hydrogenated dicyclopentadiene (DCPD-H_2).

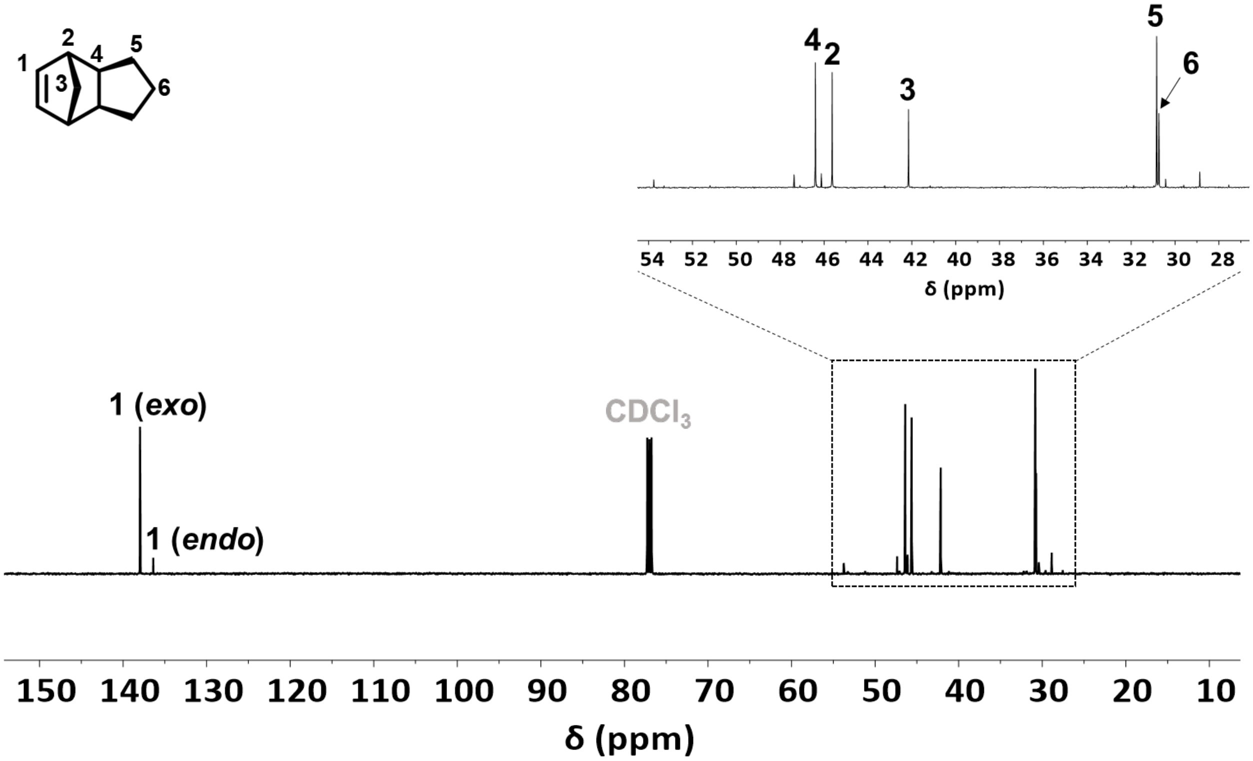

Figure S11: ^{13}C-NMR of hydrogenated dicyclopentadiene (DCPD-H_2).

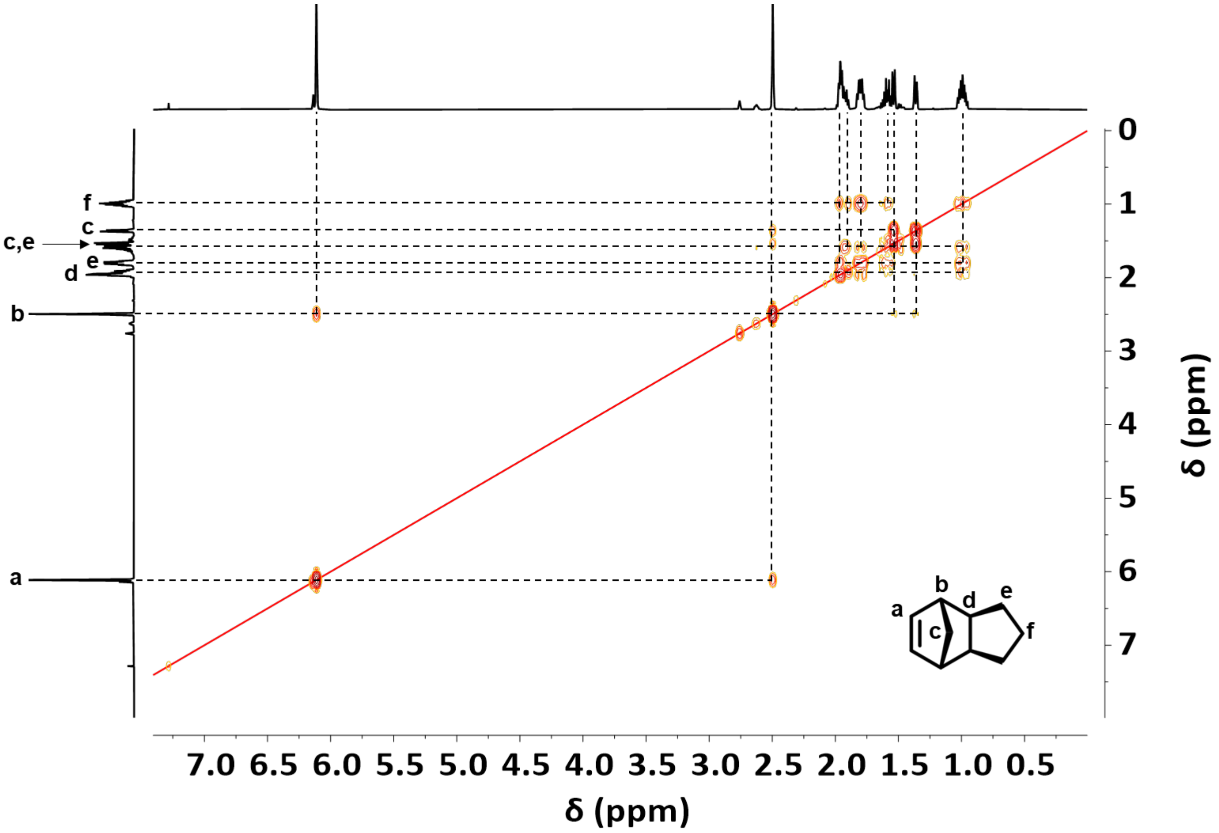

Figure S12: COSY NMR of hydrogenated dicyclopentadiene (DCPD-H_2).

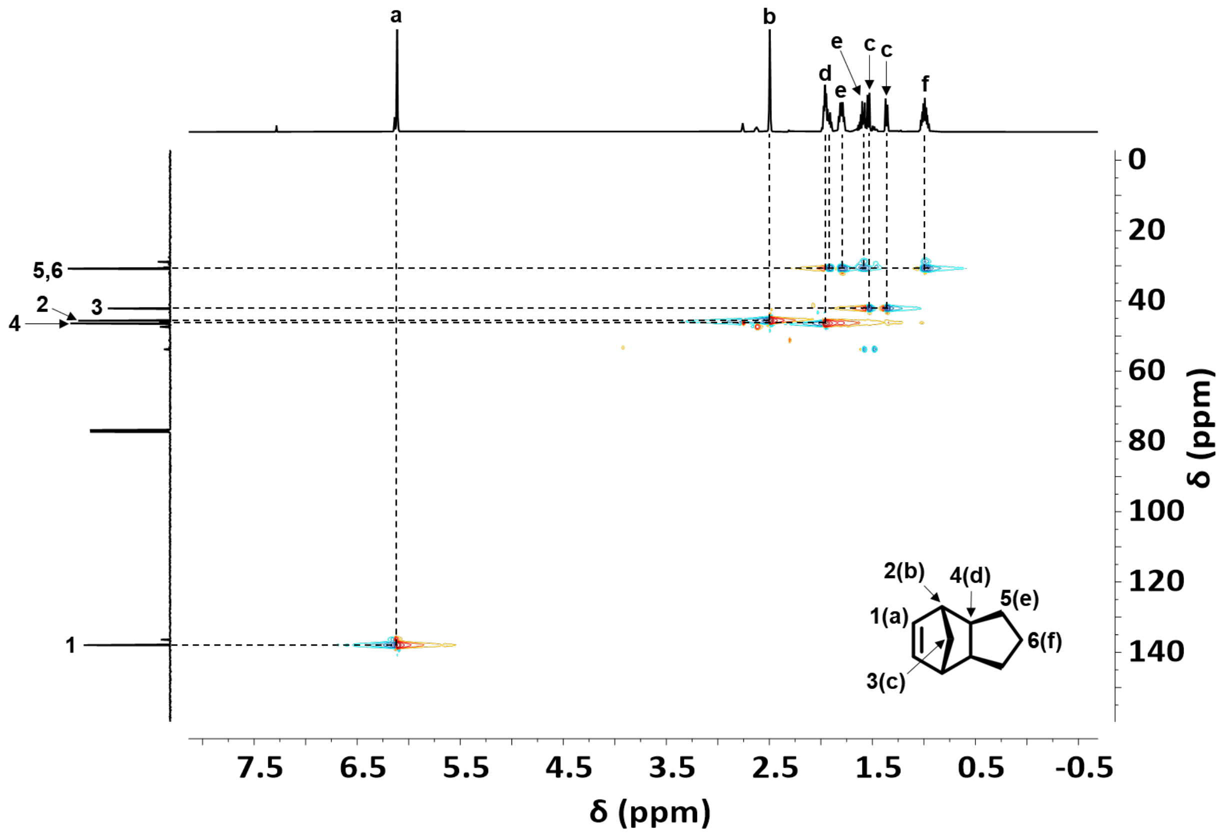

Figure S13: HSQC NMR of hydrogenated dicyclopentadiene (DCPD-H₂).

General Procedure for DCPD-H2 Resin Preparation:

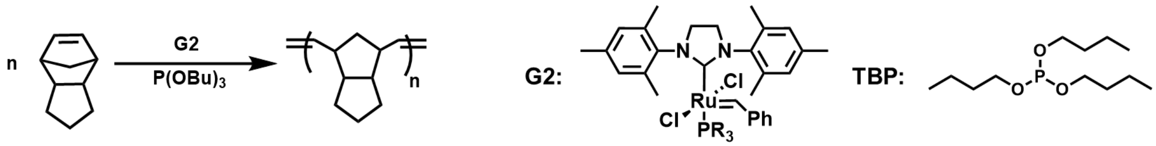

Grubbs' 2nd generation initiator was massed (G2, **w** mg, 1.00 equiv.) in a 5 mL vial prior to the addition of tributyl phosphite (TBP, **x** μL, **y** equiv.). The mixture was dissolved in monomer (*DCPD-H₂*, 500 mg, **z** equiv.) and sonicated for up to 5 minutes. The resulting solution was transferred a test tube. Test tube samples were initiated at the top or bottom of the resin using a preheated sottering iron creating a descending front.

4000:1:1 Monomer:Initiator:Inhibitor

G2: **w** = 0.79 mg

TBP: **x** = 0.25 μL, **y** = 1.00 equiv.

DCPD-H₂: **z** = 4,000 equiv.

2000:1:1 Monomer:Initiator:Inhibitor

G2: **w** = 1.58 mg

TBP: **x** = 0.50 μL, **y** = 1.00 equiv.

DCPD-H_2: **z** = 2,000 equiv.

<u>1000:1:1 Monomer:Initiator:Inhibitor</u>

G2: **w** = 3.17 mg

TBP: **x** = 1.0 μL, **y** = 1.00 equiv.

DCPD-H_2: **z** = 1,000 equiv.

<u>500:1:1 Monomer:Initiator:Inhibitor</u>

G2: **w** = 6.33 mg

TBP: **x** = 2.0 μL, **y** = 1.00 equiv.

DCPD-H_2: **z** = 500 equiv.

<u>4000:1:10 Monomer:Initiator:Inhibitor</u>

G2: **w** = 0.79 mg

TBP: **x** = 2.5 μL, **y** = 10.0 equiv.

DCPD-H_2: **z** = 4,000 equiv.

<u>2000:1:10 Monomer:Initiator:Inhibitor</u>

G2: **w** = 1.58 mg

TBP: **x** = 5.1 μL, **y** = 10.0 equiv.

DCPD-H_2: **z** = 2,000 equiv.

<u>1000:1:10 Monomer:Initiator:Inhibitor</u>

G2: **w** = 3.17 mg

TBP: **x** = 10.2 μL, **y** = 10.0 equiv.

DCPD-H_2: **z** = 1,000 equiv.

<u>500:1:10 Monomer:Initiator:Inhibitor</u>

G2: **w** = 6.33 mg

TBP: **x** = 20.3 μL, **y** = 10.0 equiv.

DCPD-H_2: **z** = 500 equiv.

<u>200:1:10 Monomer:Initiator:Inhibitor</u>

G2: **w** = 15.82 mg

TBP: **x** = 50.7 μL, **y** = 10.0 equiv.

DCPD-H_2: **z** = 200 equiv.

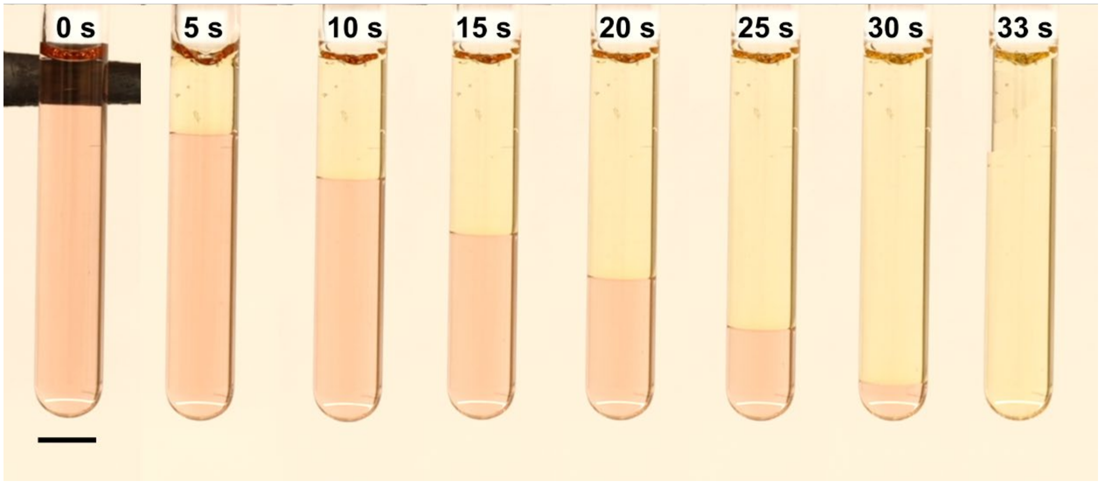

Figure S14: Representative timelapse of 4000:1:1 DCPD-H_2:G2:TBP. The scale bar is 5 mm.

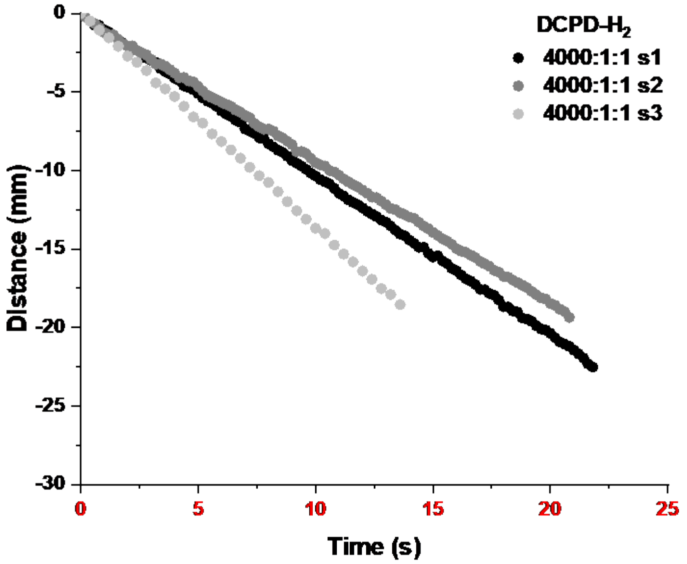

Figure S15: Front tracking of 4000:1:1 DCPD-H_2:G2:TBP in triplicate (v_f = 1.1 ± 0.2 mm/s).

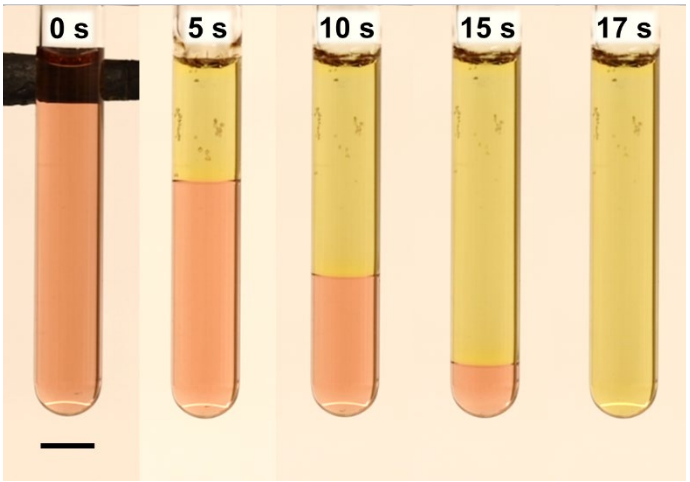

Figure S16: Representative timelapse of 2000:1:1 DCPD-H_2:G2:TBP. The scale bar is 5 mm.

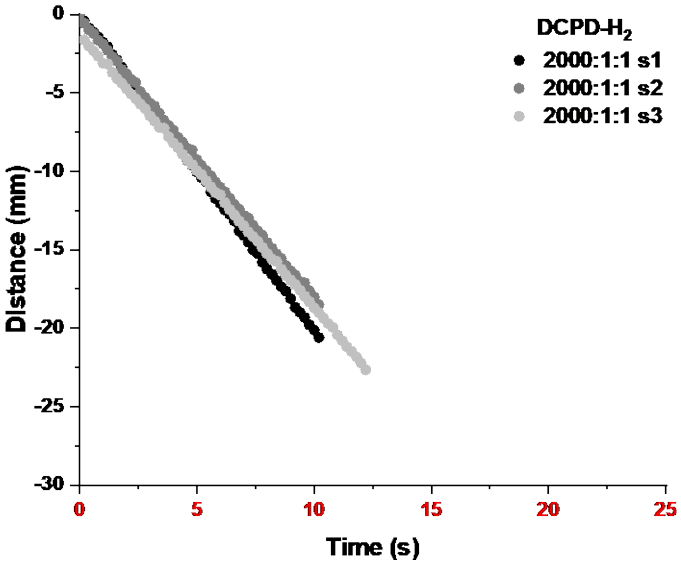

Figure S17: Front tracking of 2000:1:1 DCPD-H_2:G2:TBP in triplicate (v_f = 1.9 ± 0.1 mm/s).

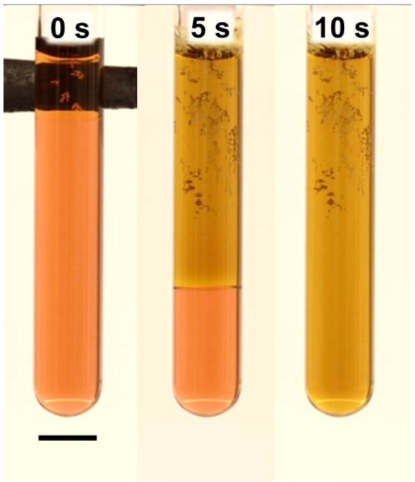

Figure S18: Representative timelapse of 1000:1:1 DCPD-H_2:G2:TBP. The scale bar is 5 mm.

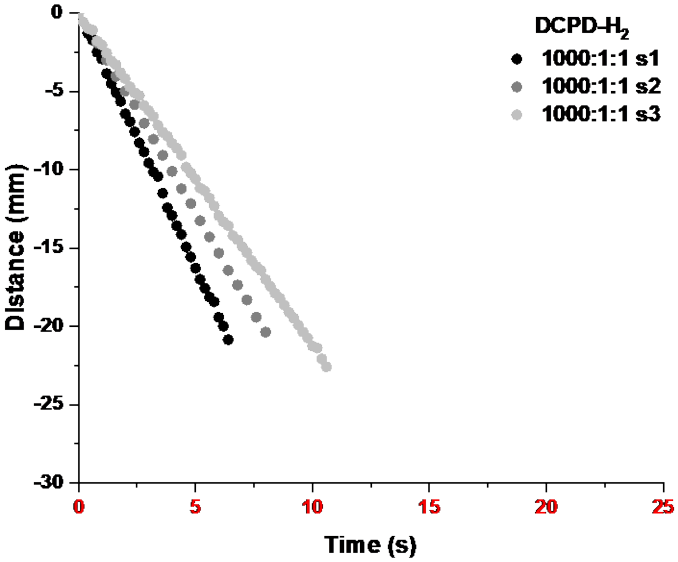

Figure S19: Front tracking of 1000:1:1 DCPD-H_2:G2:TBP in triplicate (v_f = 2.7 ± 0.5 mm/s).

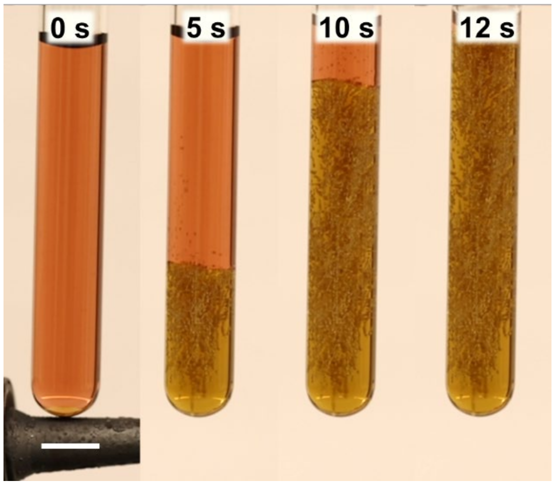

Figure S20: Representative timelapse of 1000:1:1 DCPD-H_2:G2:TBP **bottom up**. The scale bar is 5 mm.

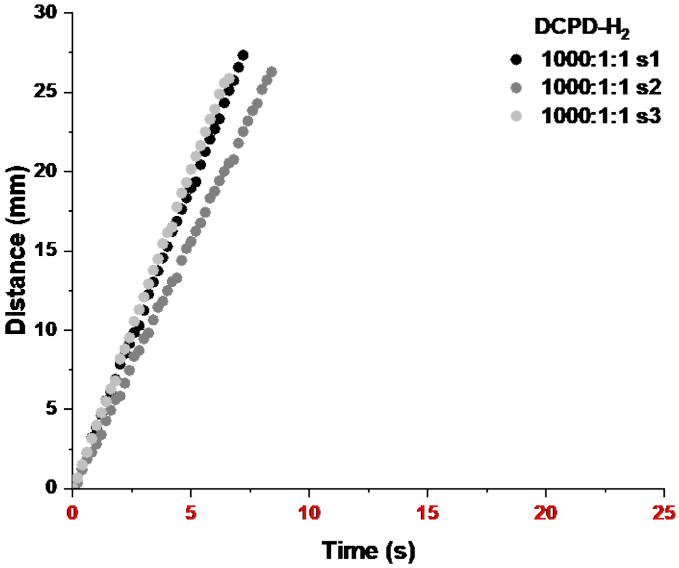

Figure S21: Front tracking of 1000:1:1 DCPD-H_2:G2:TBP in triplicate **bottom up** (v_f = 3.7 ± 0.4 mm/s).

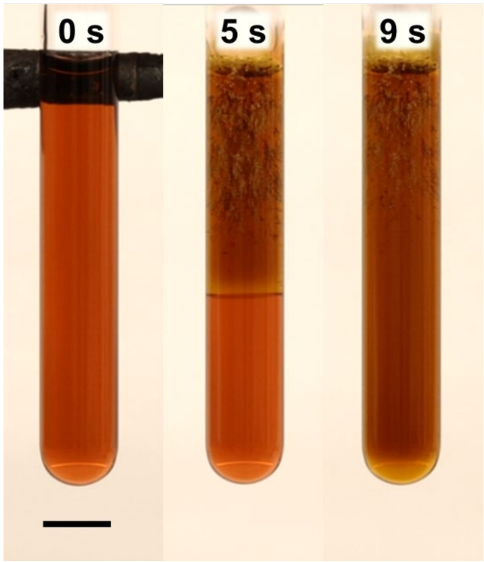

Figure S22: Representative timelapse of 500:1:1 DCPD-H_2:G2:TBP. The scale bar is 5 mm.

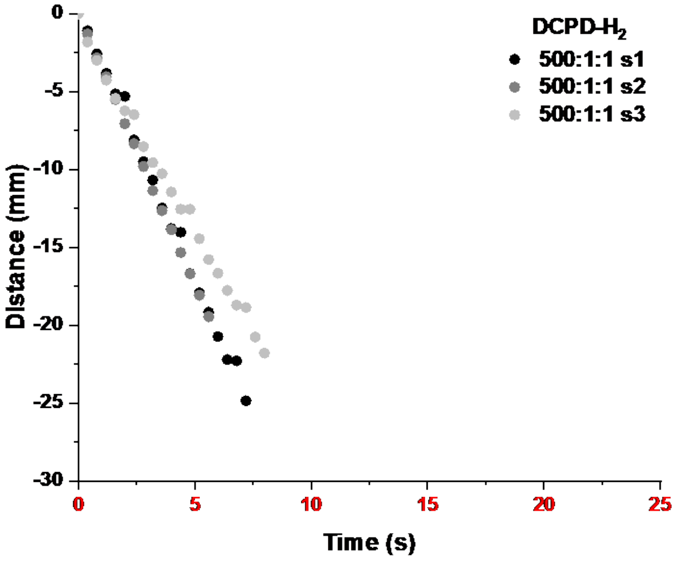

Figure S23: Front tracking of 500:1:1 DCPD-H_2:G2:TBP in triplicate (v_f = 2.7 ± 0.5 mm/s).

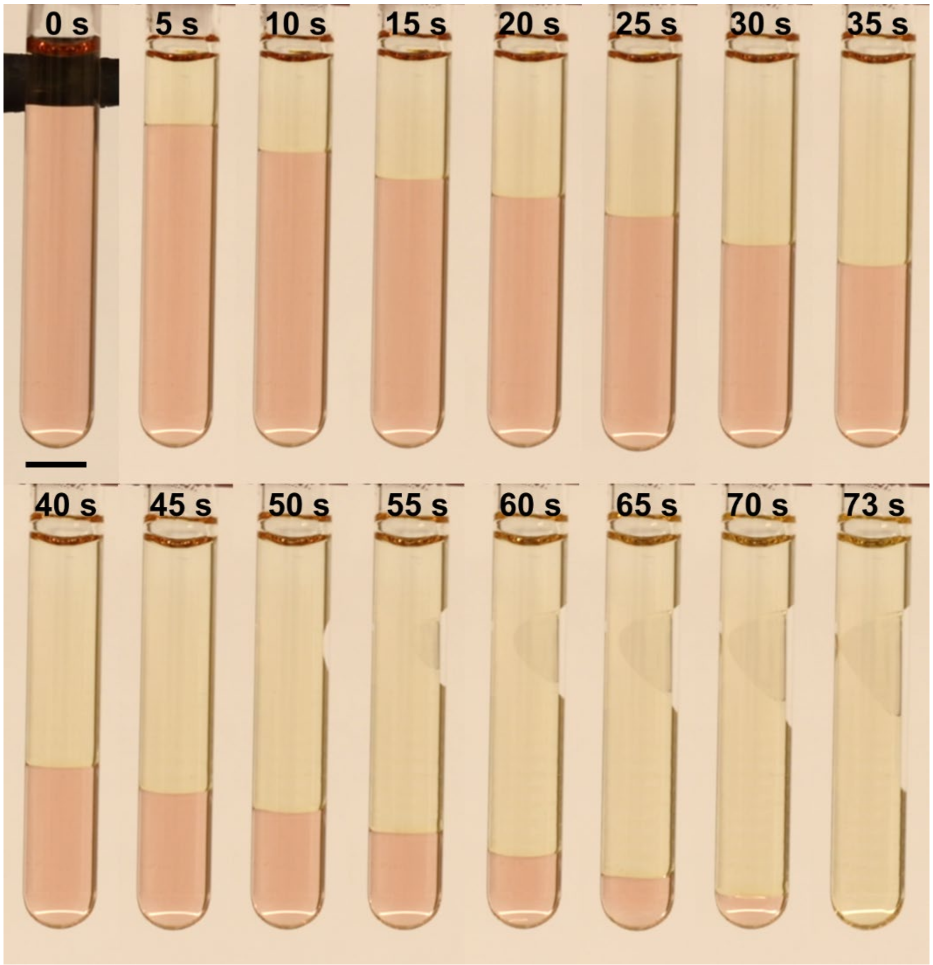

Figure S24: Representative timelapse of 4000:1:10 DCPD-H_2:G2:TBP. The scale bar is 5 mm.

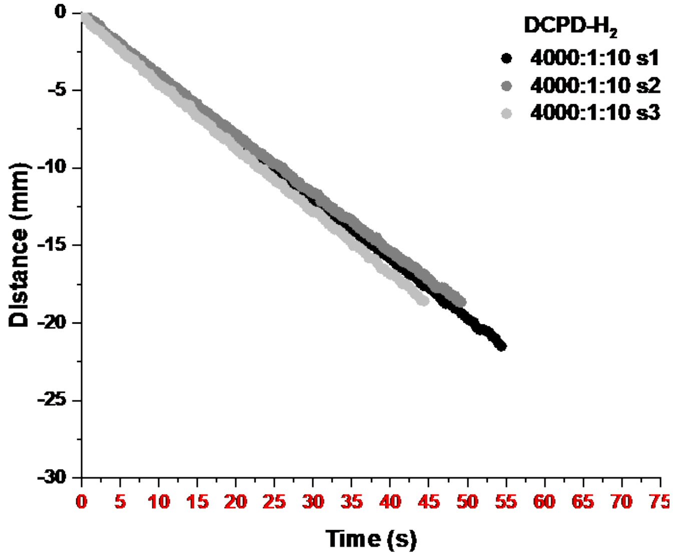

Figure S25: Front tracking of 4000:1:10 DCPD-H_2:G2:TBP in triplicate (v_f = 0.39 ± 0.01 mm/s).

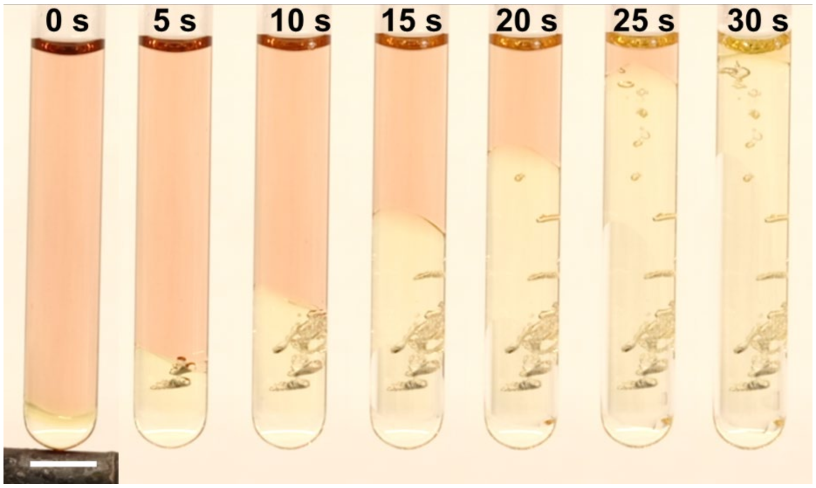

Figure S26: Representative timelapse of 4000:1:10 DCPD-H_2:G2:TBP **bottom up**. The scale bar is 5 mm.

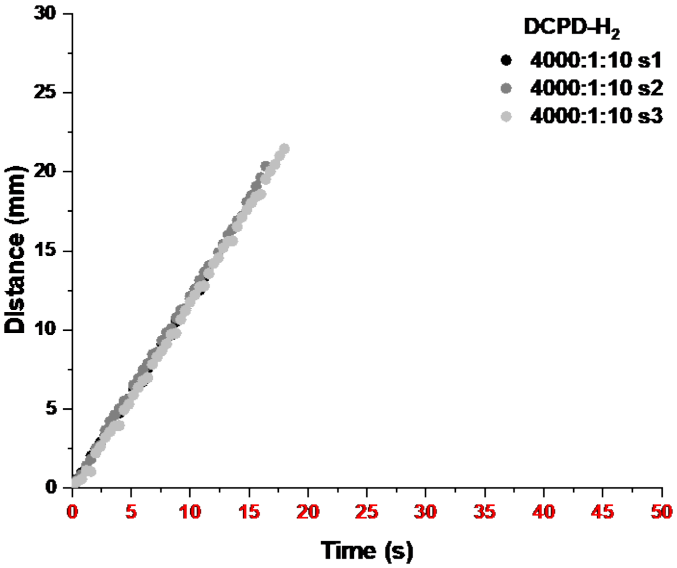

Figure S27: Front tracking of 4000:1:10 DCPD-H_2:G2:TBP in triplicate **bottom up** (v_f = 1.20 ± 0.01 mm/s).

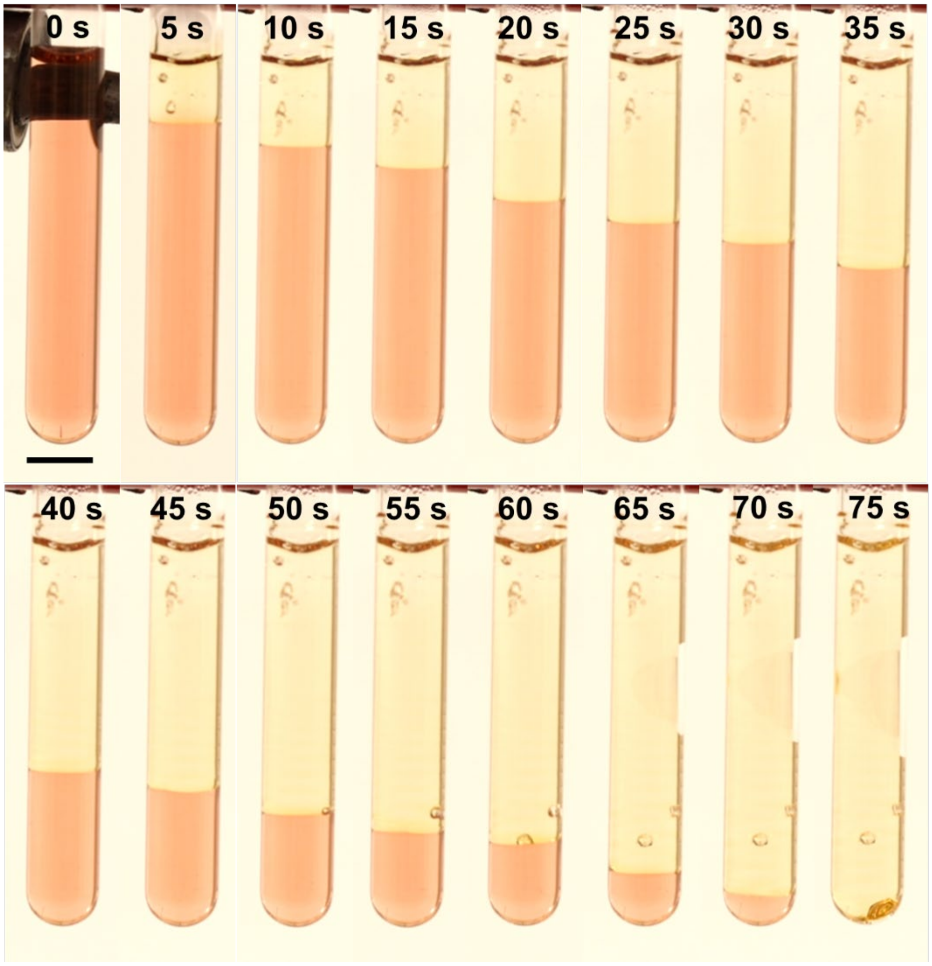

Figure S28: Representative timelapse of 2000:1:10 DCPD-H_2:G2:TBP. The scale bar is 5 mm.

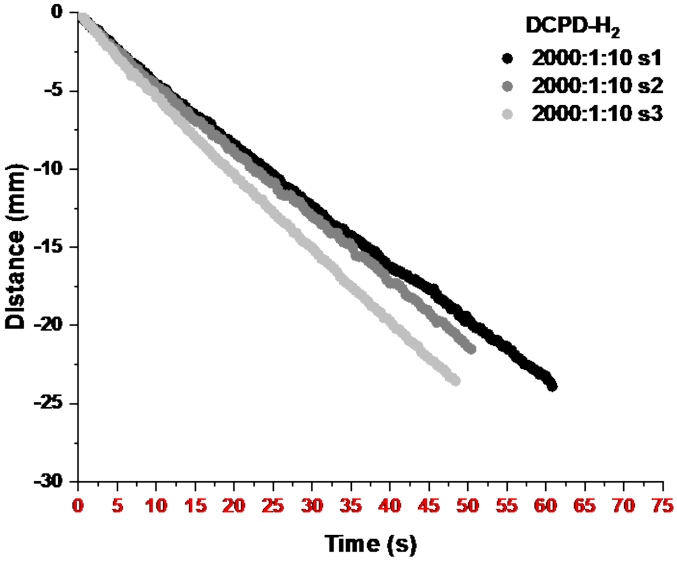

Figure S29: Front tracking of 2000:1:10 DCPD-H_2:G2:TBP in triplicate (v_f = 0.43 ± 0.04 mm/s).

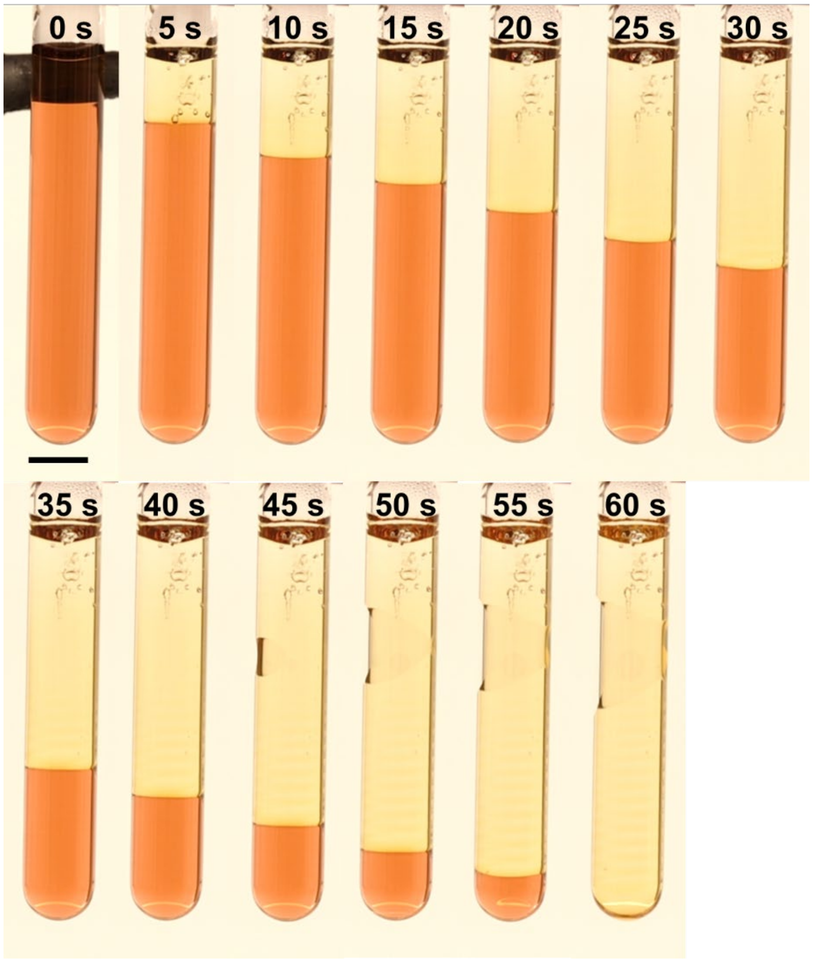

Figure S30: Representative timelapse of 1000:1:10 DCPD-H_2:G2:TBP. The scale bar is 5 mm.

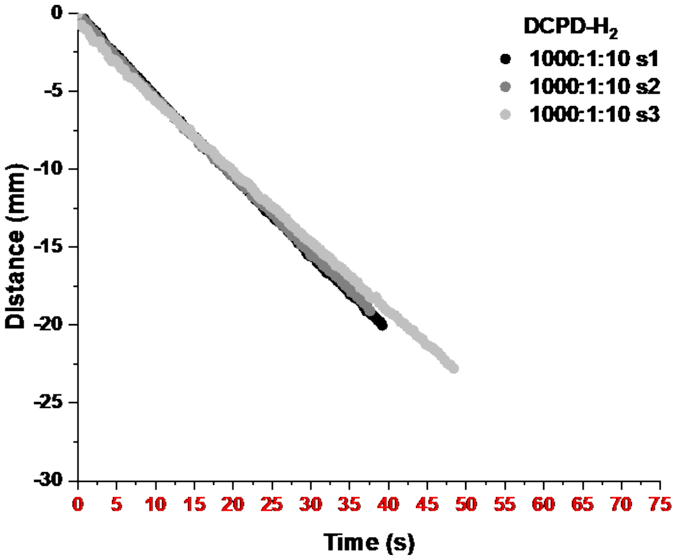

Figure S31: Front tracking of 1000:1:10 DCPD-H_2:G2:TBP in triplicate (v_f = 0.49 ± 0.02 mm/s).

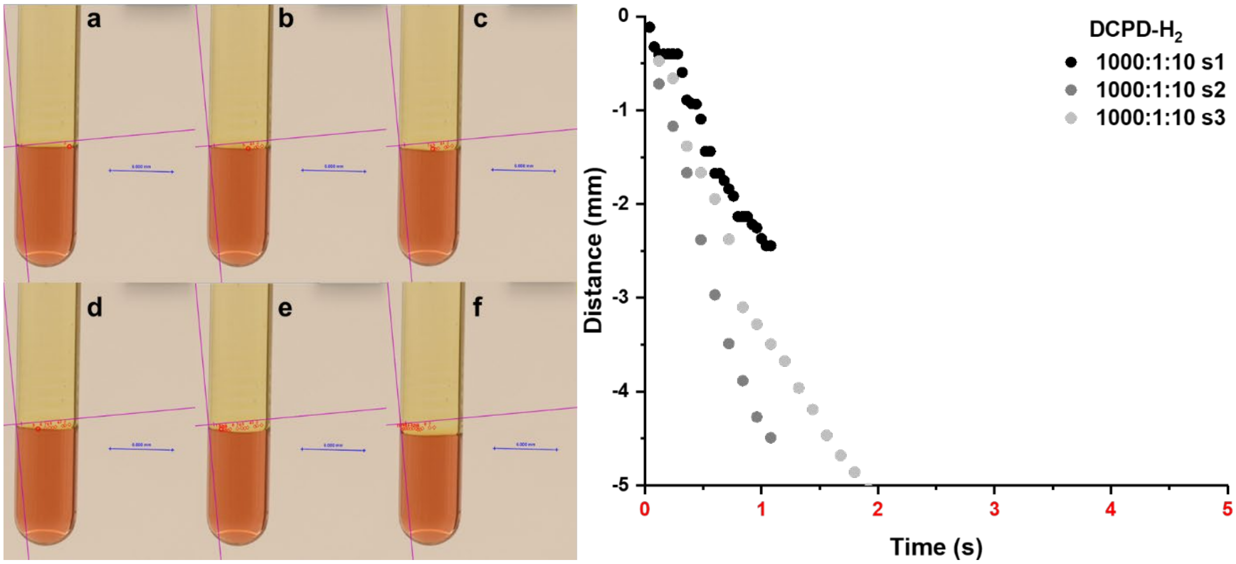

Figure S32: Representative timelapse of front tracking workflow and results for 1000:1:10 DCPD-H_2:G2:TBP patterned/across in triplicate ($v_{f,\,pattern}$ = 0.33 ± 0.07 mm/s).

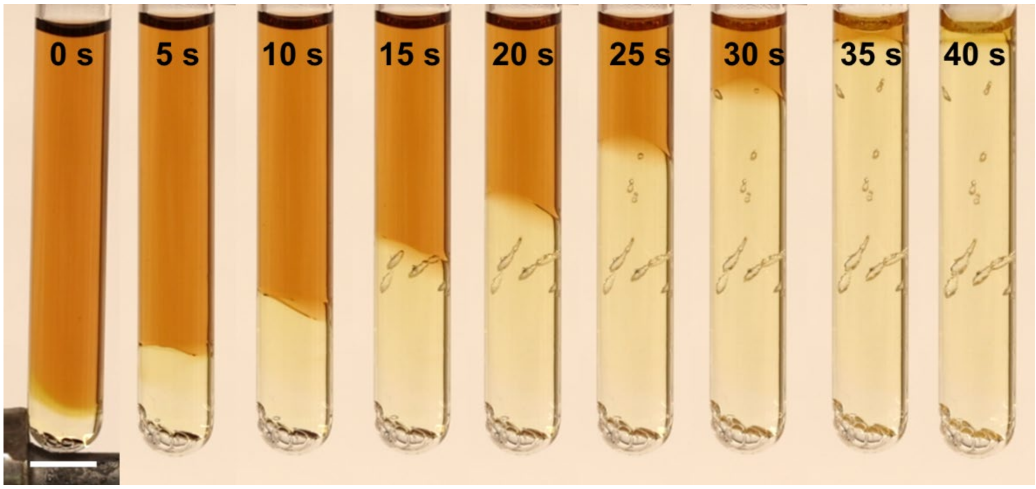

Figure S33: Representative timelapse of 1000:1:10 DCPD-H_2:G2:TBP **bottom up**. The scale bar is 5 mm.

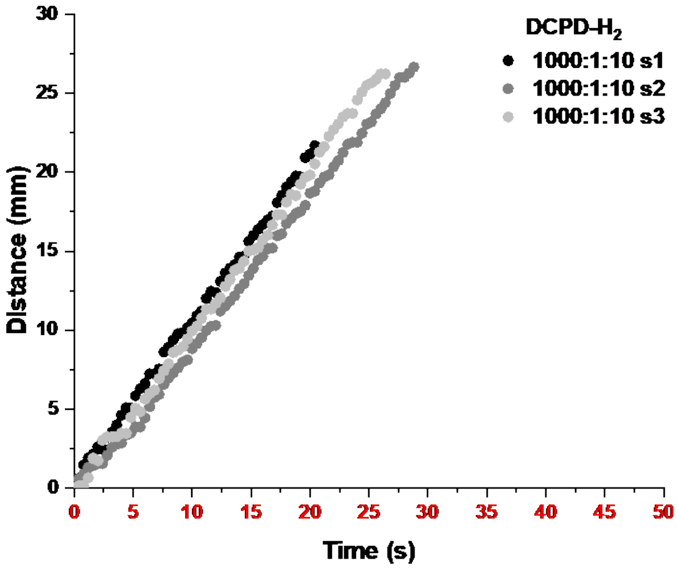

Figure S34: Front tracking of 1000:1:10 DCPD-H_2:G2:TBP in triplicate **bottom up** (v_f = 1.00 ± 0.04 mm/s).

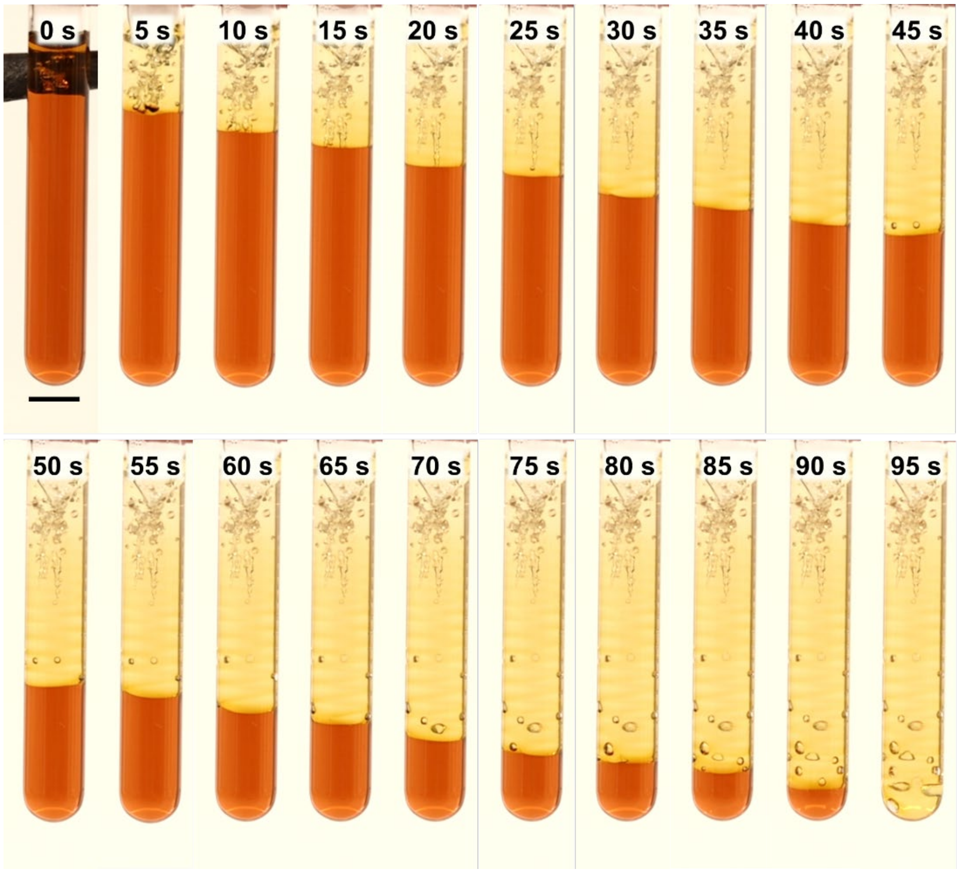

Figure S35: Representative timelapse of 500:1:10 DCPD-H_2:G2:TBP. The scale bar is 5 mm.

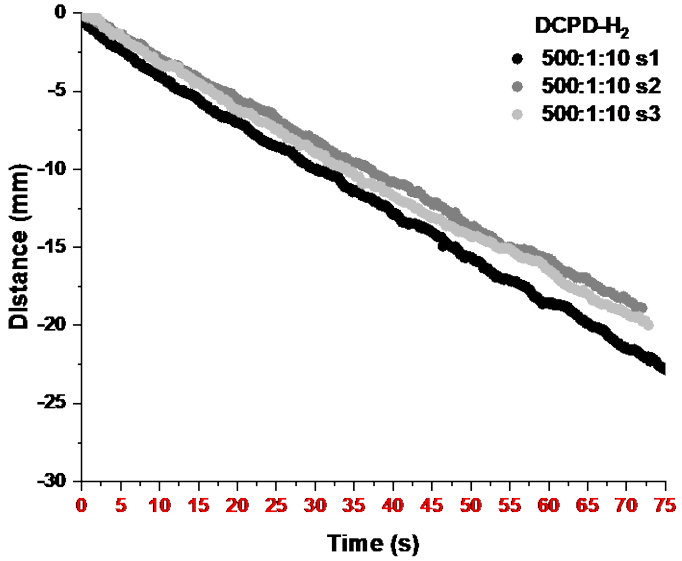

Figure S36: Front tracking of 500:1:10 DCPD-H_2:G2:TBP in triplicate ($v_{f,\ global}$ = 0.28 ± 0.01 mm/s).

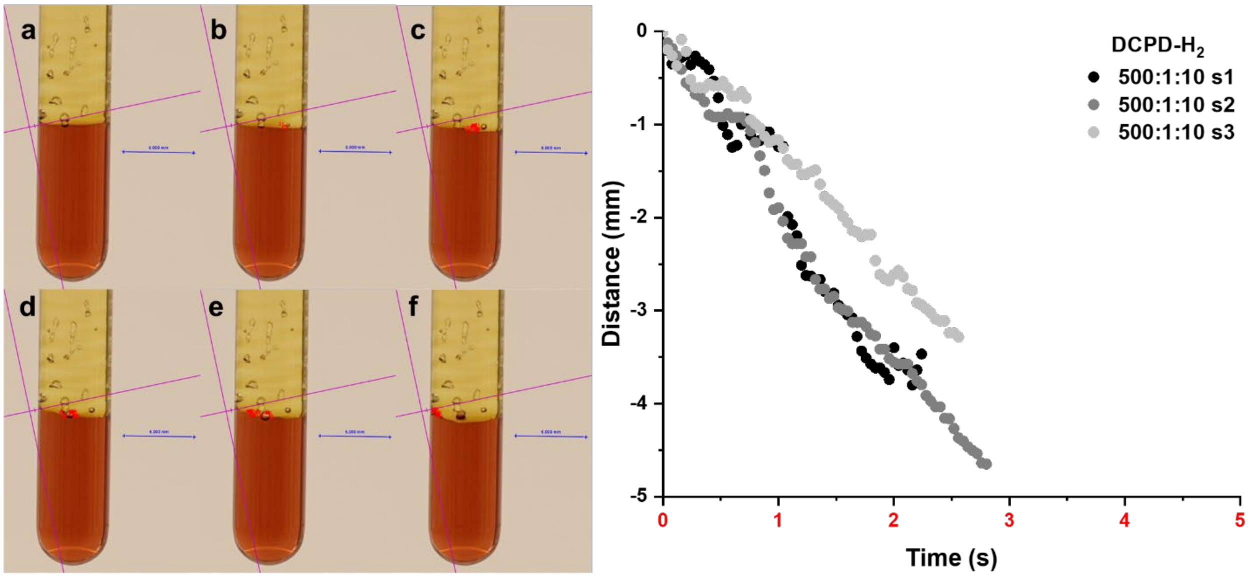

Figure S37: Representative timelapse of front tracking workflow and results for 500:1:10 DCPD-H_2:G2:TBP patterned/across in triplicate ($v_{f,\ pattern}$ = 0.62 ± 0.11 mm/s).

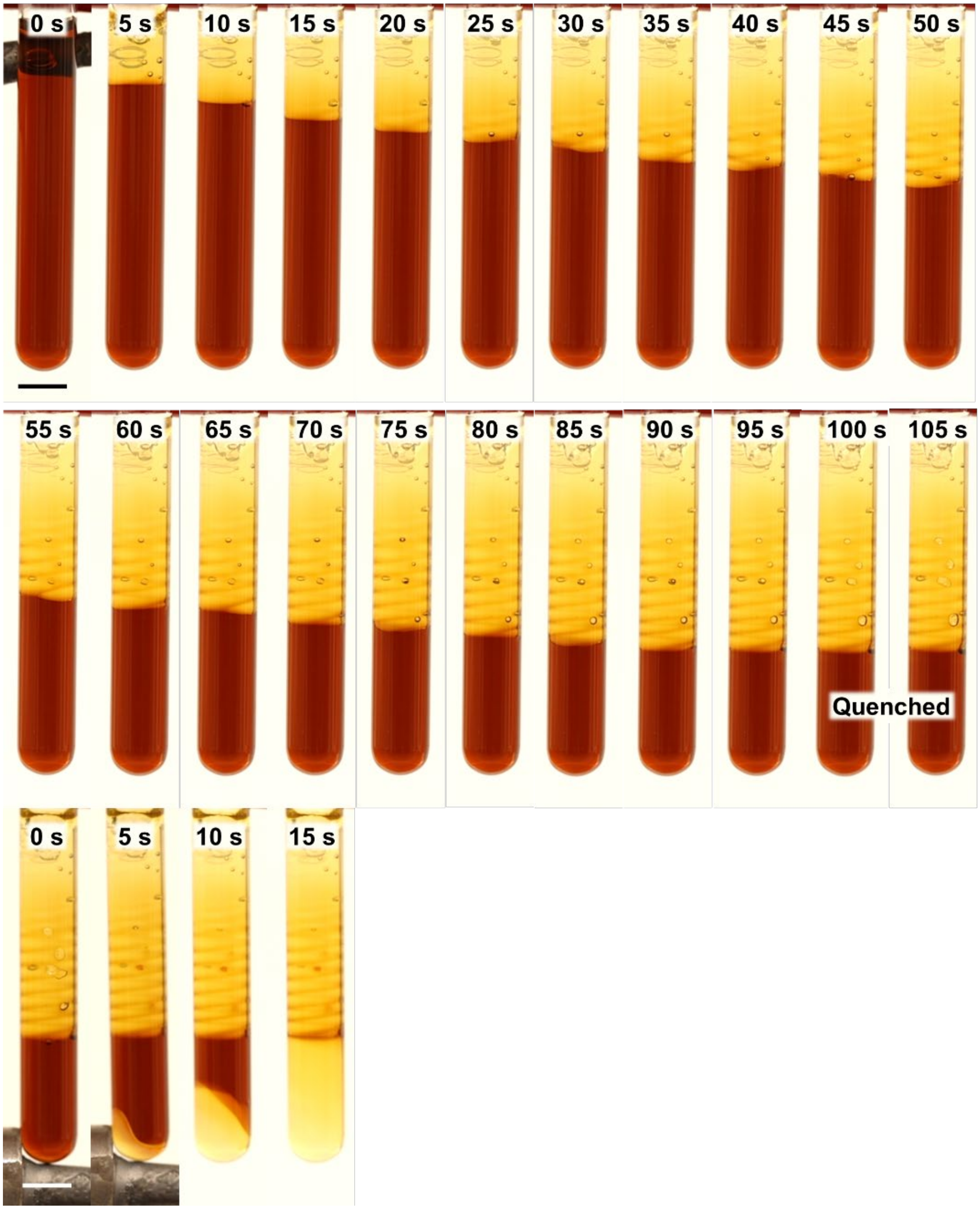

Figure S38: Representative timelapse of 200:1:10 DCPD-H_2:G2:TBP. The scale bar is 5 mm.

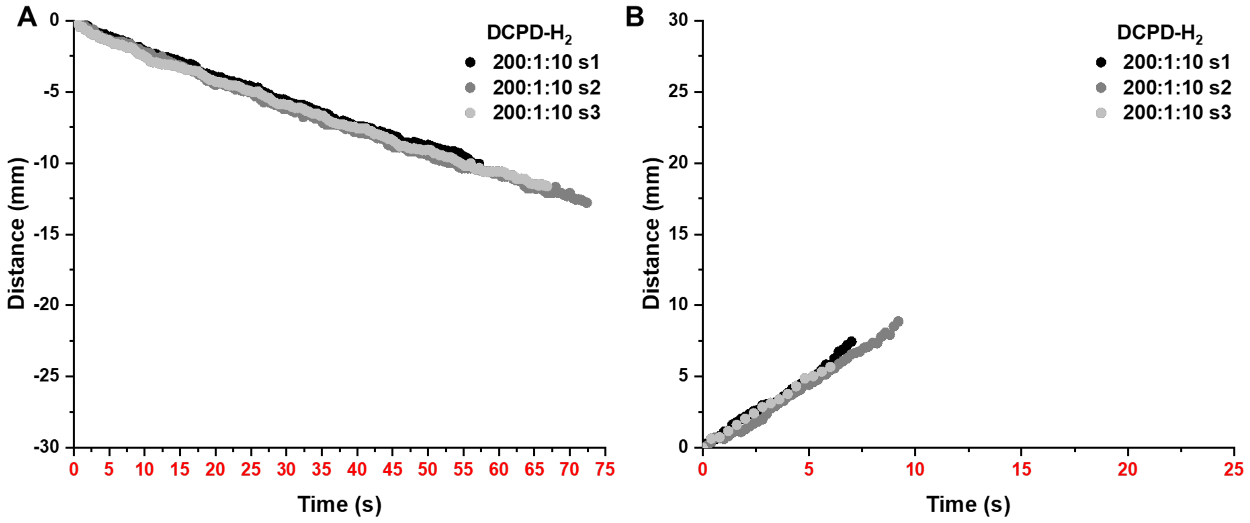

Figure S39: Front tracking of 200:1:10 DCPD-H_2:G2:TBP in triplicate for both downward (A, v_f = 0.17 ± 0.01 mm/s) and upward (B, v_f = 0.97 ± 0.02 mm/s).

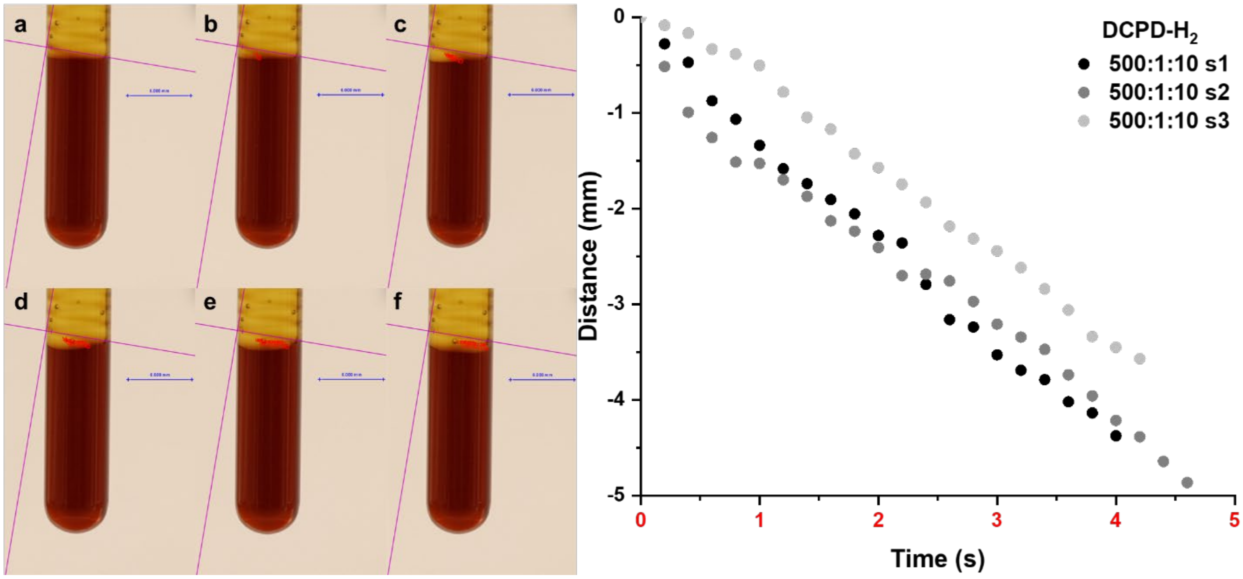

Figure S40: Representative timelapse of front tracking workflow and results for 200:1:10 DCPD-H_2:G2:TBP patterned/across in triplicate ($v_{f,\ pattern}$ = 1.02 ± 0.09mm/s).

Table S1: Front velocity (v_f) for DCPD-H_2 resins at varied loadings.

Monomer (equiv)	Initiator (equiv)	Inhibitor (equiv)	Direction	Front velocity (mm/s)	error
4000	1	1	downward	1.10	0.19
2000	1	1	downward	1.86	0.13
1000	1	1	downward	2.7	0.5
1000	1	1	upward	3.7	0.4
500	1	1	downward	3.2	0.4
4000	1	10	downward	0.39	0.01
4000	1	10	upward	1.20	0.01
2000	1	10	downward	0.43	0.04
1000	1	10	downward	0.49	0.02
1000	1	10	upward	1.00	0.04
1000	1	10	patterned/across	0.35	0.05
500	1	10	downward	0.28	0.01
500	1	10	patterned/across	0.62	0.11
200	1	10	downward	0.17	0.01
200	1	10	upward	0.97	0.02
200	1	10	patterned/across	1.02	0.09

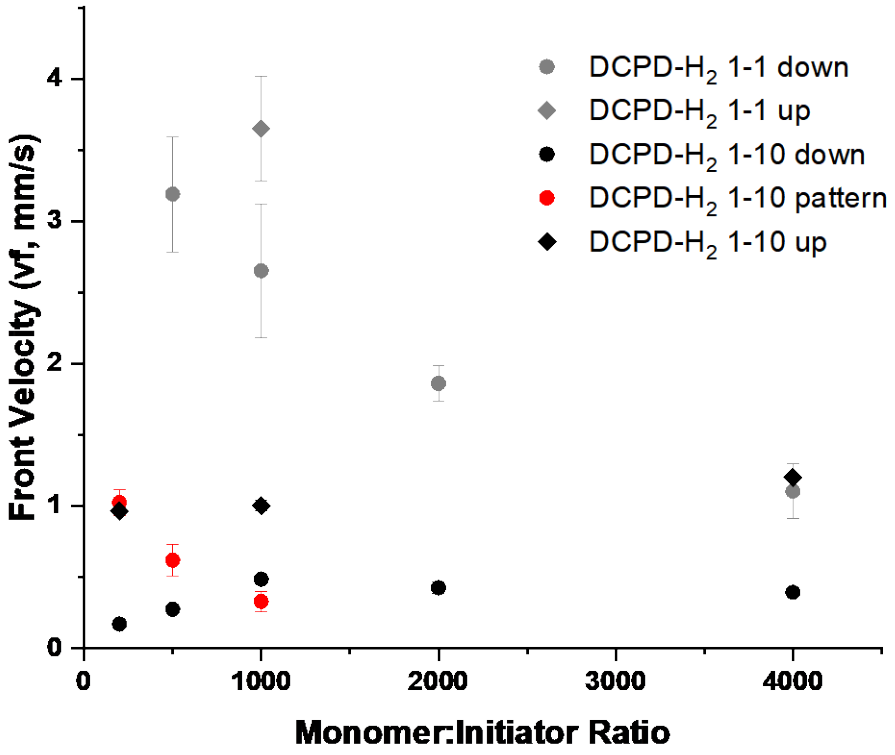

Figure S41: Front velocities for DCPD-H_2 at varied loadings showing similar trend for x:1:1 and x:1:10 (DCPD-H_2:G2:TBP) when the patterns are tracked across the tube (red) but not simply globally down the tube. Additionally, shows the upward front velocity is faster for both samples likely due to convection mixing: the heating the liquid at the bottom makes it less dense and float to the top during initiation/during front propagation and increasing the initial temperature of the entire solution.

DSC: (Cure kinetics):

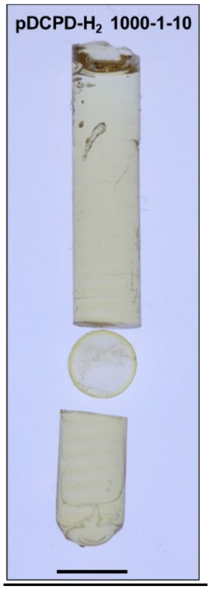

Figure S42: Representative sample cut for DSC, NMR, and SEC. Cut disk was cut again in the opposite direction (in the direction of the global front) into three samples for each test.

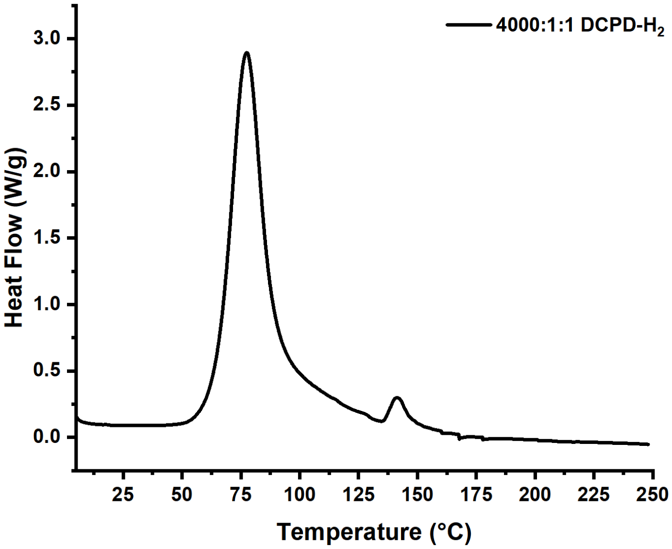

Figure S43: Representative DSC cure kinetic profile for 4000:1:1 DCPD-H_2:G2:TBP (H_r = 385 ± 7 J/g, Peak Temp = 77 ± 4 °C). Exo up

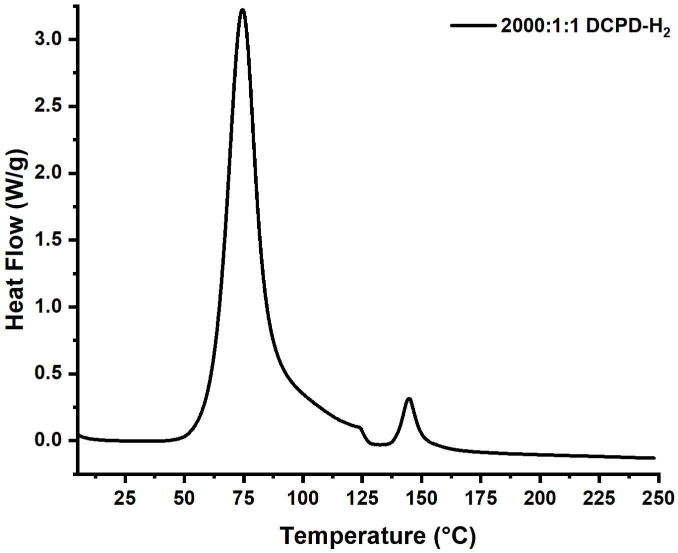

Figure S44: Representative DSC cure kinetic profile for 2000:1:1 DCPD-H_2:G2:TBP (H_r = 394 ± 15 J/g, Peak Temp = 75 ± 1 °C). Exo up

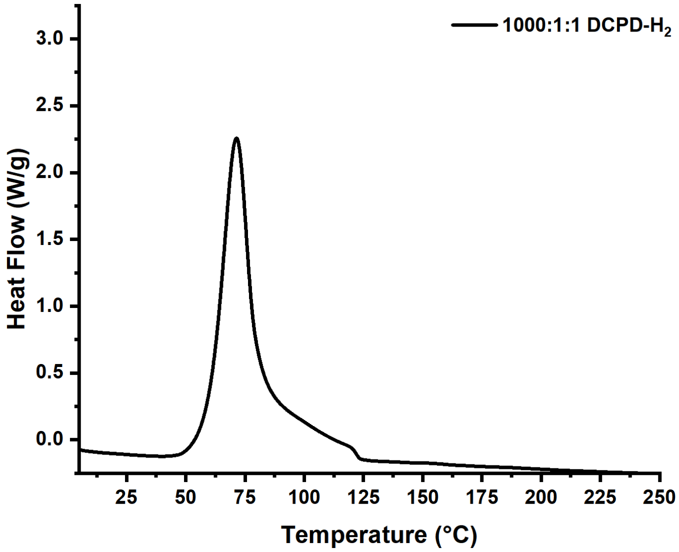

Figure S45: Representative DSC cure kinetic profile for 1000:1:1 DCPD-H_2:G2:TBP (H_r = 372 ± 9 J/g, Peak Temp = 73 ± 1 °C). Exo up

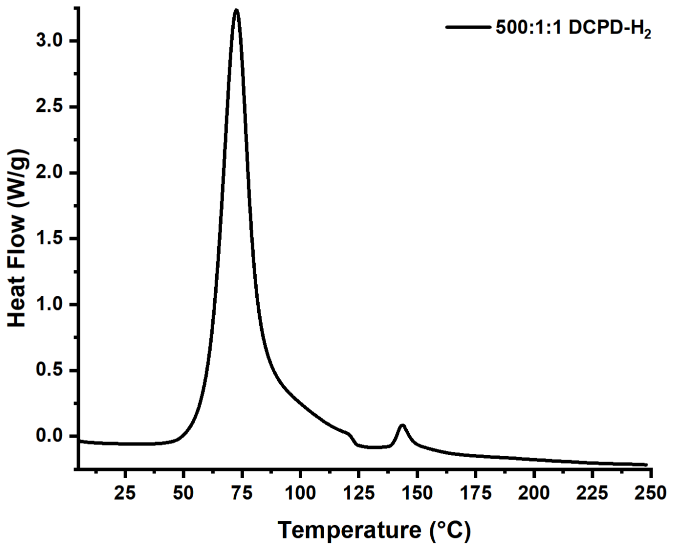

Figure S46: Representative DSC cure kinetic profile for 500:1:1 DCPD-H_2:G2:TBP (H_r = 372 ± 24 J/g, Peak Temp = 72 ± 1 °C). Exo up

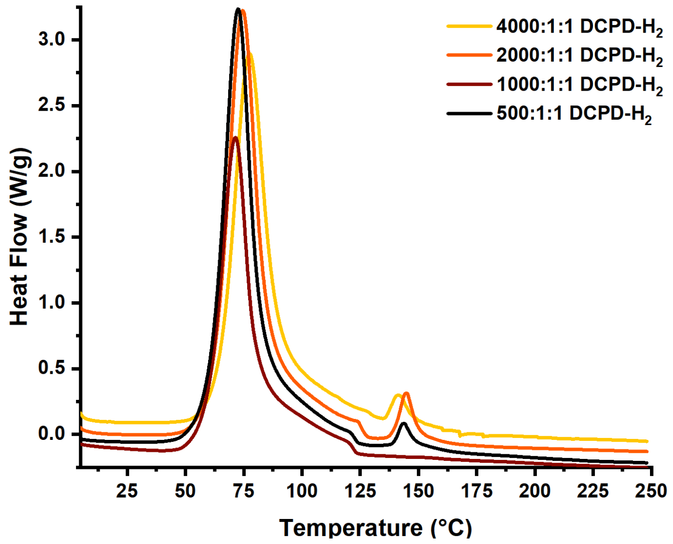

Figure S47: Stacked representative DSC cure kinetic profile for x:1:1 DCPD-H_2:G2:TBP. Exo up

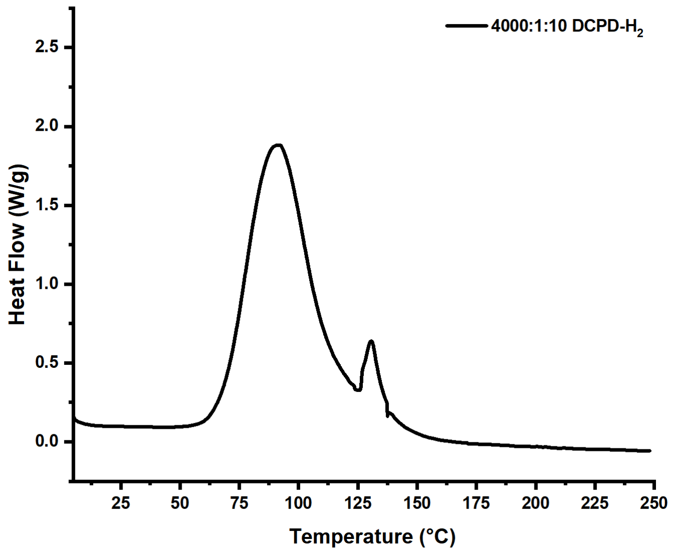

Figure S48: Representative DSC cure kinetic profile for 4000:1:10 DCPD-H_2:G2:TBP (H_r = 390 ± 8 J/g, Peak Temp = 92 ± 1 °C). Exo up

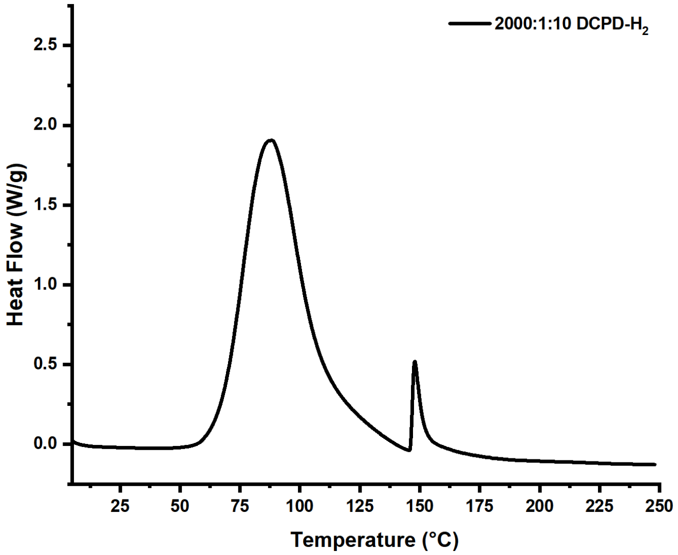

Figure S49: Representative DSC cure kinetic profile for 2000:1:10 DCPD-H_2:G2:TBP (H_r = 408 ± 10 J/g, Peak Temp = 89 ± 1 °C). Exo up

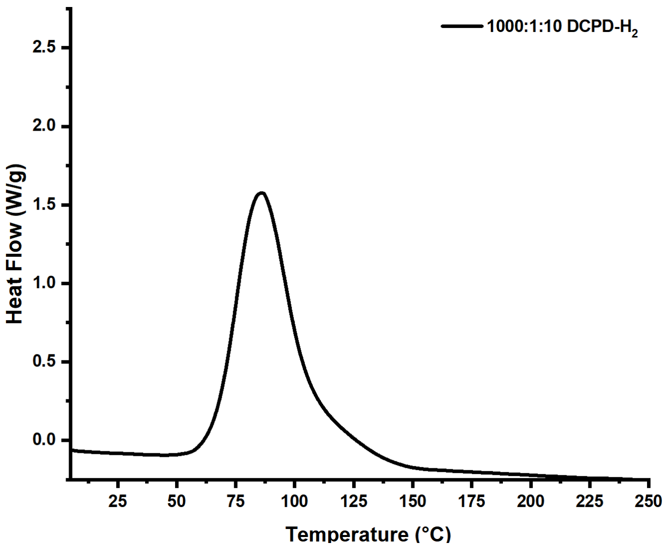

Figure S50: Representative DSC cure kinetic profile for 1000:1:10 DCPD-H_2:G2:TBP (H_r = 368 ± 34 J/g, Peak Temp = 86 ± 1 °C). Exo up

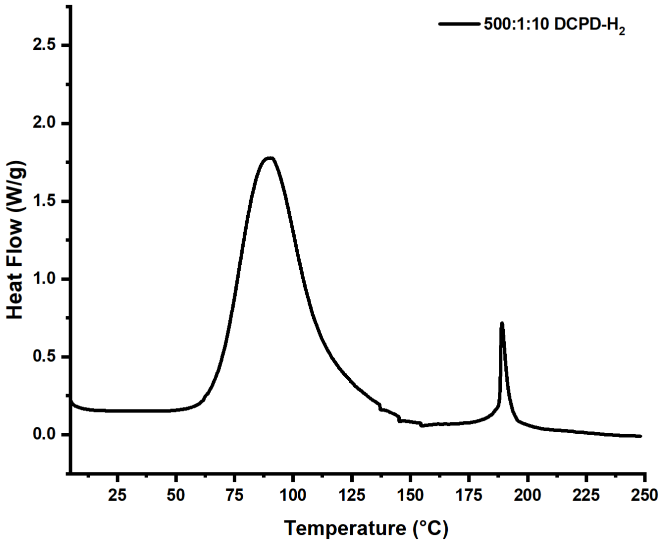

Figure S51: Representative DSC cure kinetic profile for 500:1:10 DCPD-H_2:G2:TBP (H_r = 346 ± 17 J/g, Peak Temp = 90 ± 1 °C). Exo up

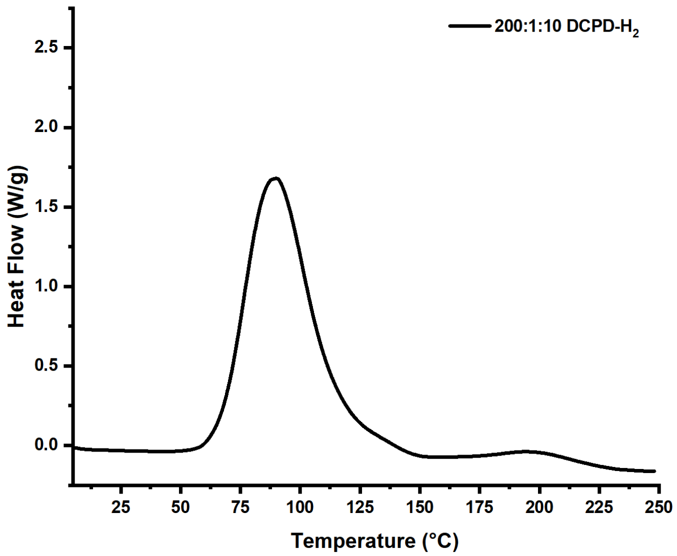

Figure S52: Representative DSC cure kinetic profile for 200:1:10 DCPD-H_2:G2:TBP (H_r = 337 ± 1 J/g, Peak Temp = 90 ± 1 °C). Exo up

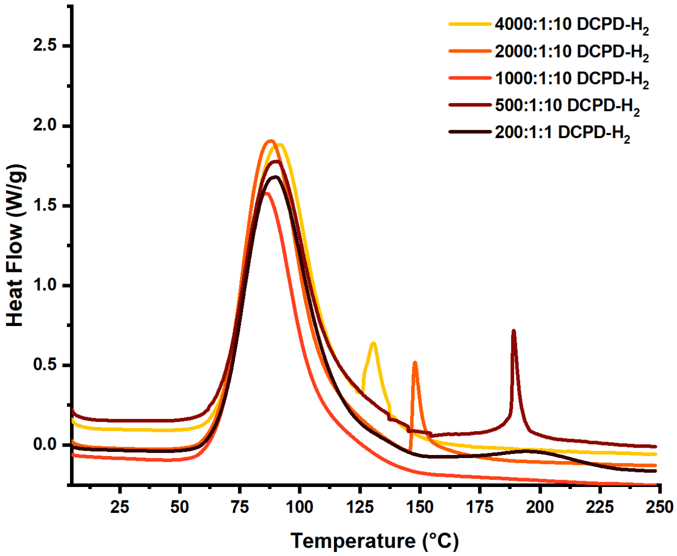

Figure S53: Stacked representative DSC cure kinetic profile for x:1:10 DCPD-H_2:G2:TBP. Exo up

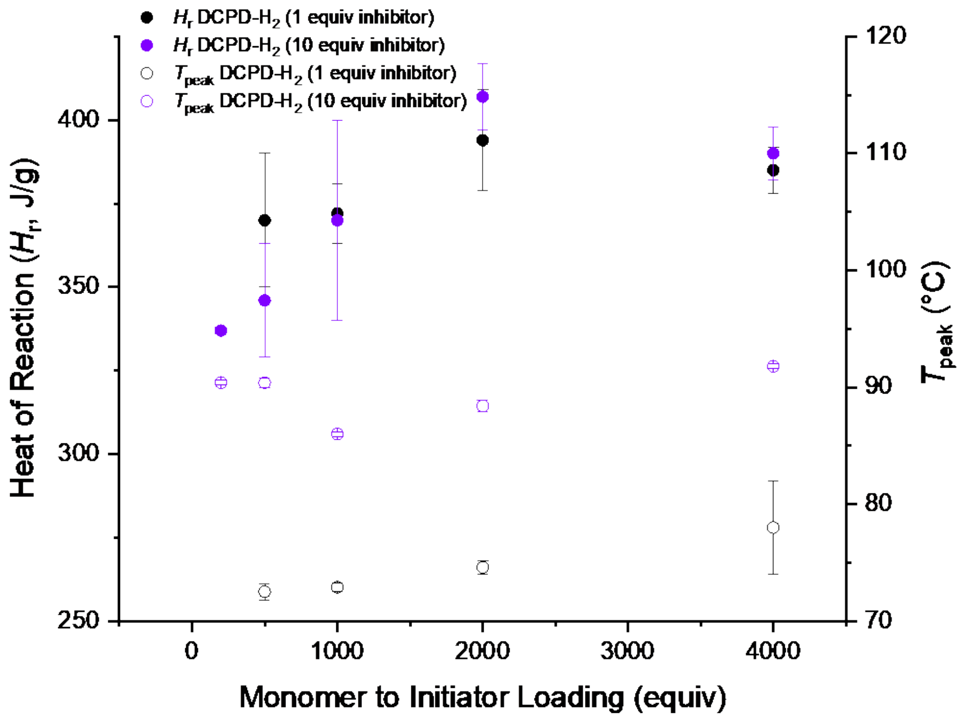

Figure S54: Heat of reaction (H_r) and temperature of peak maximum (T_{peak}) showing retention of H_r and increasing T_{peak} with increasing inhibitor loading.

Table S2: Heat of reaction (H_r) and peak temperature (T_{peak}) for DCPD-H_2 resins at varied loadings.

Monomer (equiv)	Initiator (equiv)	Inhibitor (equiv)	H_r (J/g)	error	T_{peak} (°C)	error
4000	1	1	385	7	76.6	4.4
2000	1	1	394	15	74.6	0.6
1000	1	1	372	9	72.9	0.4
500	1	1	372	24	71.7	0.7
4000	1	10	390	8	91.5	0.2
2000	1	10	408	10	88.7	0.5
1000	1	10	368	34	86.2	0.2
500	1	10	346	17	90.0	0.4
200	1	10	337	1	90.2	0.2

Sample Images:

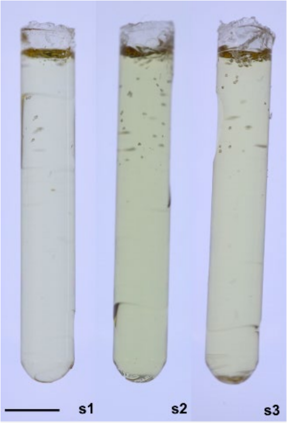

Figure S55: Image of triplicate pDCPD-H_2 samples at 4000:1:1 post-FROMP. The scale bar is 5 mm.

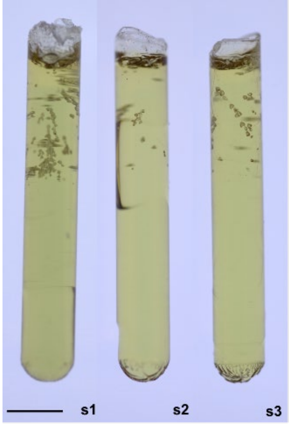

Figure S56: Image of triplicate pDCPD-H_2 samples at 2000:1:1 post-FROMP. The scale bar is 5 mm.

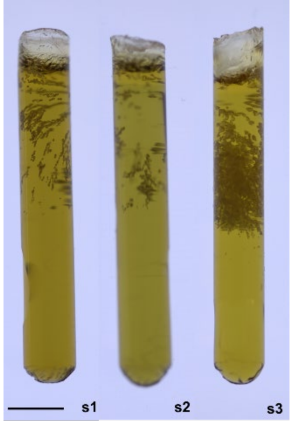

Figure S57: Image of triplicate pDCPD-H_2 samples at 1000:1:1 post-FROMP. The scale bar is 5 mm.

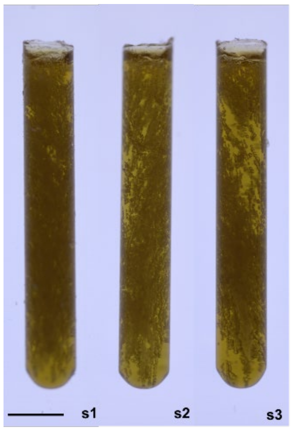

Figure S58: Image of triplicate pDCPD-H_2 samples at 1000:1:1 post-FROMP **bottom up**. The scale bar is 5 mm.

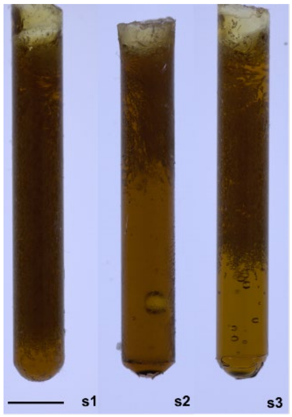

Figure S59: Image of triplicate pDCPD-H_2 samples at 500:1:1 post-FROMP. The scale bar is 5 mm.

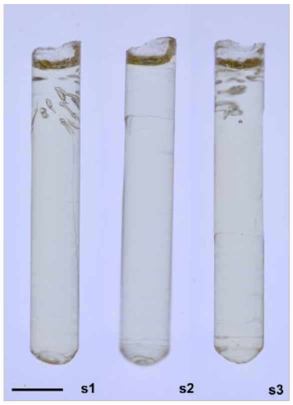

Figure S60: Image of triplicate pDCPD-H_2 samples at 4000:1:10 post-FROMP. The scale bar is 5 mm.

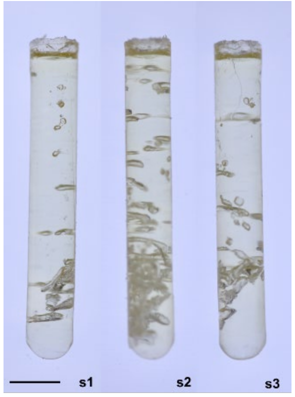

Figure S61: Image of triplicate pDCPD-H_2 samples at 4000:1:10 post-FROMP **bottom up**. The scale bar is 5 mm.

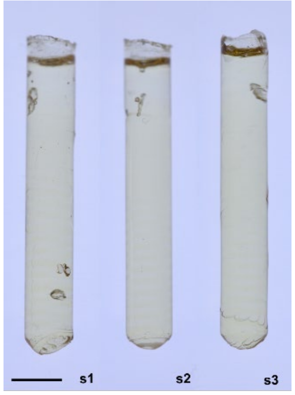

Figure S62: Image of triplicate pDCPD-H_2 samples at 2000:1:10 post-FROMP. The scale bar is 5 mm.

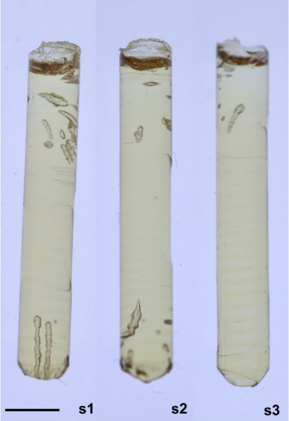

Figure S63: Image of triplicate pDCPD-H_2 samples at 1000:1:10 post-FROMP. The scale bar is 5 mm.

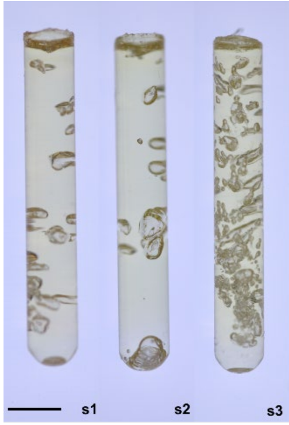

Figure S64: Image of triplicate pDCPD-H_2 samples at 1000:1:10 post-FROMP **bottom up**. The scale bar is 5 mm.

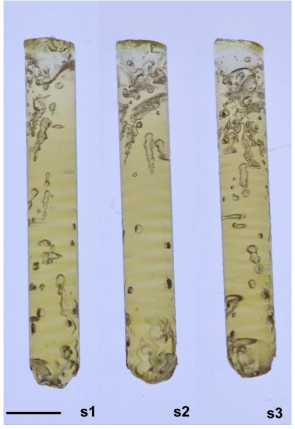

Figure S65: Image of triplicate pDCPD-H_2 samples at 500:1:10 post-FROMP. The scale bar is 5 mm.

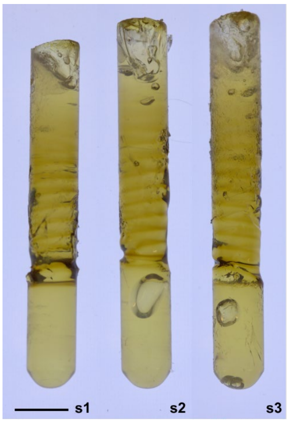

Figure S66: Image of triplicate pDCPD-H_2 samples at 200:1:10 post-FROMP. The scale bar is 5 mm.

Size Exclusion Chromatography (SEC):

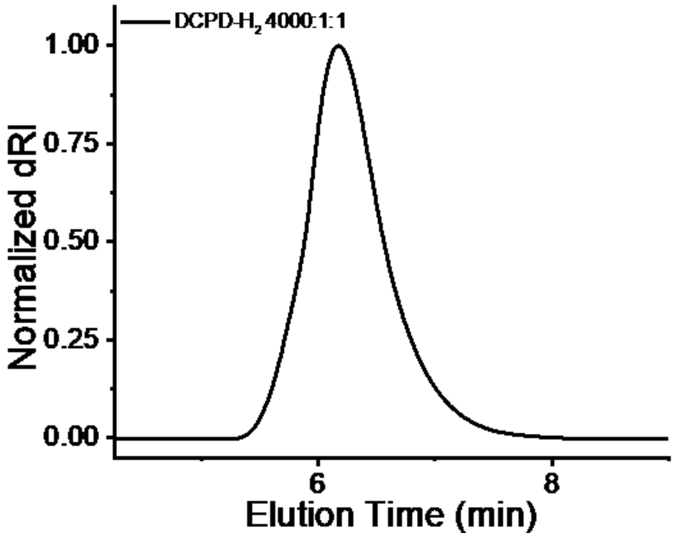

Figure S67: Representative SEC of pDCPD-H_2 post-FROMP for 4000:1:1 (M_n = 380 ± 90 kg/mol, $Đ$ = 1.57 ± 0.07).

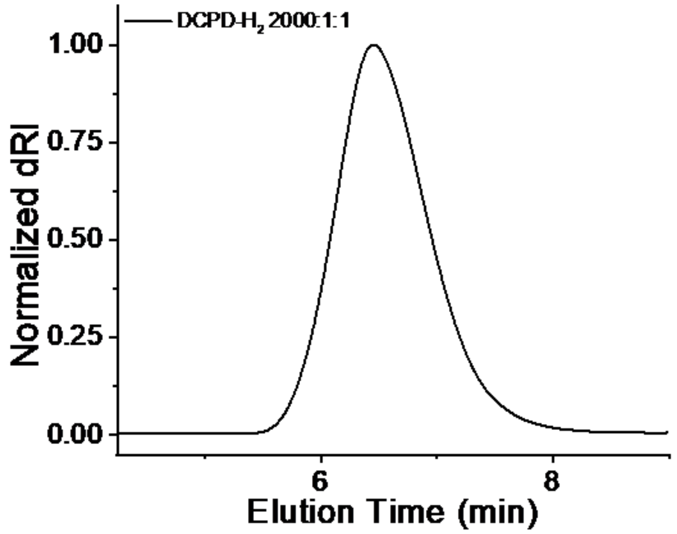

Figure S68: Representative SEC of pDCPD-H_2 post-FROMP for 2000:1:1 (M_n = 220 ± 10 kg/mol, $Đ$ = 1.67 ± 0.05).

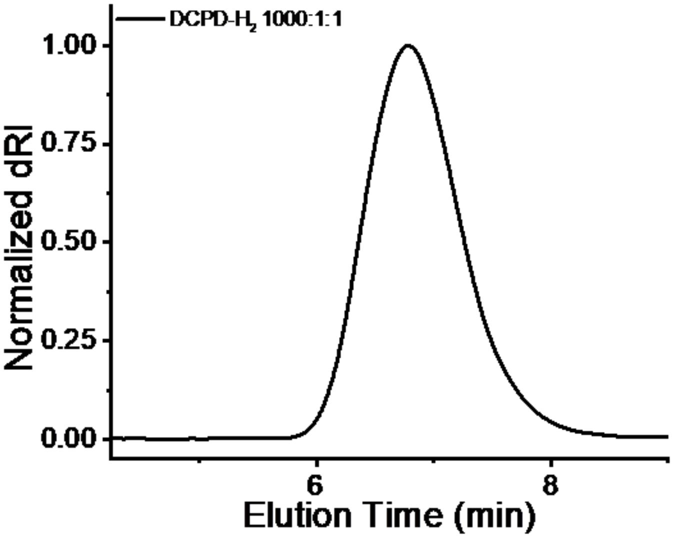

Figure S69: Representative SEC of pDCPD-H_2 post-FROMP for 1000:1:1 (M_n = 130 ± 20 kg/mol, $Đ$ = 1.68 ± 0.11).

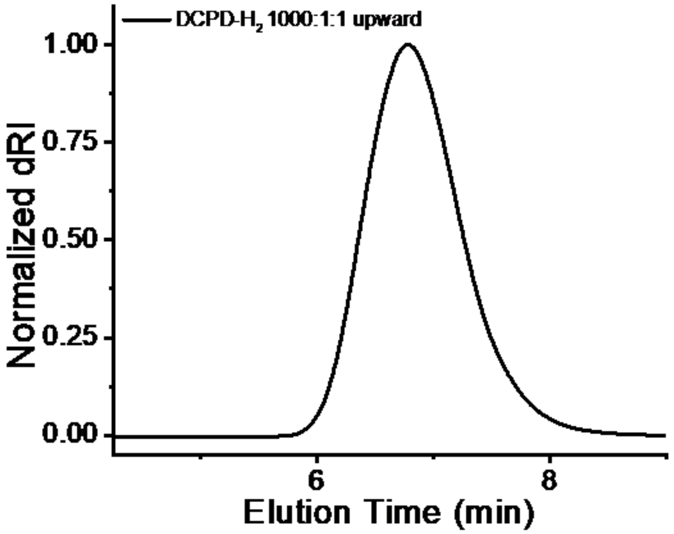

Figure S70: Representative SEC of pDCPD-H_2 post-FROMP for 1000:1:1 **bottom up** (M_n = 130 ± 10 kg/mol, $Đ$ = 1.67 ± 0.07).

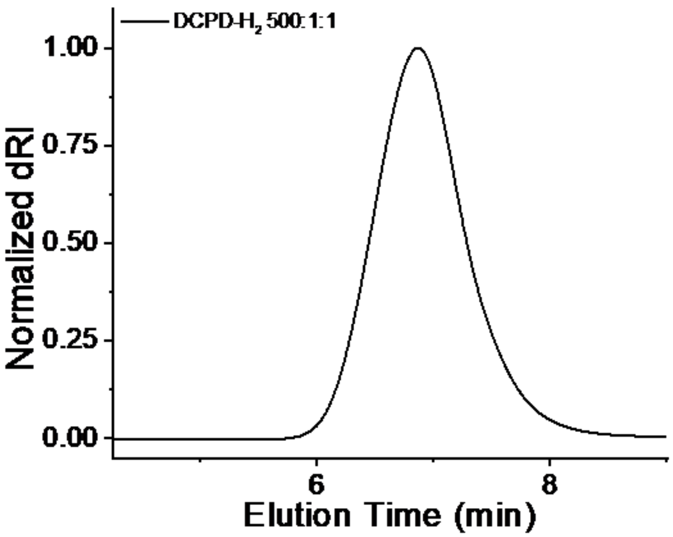

Figure S71: Representative SEC of pDCPD-H_2 post-FROMP for 500:1:1 (M_n = 108 ± 1 kg/mol, $Đ$ = 1.51 ± 0.02).

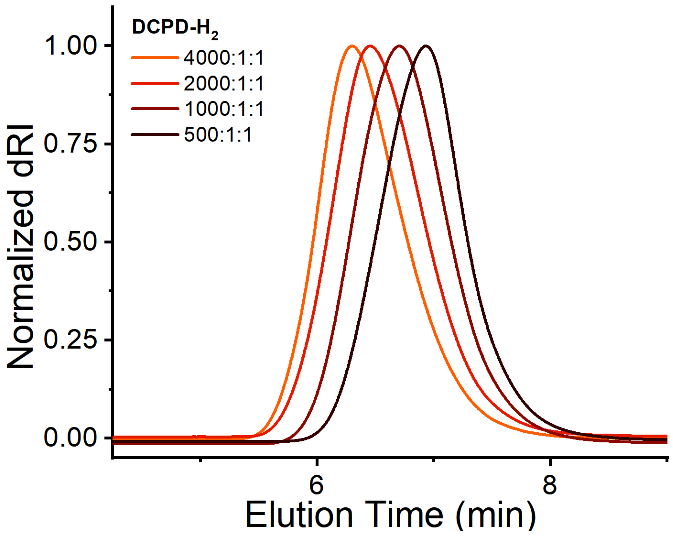

Figure S72: Representative SEC overlay of DCPD-H_2 post-FROMP for varied loadings (x:1:1 monomer:initiator:inhibitor).

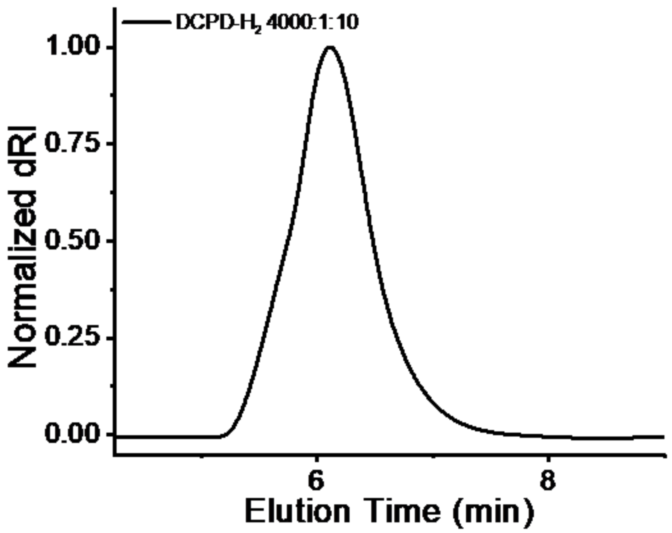

Figure S73: Representative SEC of pDCPD-H_2 post-FROMP for 4000:1:10 (M_n = 640 ± 70 kg/mol, $Đ$ = 1.55 ± 0.01).

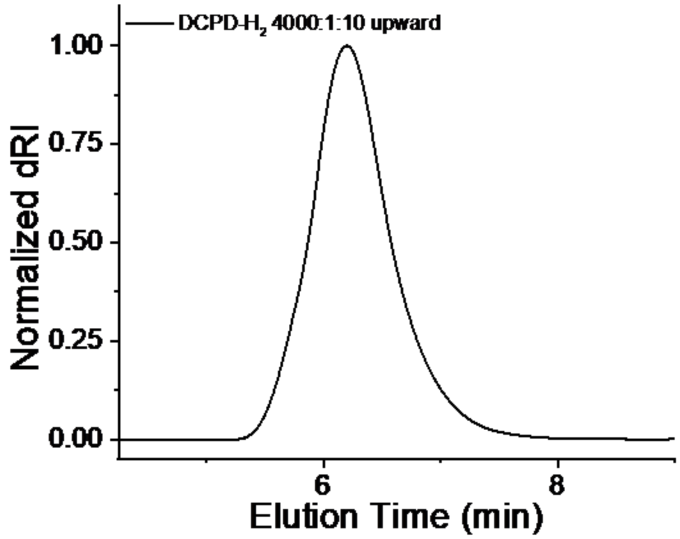

Figure S74: Representative SEC of pDCPD-H_2 post-FROMP for 4000:1:10 **bottom up** (M_n = 480 ± 90 kg/mol, $Đ$ = 1.45 ± 0.02).

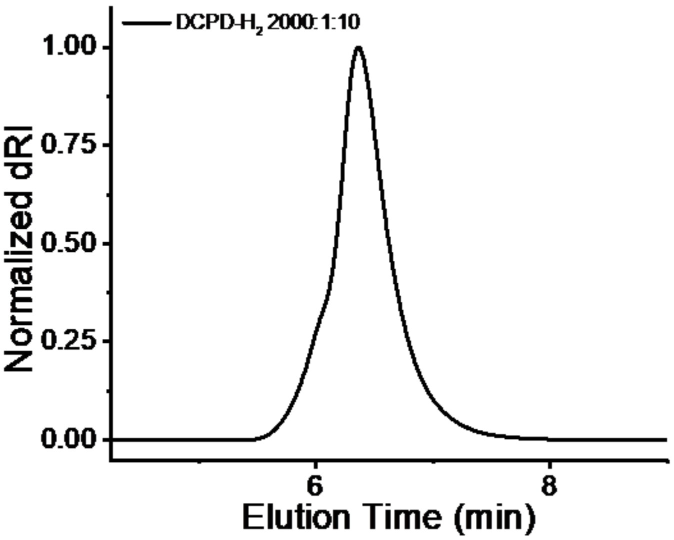

Figure S75: Representative SEC of pDCPD-H_2 post-FROMP for 2000:1:10 (M_n = 360 ± 10 kg/mol, $Đ$ = 1.31 ± 0.01).

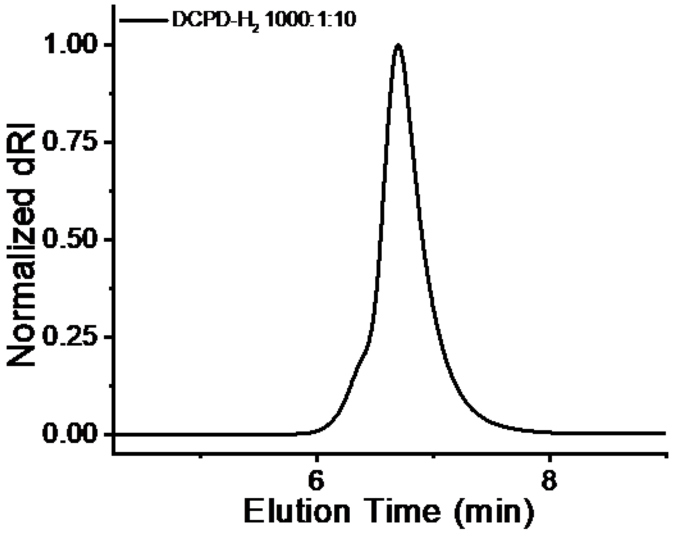

Figure S76: Representative SEC of pDCPD-H_2 post-FROMP for 1000:1:10 (M_n = 188 ± 5 kg/mol, $Đ$ = 1.21 ± 0.02).

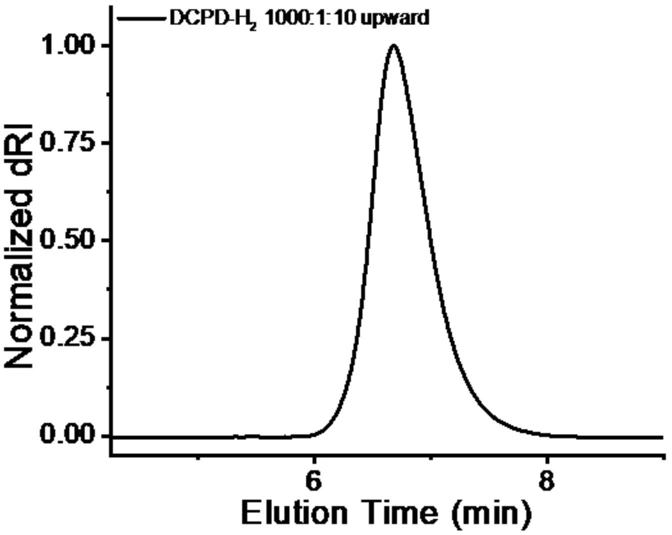

Figure S77: Representative SEC of pDCPD-H_2 post-FROMP for 1000:1:10 **bottom up** (M_n = 164 ± 7 kg/mol, $Đ$ = 1.30 ± 0.06).

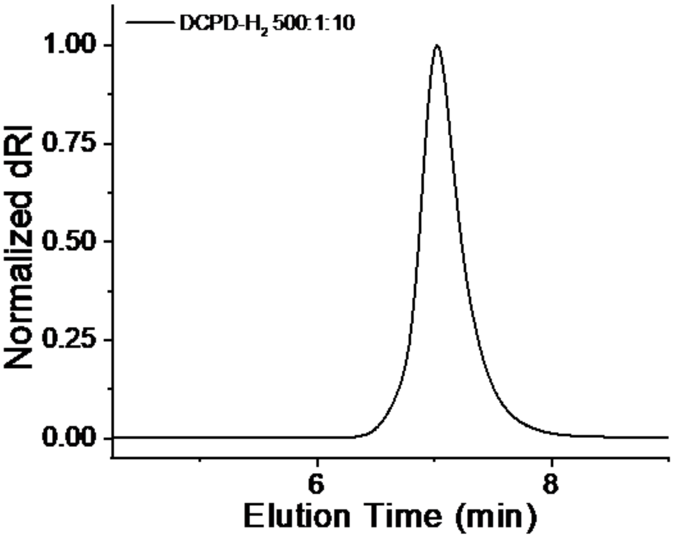

Figure S78: Representative SEC of pDCPD-H_2 post-FROMP for 500:1:10 (M_n = 101 ± 1 kg/mol, $Đ$ =1.13 ± 0.01).

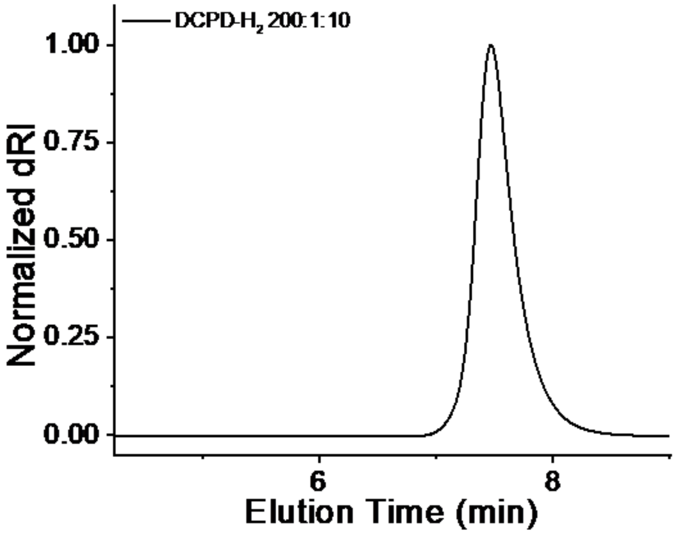

Figure S79: Representative SEC of pDCPD-H_2 post-FROMP for 200:1:10 (M_n = 41 ± 3 kg/mol, $Đ$ = 1.09 ± 0.01).

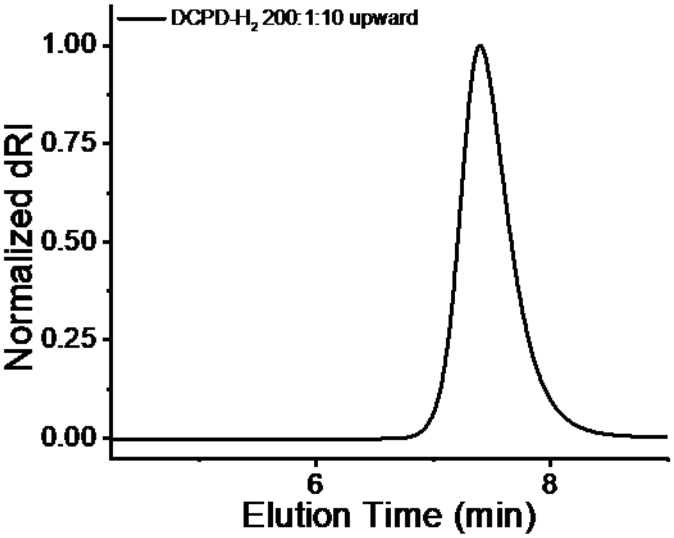

Figure S80: Representative SEC of pDCPD-H_2 post-FROMP for 200:1:10 **bottom up** (M_n = 39 ± 2 kg/mol, $Đ$ = 1.16 ± 0.01).

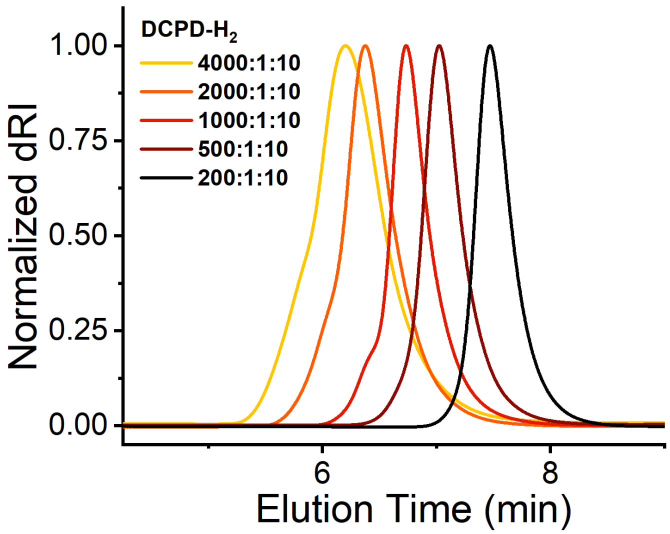

Figure S81: Representative SEC overlay of DCPD-H₂ post-FROMP for varied loadings (x:1:10 monomer:initiator:inhibitor).

Table S3: SEC molecular weights and dispersity (n = 3) for pDCPD-H₂ post-FROMP at varying loadings.

Monomer (equiv)	Initiator (equiv)	Inhibitor (equiv)	Direction	DP	error	*Đ*	error
4000	1	1	downward	2800	700	1.57	0.07
2000	1	1	downward	1630	80	1.67	0.05
1000	1	1	downward	1000	100	1.68	0.11
1000	1	1	upward	990	80	1.67	0.07
500	1	1	downward	800	10	1.51	0.02
4000	1	10	downward	4800	500	1.55	0.01
4000	1	10	upward	3600	600	1.45	0.02
2000	1	10	downward	2650	80	1.31	0.01
1000	1	10	downward	1400	40	1.21	0.02
1000	1	10	upward	1220	50	1.30	0.06
500	1	10	downward	750	10	1.13	0.00
200	1	10	downward	300	20	1.09	0.01
200	1	10	upward	290	10	1.16	0.01

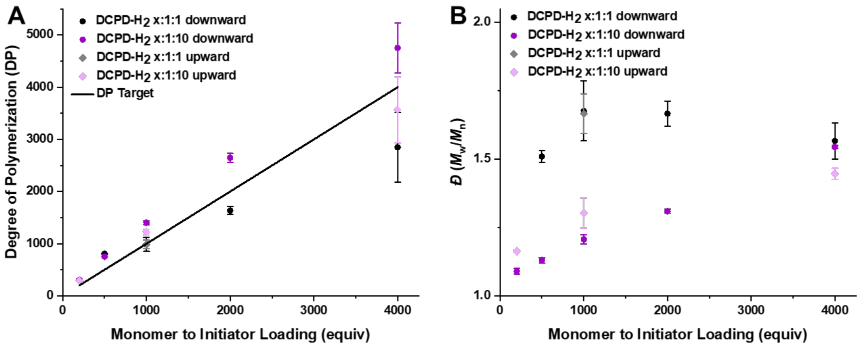

Figure S82: (A) DP and (B) *Đ* for pDCPD.

NMR:

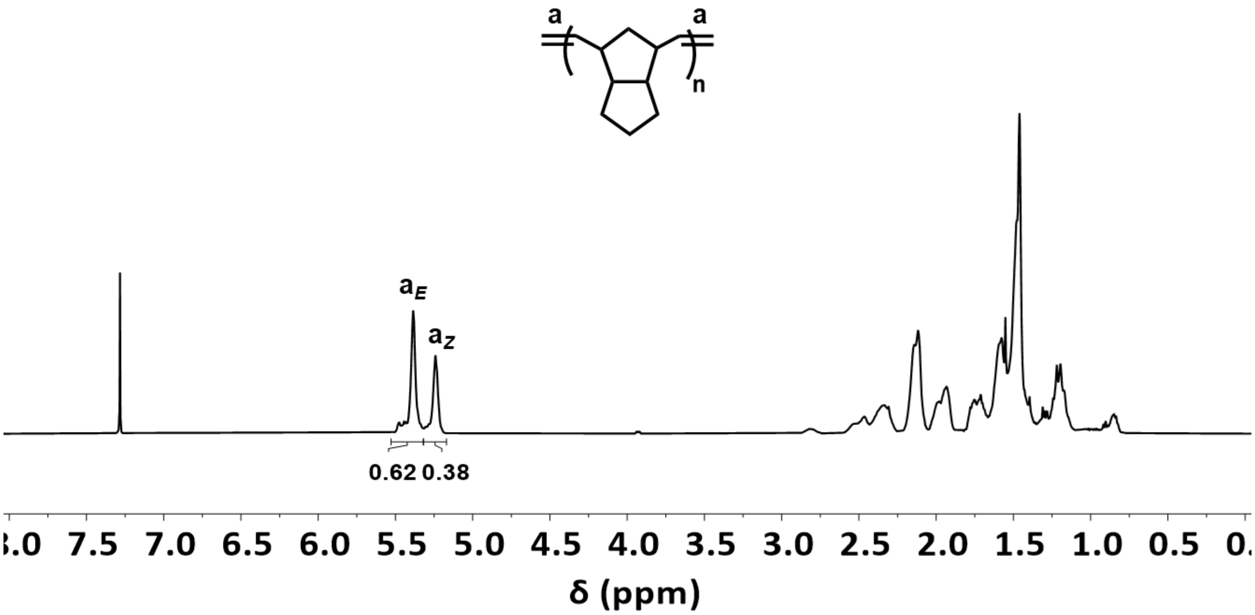

Figure S83: Representative NMR of pDCPD-H_2 post-FROMP for 4000:1:1 with *E* and *Z* configurational signals (a_E and a_Z, respectively) and ratio.

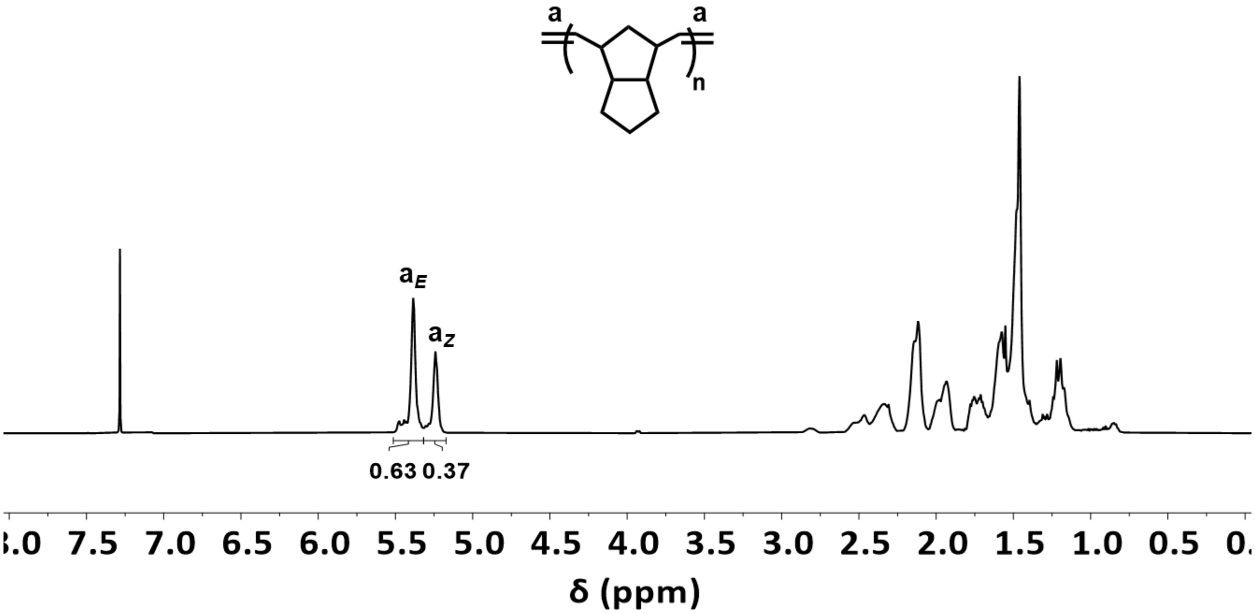

Figure S84: Representative NMR of pDCPD-H_2 post-FROMP for 2000:1:1 with *E* and *Z* configurational signals (a_E and a_Z, respectively) and ratio.

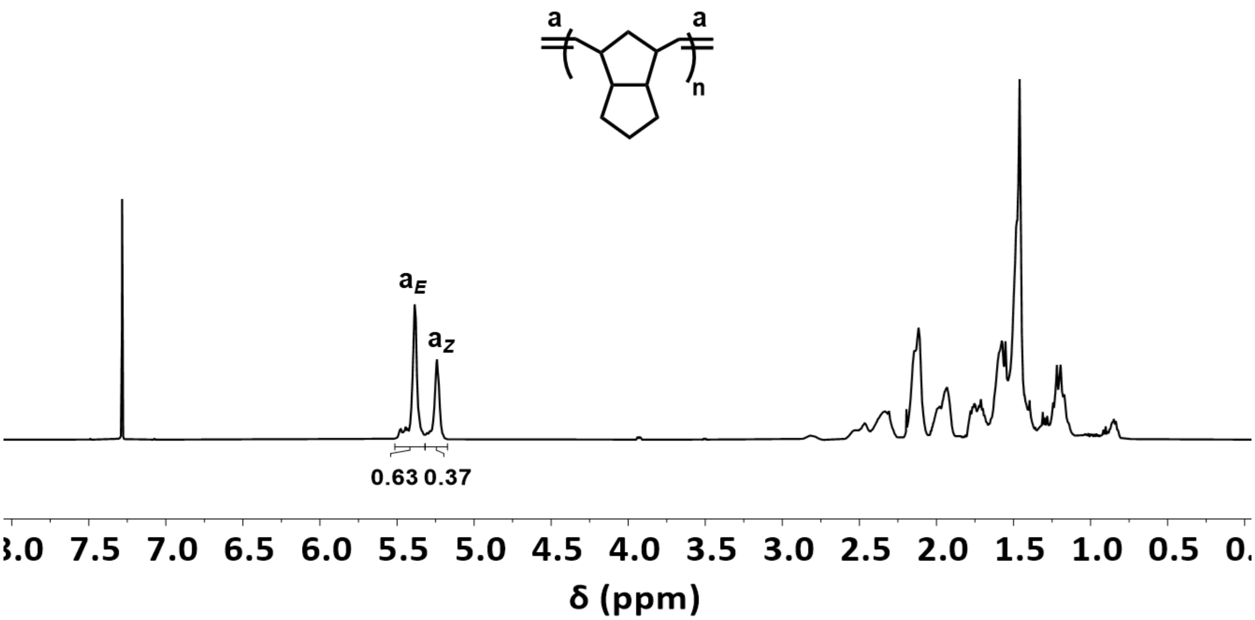

Figure S85: Representative NMR of pDCPD-H_2 post-FROMP for 1000:1:1 with *E* and *Z* configurational signals (a_E and a_Z, respectively) and ratio.

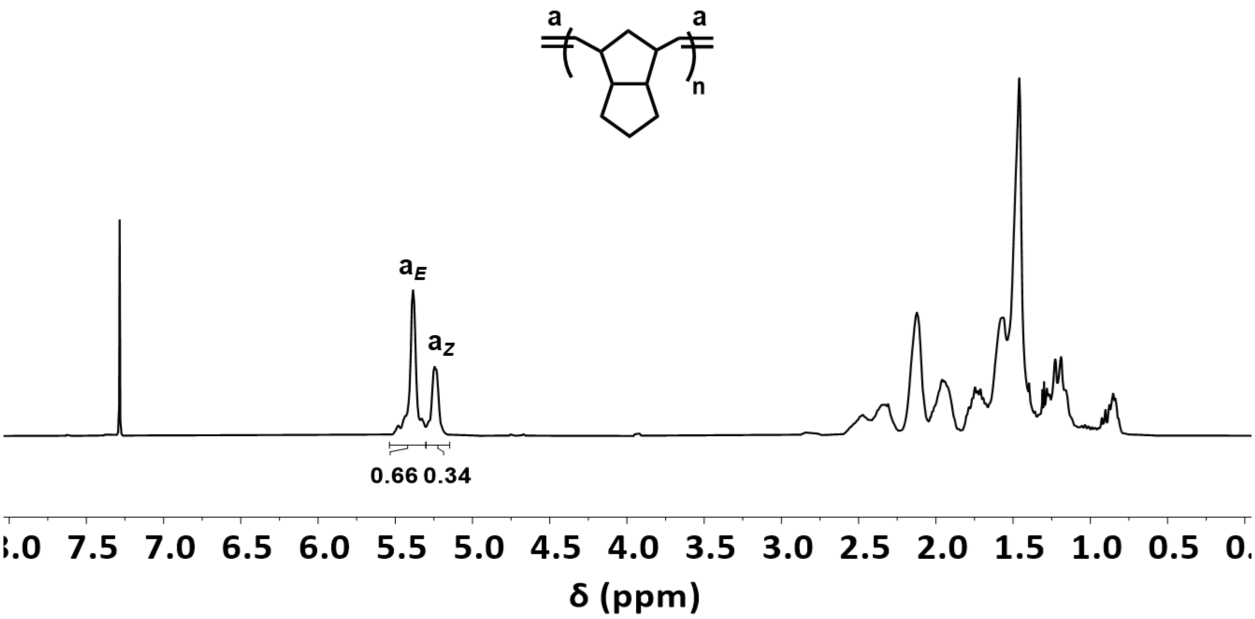

Figure S86: Representative NMR of pDCPD-H_2 post-FROMP for 1000:1:1 **bottom up** with *E* and *Z* configurational signals (a_E and a_Z, respectively) and ratio.

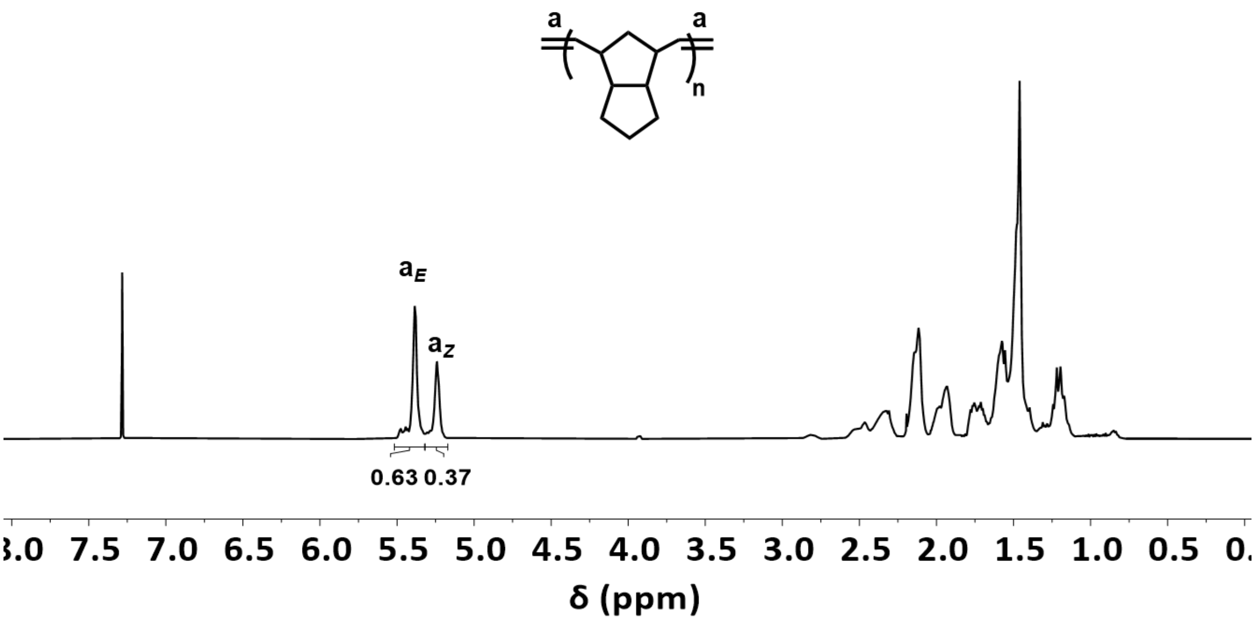

Figure S87: Representative NMR of pDCPD-H_2 post-FROMP for 500:1:1 with *E* and *Z* configurational signals (a_E and a_Z, respectively) and ratio.

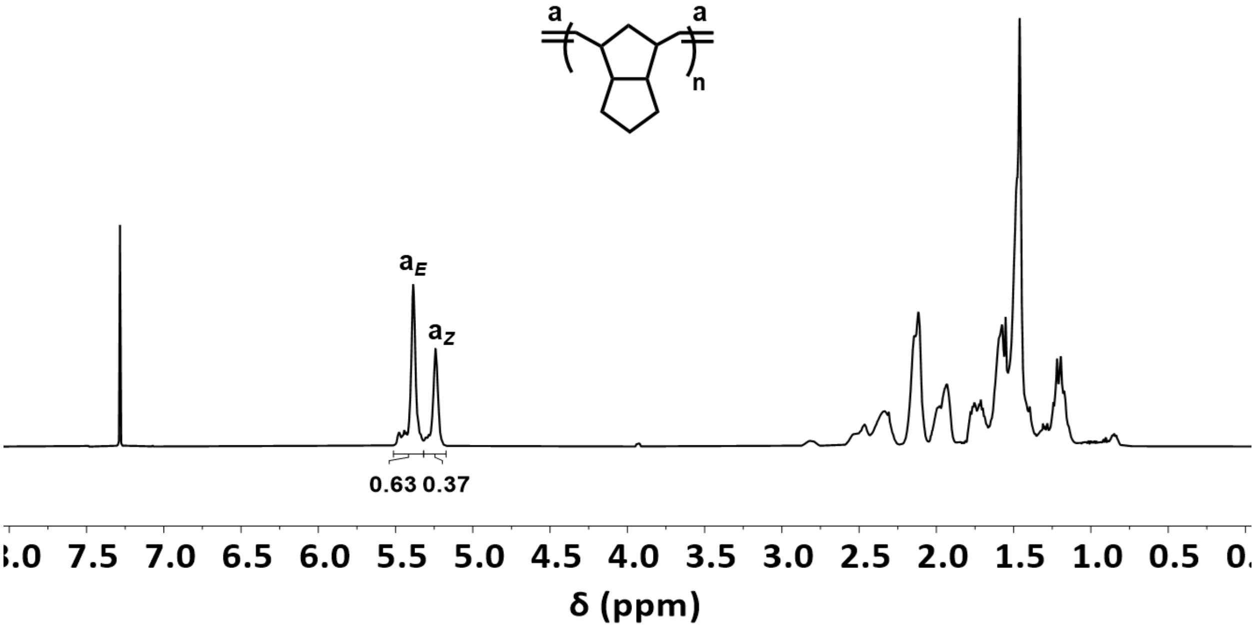

Figure S88: Representative NMR of pDCPD-H_2 post-FROMP for 200:1:1 with *E* and *Z* configurational signals (a_E and a_Z, respectively) and ratio.

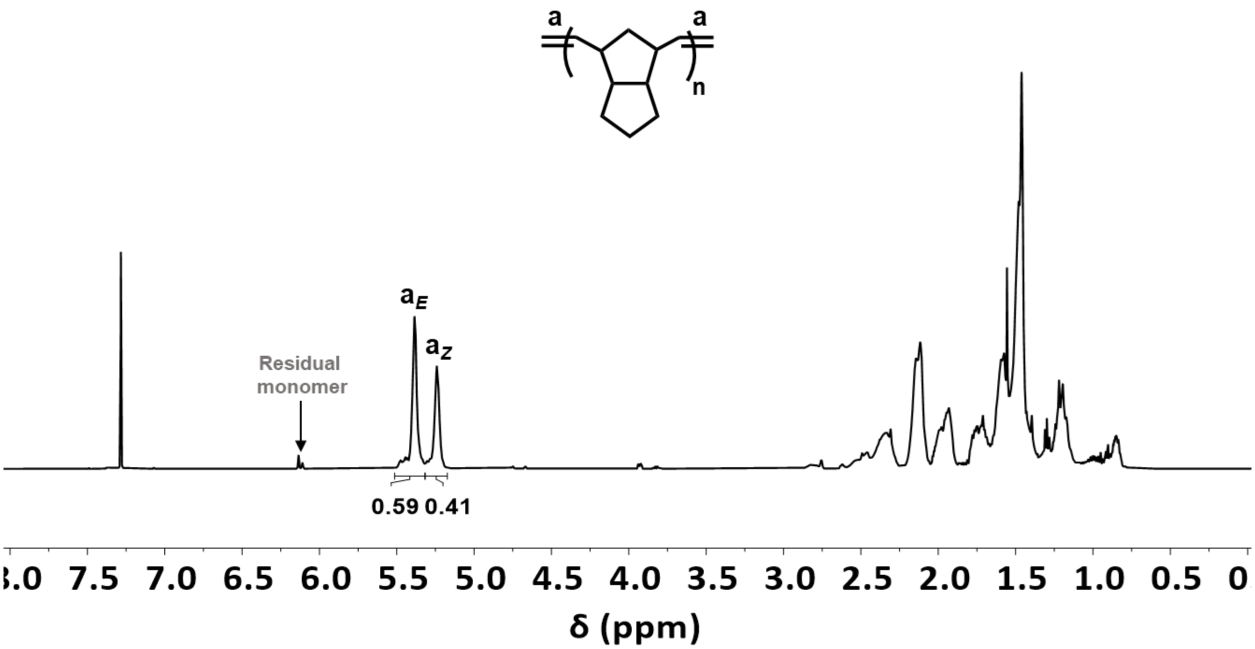

Figure S89: Representative NMR of pDCPD-H_2 post-FROMP for 4000:1:10 with *E* and *Z* configurational signals (a_E and a_Z, respectively) and ratio.

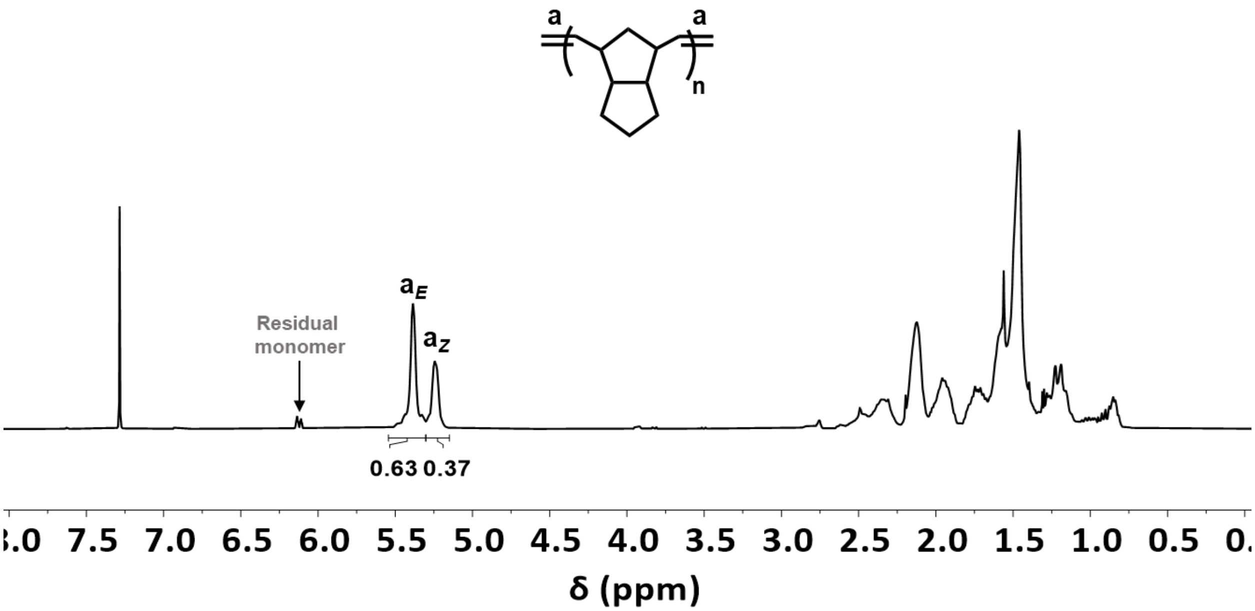

Figure S90: Representative NMR of pDCPD-H_2 post-FROMP for 4000:1:10 **bottom up** with *E* and *Z* configurational signals (a_E and a_Z, respectively) and ratio.

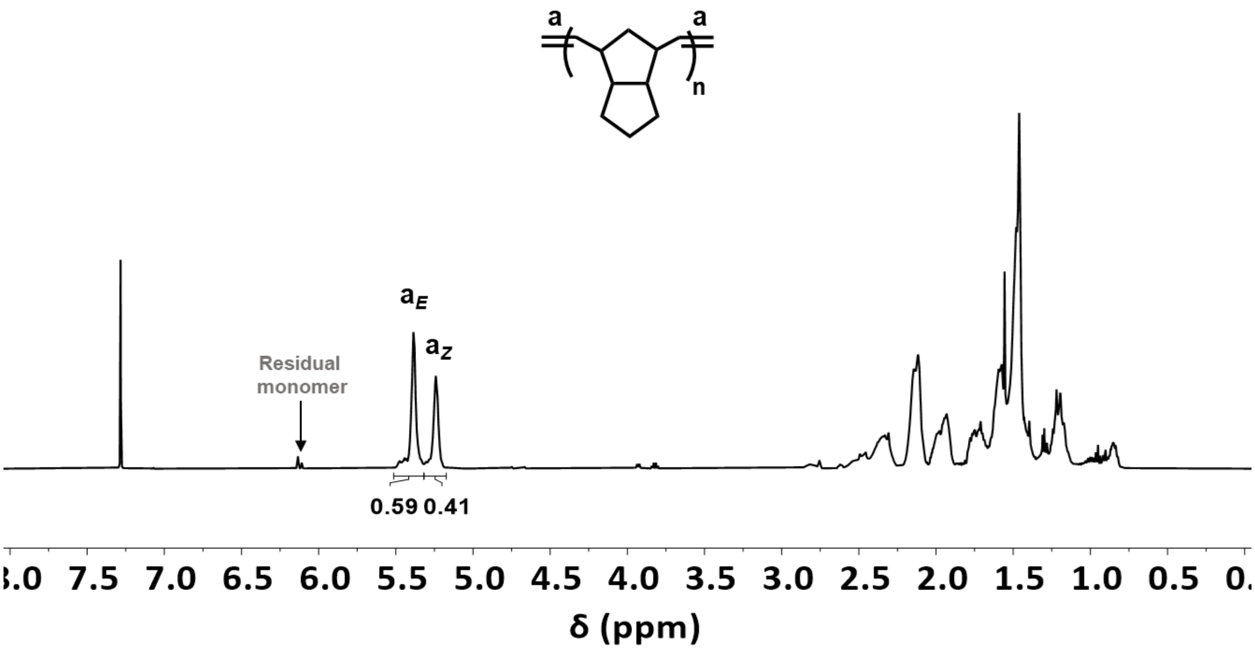

Figure S91: Representative NMR of pDCPD-H_2 post-FROMP for 2000:1:10 with *E* and *Z* configurational signals (a_E and a_Z, respectively) and ratio.

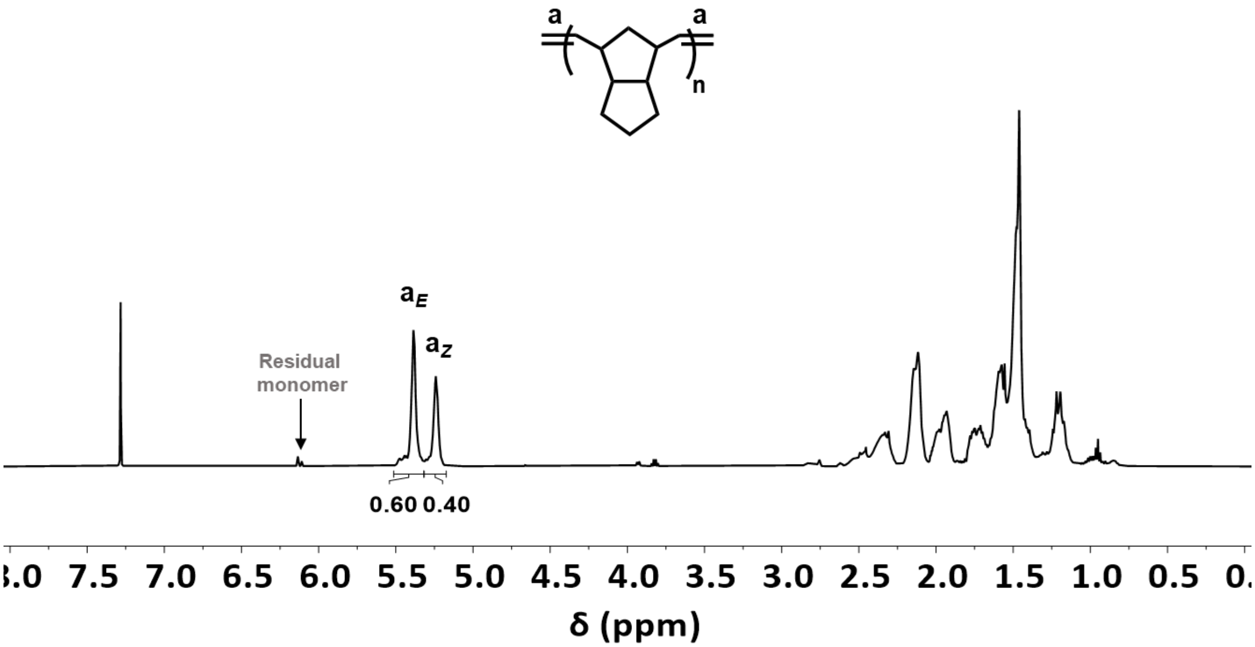

Figure S92: Representative NMR of pDCPD-H_2 post-FROMP for 1000:1:10 with *E* and *Z* configurational signals (a_E and a_Z, respectively) and ratio.

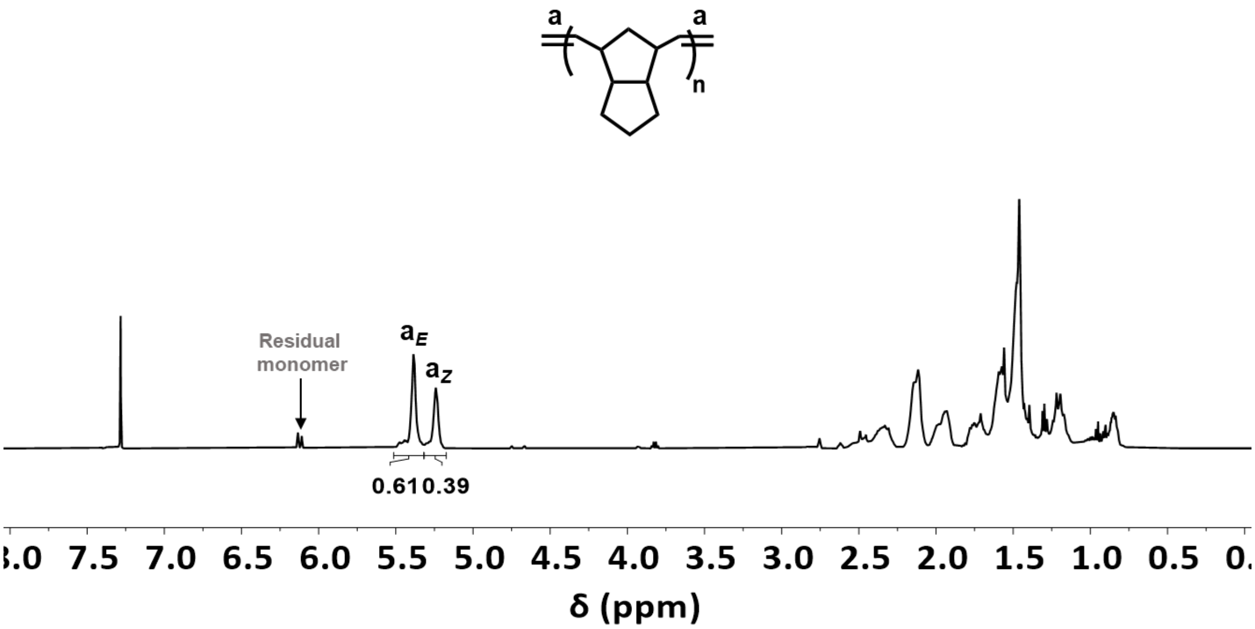

Figure S93: Representative NMR of pDCPD-H_2 post-FROMP for 1000:1:10 **bottom up** with *E* and *Z* configurational signals (a_E and a_Z, respectively) and ratio.

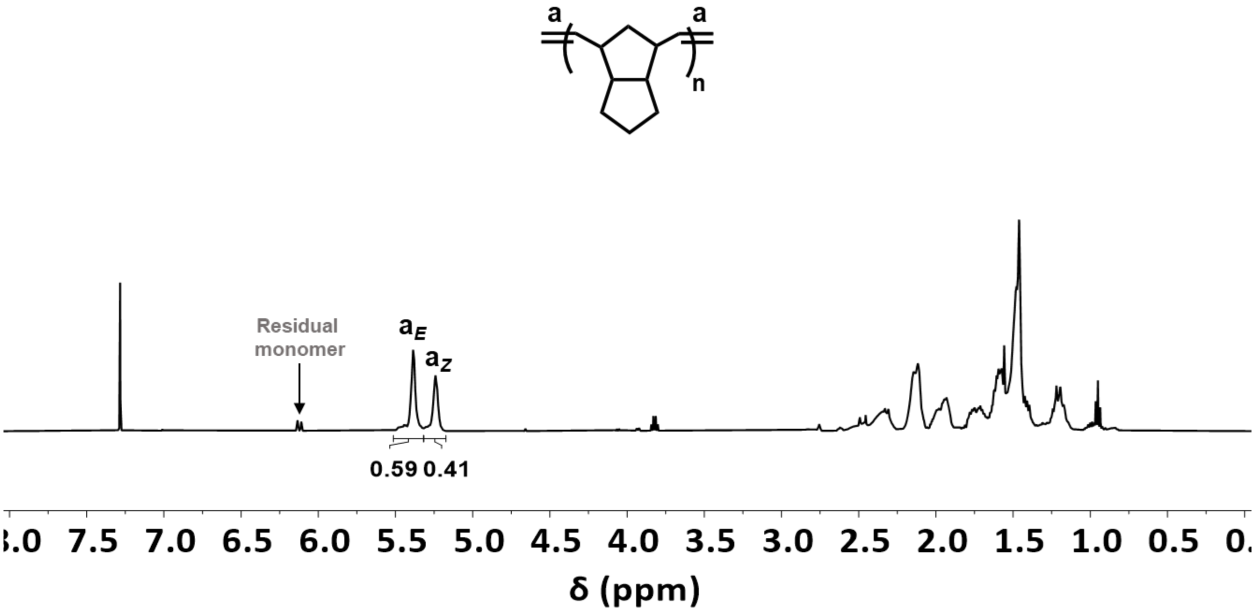

Figure S94: Representative NMR of pDCPD-H_2 post-FROMP for 500:1:10 with *E* and *Z* configurational signals (a_E and a_Z, respectively) and ratio.

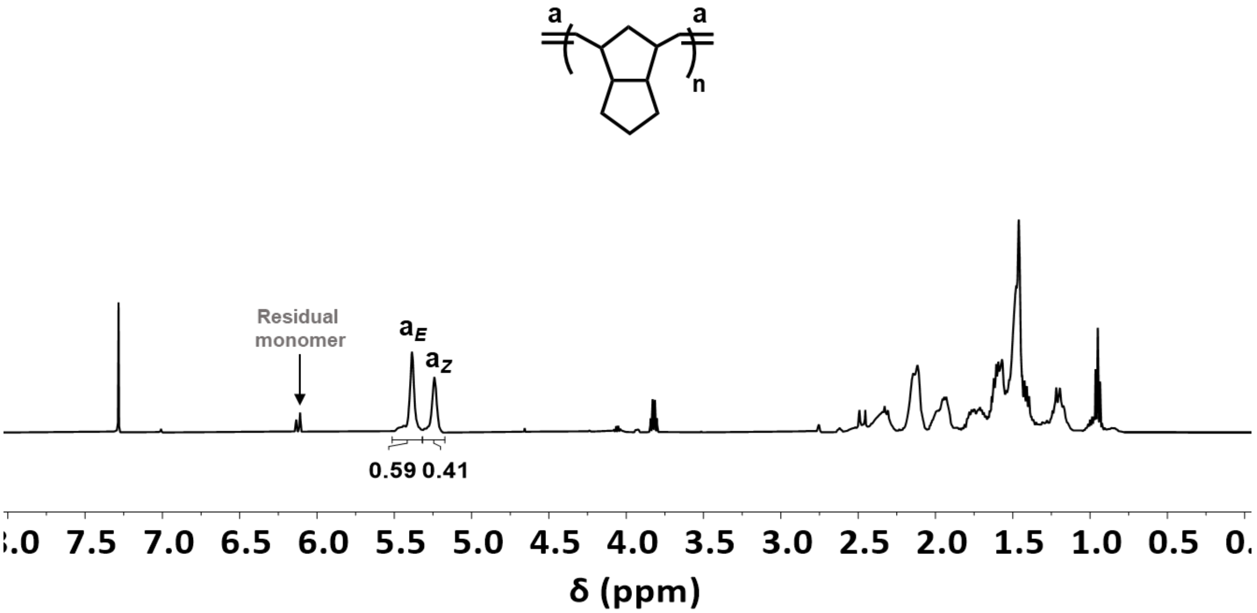

Figure S95: Representative NMR of pDCPD-H_2 post-FROMP for 200:1:10 with *E* and *Z* configurational signals (a_E and a_Z, respectively) and ratio.

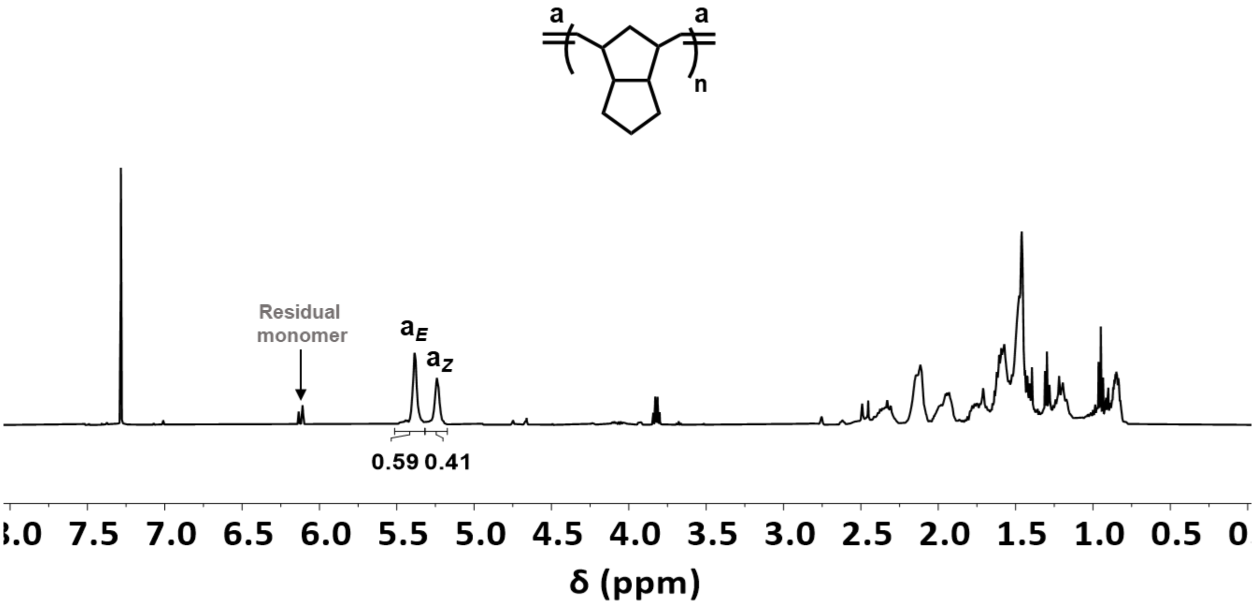

Figure S96: Representative NMR of pDCPD-H_2 post-FROMP for 200:1:10 **bottom up** with *E* and *Z* configurational signals (a_E and a_Z, respectively) and ratio.

Table S4: E/Z configuration ratio from NMR integrations for pDCPD-H_2 post-FROMP at varying loadings.

Monomer (equiv)	Initiator (equiv)	Inhibitor (equiv)	Direction	E config (%)	error
4000	1	1	downward	61.7	0.5
2000	1	1	downward	63.3	0.5
1000	1	1	downward	64.0	0.8
1000	1	1	upward	66.3	0.5
500	1	1	downward	63.7	0.9
4000	1	10	downward	59.0	0.0
4000	1	10	upward	63.3	0.5
2000	1	10	downward	59.7	0.9
1000	1	10	downward	60.0	0.0
1000	1	10	upward	61.0	0.0
500	1	10	downward	58.7	0.5
200	1	10	downward	59.0	0.8
200	1	10	upward	58.7	0.5

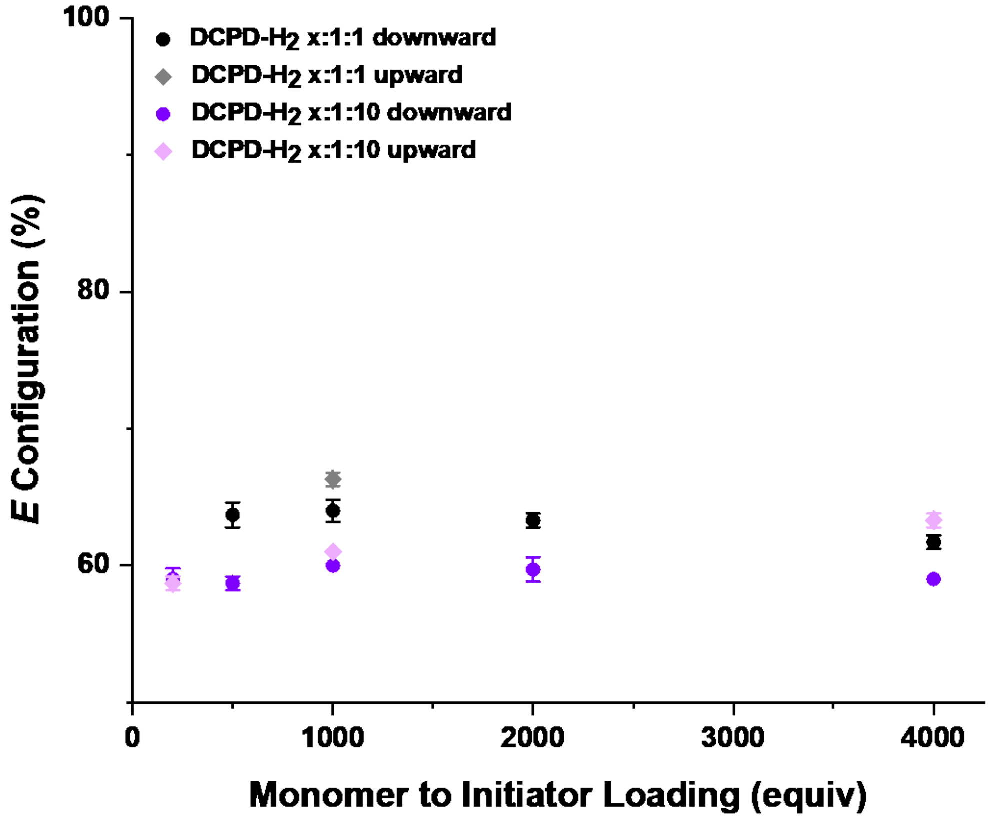

Figure S97: *E* configurational (%) at varying loadings of DCPD-H_2:initiator:inhibitor.

Dynamic Scanning Calorimetry (DSC) Post-FROMP:

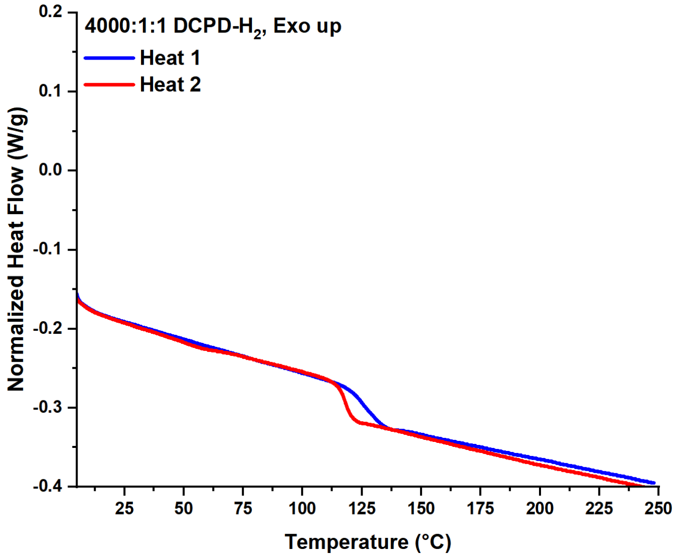

Figure S98: Representative DSC of pDCPD-H2 post-FROMP for 4000:1:1 ($T_{g,\text{Heat 1}}$ = 117 ± 1 °C, $H_{r,\text{residual}}$ =4 ± 6 J/g, $T_{g,\text{Heat 2}}$ = 119 ± 3 °C). Exo up, first (blue) and second heat (red) cycle.

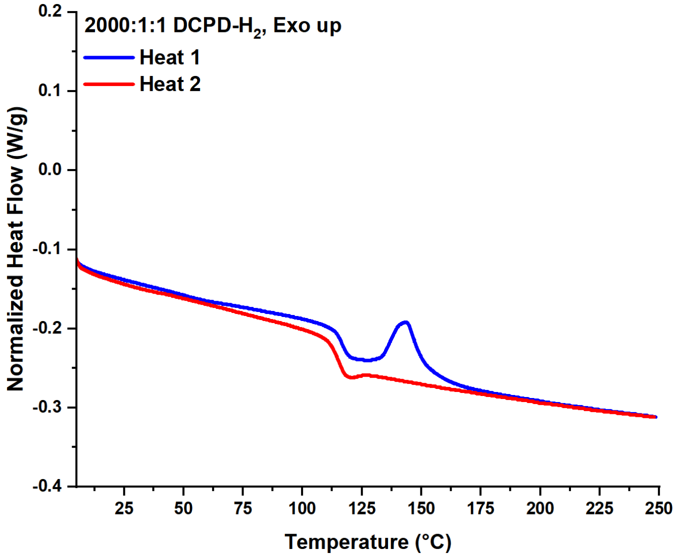

Figure S99: Representative DSC of pDCPD-H2 post-FROMP for 2000:1:1 ($T_{g,\text{Heat 1}}$ = 117 ± 1 °C, $H_{r,\text{residual}}$ = 7 ± 2 J/g, $T_{g,\text{Heat 2}}$ = 114 ± 1 °C). Exo up, first (blue) and second heat (red) cycle.

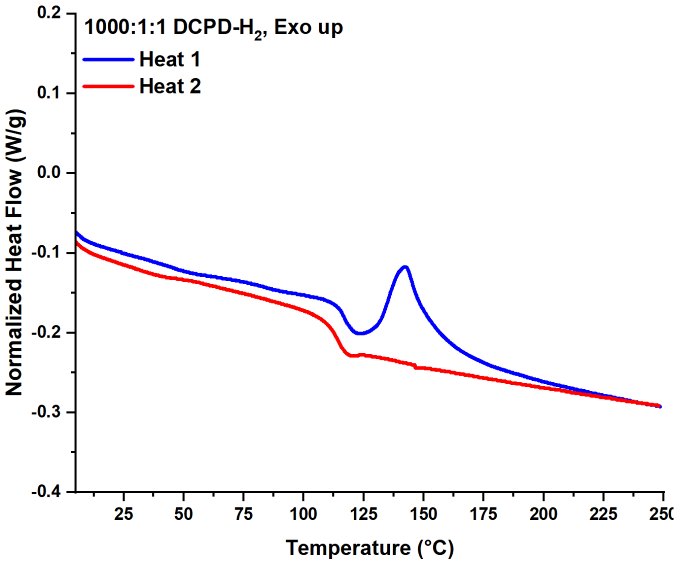

Figure S100: Representative DSC of pDCPD-H_2 post-FROMP for 1000:1:1 ($T_{g,\text{ Heat 1}}$ = 116 ± 1 °C, $H_{r,\text{ residual}}$ = 5 ± 4 J/g, $T_{g,\text{ Heat 2}}$ = 117 ± 6 °C). Exo up, first (blue) and second heat (red) cycle.

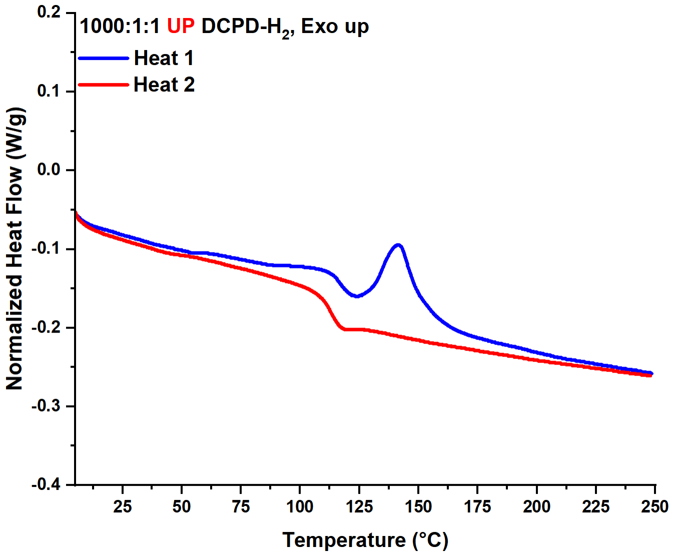

Figure S101: Representative DSC of pDCPD-H_2 post-FROMP for 1000:1:1 **bottom up** ($T_{g,\text{ Heat 1}}$ = 116 ± 1 °C, $H_{r,\text{ residual}}$ = 8 ± 3 J/g, $T_{g,\text{ Heat 2}}$ = 112 ± 1 °C). Exo up, first (blue) and second heat (red) cycle.

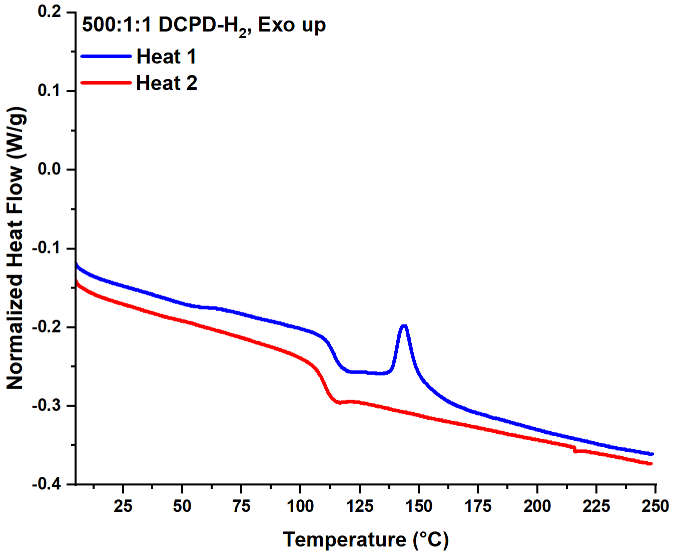

Figure S102: Representative DSC of pDCPD-H_2 post-FROMP for 500:1:1 ($T_{g,\text{ Heat 1}}$ = 111 ± 5 °C, $H_{r,\text{ residual}}$ = 4 ± 2 J/g, $T_{g,\text{ Heat 2}}$ = 106 ± 5 °C). Exo up, first (blue) and second heat (red) cycle.

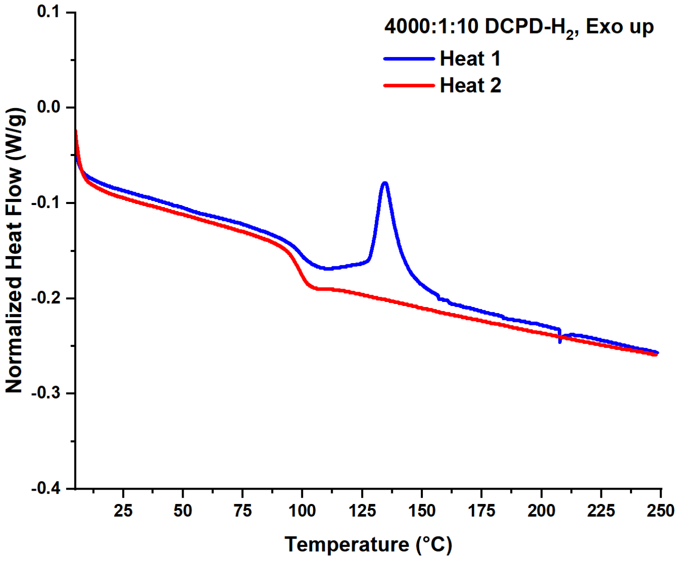

Figure S103: Representative DSC of pDCPD-H_2 post-FROMP for 4000:1:10 ($T_{g,\text{ Heat 1}}$ = 104 ± 4 °C, $H_{r,\text{ residual}}$ = 6 ± 1 J/g, $T_{g,\text{ Heat 2}}$ = 102 ± 5 °C). Exo up, first (blue) and second heat (red) cycle.

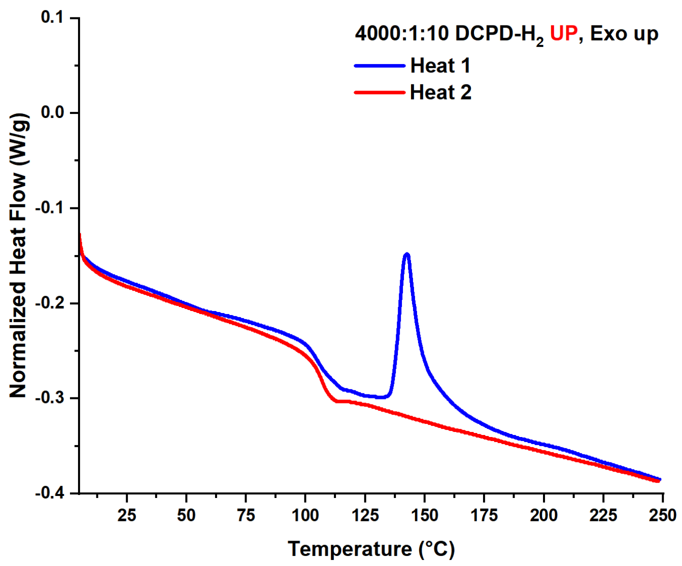

Figure S104: Representative DSC of pDCPD-H_2 post-FROMP for 4000:1:10 **bottom up** ($T_{g,\text{ Heat 1}}$ = 106 ± 3 °C, $H_{r,\text{ residual}}$ = 7 ± 5 J/g, $T_{g,\text{ Heat 2}}$ = 107 ± 5 °C). Exo up, first (blue) and second heat (red) cycle.

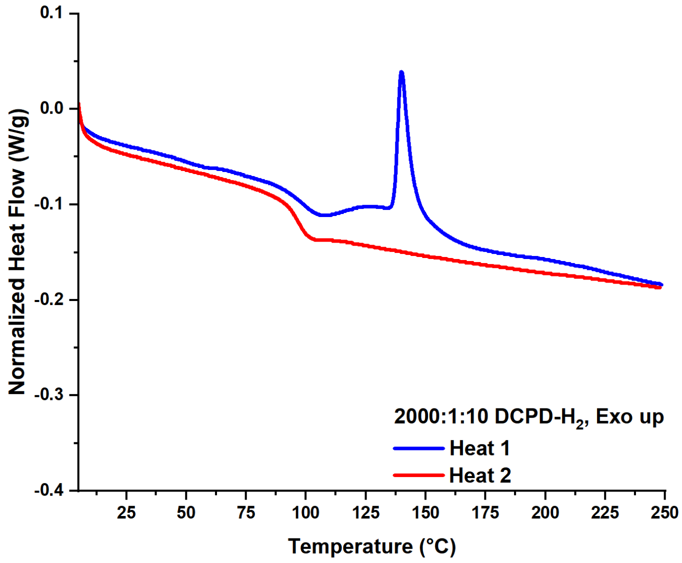

Figure S105: Representative DSC of pDCPD-H_2 post-FROMP for 2000:1:10 ($T_{g,\text{ Heat 1}}$ = 99 ± 2 °C, $H_{r,\text{ residual}}$ = 7 ± 2 J/g, $T_{g,\text{ Heat 2}}$ = 97 ± 2 °C). Exo up, first (blue) and second heat (red) cycle.

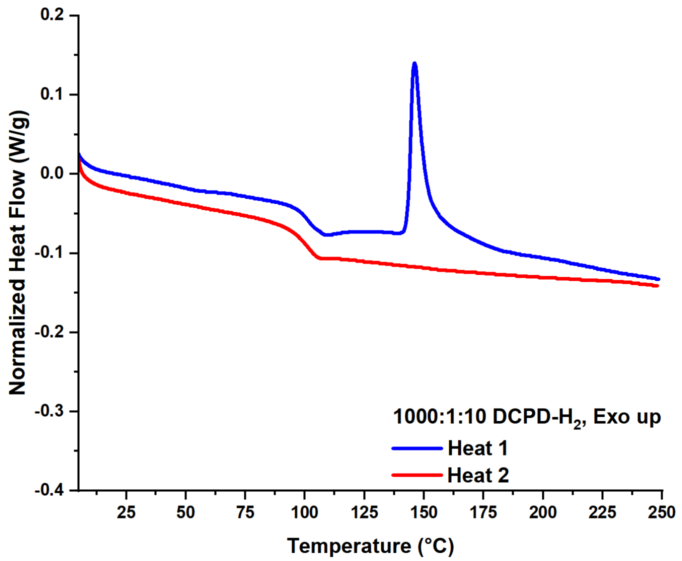

Figure S106: Representative DSC of pDCPD-H$_2$ post-FROMP for 1000:1:10 ($T_{g,\text{ Heat 1}}$ = 101 ± 1 °C, $H_{r,\text{ residual}}$ = 8 ± 3 J/g, $T_{g,\text{ Heat 2}}$ = 98 ± 2 °C). Exo up, first (blue) and second heat (red) cycle.

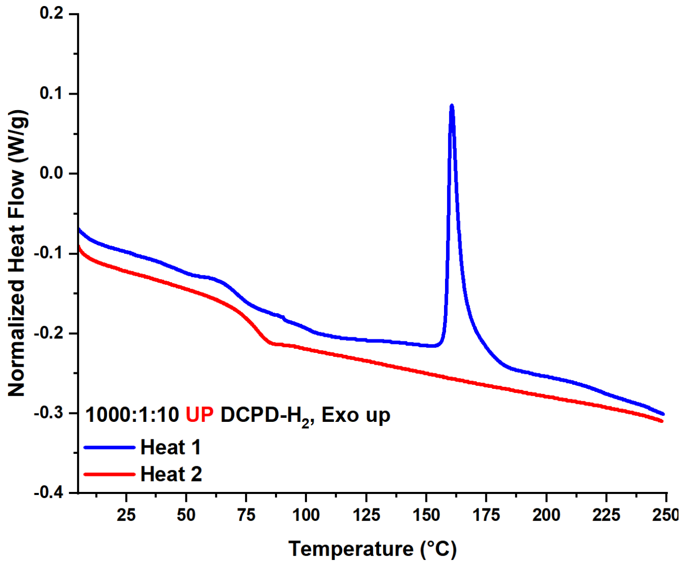

Figure S107: Representative DSC of pDCPD-H$_2$ post-FROMP for 1000:1:10 **bottom up** ($T_{g,\text{ Heat 1}}$ = 76 ± 4 °C, $H_{r,\text{ residual}}$ = 7 ± 4 J/g, $T_{g,\text{ Heat 2}}$ = 80 ± 2 °C). Exo up, first (blue) and second heat (red) cycle.

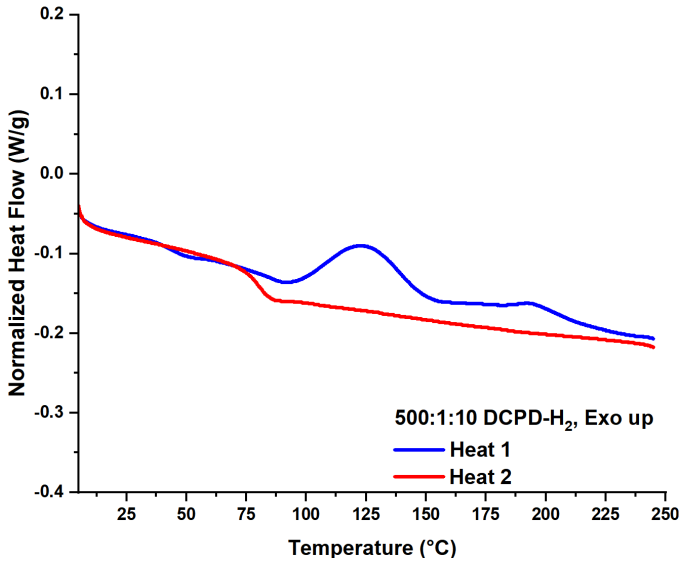

Figure S108: Representative DSC of pDCPD-H2 post-FROMP for 500:1:10 ($T_{g,\text{ Heat 1}}$ = 44 ± 1 °C, $H_{r,\text{ residual}}$ = 7 ± 3 J/g, $T_{g,\text{ Heat 2}}$ = 77 ± 1 °C). Exo up, first (blue) and second heat (red) cycle.

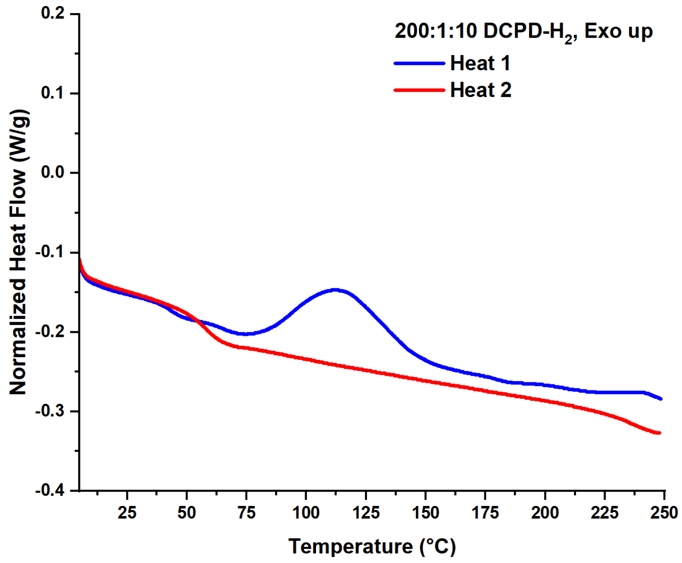

Figure S109: Representative DSC of pDCPD-H2 post-FROMP for 200:1:10 ($T_{g,\text{ Heat 1}}$ = 41 ± 1 °C, $H_{r,\text{ residual}}$ = 18 ± 1 J/g, $T_{g,\text{ Heat 2}}$ = 59 ± 2 °C). Exo up, first (blue) and second heat (red) cycle.

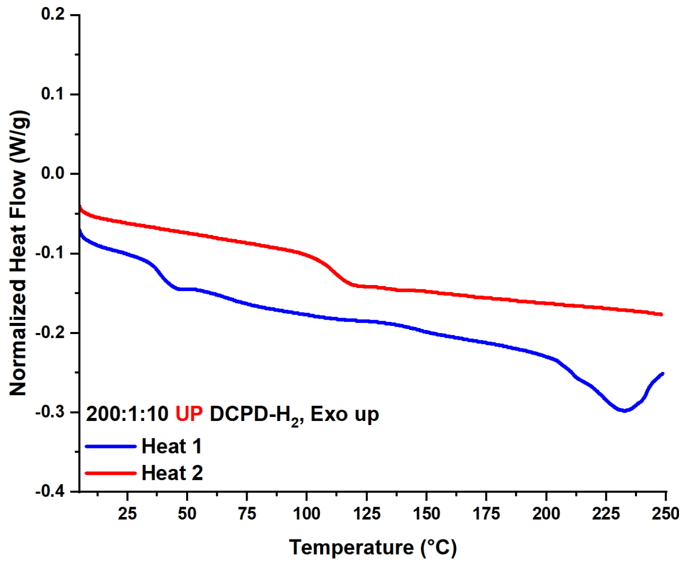

Figure S110: Representative DSC of pDCPD-H_2 post-FROMP for 200:1:10 **bottom up** ($T_{g,\,\mathrm{Heat\ 1}}$ = 37 ± 1 °C, $H_{r,\,\mathrm{residual}}$ = *N/A* - *boiling endotherm observed in all samples*, $T_{g,\,\mathrm{Heat\ 2}}$ = 109 ± 5 °C). Exo up, first (blue) and second heat (red) cycle.

Table S5: Glass Transition (T_g) for first and second heat for pDCPD-H_2 post-FROMP at varied loadings.

Monomer (equiv)	Initiator (equiv)	Inhibitor (equiv)	Direction	$T_{g,\,\mathrm{Heat\ 1}}$ (°C)	error	$T_{g,\,\mathrm{Heat\ 2}}$ (°C)	error
4000	1	1	downward	117	1	119	3
2000	1	1	downward	117	1	114	1
1000	1	1	downward	116	1	117	6
1000	1	1	upward	116	1	112	1
500	1	1	downward	111	5	106	5
4000	1	10	downward	104	4	102	5
4000	1	10	upward	106	3	107	5
2000	1	10	downward	99	2	97	2
1000	1	10	downward	101	1	98	2
1000	1	10	upward	76	4	80	2
500	1	10	downward	44	1	77	1
200	1	10	downward	41	1	59	2
200	1	10	upward	37	1	109	5

Table S6: Residual heat of reaction ($H_{r,\text{ residual}}$) and percent monomer conversion for pDCPD-H_2 post-FROMP at varied loadings.

Monomer (equiv)	Initiator (equiv)	Inhibitor (equiv)	Direction	$H_{r,\text{ residual}}$ (J/g)	error	Calculated Conversion (%)	error
4000	1	1	downward	4	6	99	2
2000	1	1	downward	7	2	98	1
1000	1	1	downward	6	4	99	1
1000	1	1	upward	8	3	98	1
500	1	1	downward	4	2	99	1
4000	1	10	downward	6	1	98	1
4000	1	10	upward	7	5	98	1
2000	1	10	downward	7	2	98	1
1000	1	10	downward	8	3	98	1
1000	1	10	upward	7	4	98	1
500	1	10	downward	7	3	98	1
200	1	10	downward	18	1	95	1
200	1	10	upward	*Boiled*	N/A	N/A	N/A

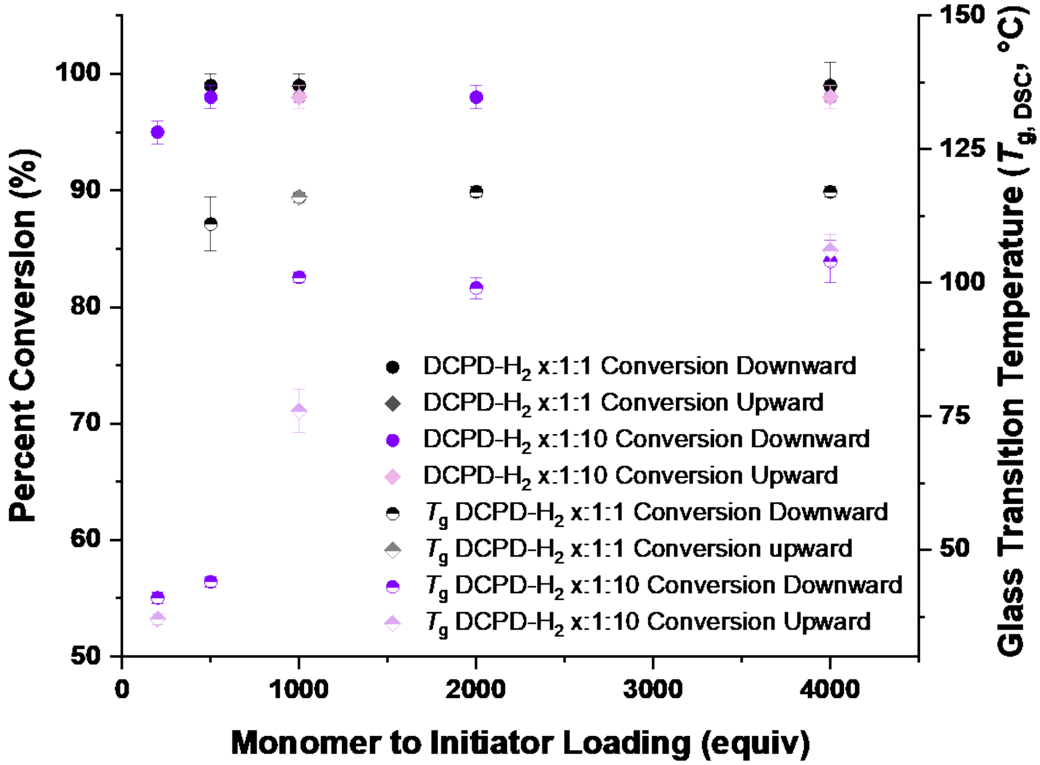

Figure S111: Conversion from DSC and glass transition temperatures ($T_{g,\ Heat\ 1}$) from DSC for pDCPD-H_2.

Copolymerization:

Synthesis of Butyl Norbornene Imide (NBI_4):

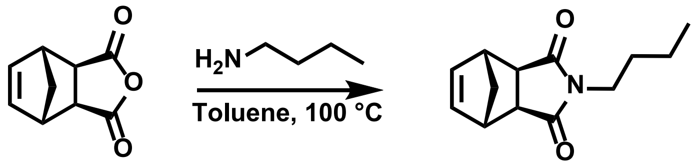

Norbornene-exo-dicarboxylic anhydride (30.0 g, 183 mmol, 1.00 equiv) was added to an oven-dried 1 L round-bottomed flask. Next, 600 mL of anhydrous toluene was added to the reaction vessel and a stir bar was equipped. While stirring at room temperature, 18.7 mL of *n*-butylamine (189 mmol, 1.04 equiv) was added slowly over approximately 10 min. After full addition of amine, the flask was equipped with a reflux condenser and the slurry was placed in a preheated 110 °C oil bath for 16 h. The reaction vessel was allowed to cool to room temperature, and the toluene was rotary evaporated yielding a clear, slightly yellow viscous oil. The crude product was reconstituted in 500 mL of ethyl acetate and added to a 2 L separatory funnel. The organic solution was then washed with 1 M hydrochloric acid (3 x 500 mL), deionized water (2 x 500 mL), and brine (2 x 500 mL). The organic layer was collected, dried with sodium sulfate, rotary evaporated, and dried under reduced pressure for 48h to yield a clear, slightly yellow viscous oil (38.8 g, 96.8% yield).

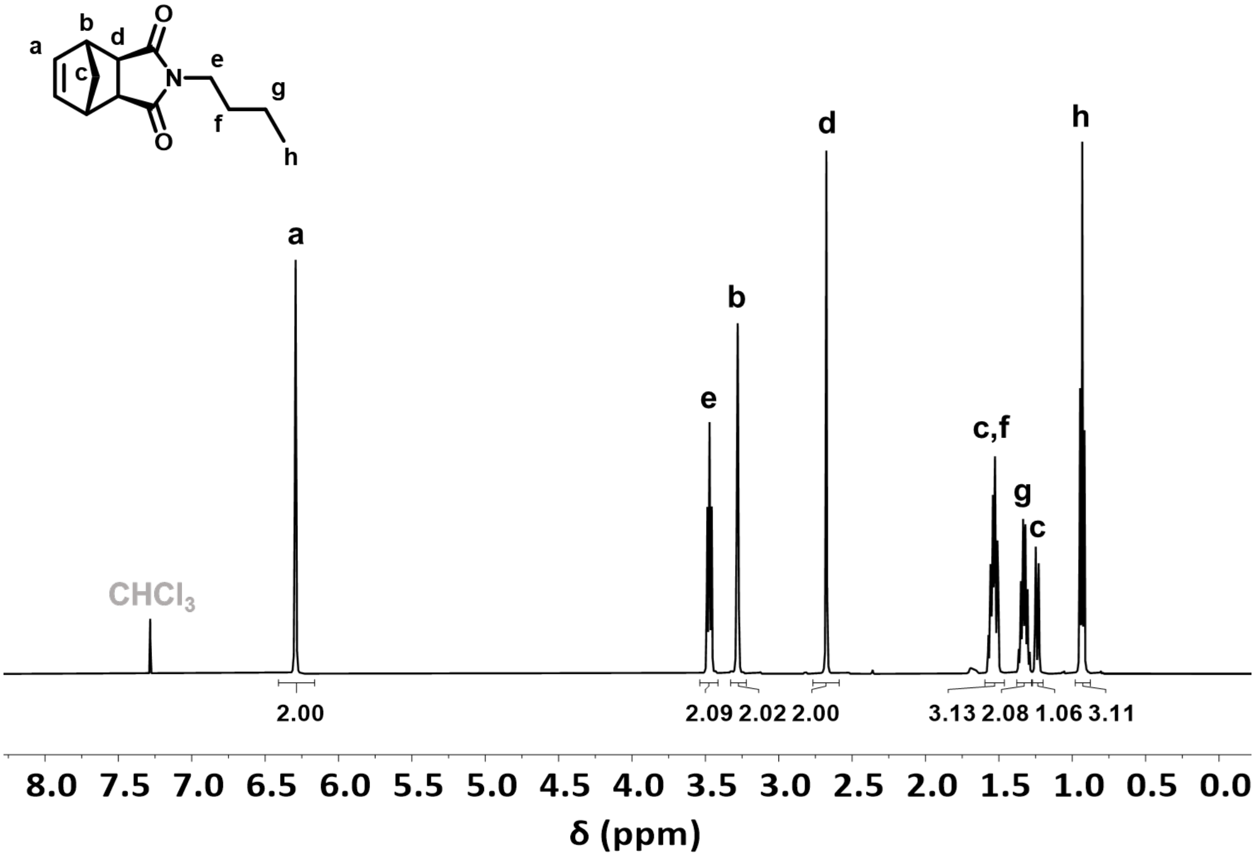

Figure S112: ^{1}H NMR of butyl norbornene imide (NBI_4).

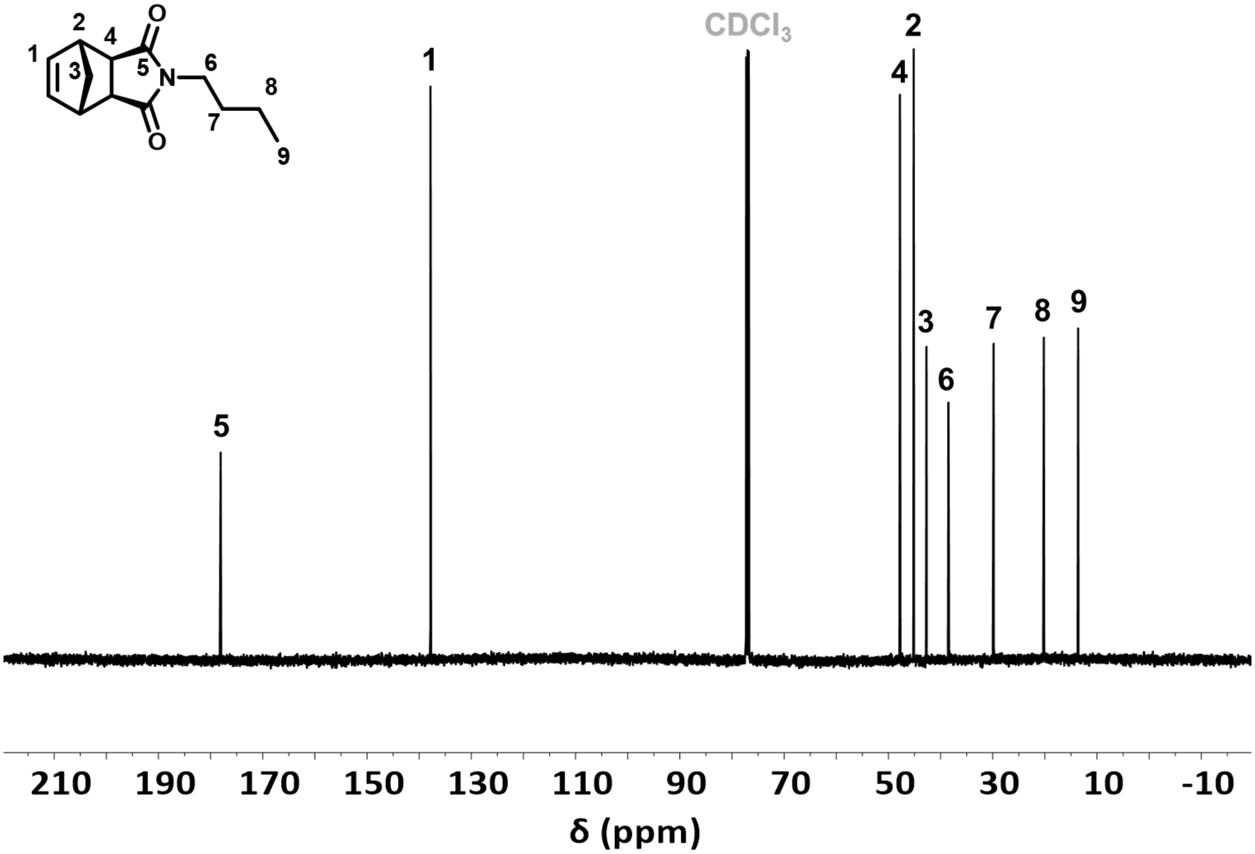

Figure S113: ^{13}C NMR of butyl norbornene imide (NBI_4).

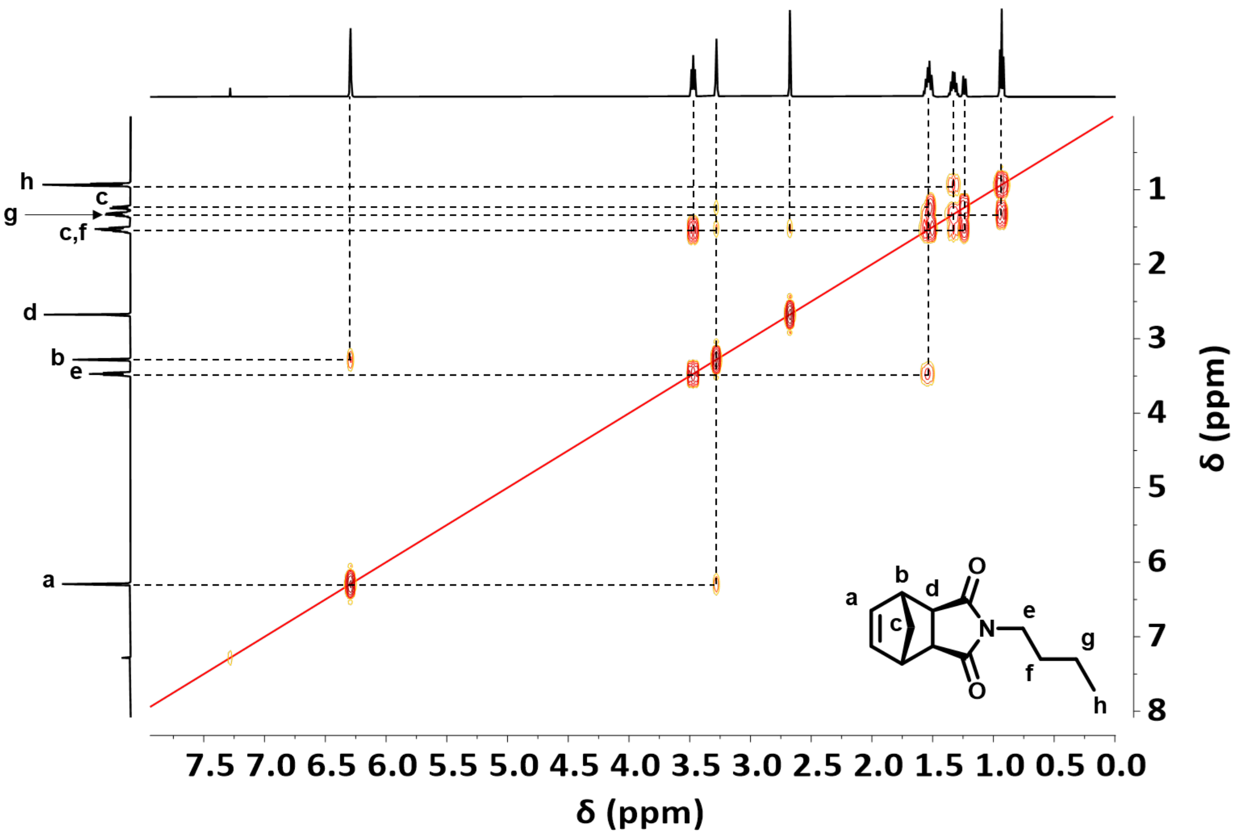

Figure S114: COSY NMR of butyl norbornene imide (NBI_4).

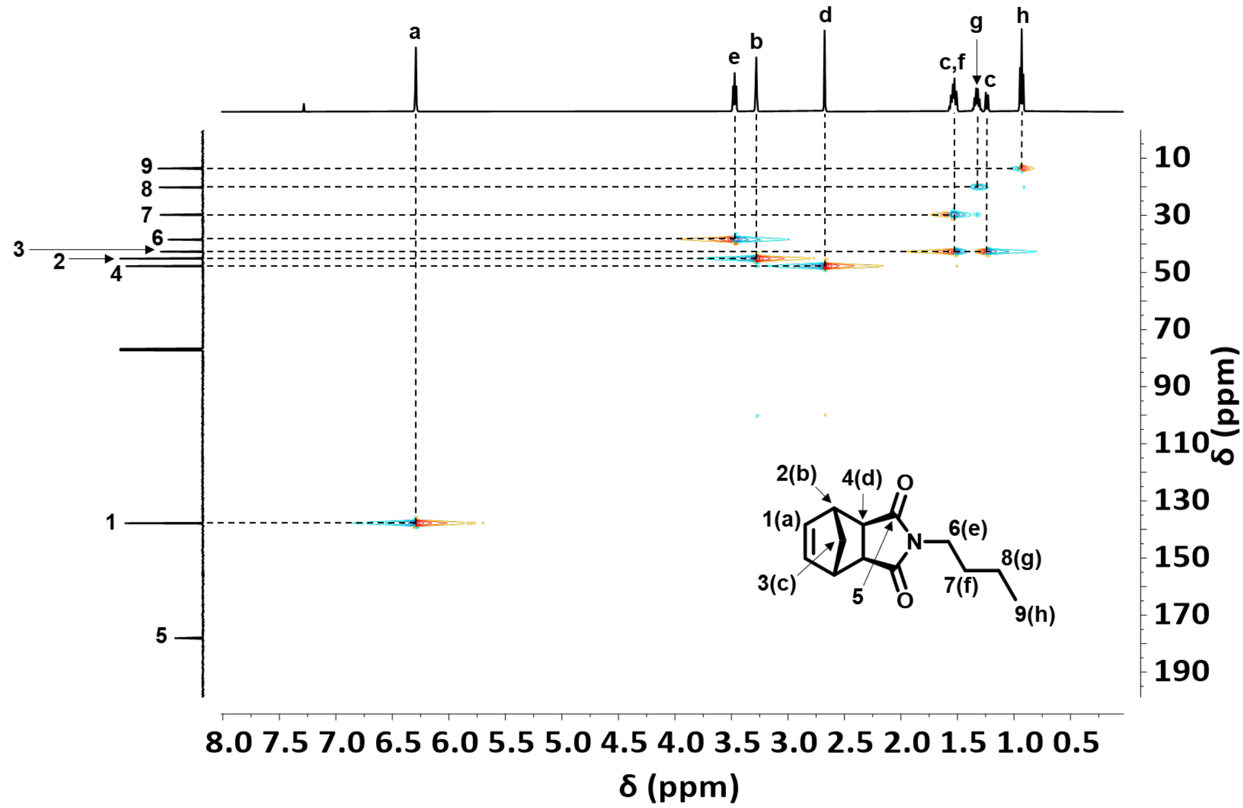

Figure S115: HSQC NMR of butyl norbornene imide (NBI_4).

General Procedure for DCPD-H_2 and NBI_4 Resin Preparation:

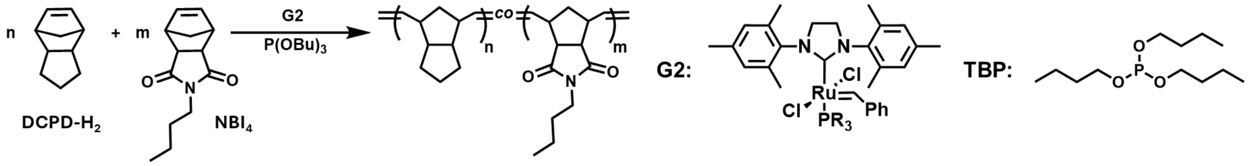

Grubbs' 2nd generation initiator was massed (G2, **w** mg, 1.00 equiv.) in a 5 mL vial prior to the addition of tributyl phosphite (TBP, **x** µL, **y** equiv.). The mixture was dissolved in monomer (*DCPD-H_2 & NBI_4*, 500 mg, **z** equiv.) and sonicated for up to 5 minutes. The resulting solution was transferred 6 mm diameter test tube. Test tube samples were initiated at the top or bottom of the resin using a preheated sottering iron creating a descending front.

10% NBI_4:

<u>4000:1:1 Monomer:Initiator:Inhibitor</u>

G2: **w** = 0.74 mg

TBP: **x** = 0.20 µL, **y** = 1.00 equiv.

Monomer Mixture: **z** = 4,000 equiv. (DCPD-H_2: 3,600 equiv., NBI_4: 400 equiv.)

<u>2000:1:1 Monomer:Initiator:Inhibitor</u>

G2: **w** = 1.49 mg

TBP: **x** = 0.50 μL, **y** = 1.00 equiv.

Monomer Mixture: **z** = 2,000 equiv. (DCPD-H_2: 1,800 equiv., NBI_4: 200 equiv.)

<u>1000:1:1 Monomer:Initiator:Inhibitor</u>

G2: **w** = 2.97 mg

TBP: **x** = 0.95 μL, **y** = 1.00 equiv.

Monomer Mixture: **z** = 1,000 equiv. (DCPD-H_2: 1,900 equiv., NBI_4: 100 equiv.)

<u>500:1:1 Monomer:Initiator:Inhibitor</u>

G2: **w** = 5.95 mg

TBP: **x** = 1.90 μL, **y** = 1.00 equiv.

Monomer Mixture: **z** = 500 equiv. (DCPD-H_2: 450 equiv., NBI_4: 50 equiv.)

<u>200:1:1 Monomer:Initiator:Inhibitor</u>

G2: **w** = 14.87 mg

TBP: **x** = 4.8 μL, **y** = 1.00 equiv.

Monomer Mixture: **z** = 200 equiv. (DCPD-H_2: 180 equiv., NBI_4: 20 equiv.)

25% NBI_4:

<u>4000:1:1 Monomer:Initiator:Inhibitor</u>

G2: **w** = 0.68 mg

TBP: **x** = 0.20 μL, **y** = 1.00 equiv.

Monomer Mixture: **z** = 4,000 equiv. (DCPD-H_2: 3,000 equiv., NBI_4: 1,000 equiv.)

<u>2000:1:1 Monomer:Initiator:Inhibitor</u>

G2: **w** = 1.37 mg

TBP: **x** = 0.45 μL, **y** = 1.00 equiv.

Monomer Mixture: **z** = 2,000 equiv. (DCPD-H_2: 1,500 equiv., NBI_4: 500 equiv.)

<u>1000:1:1 Monomer:Initiator:Inhibitor</u>

G2: **w** = 2.73 mg

TBP: **x** = 0.90 μL, **y** = 1.00 equiv.

Monomer Mixture: **z** = 1,000 equiv. (DCPD-H_2: 750 equiv., NBI_4: 250 equiv.)

<u>500:1:1 Monomer:Initiator:Inhibitor</u>

G2: **w** = 5.47 mg

TBP: **x** = 1.75 μL, **y** = 1.00 equiv.

Monomer Mixture: **z** = 500 equiv. (DCPD-H_2: 375 equiv., NBI_4: 125 equiv.)

<u>200:1:1 Monomer:Initiator:Inhibitor</u>

G2: **w** = 13.65 mg

TBP: **x** = 4.40 μL, **y** = 1.00 equiv.

Monomer Mixture: **z** = 200 equiv. (DCPD-H_2: 150 equiv., NBI_4: 50 equiv.)

50% NBI_4:

<u>4000:1:1 Monomer:Initiator:Inhibitor</u>

G2: **w** = 0.60 mg

TBP: **x** = 0.20 μL, **y** = 1.00 equiv.

Monomer Mixture: **z** = 4,000 equiv. (DCPD-H_2: 2,000 equiv., NBI_4: 2,000 equiv.)

<u>2000:1:1 Monomer:Initiator:Inhibitor</u>

G2: **w** = 1.20 mg

TBP: **x** = 0.40 μL, **y** = 1.00 equiv.

Monomer Mixture: **z** = 2,000 equiv. (DCPD-H_2: 1,000 equiv., NBI_4: 1,000 equiv.)

<u>1000:1:1 Monomer:Initiator:Inhibitor</u>

G2: **w** = 2.40 mg

TBP: **x** = 0.80 μL, **y** = 1.00 equiv.

Monomer Mixture: **z** = 1,000 equiv. (DCPD-H_2: 500 equiv., NBI_4: 500 equiv.)

<u>500:1:1 Monomer:Initiator:Inhibitor</u>

G2: **w** = 4.81 mg

TBP: **x** = 1.55 μL, **y** = 1.00 equiv.

Monomer Mixture: **z** = 500 equiv. (DCPD-H_2: 250 equiv., NBI_4: 250 equiv.)

<u>200:1:1 Monomer:Initiator:Inhibitor</u>

G2: **w** = 12.01 mg

TBP: **x** = 3.85 μL, **y** = 1.00 equiv.

Monomer Mixture: **z** = 200 equiv. (DCPD-H_2: 100 equiv., NBI_4: 100 equiv.)

4000:1:2 Monomer:Initiator:Inhibitor

G2: **w** = 0.60 mg

TBP: **x** = 0.40 µL, **y** = 2.00 equiv.

Monomer Mixture: **z** = 1,000 equiv. (DCPD-H_2: 500 equiv., NBI_4: 500 equiv.)

1000:1:2 Monomer:Initiator:Inhibitor

G2: **w** = 2.40 mg

TBP: **x** = 1.55 µL, **y** = 2.00 equiv.

Monomer Mixture: **z** = 500 equiv. (DCPD-H_2: 250 equiv., NBI_4: 250 equiv.)

200:1:2 Monomer:Initiator:Inhibitor

G2: **w** = 12.01 mg

TBP: **x** = 7.70 µL, **y** = 2.00 equiv.

Monomer Mixture: **z** = 200 equiv. (DCPD-H_2: 100 equiv., NBI_4: 100 equiv.)

Front velocities and Timelapse:

10 mol% NBI_4:

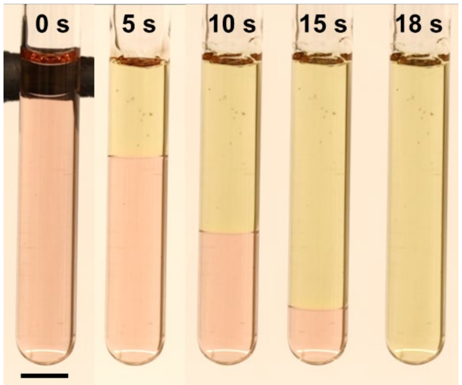

Figure S116: Representative timelapse of 4000:1:1 Monomer:G2:TBP (10:90 NBI_4:DCPD-H_2). The scale bar is 5 mm.

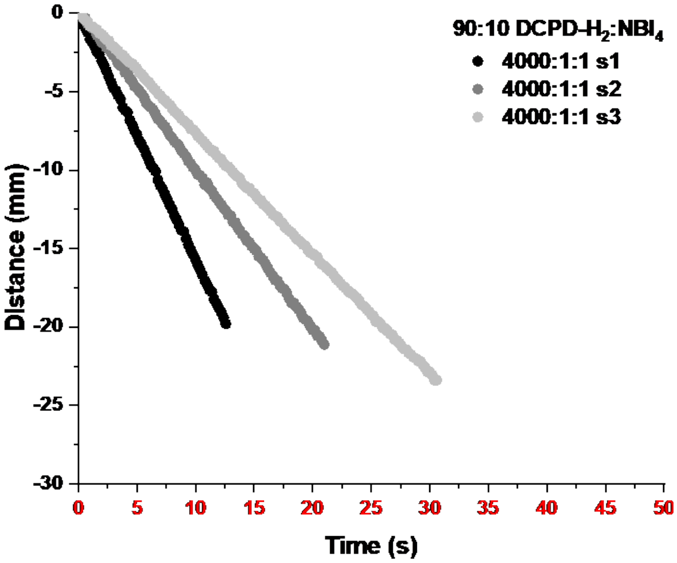

Figure S117: Front tracking of 4000:1:1 Monomer:G2:TBP (10:90 NBI_4:DCPD-H_2) in triplicate (v_f = 1.1 ± 0.3 mm/s).

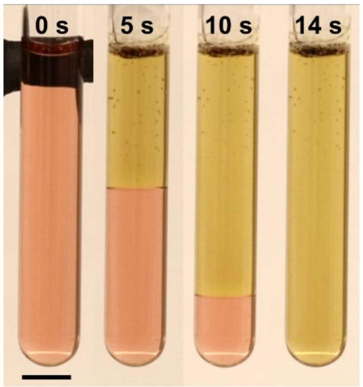

Figure S118: Representative timelapse of 2000:1:1 Monomer:G2:TBP (10:90 NBI_4:DCPD-H_2). The scale bar is 5 mm.

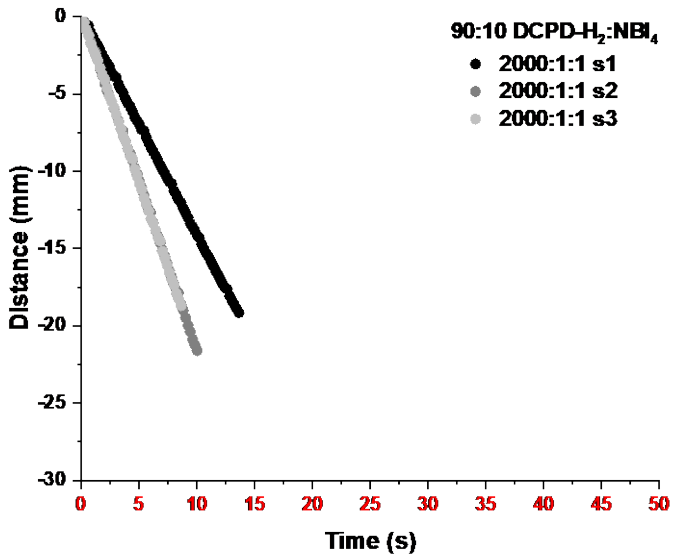

Figure S119: Front tracking of 2000:1:1 Monomer:G2:TBP (10:90 NBI_4:DCPD-H_2) in triplicate (v_f = 2.0 ± 0.4 mm/s).

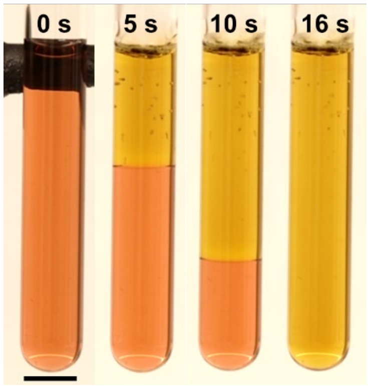

Figure S120: Representative timelapse of 1000:1:1 Monomer:G2:TBP (10:90 NBI_4:DCPD-H_2). The scale bar is 5 mm.

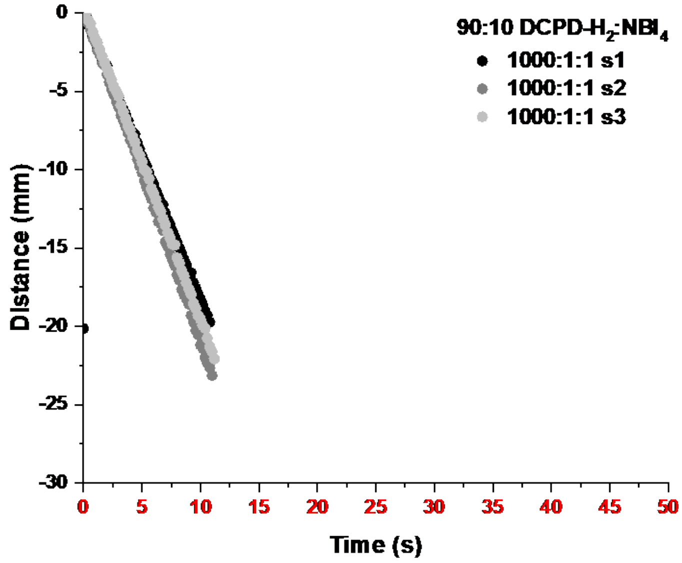

Figure S121: Front tracking of 1000:1:1 Monomer:G2:TBP (10:90 NBI_4:DCPD-H_2) in triplicate (v_f = 2.0 ± 0.1 mm/s).

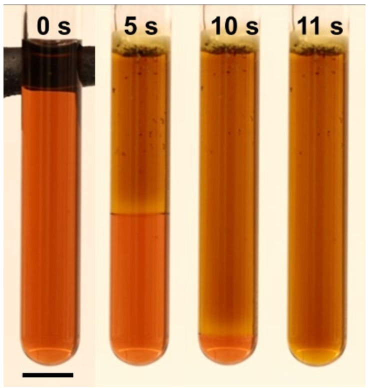

Figure S122: Representative timelapse of 500:1:1 Monomer:G2:TBP (10:90 NBI_4:DCPD-H_2). The scale bar is 5 mm.

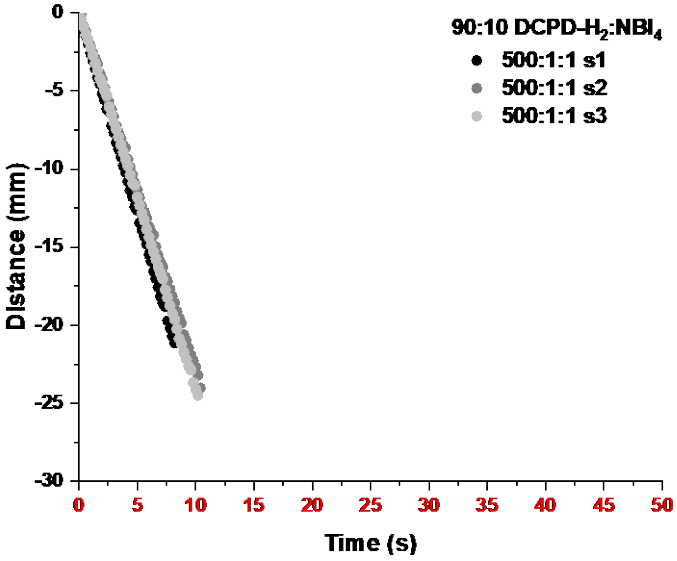

Figure S123: Front tracking of 500:1:1 Monomer:G2:TBP (10:90 NBI_4:DCPD-H_2) in triplicate (v_f = 2.4 ± 0.1 mm/s).

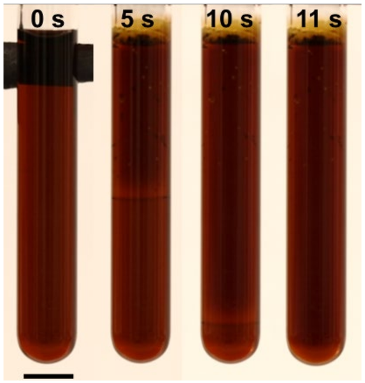

Figure S124: Representative timelapse of 200:1:1 Monomer:G2:TBP (10:90 NBI_4:DCPD-H_2). The scale bar is 5 mm.

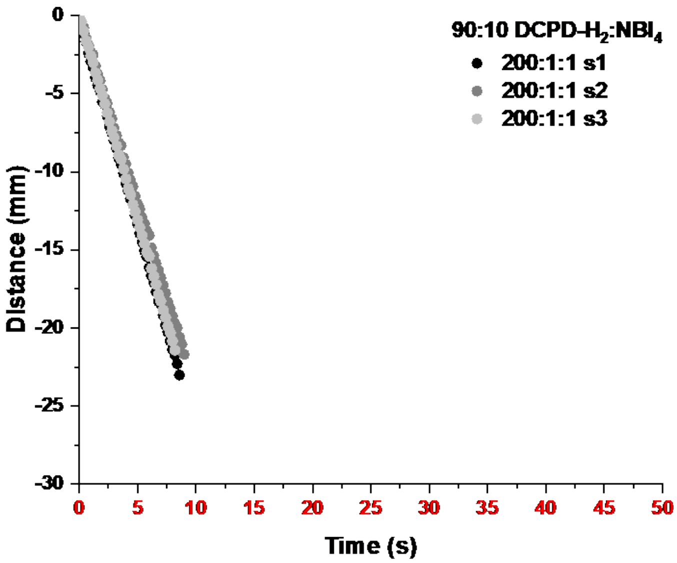

Figure S125: Front tracking of 200:1:1 Monomer:G2:TBP (10:90 NBI_4:DCPD-H_2) in triplicate (v_f = 2.6 ± 0.1 mm/s).

<u>25 mol% NBI_4:</u>

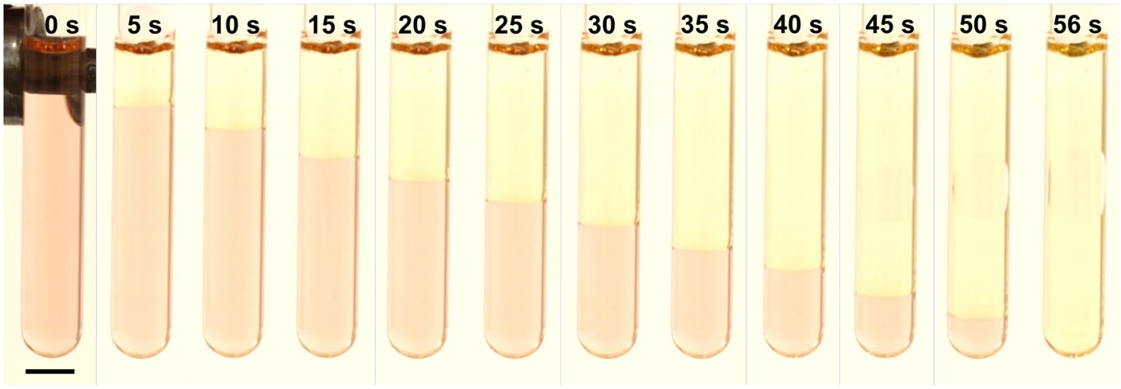

Figure S126: Representative timelapse of 4000:1:1 Monomer:G2:TBP (25:75 NBI_4:DCPD-H_2). The scale bar is 5 mm.

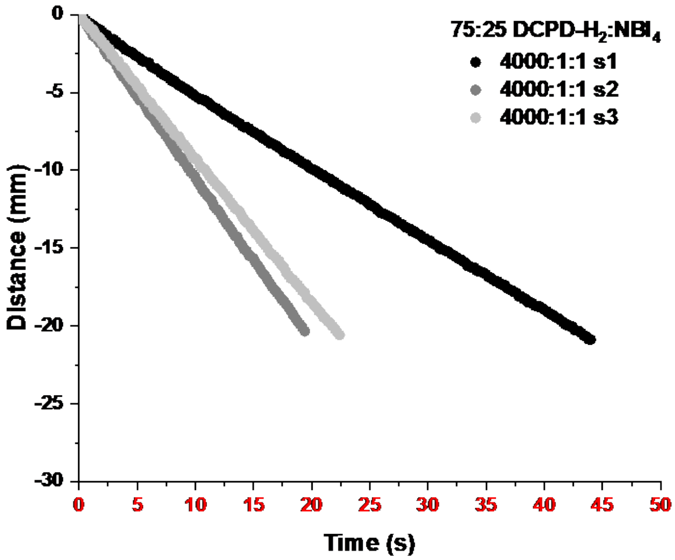

Figure S127: Front tracking of 4000:1:1 Monomer:G2:TBP (25:75 NBI_4:DCPD-H_2) in triplicate (v_f = 0.8 ± 0.3 mm/s).

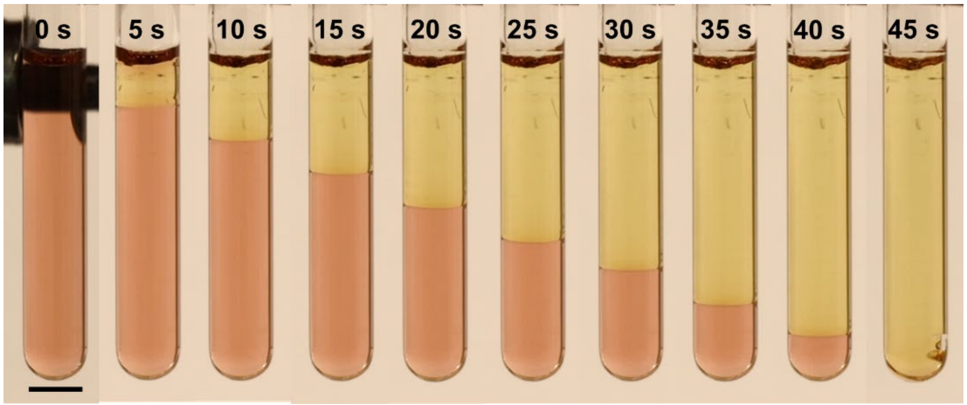

Figure S128: Representative timelapse of 2000:1:1 Monomer:G2:TBP (25:75 NBI_4:DCPD-H_2). The scale bar is 5 mm.

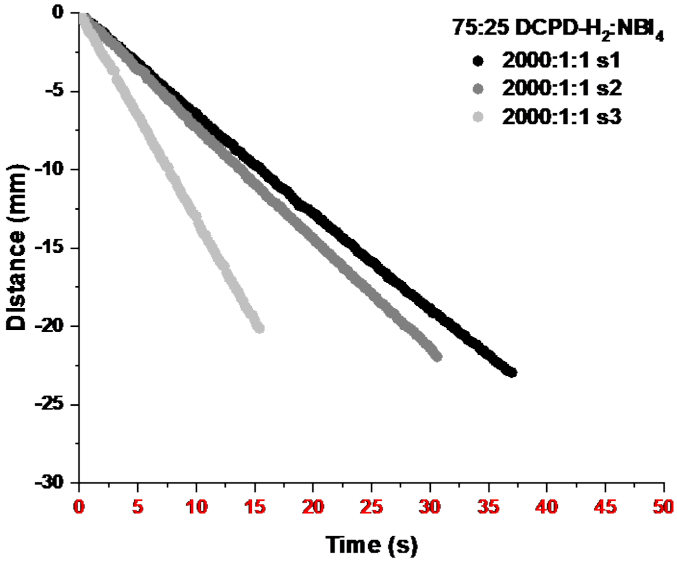

Figure S129: Front tracking of 2000:1:1 Monomer:G2:TBP (25:75 NBI_4:DCPD-H_2) in triplicate (v_f = 0.9 ± 0.3 mm/s).

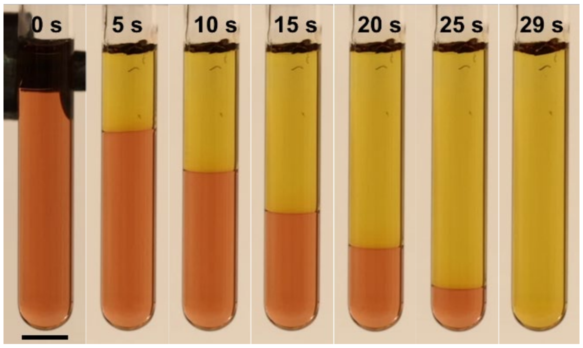

Figure S130: Representative timelapse of 1000:1:1 Monomer:G2:TBP (25:75 NBI_4:DCPD-H_2). The scale bar is 5 mm.

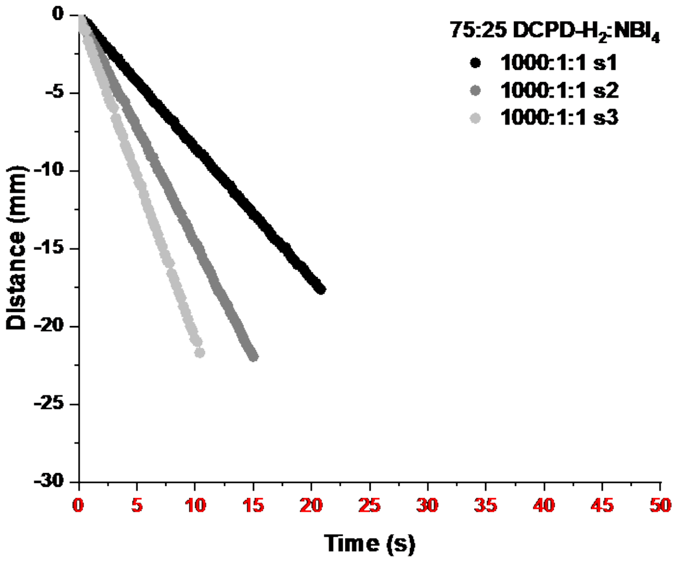

Figure S131: Front tracking of 1000:1:1 Monomer:G2:TBP (25:75 NBI_4:DCPD-H_2) in triplicate (v_f = 1.5 ± 0.5 mm/s).

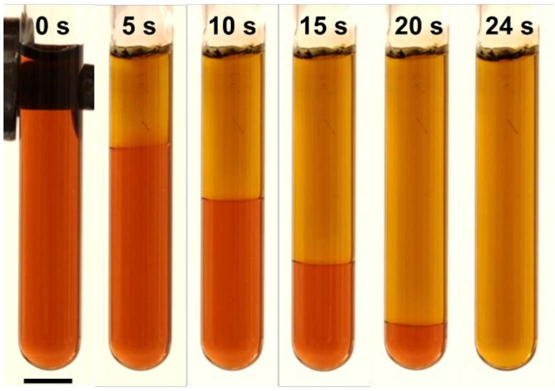

Figure S132: Representative timelapse of 500:1:1 Monomer:G2:TBP (25:75 NBI_4:DCPD-H_2). The scale bar is 5 mm.

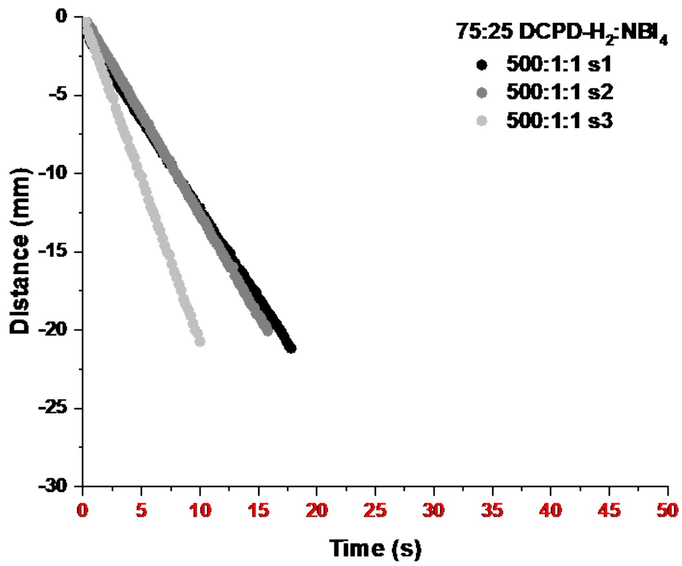

Figure S133: Front tracking of 500:1:1 Monomer:G2:TBP (25:75 NBI_4:DCPD-H_2) in triplicate (v_f = 1.5 ± 0.4 mm/s).

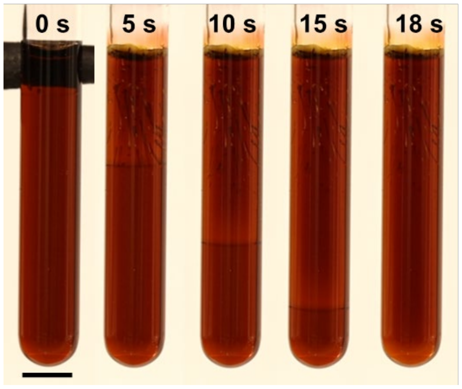

Figure S134: Representative timelapse of 200:1:1 Monomer:G2:TBP (25:75 NBI_4:DCPD-H_2). The scale bar is 5 mm.

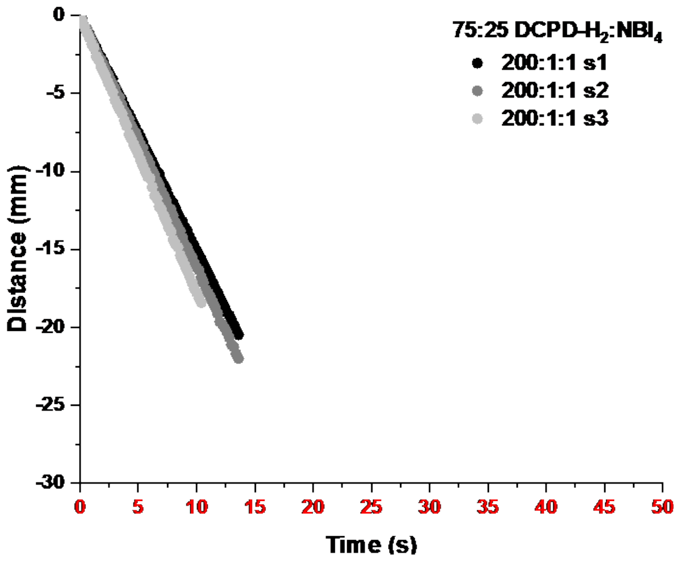

Figure S135: Front tracking of 200:1:1 Monomer:G2:TBP (25:75 NBI_4:DCPD-H_2) in triplicate (v_f = 2.6 ± 0.1 mm/s).

<u>50 mol% NBI_4:</u>

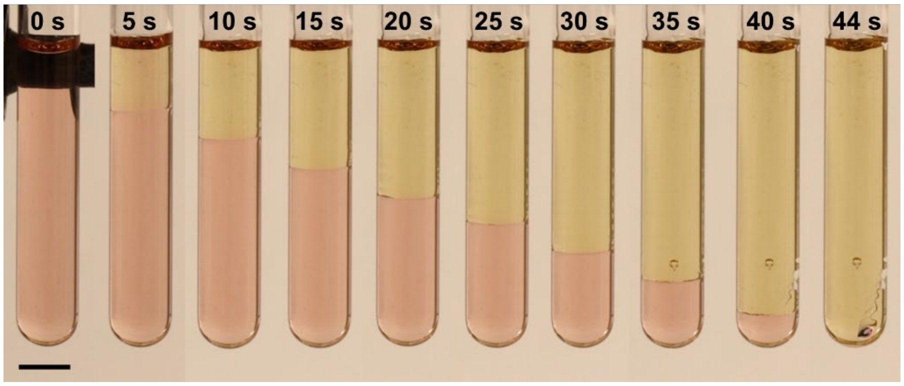

Figure S136: Representative timelapse of 4000:1:1 Monomer:G2:TBP (50:50 NBI_4:DCPD-H_2). The scale bar is 5 mm.

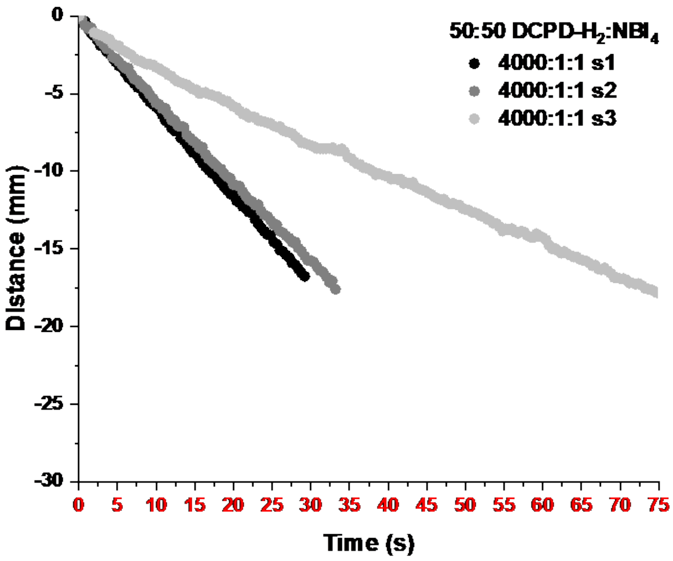

Figure S137: Front tracking of 4000:1:1 Monomer:G2:TBP (50:50 NBI_4:DCPD-H_2) in triplicate (v_f = 0.4 ± 0.2 mm/s).

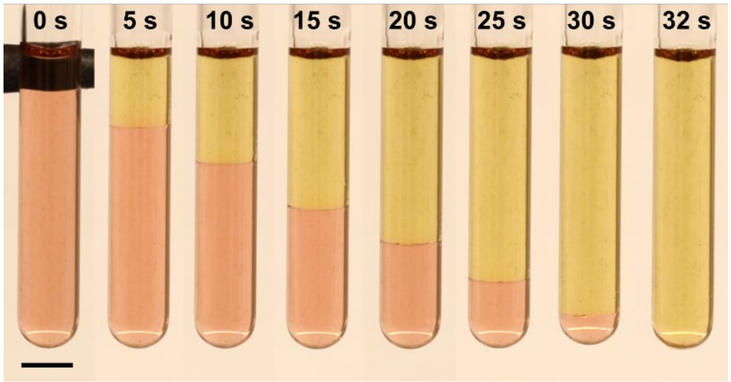

Figure S138: Representative timelapse of 2000:1:1 Monomer:G2:TBP (50:50 NBI_4:DCPD-H_2). The scale bar is 5 mm.

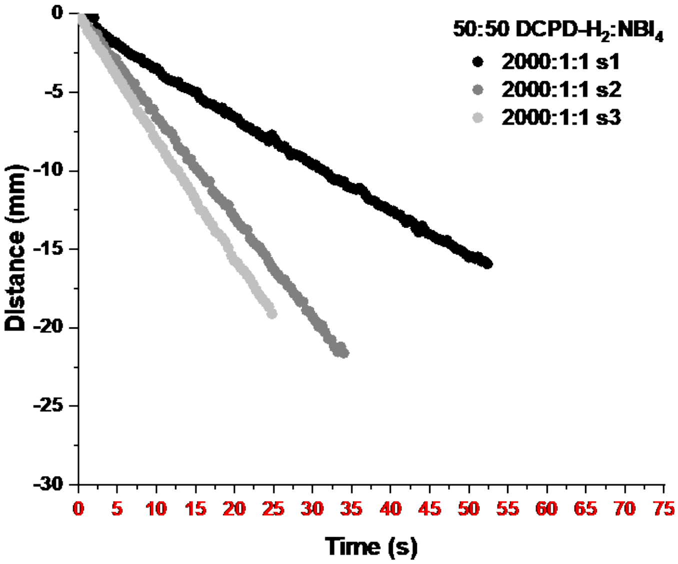

Figure S139: Front tracking of 2000:1:1 Monomer:G2:TBP (50:50 NBI_4:DCPD-H_2) in triplicate (v_f = 0.6 ± 0.2 mm/s).

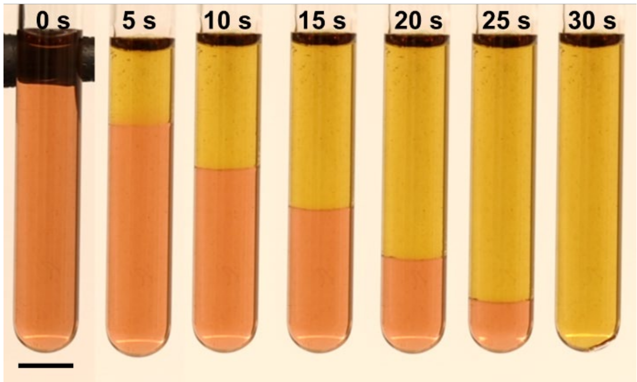

Figure S140: Representative timelapse of 1000:1:1 Monomer:G2:TBP (50:50 NBI_4:DCPD-H_2). The scale bar is 5 mm.

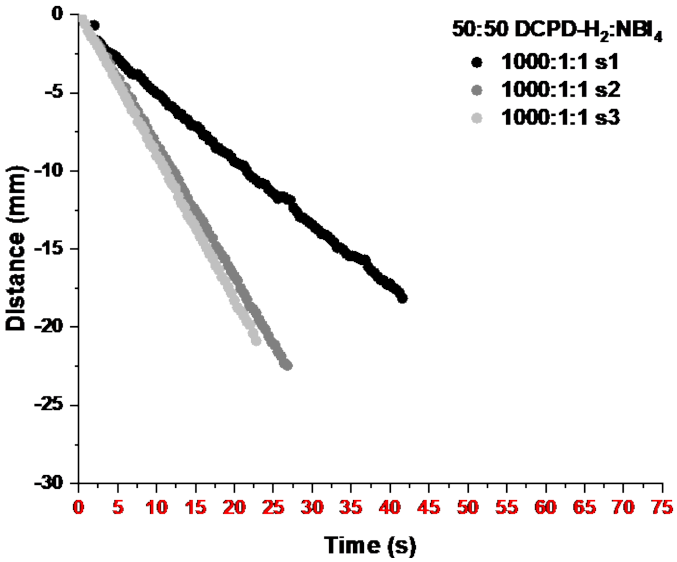

Figure S141: Front tracking of 1000:1:1 Monomer:G2:TBP (50:50 NBI_4:DCPD-H_2) in triplicate (v_f = 0.7 ± 0.2 mm/s).

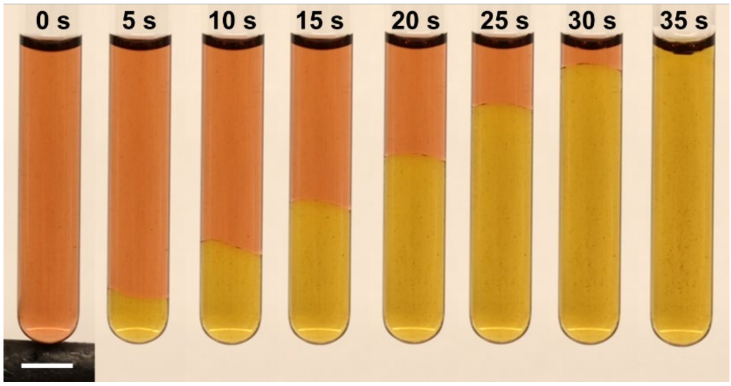

Figure S142: Representative timelapse of 1000:1:1 Monomer:G2:TBP **bottom up** (50:50 NBI_4:DCPD-H_2). The scale bar is 5 mm.

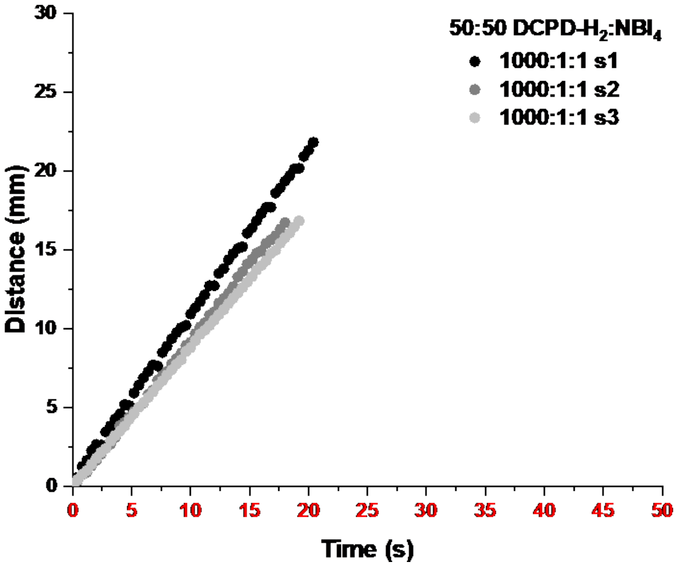

Figure S143: Front tracking of 1000:1:1 Monomer:G2:TBP (50:50 NBI_4:DCPD-H_2) **bottom up** in triplicate (v_f = 0.96 ± 0.07 mm/s).

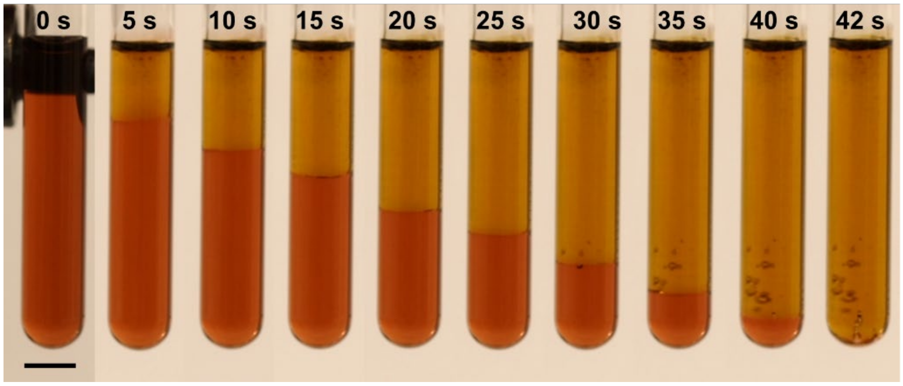

Figure S144: Representative timelapse of 500:1:1 Monomer:G2:TBP (50:50 NBI_4:DCPD-H_2). The scale bar is 5 mm.

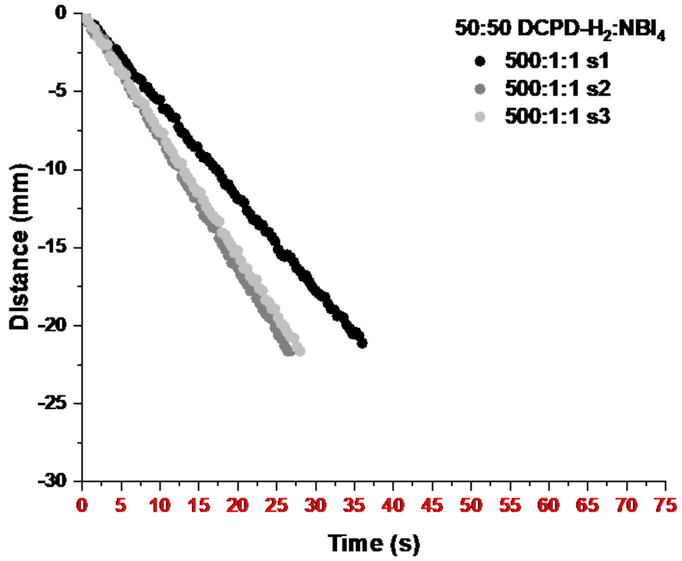

Figure S145: Front tracking of 500:1:1 Monomer:G2:TBP (50:50 NBI_4:DCPD-H_2) in triplicate (v_f = 0.7 ± 0.1 mm/s).

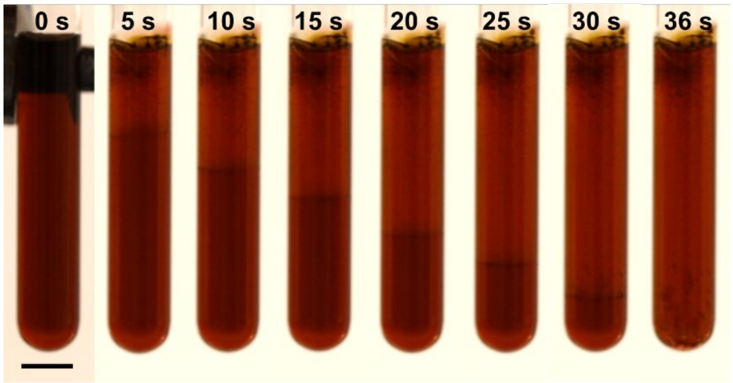

Figure S146: Representative timelapse of 200:1:1 Monomer:G2:TBP (50:50 NBI_4:DCPD-H_2). The scale bar is 5 mm.

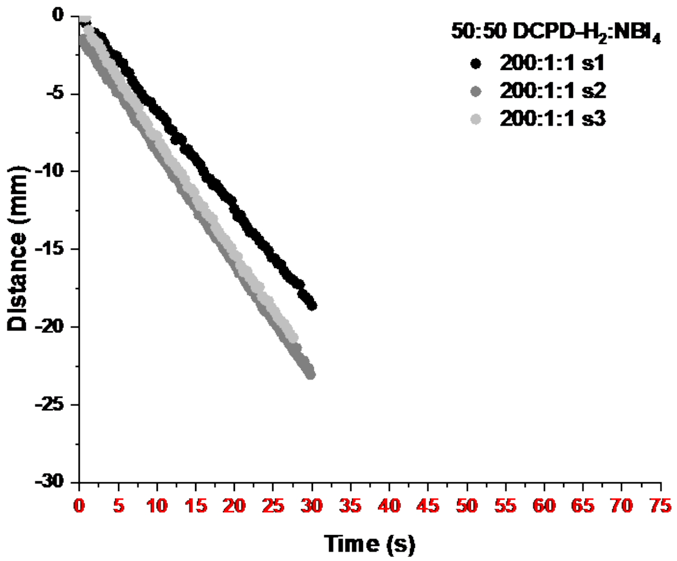

Figure S147: Front tracking of 200:1:1 Monomer:G2:TBP (50:50 NBI_4:DCPD-H_2) in triplicate (v_f = 0.7± 0.06 mm/s).

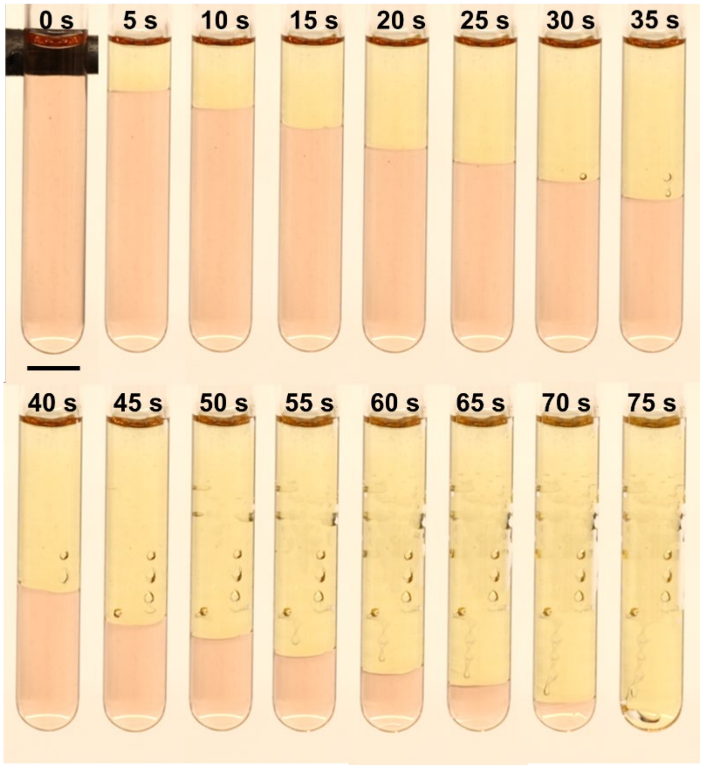

Figure S148: Representative timelapse of 4000:1:2 Monomer:G2:TBP (50:50 NBI_4:DCPD-H_2). The scale bar is 5 mm.

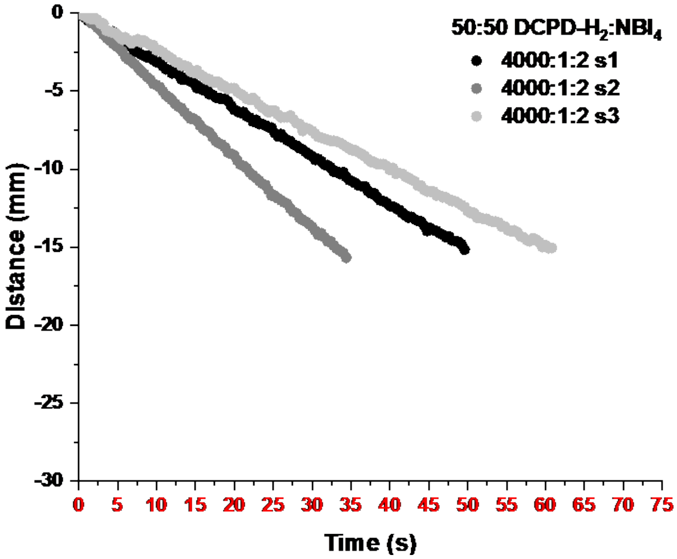

Figure S149: Front tracking of 4000:1:2 Monomer:G2:TBP (50:50 NBI_4:DCPD-H_2) in triplicate (v_f = 0.34 ± 0.09 mm/s).

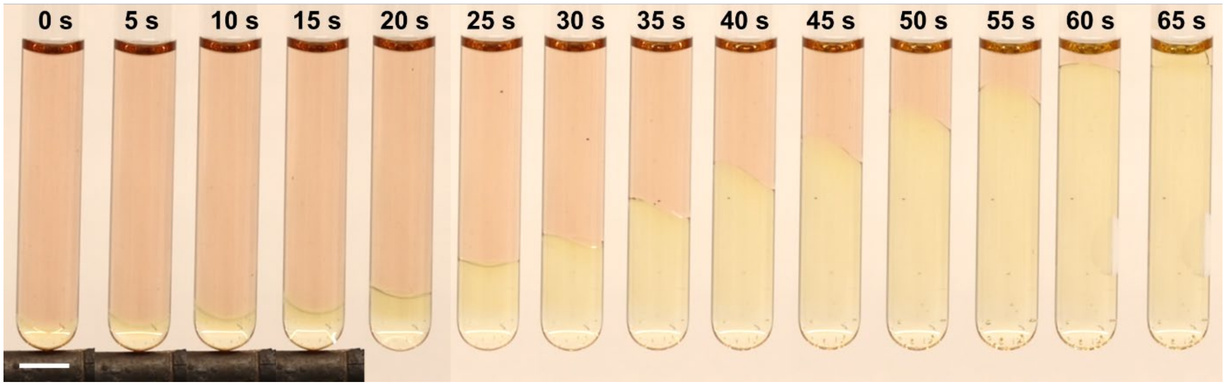

Figure S150: Representative timelapse of 4000:1:2 Monomer:G2:TBP **bottom up** (50:50 NBI_4:DCPD-H_2). The scale bar is 5 mm.

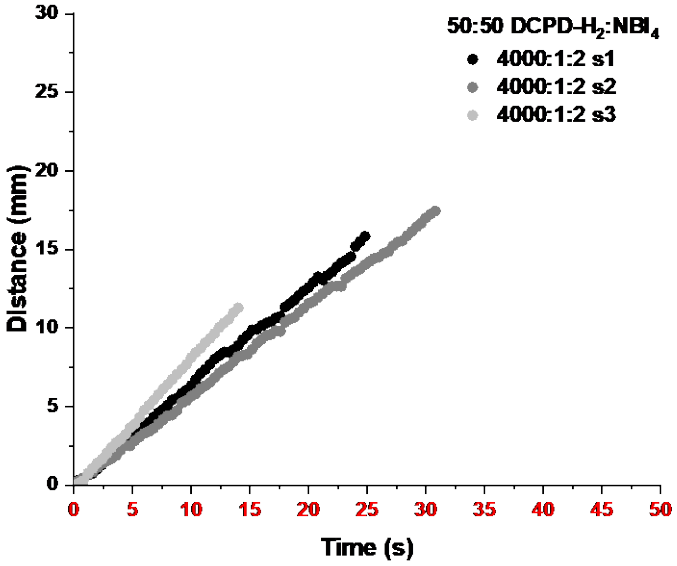

Figure S151: Front tracking of 4000:1:2 Monomer:G2:TBP (50:50 NBI_4:DCPD-H_2) **bottom up** in triplicate (v_f = 0.67 ± 0.11 mm/s).

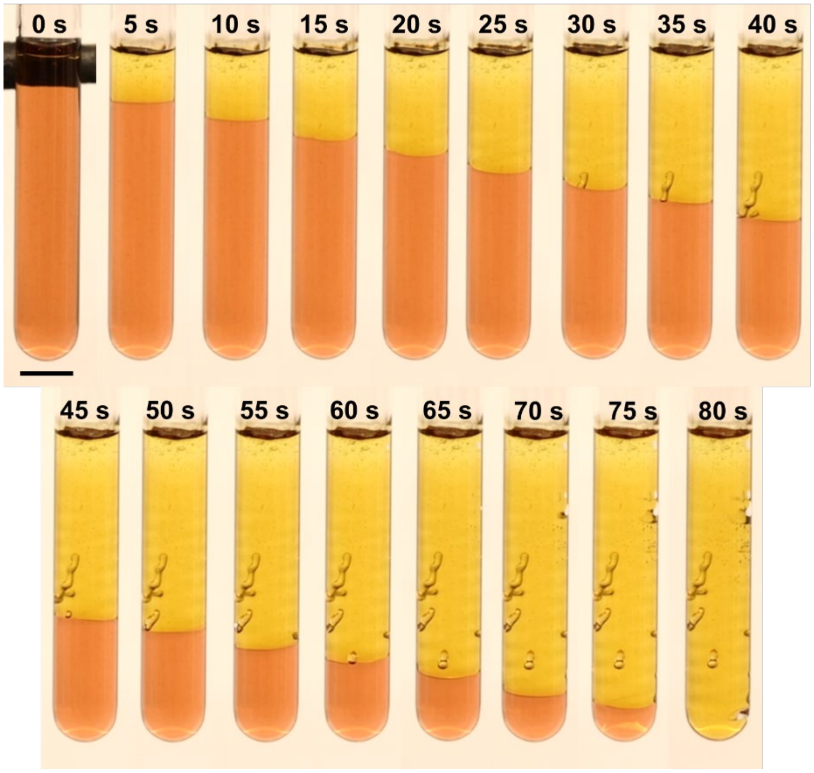

Figure S152: Representative timelapse of 1000:1:2 Monomer:G2:TBP (50:50 NBI_4:DCPD-H_2). The scale bar is 5 mm.

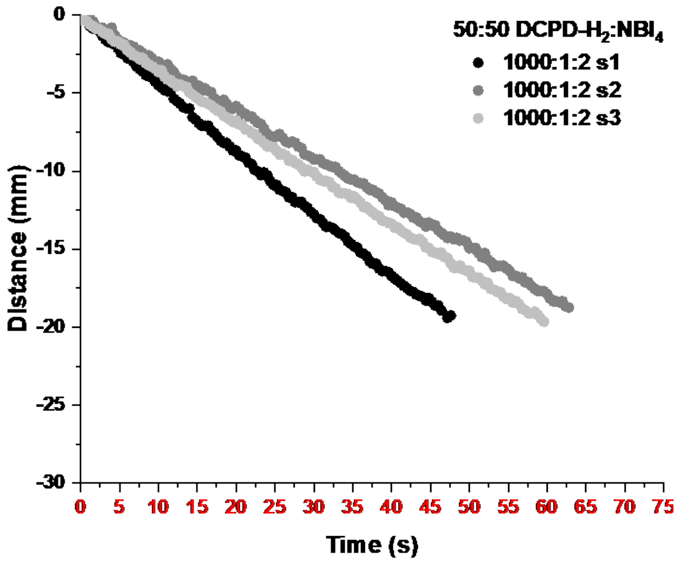

Figure S153: Front tracking of 1000:1:2 Monomer:G2:TBP (50:50 NBI_4:DCPD-H_2) in triplicate (v_f = 0.34 ± 0.05 mm/s).

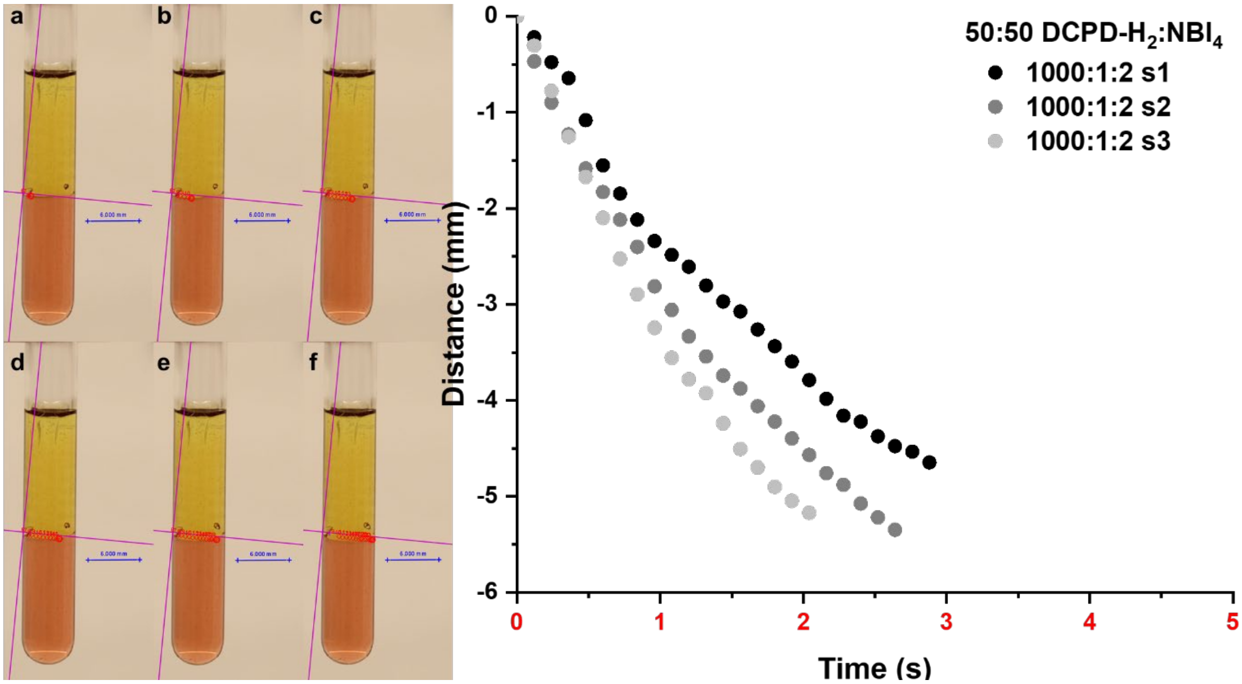

Figure S154: Representative timelapse of front tracking workflow and results for 1000:1:2 Monomer:G2:TBP (50:50 NBI_4:DCPD-H_2) patterned/across in triplicate ($v_{f,\,pattern}$ = 0.50 ± 0.09 mm/s).

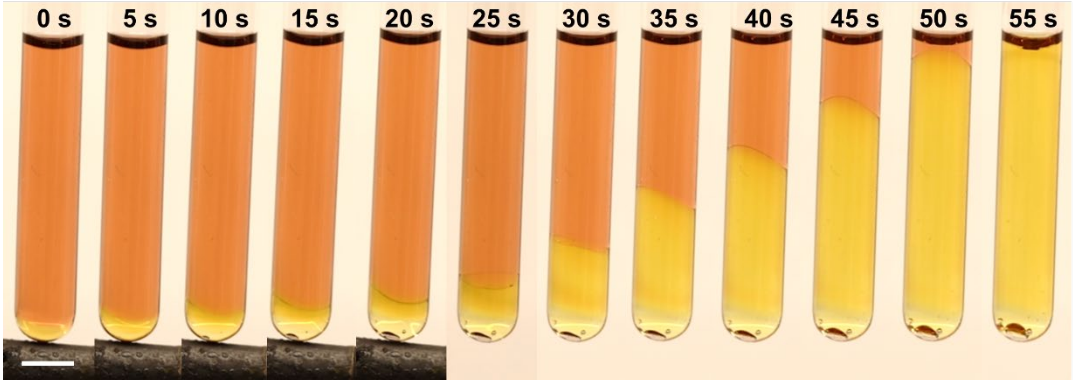

Figure S155: Representative timelapse of 1000:1:2 Monomer:G2:TBP **bottom up** (50:50 NBI_4:DCPD-H_2). The scale bar is 5 mm.

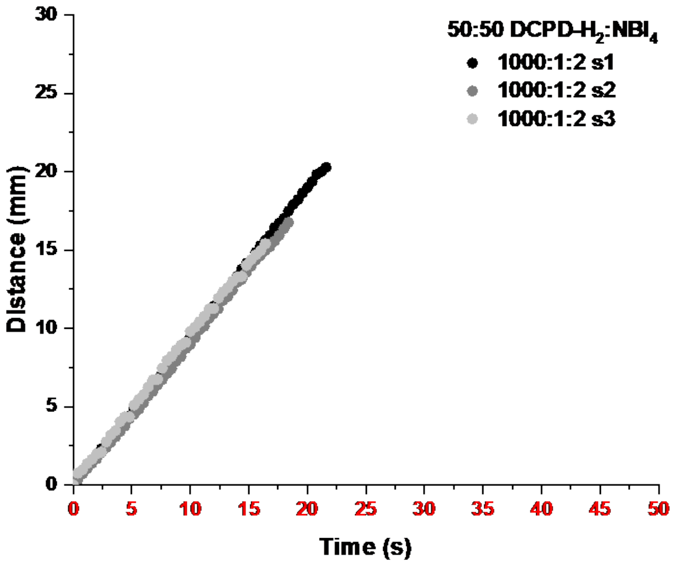

Figure S156: Front tracking of 1000:1:2 Monomer:G2:TBP (50:50 NBI_4:DCPD-H_2) **bottom up** in triplicate (v_f = 0.94 ± 0.01 mm/s).

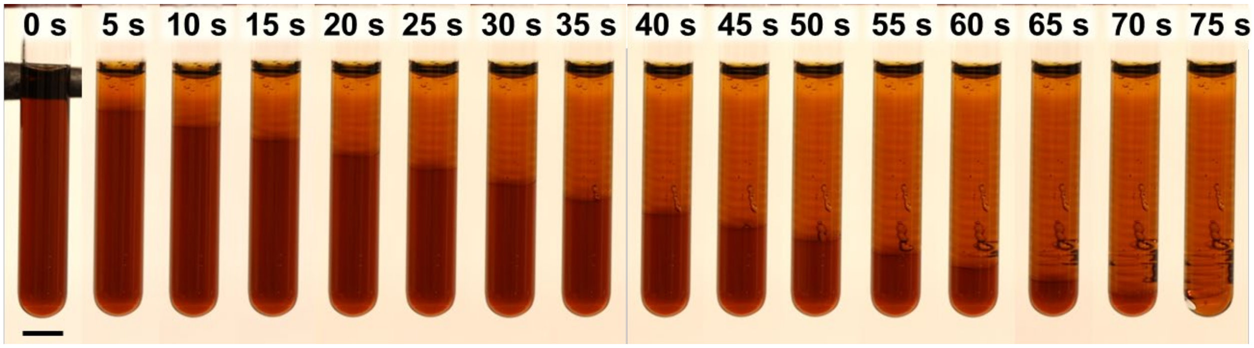

Figure S157: Representative timelapse of 200:1:2 Monomer:G2:TBP (50:50 NBI_4:DCPD-H_2). The scale bar is 5 mm.

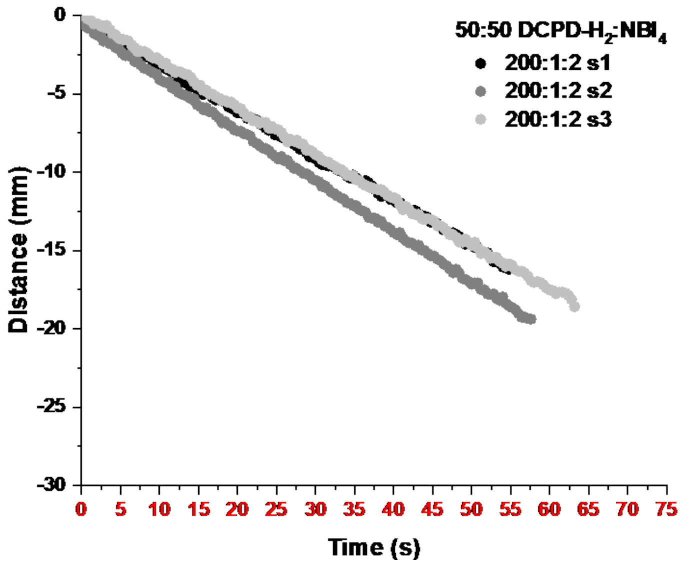

Figure S158: Front tracking of 200:1:2 Monomer:G2:TBP (50:50 NBI$_4$:DCPD-H$_2$) in triplicate (v_f = 0.30 ± 0.02 mm/s).

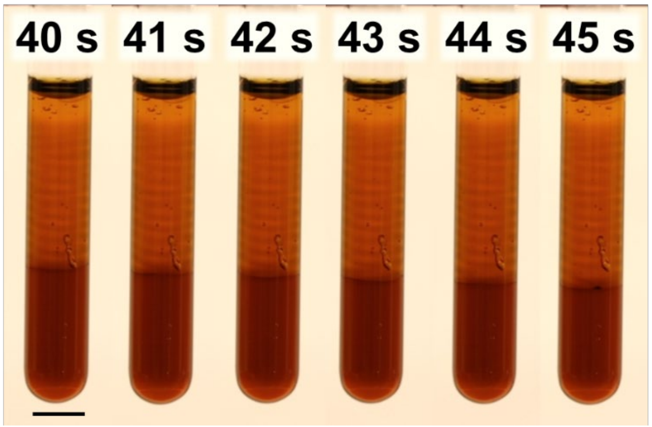

Figure S159: Representative timelapse of 200:1:2 Monomer:G2:TBP (50:50 NBI$_4$:DCPD-H$_2$) showing pattern progress across the tube in 1 second intervals. The scale bar is 5 mm.

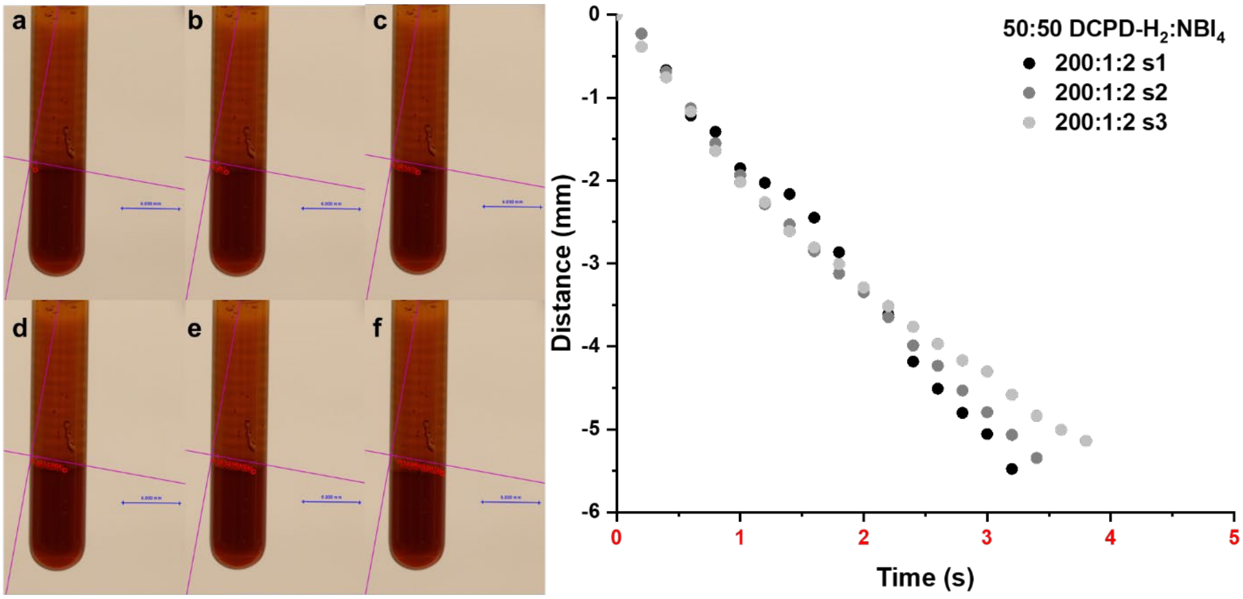

Figure S160: Representative timelapse of front tracking workflow and results for 200:1:2 Monomer:G2:TBP (50:50 NBI_4:DCPD-H_2) patterned/across in triplicate ($v_{f,\,pattern}$ = 0.66 ± 0.06 mm/s).

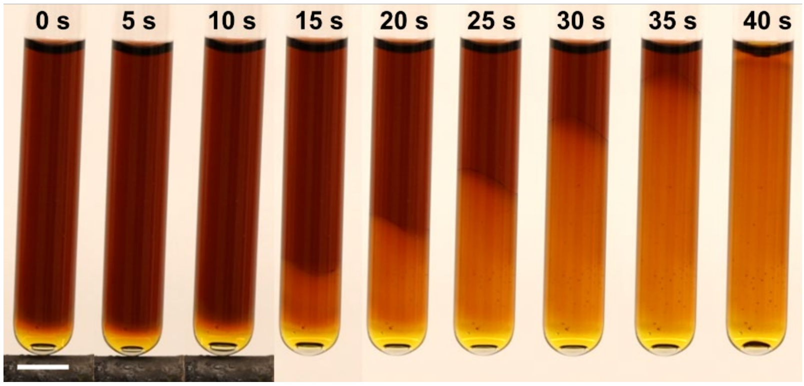

Figure S161: Representative timelapse of 200:1:2 Monomer:G2:TBP **bottom up** (50:50 NBI_4:DCPD-H_2). The scale bar is 5 mm.

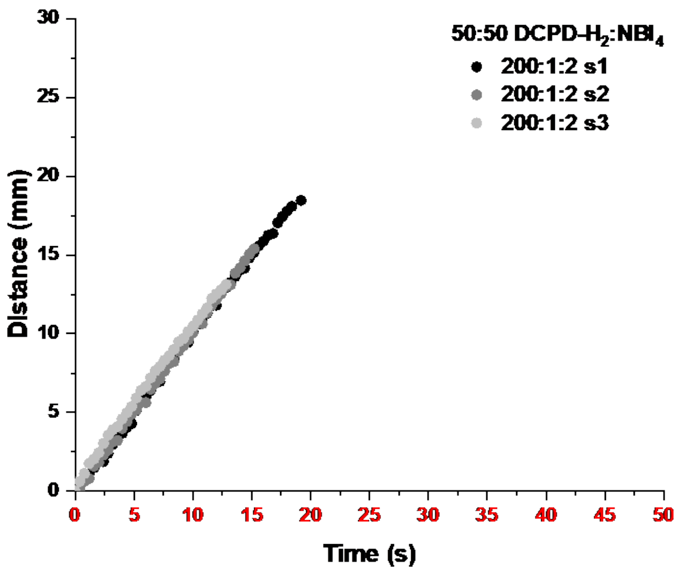

Figure S162: Front tracking of 200:1:2 Monomer:G2:TBP (50:50 NBI_4:DCPD-H_2) **bottom up** in triplicate (v_f = 1.01 ± 0.01 mm/s).

Table S7: Front velocity for copolymerizations at varying loadings.

Mixture	Monomer (equiv)	Initiator (equiv)	Inhibitor (equiv)	Direction	Front velocity (mm/s)	error
90:10	4000	1	1	downward	1.1	0.3
90:10	2000	1	1	downward	2.0	0.4
90:10	1000	1	1	downward	2.0	0.1
90:10	500	1	1	downward	2.4	0.1
90:10	200	1	1	downward	2.6	0.1
75:25	4000	1	1	downward	0.8	0.3
75:25	2000	1	1	downward	0.9	0.3
75:25	1000	1	1	downward	1.5	0.5
75:25	500	1	1	downward	1.5	0.4
75:25	200	1	1	downward	1.6	0.1
50:50	4000	1	1	downward	0.4	0.2
50:50	2000	1	1	downward	0.6	0.2
50:50	1000	1	1	downward	0.7	0.2
50:50	1000	1	1	upward	0.96	0.07
50:50	500	1	1	downward	0.7	0.1
50:50	200	1	1	downward	0.7	0.1
50:50	4000	1	**2**	downward	0.34	0.09
50:50	4000	1	**2**	upward	0.7	0.1
50:50	1000	1	**2**	downward	0.34	0.05
50:50	1000	1	**2**	upward	0.94	0.01
50:50	1000	1	**2**	patterned/across	0.50	0.09
50:50	200	1	**2**	downward	0.30	0.02
50:50	200	1	**2**	upward	1.01	0.01
50:50	200	1	**2**	patterned/across	0.66	0.06

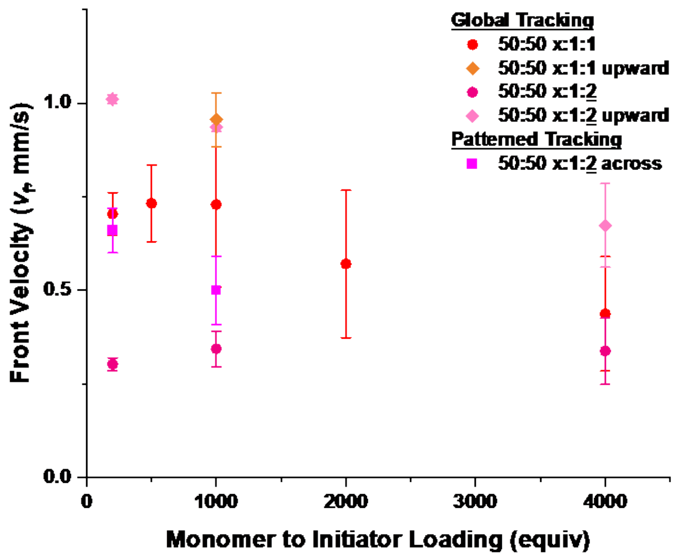

Figure S163: Front velocities for 50 mol% NBI_4 at varied loadings showing down the tube (with gravity), upward (against gravity), and across the tube when spin modes were present.

DSC Cure Kinetics:

<u>10 mol% NBI_4:</u>

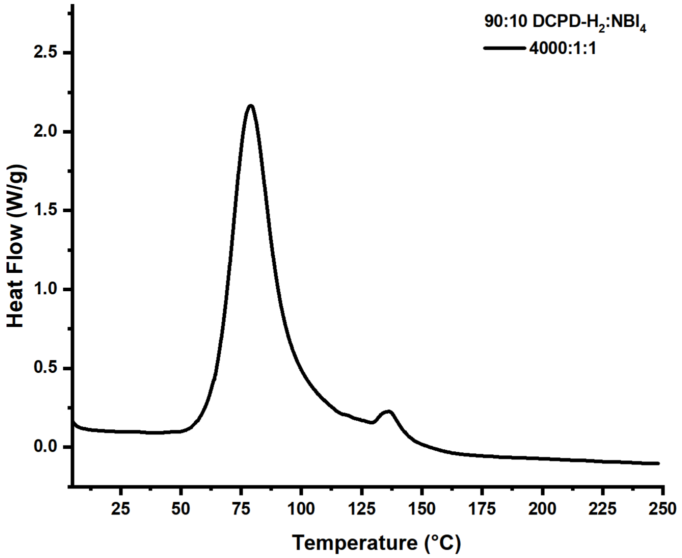

Figure S164: Representative DSC cure kinetic profile for 4000:1:1 Monomer:G2:TBP (10:90 NBI_4:DCPD-H_2, H_r = 356 ± 13 J/g, Peak Temp = 76 ± 2 °C). Exo up

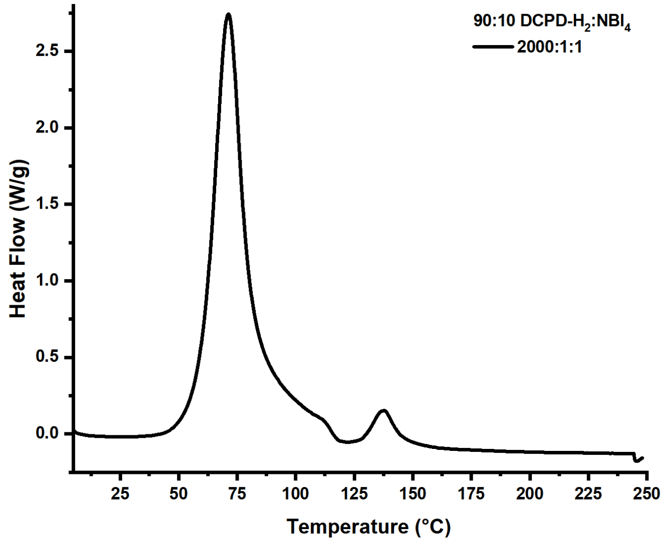

Figure S165: Representative DSC cure kinetic profile for 2000:1:1 Monomer:G2:TBP (10:90 NBI4:DCPD-H2, H_r = 338 ± 9 J/g, Peak Temp = 72 ± 1 °C). Exo up

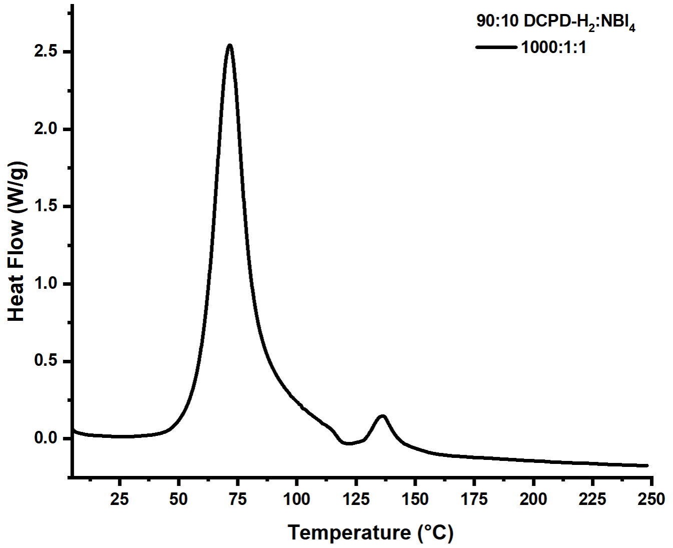

Figure S166: Representative DSC cure kinetic profile for 1000:1:1 Monomer:G2:TBP (10:90 NBI_4:DCPD-H_2, H_r = 345 ± 3 J/g, Peak Temp = 72 ± 1 °C). Exo up

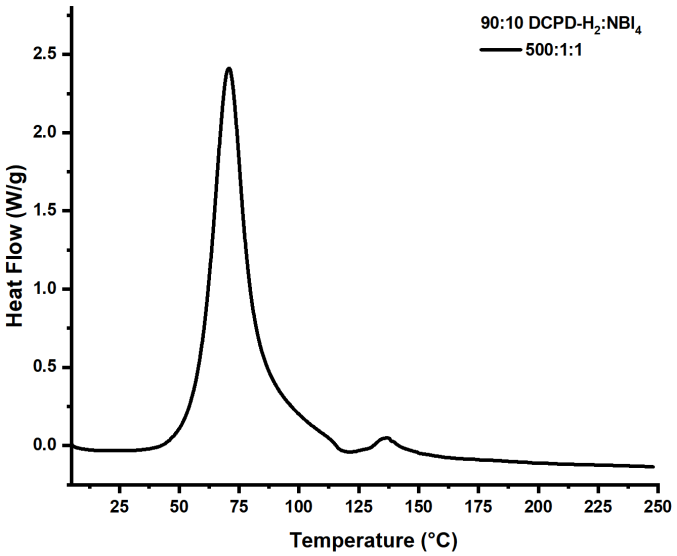

Figure S167: Representative DSC cure kinetic profile for 500:1:1 Monomer:G2:TBP (10:90 NBI_4:DCPD-H_2, H_r = 318 ± 5 J/g, Peak Temp = 71 ± 1 °C). Exo up

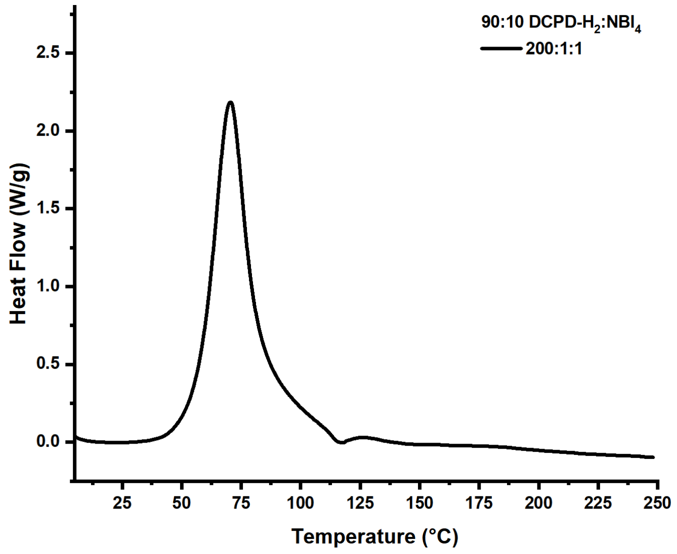

Figure S168: Representative DSC cure kinetic profile for 200:1:1 Monomer:G2:TBP (10:90 NBI_4:DCPD-H_2, H_r = 293 ± 9 J/g, Peak Temp = 70 ± 1 °C). Exo up

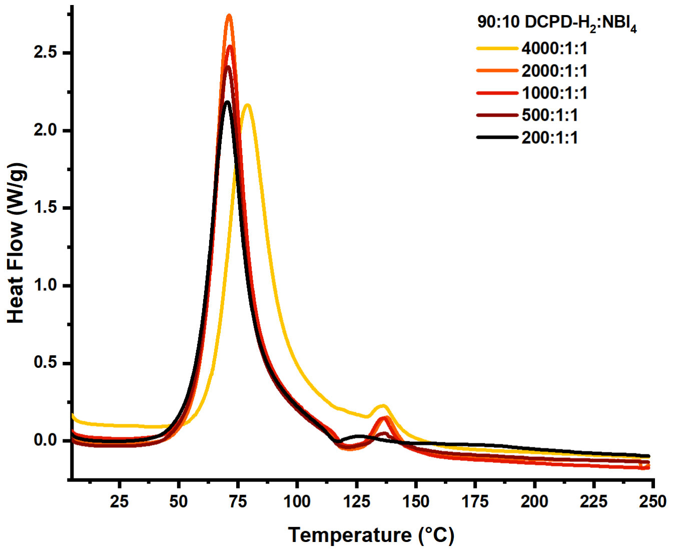

Figure S169: Stacked representative DSC cure kinetic profiles for x:1:1 Monomer:G2:TBP (10:90 NBI_4:DCPD-H_2). Exo up

25 mol% NBI_4:

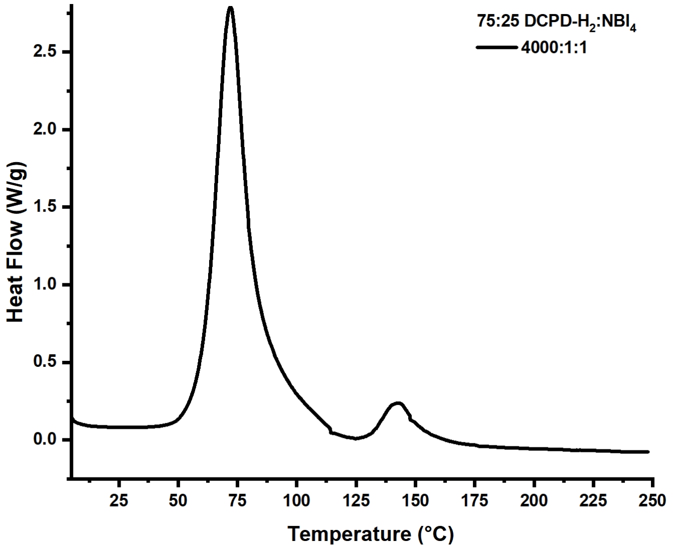

Figure S170: Representative DSC cure kinetic profile for 4000:1:1 Monomer:G2:TBP (25:75 NBI_4:DCPD-H_2, H_r = 335 ± 14 J/g, Peak Temp = 75 ± 3 °C). Exo up

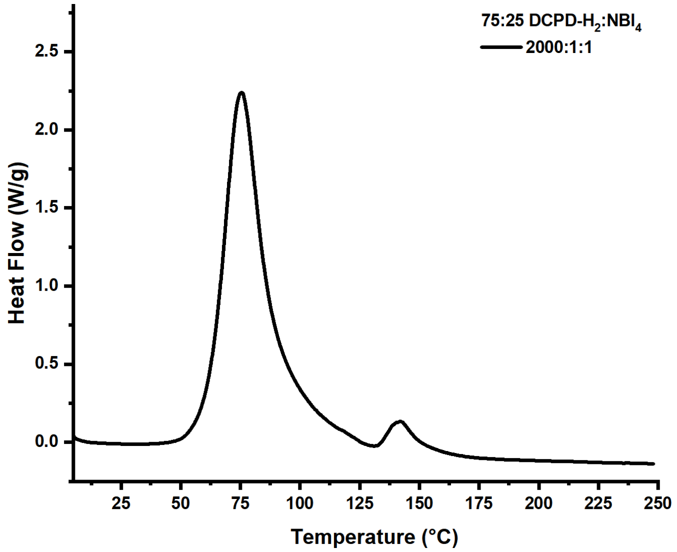

Figure S171: Representative DSC cure kinetic profile for 2000:1:1 Monomer:G2:TBP (25:75 NBI_4:DCPD-H_2, H_r = 340 ± 7 J/g, Peak Temp = 74 ± 2 °C). Exo up

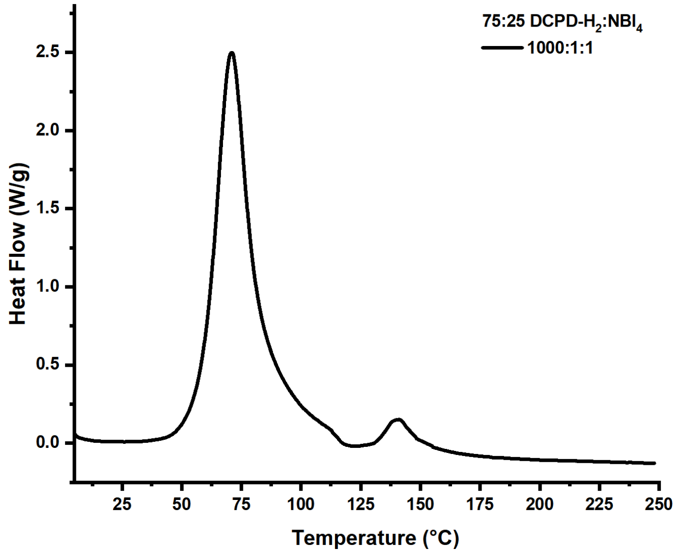

Figure S172: Representative DSC cure kinetic profile for 1000:1:1 Monomer:G2:TBP (25:75 NBI_4:DCPD-H_2, H_r = 327 ± 4 J/g, Peak Temp = 71 ± 2 °C). Exo up

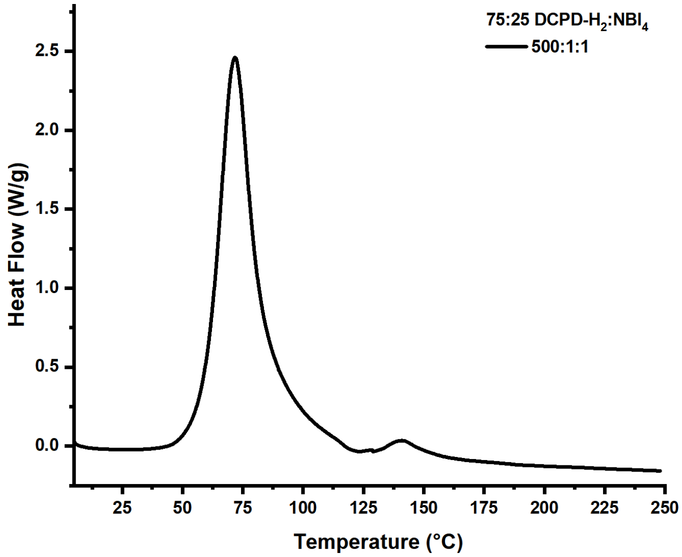

Figure S173: Representative DSC cure kinetic profile for 500:1:1 Monomer:G2:TBP (25:75 NBI_4:DCPD-H_2, H_r = 327 ± 8 J/g, Peak Temp = 71 ± 2 °C). Exo up

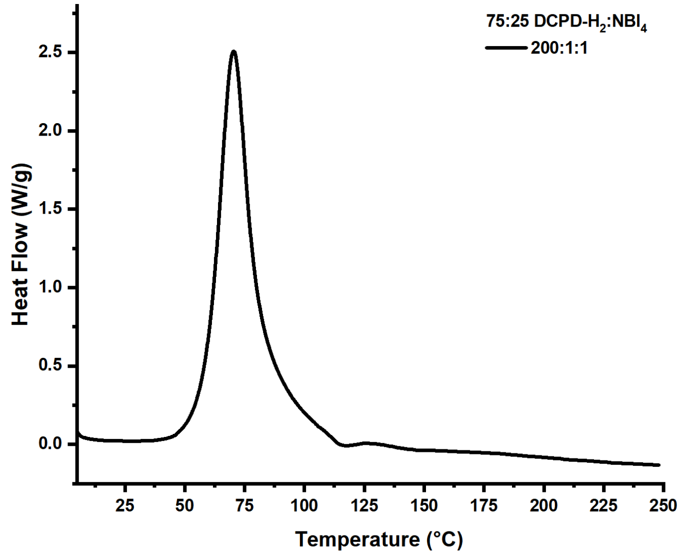

Figure S174: Representative DSC cure kinetic profile for 200:1:1 Monomer:G2:TBP (25:75 NBI_4:DCPD-H_2, H_r = 288 ± 17 J/g, Peak Temp = 70 ± 1 °C). Exo up

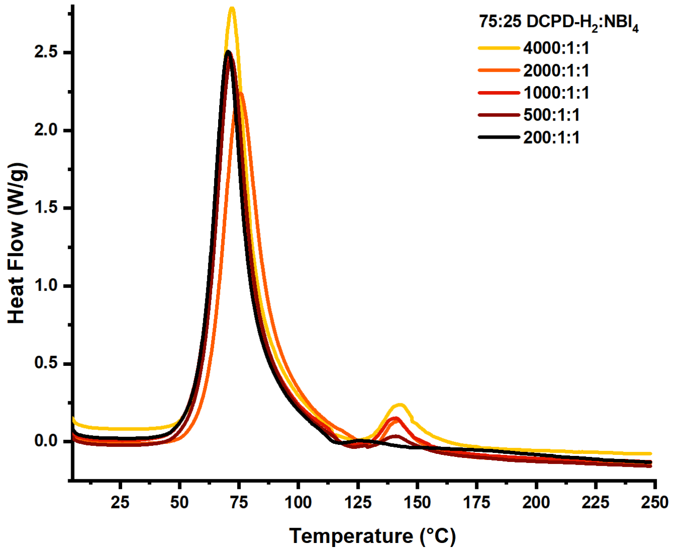

Figure S175: Stacked representative DSC cure kinetic profiles for x:1:1 Monomer:G2:TBP (25:75 NBI_4:DCPD-H_2). Exo up

50 mol% NBI_4:

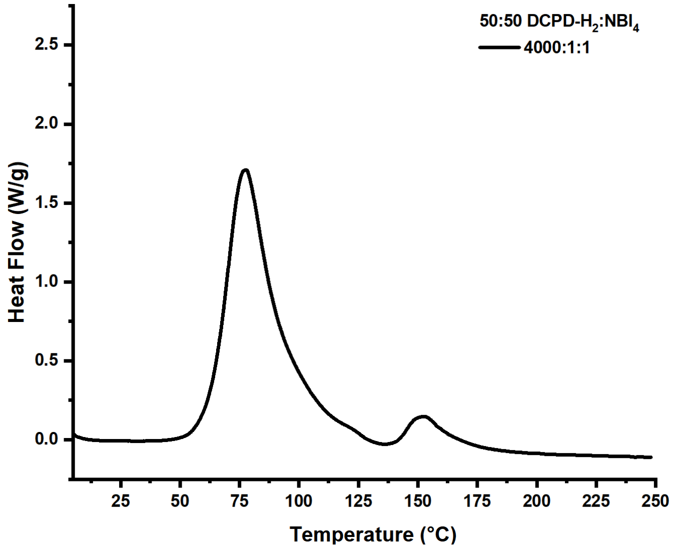

Figure S176: Representative DSC cure kinetic profile for 4000:1:1 Monomer:G2:TBP (50:50 NBI_4:DCPD-H_2, H_r = 292 ± 13 J/g, Peak Temp = 75 ± 2 °C). Exo up

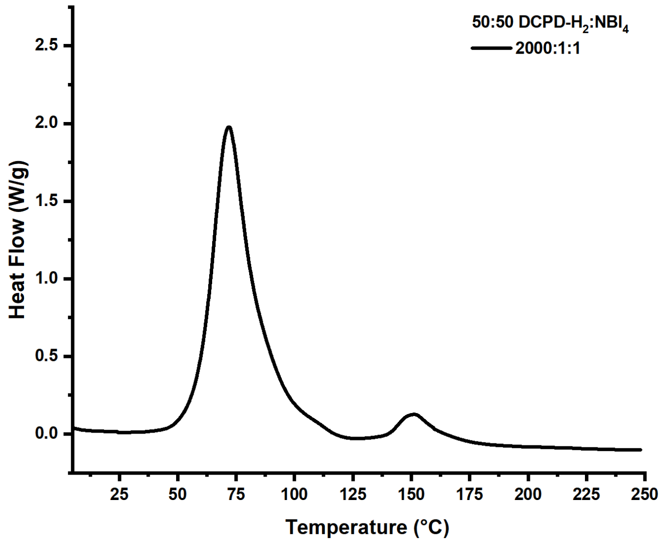

Figure S177: Representative DSC cure kinetic profile for 2000:1:1 Monomer:G2:TBP (50:50 NBI_4:DCPD-H_2, H_r = 281 ± 9 J/g, Peak Temp = 74 ± 2 °C). Exo up

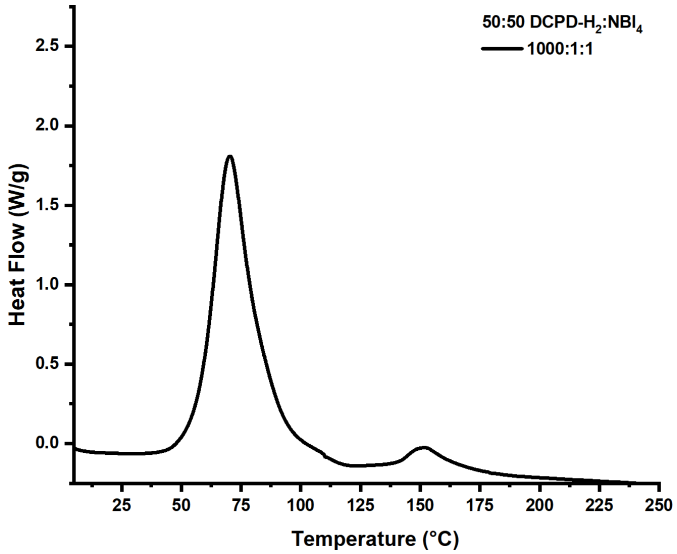

Figure S178: Representative DSC cure kinetic profile for 1000:1:1 Monomer:G2:TBP (50:50 NBI_4:DCPD-H_2, H_r = 273 ± 6 J/g, Peak Temp = 72 ± 2 °C). Exo up

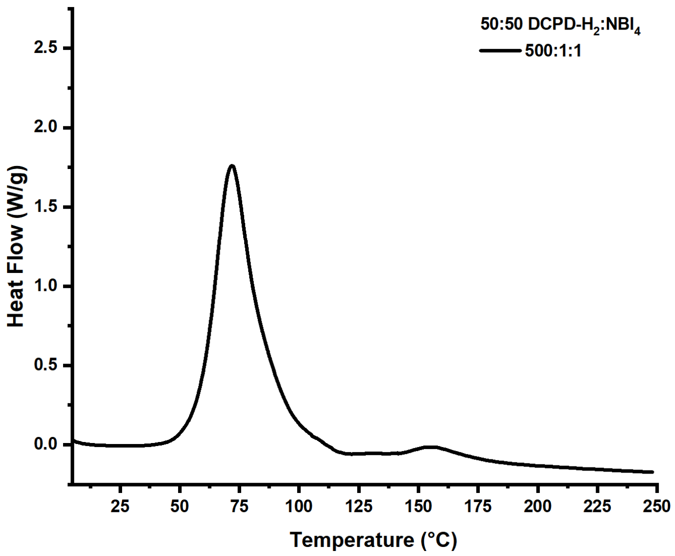

Figure S179: Representative DSC cure kinetic profile for 500:1:1 Monomer:G2:TBP (50:50 NBI_4:DCPD-H_2, H_r = 254 ± 16 J/g, Peak Temp = 72 ± 1 °C). Exo up

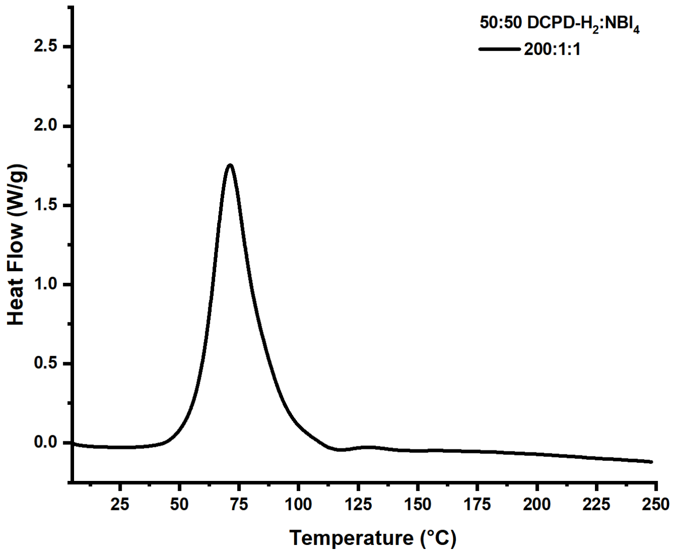

Figure S180: Representative DSC cure kinetic profile for 200:1:1 Monomer:G2:TBP (50:50 NBI_4:DCPD-H_2, H_r = 243 ± 6 J/g, Peak Temp = 72 ± 1 °C). Exo up

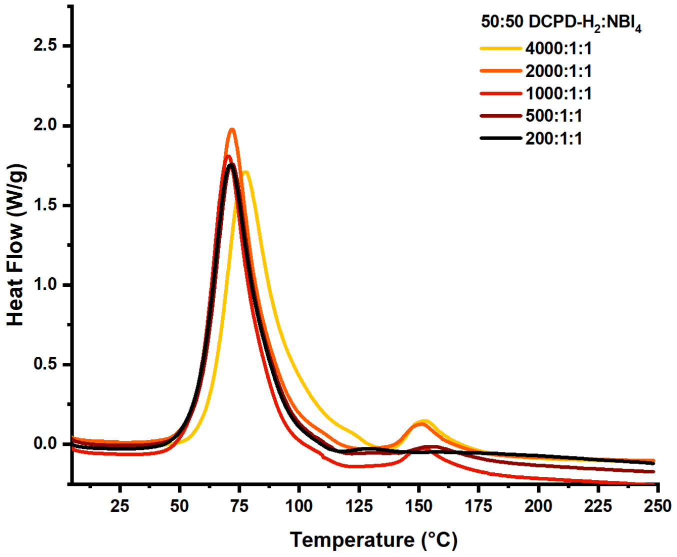

Figure S181: Stacked representative DSC cure kinetic profiles for x:1:1 Monomer:G2:TBP (50:50 NBI_4:DCPD-H_2). Exo up

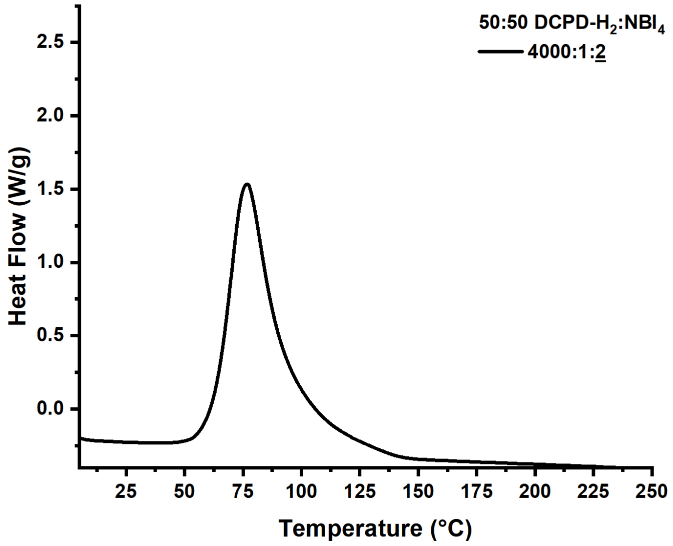

Figure S182: Representative DSC cure kinetic profile for 4000:1:2 Monomer:G2:TBP (50:50 NBI_4:DCPD-H_2, H_r = 298 ± 9 J/g, Peak Temp = 76 ± 1 °C). Exo up

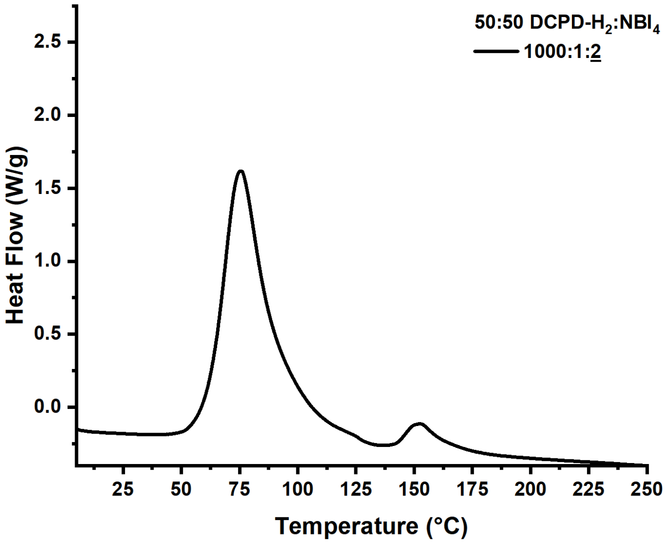

Figure S183: Representative DSC cure kinetic profile for 1000:1:2 Monomer:G2:TBP (50:50 NBI_4:DCPD-H_2, H_r = 293 ± 23 J/g, Peak Temp = 76 ± 1 °C). Exo up

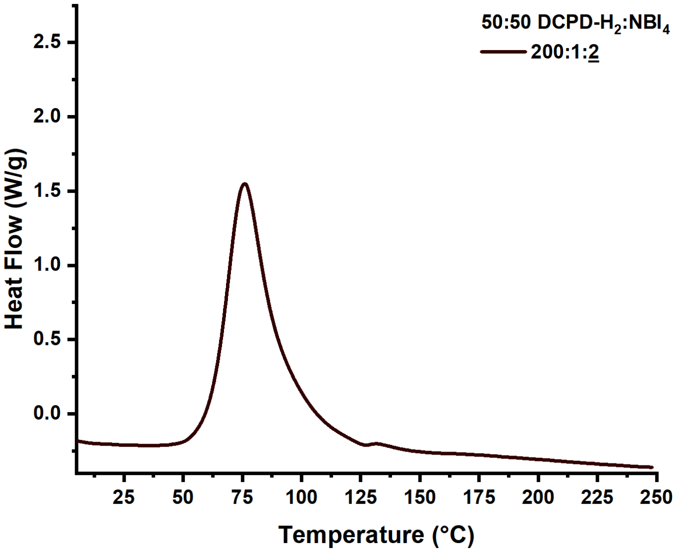

Figure S184: Representative DSC cure kinetic profile for 200:1:2 Monomer:G2:TBP (50:50 NBI_4:DCPD-H_2, H_r = 263 ± 15 J/g, Peak Temp = 76 ± 1 °C). Exo up

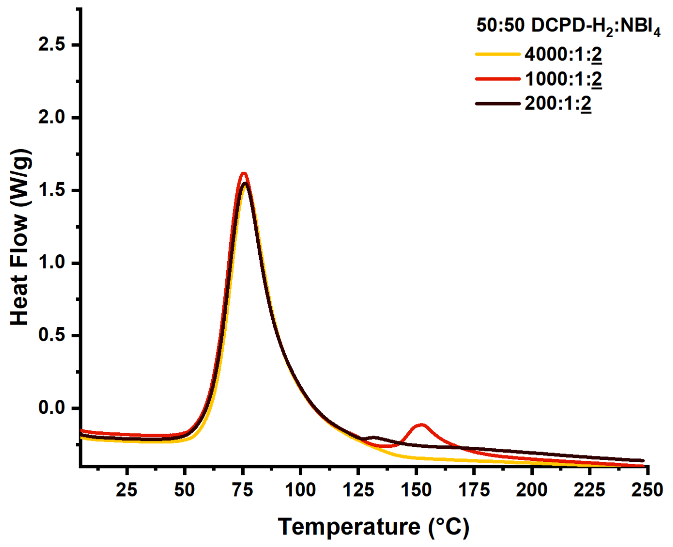

Figure S185: Stacked representative DSC cure kinetic profiles for x:1:2 Monomer:G2:TBP (50:50 NBI_4:DCPD-H_2). Exo up

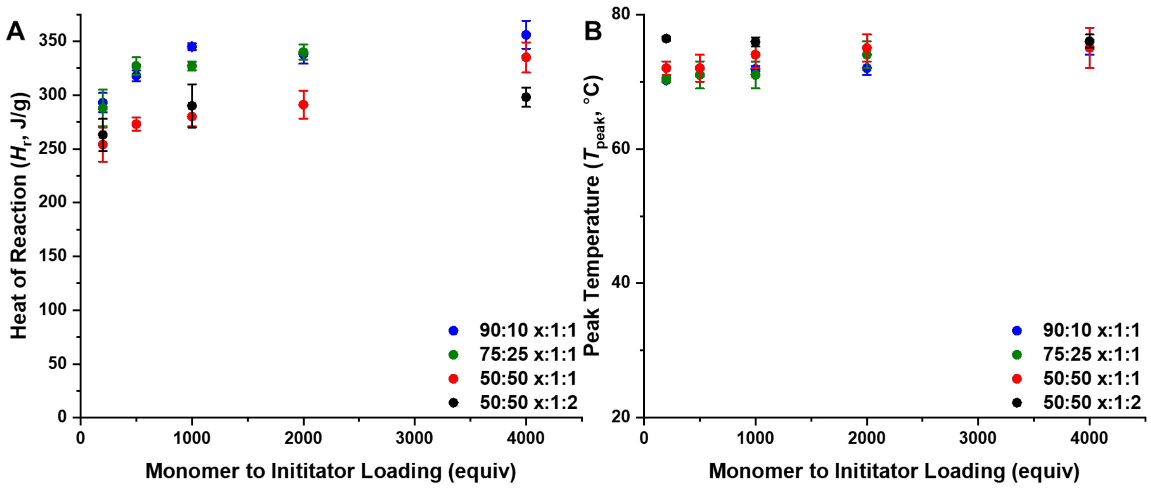

Figure S186: (A) Heat of reaction (H_r) and (B) temperature of peak maximum (T_{peak}) showing retention of H_r and increasing T_{peak} with increasing inhibitor loading.

Table S8: Heat of reaction (H_r) and peak temperature (T_{peak}) for copolymerization resins at varied loadings.

Mixture	Monomer (equiv)	Initiator (equiv)	Inhibitor (equiv)	H_r (J/g)	error	T_{peak} (°C)	error
90:10	4000	1	1	356	13	76	2
90:10	2000	1	1	338	9	72	1
90:10	1000	1	1	345	3	72	1
90:10	500	1	1	318	5	71	1
90:10	200	1	1	293	9	7	1
75:25	4000	1	1	335	14	75	3
75:25	2000	1	1	340	7	74	2
75:25	1000	1	1	327	4	71	2
75:25	500	1	1	327	8	71	2
75:25	200	1	1	288	17	70	1
50:50	4000	1	1	292	13	75	3
50:50	2000	1	1	281	9	74	2
50:50	1000	1	1	273	6	74	2
50:50	500	1	1	254	6	72	1
50:50	200	1	1	243	6	72	1
50:50	4000	1	**2**	298	9	76	1
50:50	1000	1	**2**	293	23	76	1
50:50	200	1	**2**	263	15	76	1

Sample Images:

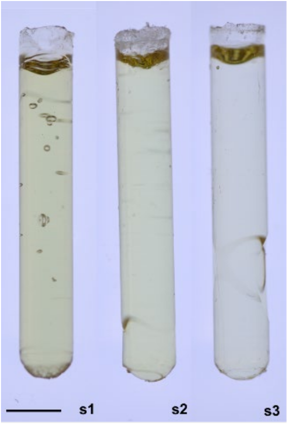

Figure S187: Image of triplicate 10 mol% NBI_4 samples at 4000:1:1 post-FROMP. The scale bar is 5 mm.

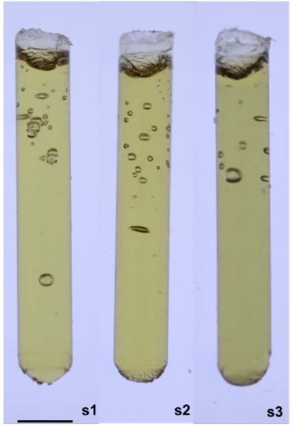

Figure S188: Image of triplicate 10 mol% NBI_4 samples at 2000:1:1 post-FROMP. The scale bar is 5 mm.

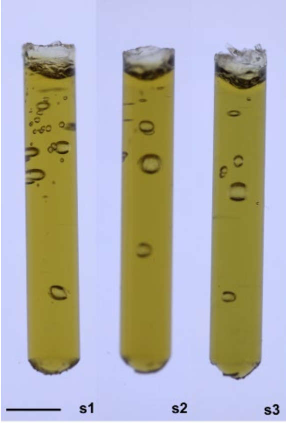

Figure S189: Image of triplicate 10 mol% NBI_4 samples at 1000:1:1 post-FROMP. The scale bar is 5 mm.

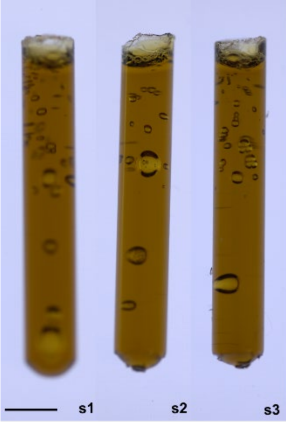

Figure S190: Image of triplicate 10 mol% NBI_4 samples at 500:1:1 post-FROMP. The scale bar is 5 mm.

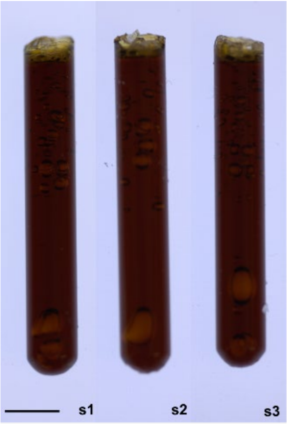

Figure S191: Image of triplicate 10 mol% NBI_4 samples at 200:1:1 post-FROMP. The scale bar is 5 mm.

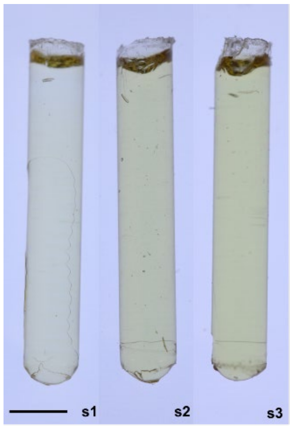

Figure S192: Image of triplicate 25 mol% NBI_4 samples at 4000:1:1 post-FROMP. The scale bar is 5 mm.

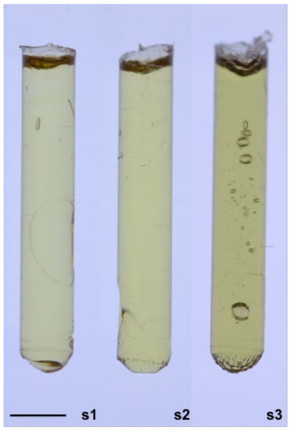

Figure S193: Image of triplicate 25 mol% NBI_4 samples at 2000:1:1 post-FROMP. The scale bar is 5 mm.

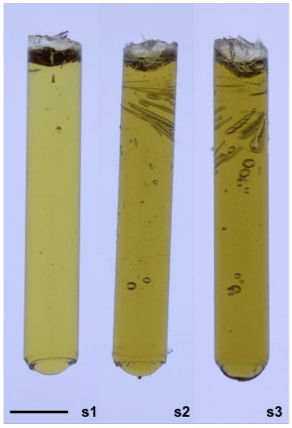

Figure S194: Image of triplicate 25 mol% NBI_4 samples at 1000:1:1 post-FROMP. The scale bar is 5 mm.

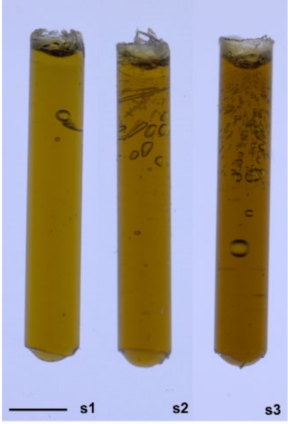

Figure S195: Image of triplicate 25 mol% NBI_4 samples at 500:1:1 post-FROMP. The scale bar is 5 mm.

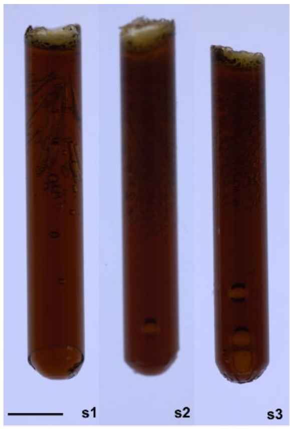

Figure S196: Image of triplicate 25 mol% NBI_4 samples at 200:1:1 post-FROMP. The scale bar is 5 mm.

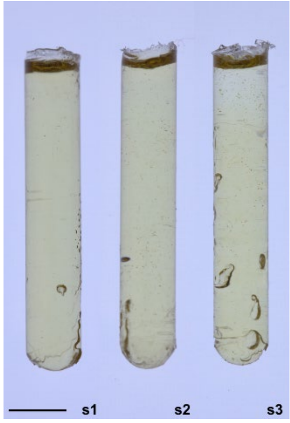

Figure S197: Image of triplicate 50 mol% NBI_4 samples at 4000:1:1 post-FROMP. The scale bar is 5 mm.

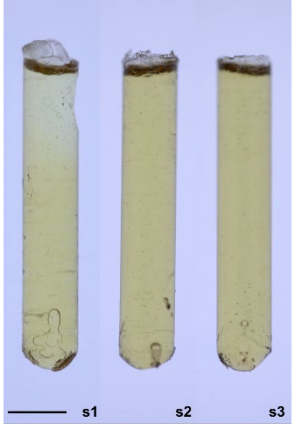

Figure S198: Image of triplicate 50 mol% NBI_4 samples at 2000:1:1 post-FROMP. The scale bar is 5 mm.

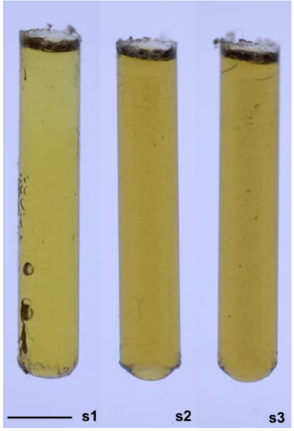

Figure S199: Image of triplicate 50 mol% NBI_4 samples at 1000:1:1 post-FROMP. The scale bar is 5 mm.

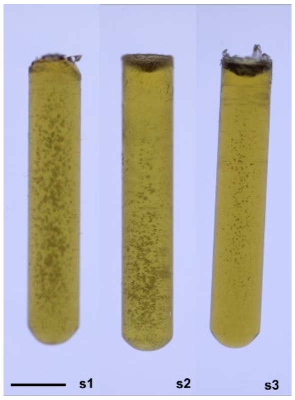

Figure S200: Image of triplicate 50 mol% NBI_4 samples at 1000:1:1 **bottom up** post-FROMP. The scale bar is 5 mm.

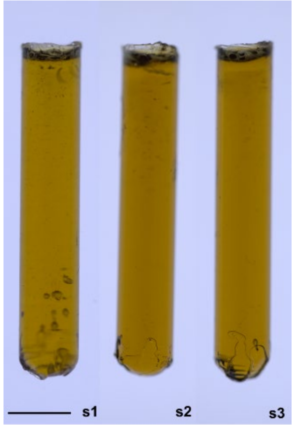

Figure S201: Image of triplicate 50 mol% NBI_4 samples at 500:1:1 post-FROMP. The scale bar is 5 mm.

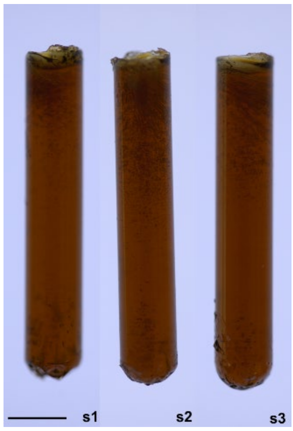

Figure S202: Image of triplicate 50 mol% NBI_4 samples at 200:1:1 post-FROMP. The scale bar is 5 mm.

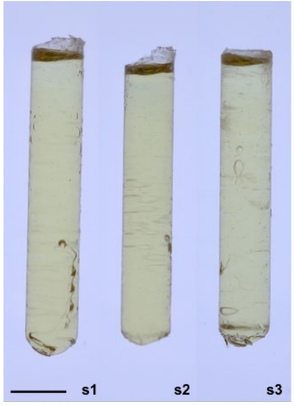

Figure S203: Image of triplicate 50 mol% NBI_4 samples at 4000:1:2 post-FROMP. The scale bar is 5 mm.

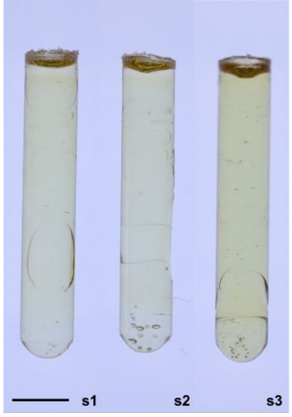

Figure S204: Image of triplicate 50 mol% NBI_4 samples at 4000:1:2 **bottom up** post-FROMP. The scale bar is 5 mm.

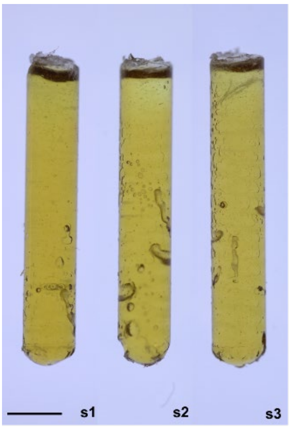

Figure S205: Image of triplicate 50 mol% NBI_4 samples at 1000:1:2 post-FROMP. The scale bar is 5 mm.

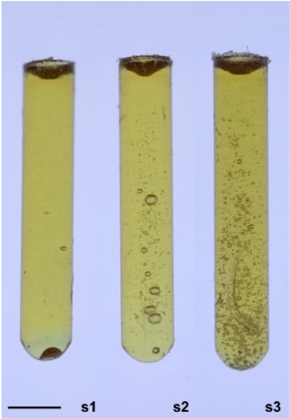

Figure S206: Image of triplicate 50 mol% NBI_4 samples at 1000:1:2 **bottom up** post-FROMP. The scale bar is 5 mm.

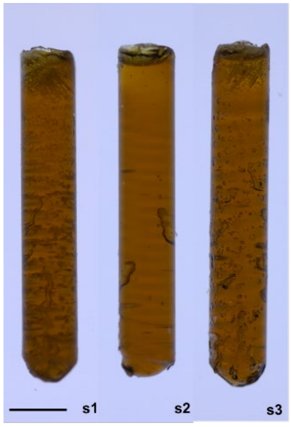

Figure S207: Image of triplicate 50 mol% NBI_4 samples at 200:1:2 post-FROMP. The scale bar is 5 mm.

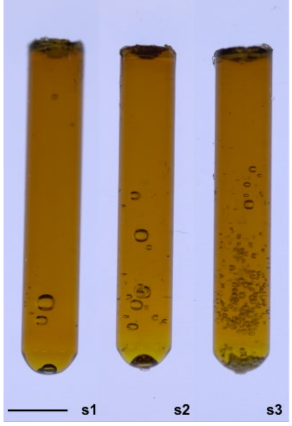

Figure S208: Image of triplicate 50 mol% NBI_4 samples at 200:1:2 **bottom up** post-FROMP. The scale bar is 5 mm.

Size Exclusion Chromatography (SEC):

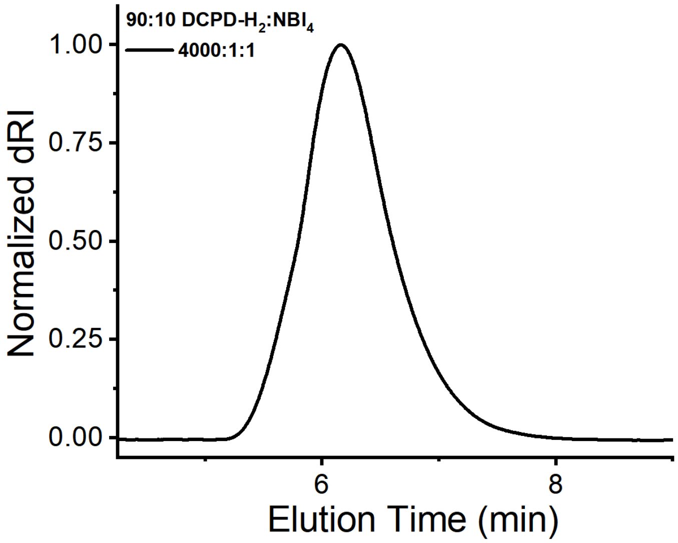

Figure S209: Representative SEC of 10 mol% NBI_4 post-FROMP for 4000:1:1 (M_n = 440 ± 40 kg/mol, $Đ$ = 1.71 ± 0.02).

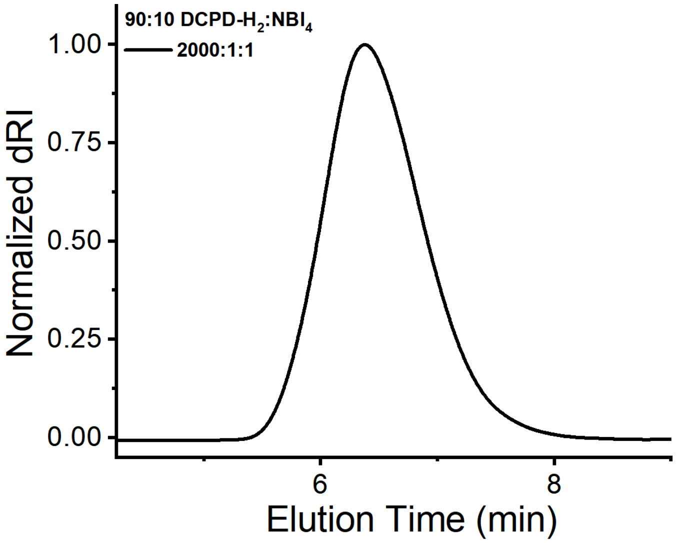

Figure S210: Representative SEC of 10 mol% NBI_4 post-FROMP for 2000:1:1 (M_n = 260 ± 70 kg/mol, $Đ$ = 1.67 ± 0.2).

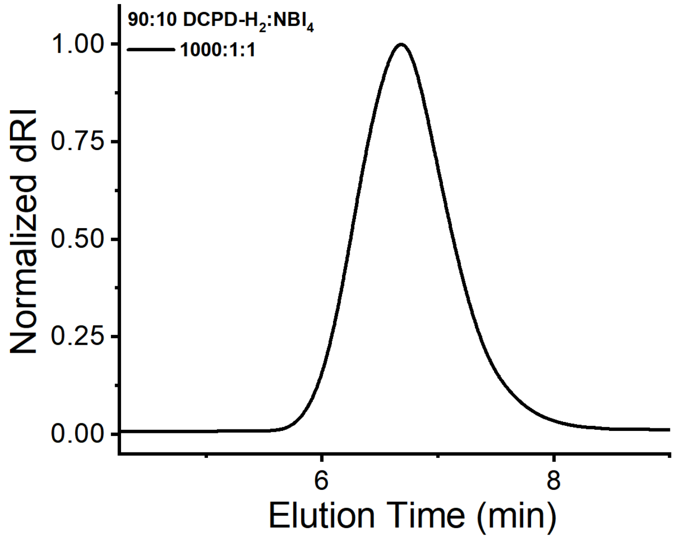

Figure S211: Representative SEC of 10 mol% NBI_4 post-FROMP for 1000:1:1 (M_n = 160 ± 20 kg/mol, $Đ$ = 1.69 ± 0.09).

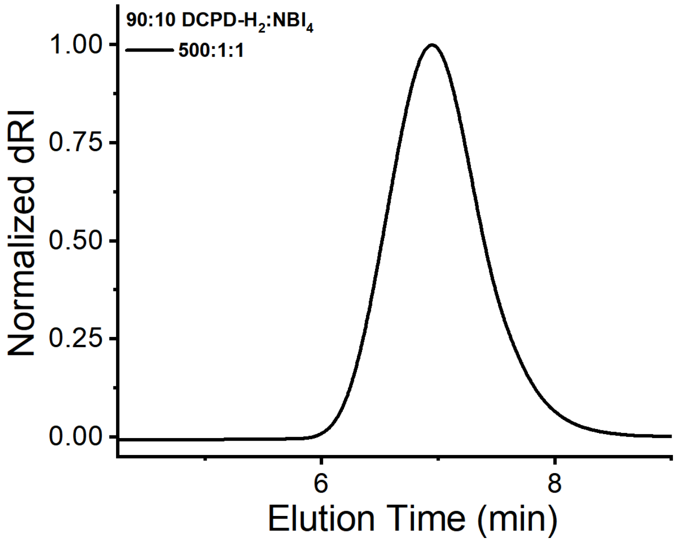

Figure S212: Representative SEC of 10 mol% NBI_4 post-FROMP for 500:1:1 (M_n = 93 ± 3 kg/mol, $Đ$ = 1.58 ± 0.07).

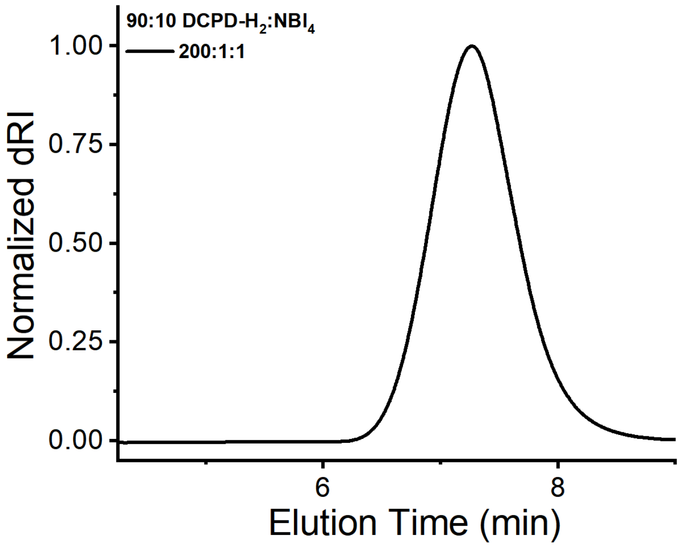

Figure S213: Representative SEC of 10 mol% NBI_4 post-FROMP for 200:1:1 (M_n = 51 ± 5 kg/mol, $Đ$ = 1.52 ± 0.08.

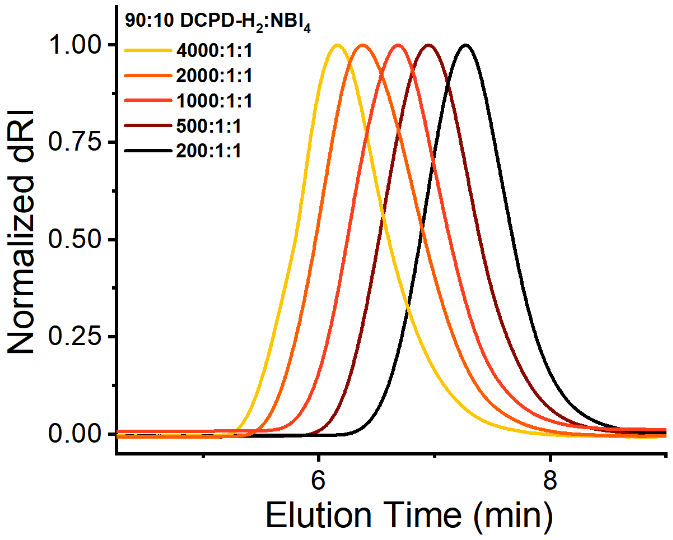

Figure S214: Representative SEC overlay of 10 mol% NBI_4 post-FROMP for varied loadings (x:1:1 monomer:initiator:inhibitor).

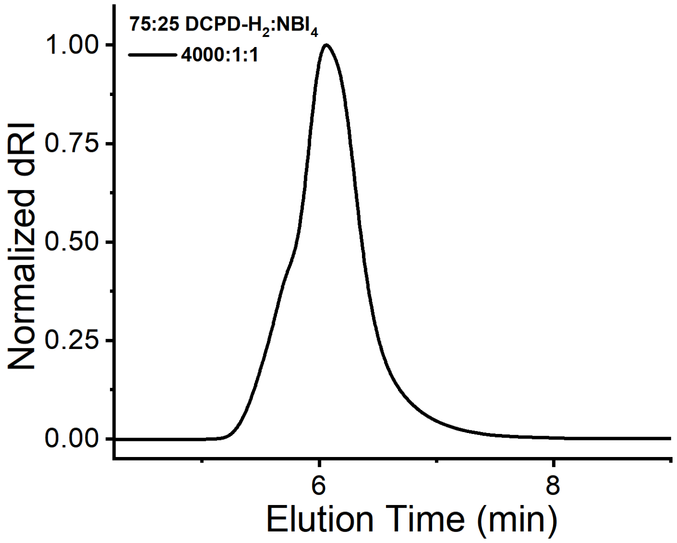

Figure S215: Representative SEC of 25 mol% NBI_4 post-FROMP for 4000:1:1 (M_n = 690 ± 90 kg/mol, $Đ$ = 1.45 ± 0.09).

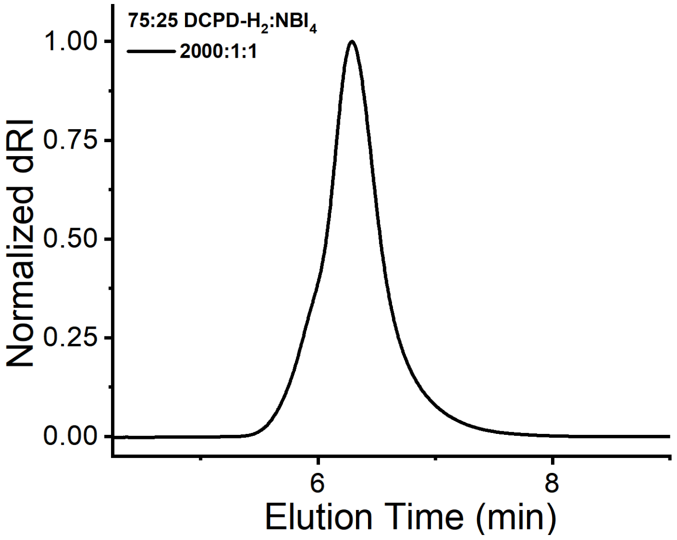

Figure S216: Representative SEC of 25 mol% NBI_4 post-FROMP for 2000:1:1 (M_n = 410 ± 20 kg/mol, $Đ$ = 1.36 ± 0.12).

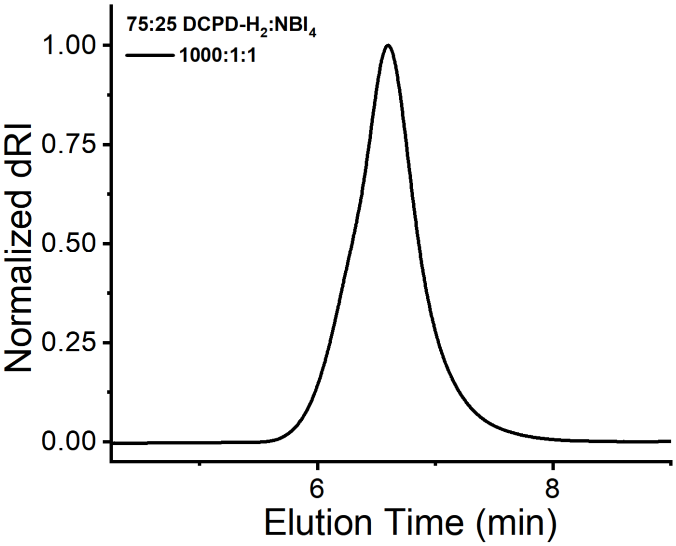

Figure S217: Representative SEC of 25 mol% NBI_4 post-FROMP for 1000:1:1 (M_n = 210 ± 10 kg/mol, $Đ$ = 1.46 ± 0.05).

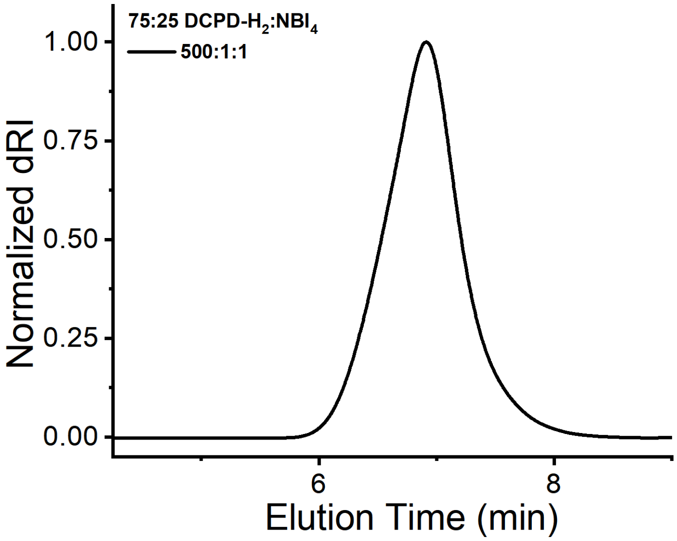

Figure S218: Representative SEC of 25 mol% NBI_4 post-FROMP for 500:1:1 (M_n = 130 ± 10 kg/mol, $Đ$ = 1.37 ± 0.06).

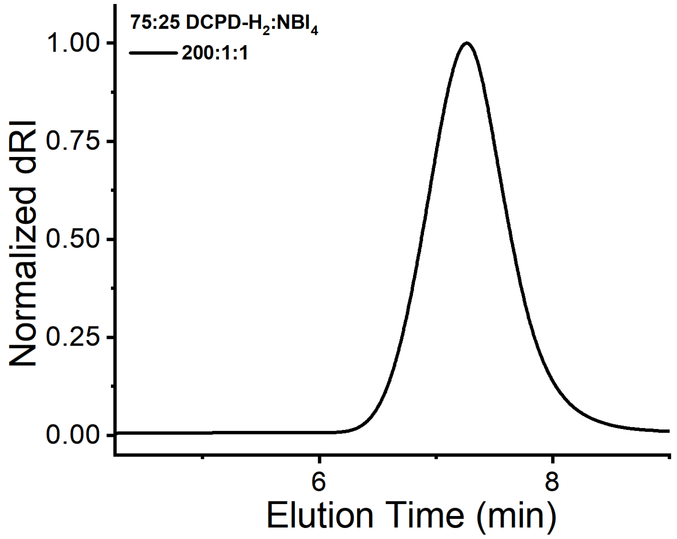

Figure S219: Representative SEC of 25 mol% NBI_4 post-FROMP for 200:1:1 (M_n = 60 ± 7 kg/mol, $Đ$ = 1.40 ± 0.11).

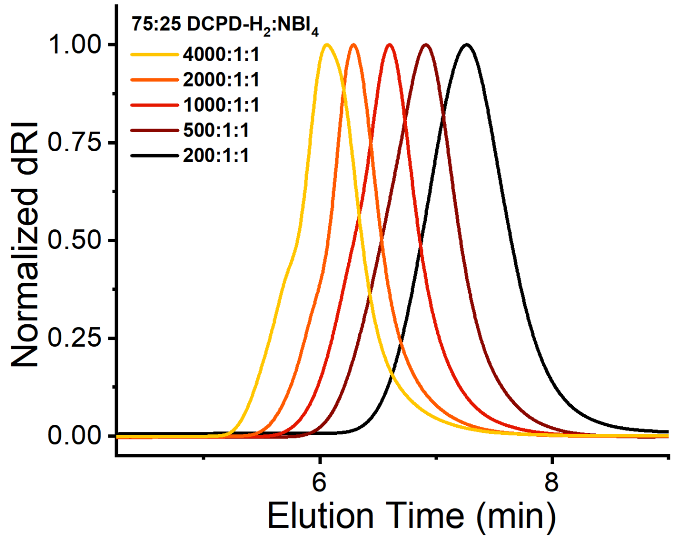

Figure S220: Representative SEC overlay of 25 mol% NBI_4 post-FROMP for varied loadings (x:1:1 monomer:initiator:inhibitor).

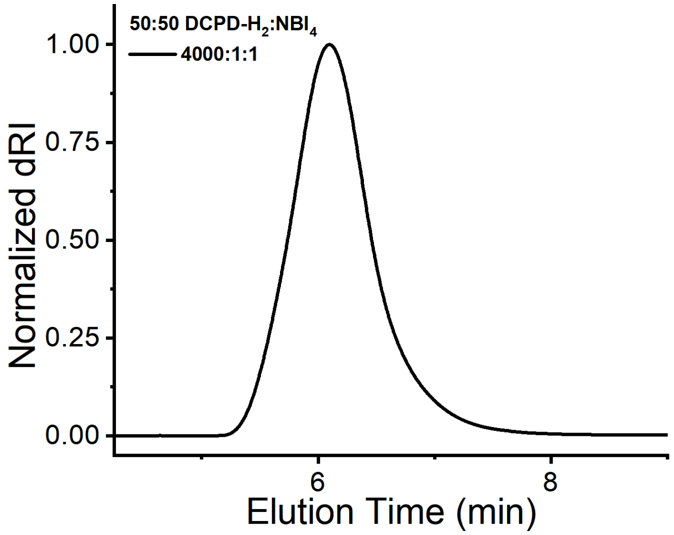

Figure S221: Representative SEC of 50 mol% NBI_4 post-FROMP for 4000:1:1 (M_n = 700 ± 40 kg/mol, $Đ$ = 1.33 ± 0.05).

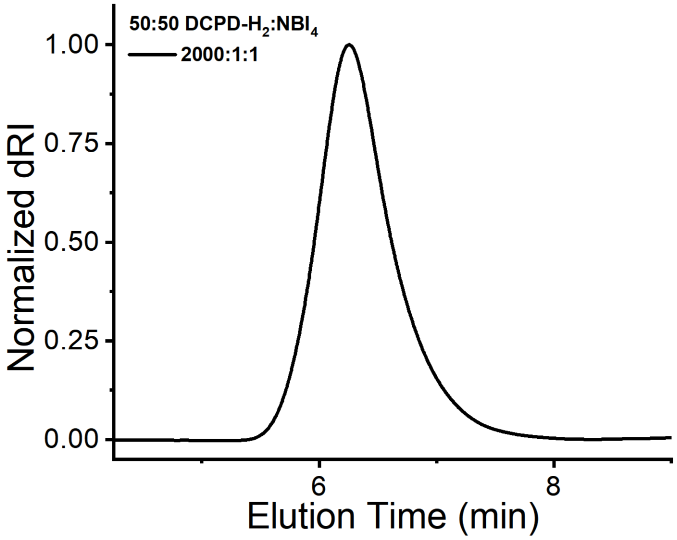

Figure S222: Representative SEC of 50 mol% NBI_4 post-FROMP for 2000:1:1 (M_n = 410 ± 70 kg/mol, $Đ$ = 1.36 ± 0.14).

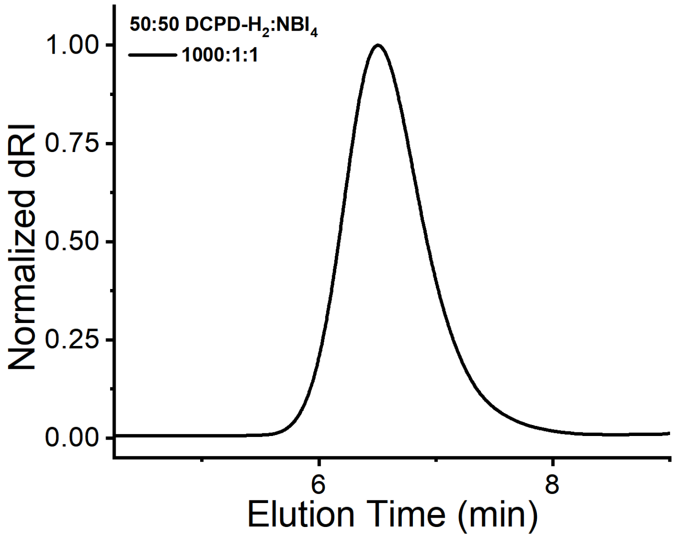

Figure S223: Representative SEC of 50 mol% NBI_4 post-FROMP for 1000:1:1 (M_n = 240 ± 20 kg/mol, $Đ$ = 1.37 ± 0.15).

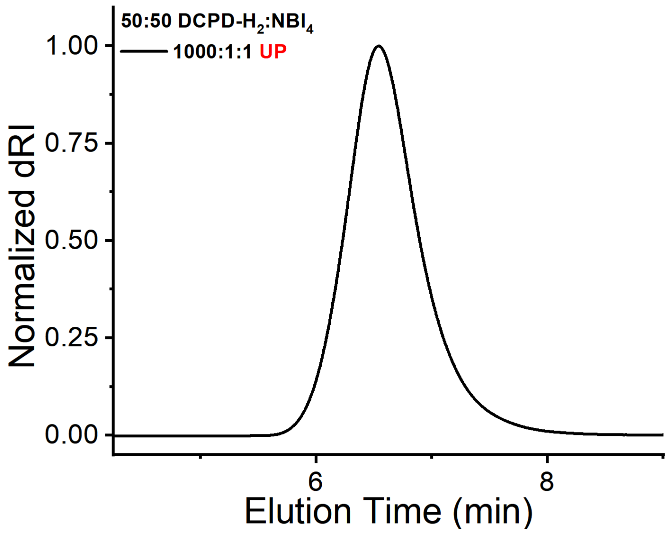

Figure S224: Representative SEC of 50 mol% NBI_4 post-FROMP for 1000:1:1 **bottom up** (M_n = 220 ± 10 kg/mol, $Đ$ = 1.38 ± 0.03).

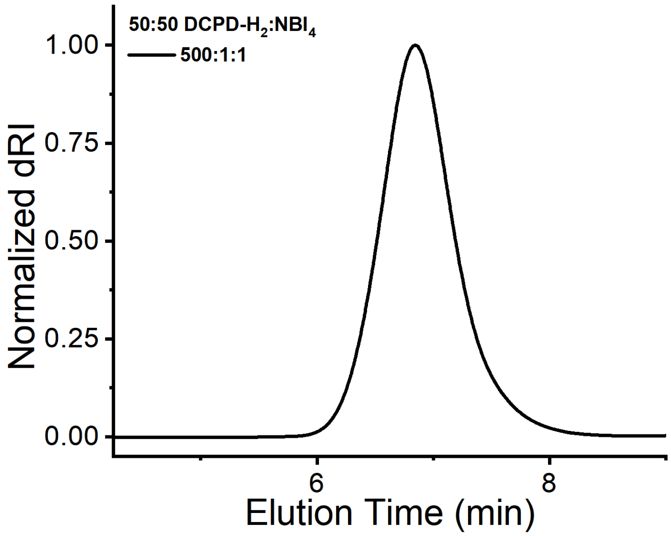

Figure S225: Representative SEC of 50 mol% NBI_4 post-FROMP for 500:1:1 (M_n = 140 ± 1 kg/mol, $Đ$ = 1.31 ± 0.05).

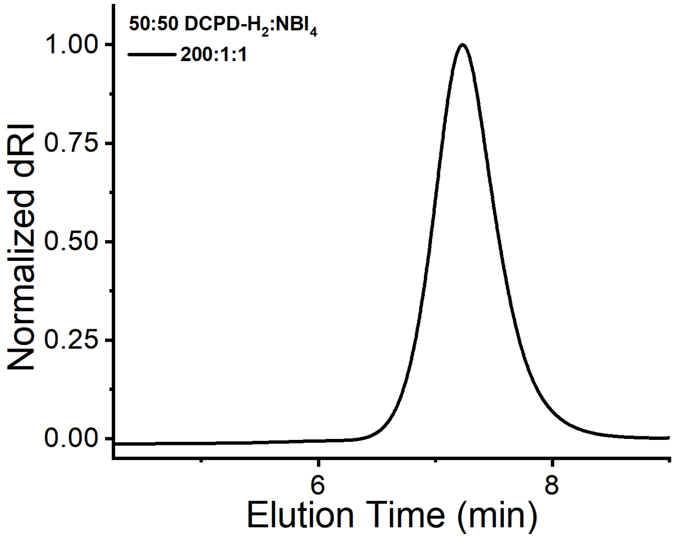

Figure S226: Representative SEC of 50 mol% NBI_4 post-FROMP for 200:1:1 (M_n = 59 ± 2 kg/mol, $Đ$ = 1.30 ± 0.04).

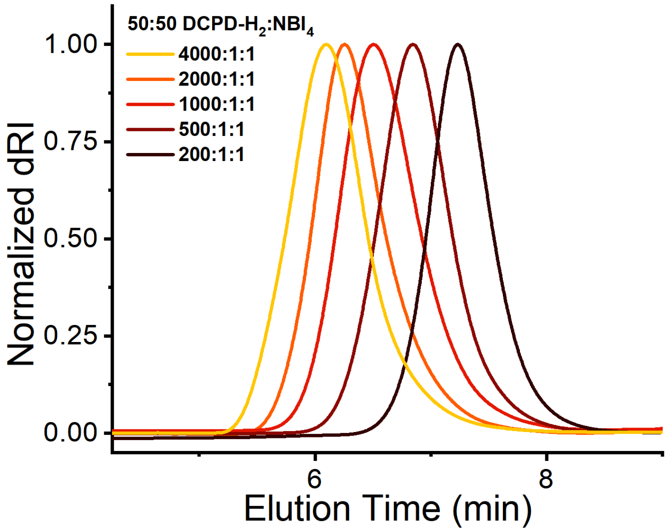

Figure S227: Representative SEC overlay of 50 mol% NBI_4 post-FROMP for varied loadings (x:1:1 monomer:initiator:inhibitor).

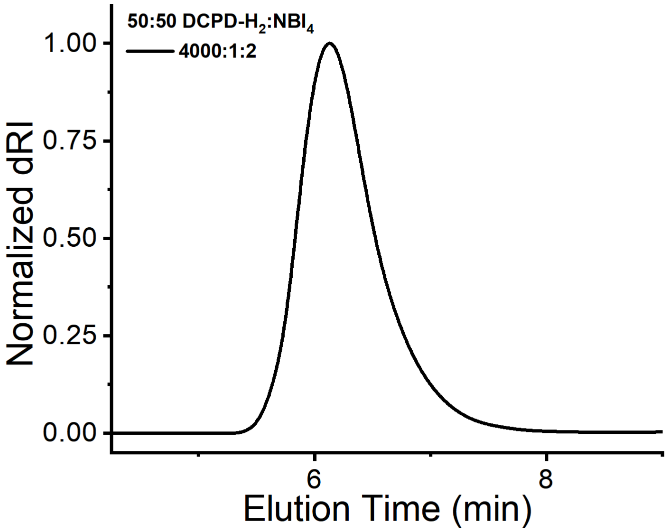

Figure S228: Representative SEC of 50 mol% NBI_4 post-FROMP for 4000:1:2 (M_n = 510 ± 60 kg/mol, $Đ$ = 1.39 ± 0.05).

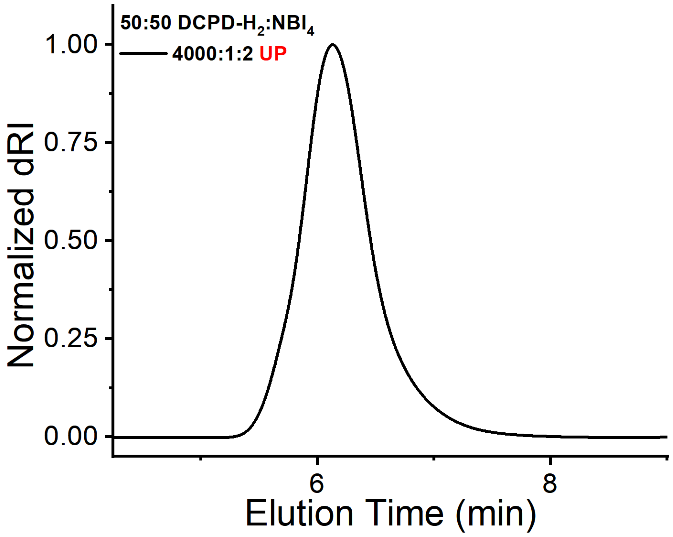

Figure S229: Representative SEC of 50 mol% NBI_4 post-FROMP for 4000:1:2 **bottom up** (M_n = 590 ± 90 kg/mol, $Đ$ = 1.39 ± 0.07).

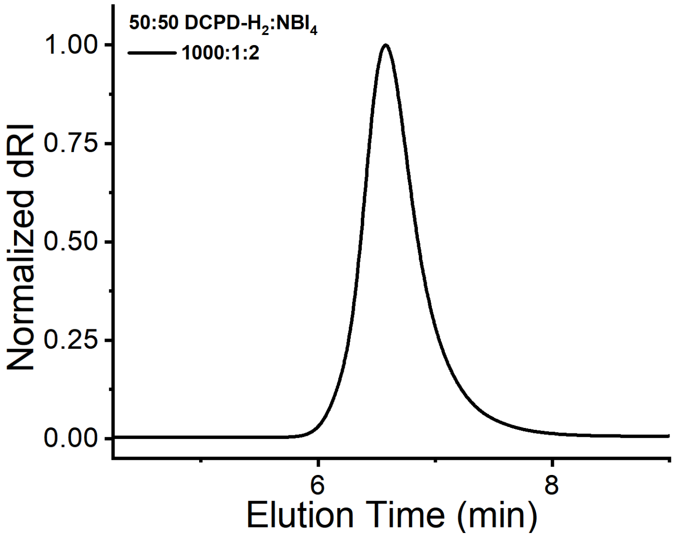

Figure S230: Representative SEC of 50 mol% NBI_4 post-FROMP for 1000:1:2 (M_n = 180 ± 20 kg/mol, $Đ$ = 1.26 ± 0.02).

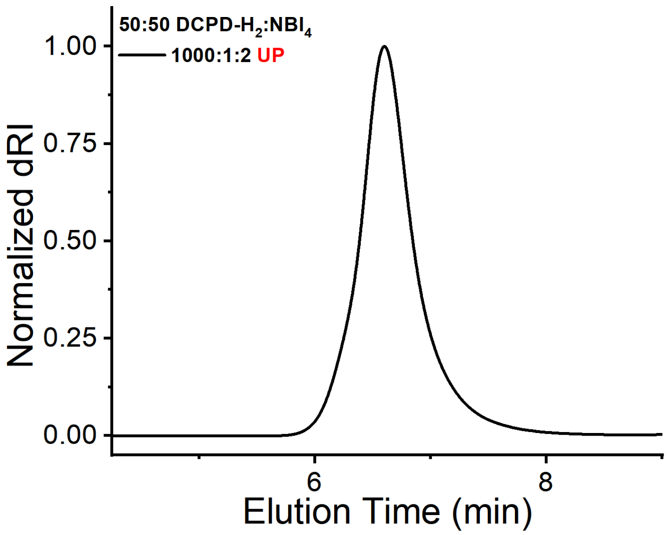

Figure S231: Representative SEC of 50 mol% NBI_4 post-FROMP for 1000:1:2 **bottom up** (M_n = 220 ± 10 kg/mol, $Đ$ = 1.21 ± 0.01).

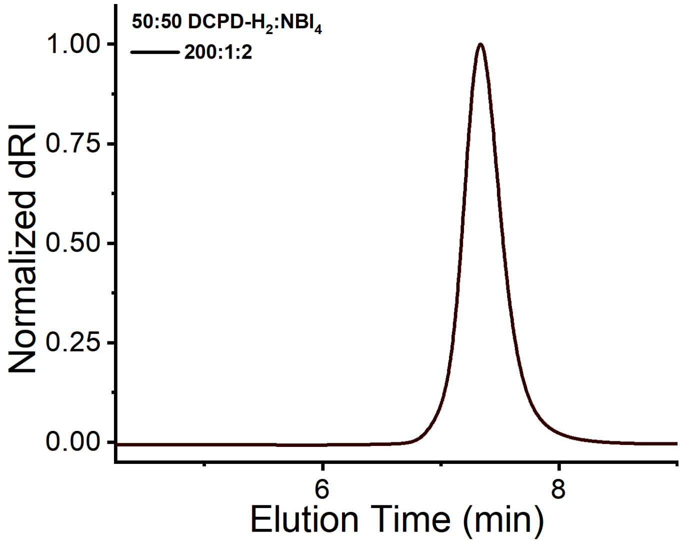

Figure S232: Representative SEC of 50 mol% NBI_4 post-FROMP for 200:1:2 (M_n = 54 ± 1 kg/mol, $Đ$ = 1.07 ± 0.01).

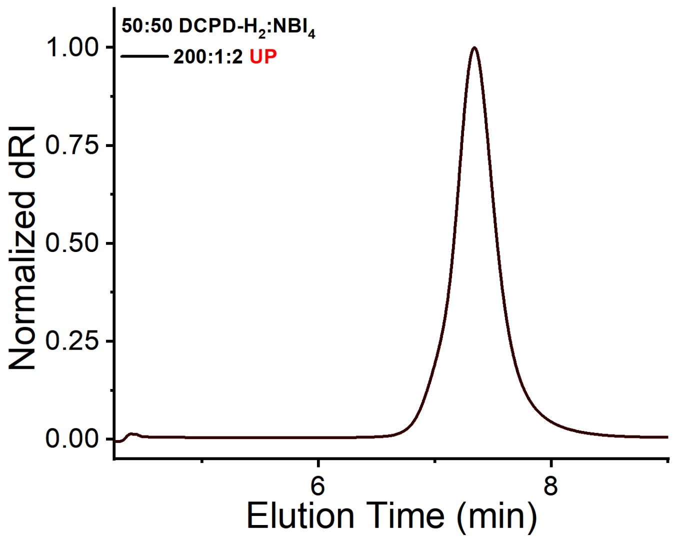

Figure S233: Representative SEC of 50 mol% NBI_4 post-FROMP for 200:1:2 **bottom up** (M_n = 56 ± 6 kg/mol, $Đ$ = 1.12 ± 0.01).

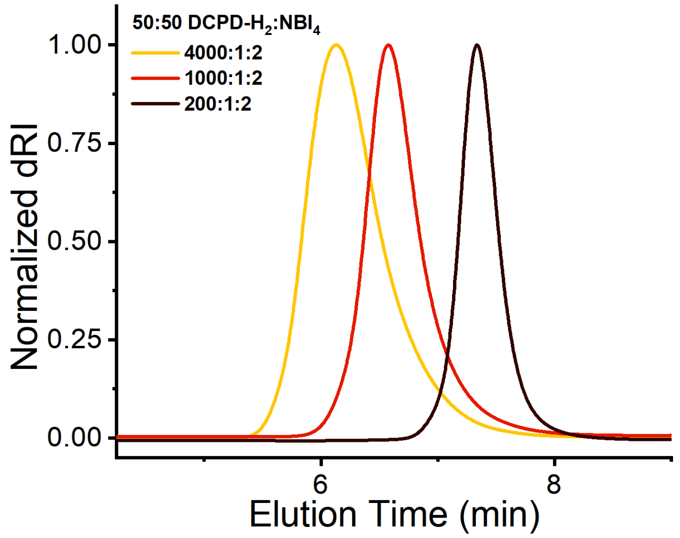

Figure S234: Representative SEC overlay of 50 mol% NBI_4 post-FROMP for varied loadings (x:1:2 monomer:initiator:inhibitor).

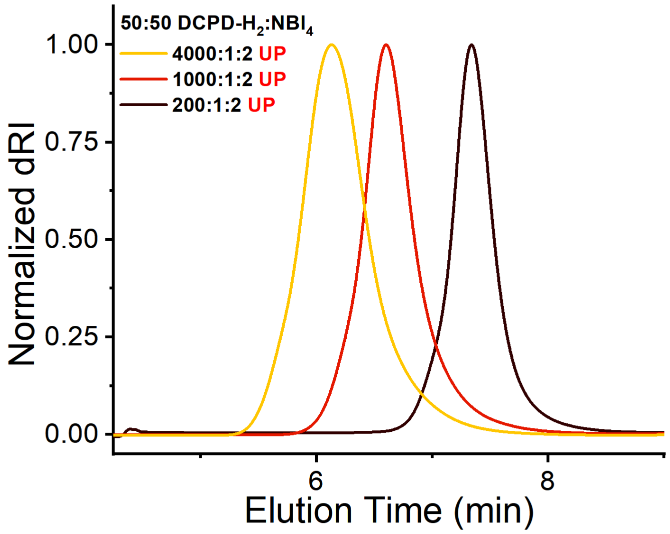

Figure S235: Representative SEC overlay of 50 mol% NBI$_4$ post-FROMP for varied loadings from **bottom up** (x:1:2 monomer:initiator:inhibitor).

Table S9: SEC molecular weight and dispersity (n = 3) for varying mol% NBI$_4$ post-FROMP at varying loadings.

Mixture	Monomer (equiv)	Initiator (equiv)	Inhibitor (equiv)	Direction	DP	error	*Đ*	error
90:10	4000	1	1	downward	3000	300	1.71	0.02
90:10	2000	1	1	downward	1800	500	1.7	0.2
90:10	1000	1	1	downward	1100	100	1.69	0.09
90:10	500	1	1	downward	650	20	1.58	0.07
90:10	200	1	1	downward	360	30	1.52	0.08
75:25	4000	1	1	downward	4500	600	1.45	0.09
75:25	2000	1	1	downward	2700	100	1.36	0.12
75:25	1000	1	1	downward	1340	40	1.46	0.05
75:25	500	1	1	downward	800	60	1.37	0.06
75:25	200	1	1	downward	380	40	1.40	0.11
50:50	4000	1	1	downward	4000	200	1.33	0.05
50:50	2000	1	1	downward	2300	400	1.36	0.14
50:50	1000	1	1	downward	1300	100	1.38	0.15
50:50	1000	1	1	upward	1220	50	1.38	0.03
50:50	500	1	1	downward	760	2	1.31	0.05
50:50	200	1	1	downward	330	10	1.30	0.04
50:50	4000	1	**2**	downward	2900	300	1.39	0.05
50:50	4000	1	**2**	upward	3300	500	1.39	0.07
50:50	1000	1	**2**	downward	1000	100	1.26	0.01
50:50	1000	1	**2**	upward	1240	40	1.21	0.01
50:50	200	1	**2**	downward	310	5	1.07	0.01
50:50	200	1	**2**	upward	320	30	1.12	0.01

Dynamic Scanning Calorimetry (DSC) Post-FROMP:

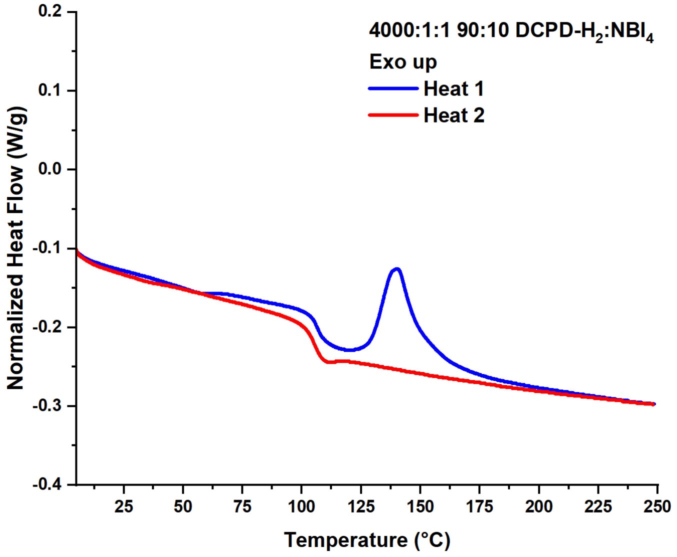

Figure S236: Representative DSC of 10 mol% NBI_4 post-FROMP for 4000:1:1 ($T_{g,\text{ Heat 1}}$ = 106 ± 1 °C, $H_{r,\text{ residual}}$ = 6 ± 5 J/g, $T_{g,\text{ Heat 2}}$ = 109 ± 7 °C). Exo up, first (blue) and second heat (red) cycle.

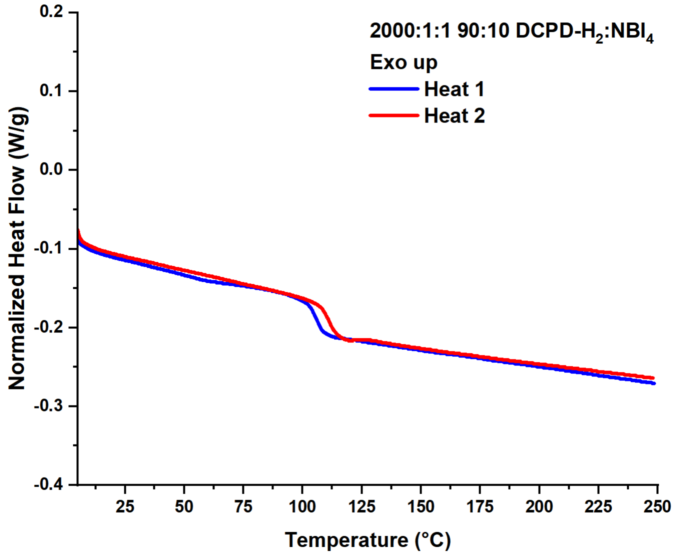

Figure S237: Representative DSC of 10 mol% NBI_4 post-FROMP for 2000:1:1 ($T_{g,\text{ Heat 1}}$ = 107 ± 1 °C, $H_{r,\text{ residual}}$ = 5 ± 4 J/g, $T_{g,\text{ Heat 2}}$ = 106 ± 4 °C). Exo up, first (blue) and second heat (red) cycle.

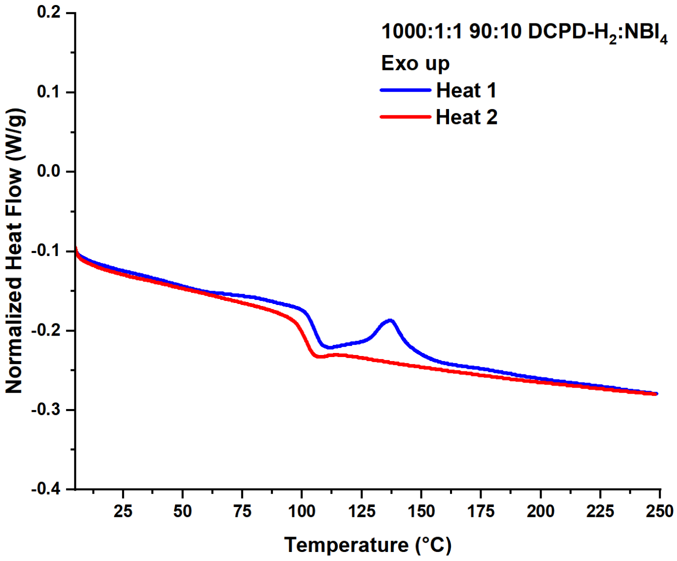

Figure S238: Representative DSC of 10 mol% NBI_4 post-FROMP for 1000:1:1 ($T_{g,\text{ Heat 1}}$ = 105 ± 1 °C, $H_{r,\text{ residual}}$ = 1 ± 1 J/g, $T_{g,\text{ Heat 2}}$ = 104 ± 2 °C). Exo up, first (blue) and second heat (red) cycle.

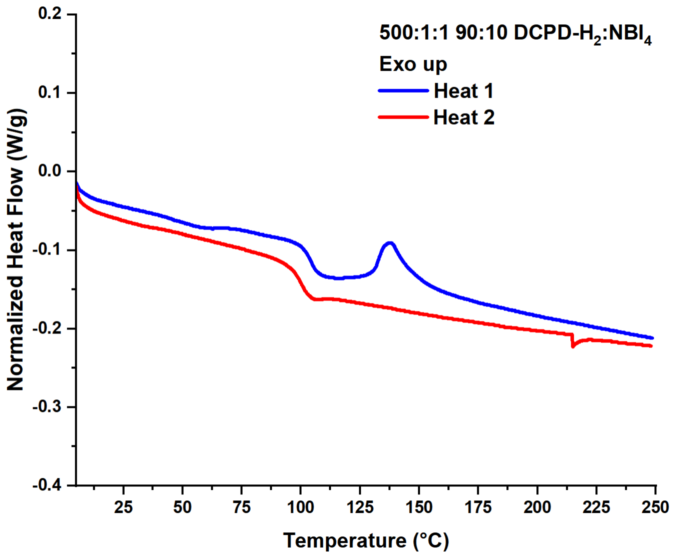

Figure S239: Representative DSC of 10 mol% NBI_4 post-FROMP for 500:1:1 ($T_{g,\text{ Heat 1}}$ = 104 ± 1 °C, $H_{r,\text{ residual}}$ = 5 ± 2 J/g, $T_{g,\text{ Heat 2}}$ = 99 ± 1 °C). Exo up, first (blue) and second heat (red) cycle.

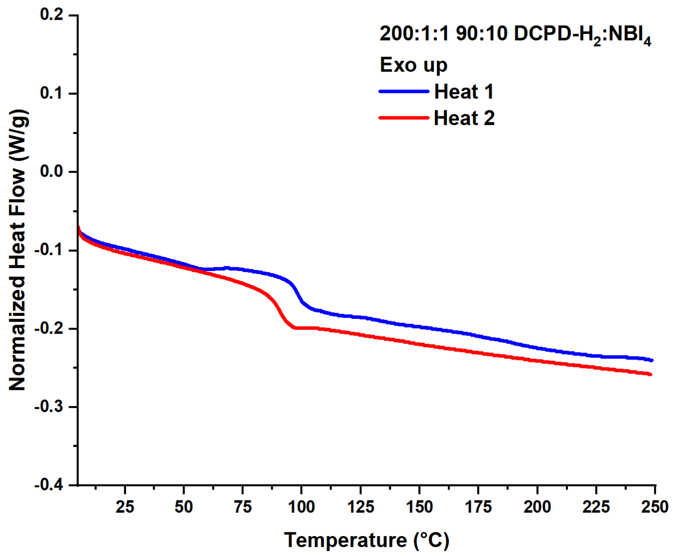

Figure S240: Representative DSC of 10 mol% NBI_4 post-FROMP for 200:1:1 ($T_{g,\text{ Heat 1}}$ = 98 ± 1 °C, $H_{r,\text{ residual}}$ = 0 J/g, $T_{g,\text{ Heat 2}}$ = 91 ± 1 °C). Exo up, first (blue) and second heat (red) cycle.

Table S10: Glass Transition (T_g) for first and second heat for 10 mol% NBI_4 post-FROMP at varied loadings.

Monomer (equiv)	Initiator (equiv)	Inhibitor (equiv)	Direction	$T_{g,\text{ Heat 1}}$ (°C)	error	$T_{g,\text{ Heat 2}}$ (°C)	error
4000	1	1	downward	106.6	0.42	109	7
2000	1	1	downward	106.5	0.8	106	4
1000	1	1	downward	105.3	0.2	104	2
500	1	1	downward	104.2	0.1	98.8	0.4
200	1	1	downward	98.2	0.4	90.9	0.8

Table S11: Residual heat of reaction ($H_{r,\text{ residual}}$) from first heat cycle and percent monomer conversion for 10 mol% NBI_4 post-FROMP at varied loadings.

Monomer (equiv)	Initiator (equiv)	Inhibitor (equiv)	Direction	$H_{r,\text{ residual}}$ (J/g)	error	Calculated Conversion (%)	error
4000	1	1	downward	6	5	98	1
2000	1	1	downward	5	4	98	1
1000	1	1	downward	1	2	99.7	0.5
500	1	1	downward	5	2	98.4	0.5
200	1	1	downward	0	0	100	0

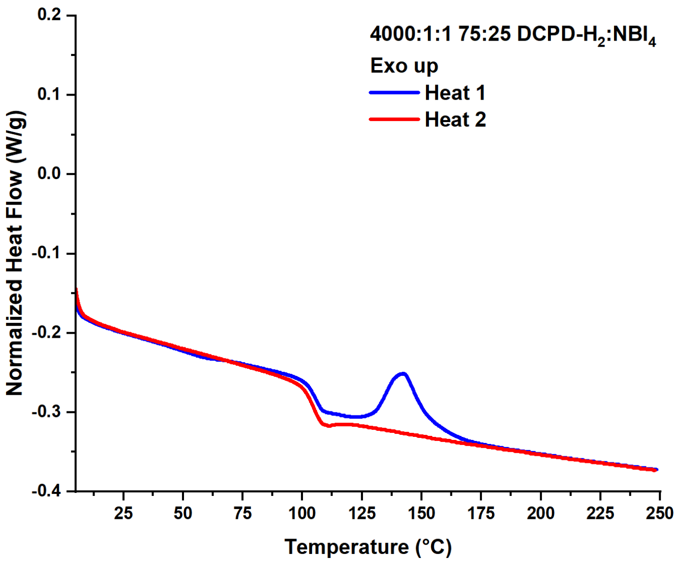

Figure S241: Representative DSC of 25 mol% NBI$_4$ post-FROMP for 4000:1:1 ($T_{g,\text{ Heat 1}}$ = 106 ± 1 °C, $H_{r,\text{ residual}}$ = 7 ± 4 J/g, $T_{g,\text{ Heat 2}}$ = 104 ± 1 °C). Exo up, first (blue) and second heat (red) cycle.

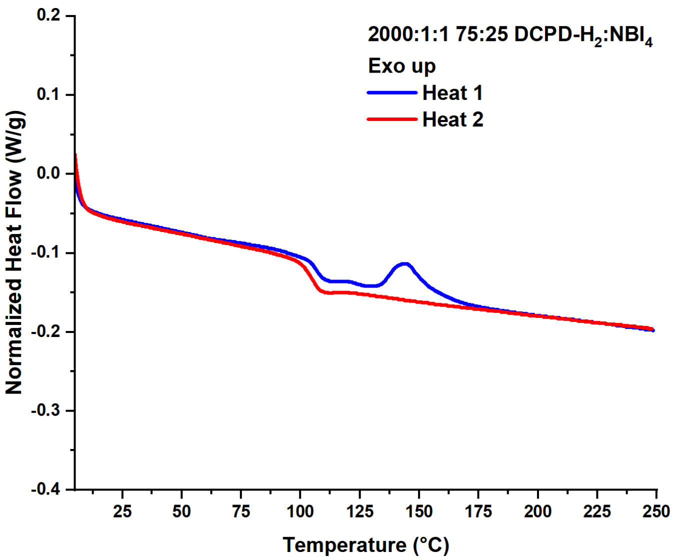

Figure S242: Representative DSC of 25 mol% NBI$_4$ post-FROMP for 2000:1:1 ($T_{g,\text{ Heat 1}}$ = 106 ± 1 °C, $H_{r,\text{ residual}}$ = 7 ± 3 J/g, $T_{g,\text{ Heat 2}}$ = 103 ± 1 °C). Exo up, first (blue) and second heat (red) cycle.

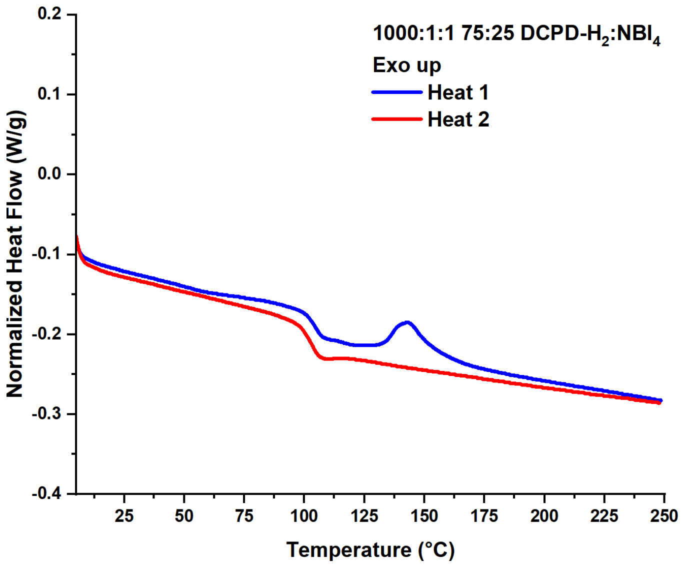

Figure S243: Representative DSC of 25 mol% NBI_4 post-FROMP for 1000:1:1 ($T_{g,\ Heat\ 1}$ = 105 ± 1 °C, $H_{r,\ residual}$ = 9 ± 4 J/g, $T_{g,\ Heat\ 2}$ = 102 ± 1 °C). Exo up, first (blue) and second heat (red) cycle.

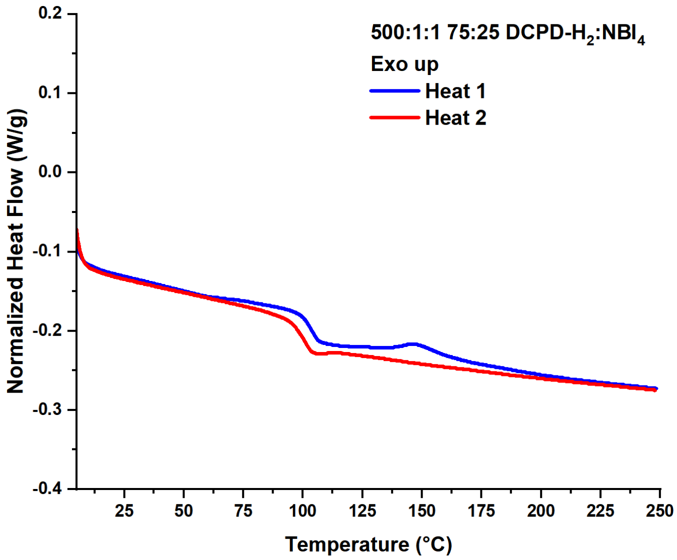

Figure S244: Representative DSC of 25 mol% NBI_4 post-FROMP for 500:1:1 ($T_{g,\ Heat\ 1}$ = 104 ± 1 °C, $H_{r,\ residual}$ = 6 ± 3 J/g, $T_{g,\ Heat\ 2}$ = 100 ± 1 °C). Exo up, first (blue) and second heat (red) cycle.

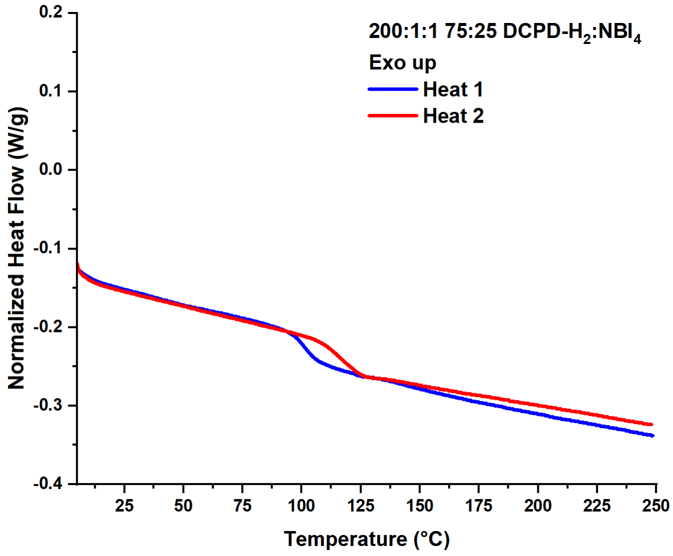

Figure S245: Representative DSC of 25 mol% NBI_4 post-FROMP for 200:1:1 ($T_{g,\ Heat\ 1}$ = 100 ± 1 °C, $H_{r,\ residual}$ = 0 J/g, $T_{g,\ Heat\ 2}$ = 105 ± 10 °C). Exo up, first (blue) and second heat (red) cycle.

Table S12: Glass Transition (T_g) for first and second heat for 25 mol% NBI_4 post-FROMP at varied loadings.

Monomer (equiv)	Initiator (equiv)	Inhibitor (equiv)	Direction	$T_{g,\ Heat\ 1}$ (°C)	error	$T_{g,\ Heat\ 2}$ (°C)	error
4000	1	1	downward	105.5	0.4	104.1	0.6
2000	1	1	downward	106	1	103.3	0.2
1000	1	1	downward	105	1	102.0	0.5
500	1	1	downward	103.8	0.5	99.9	0.8
200	1	1	downward	99.6	0.9	105	10

Table S13: Residual heat of reaction ($H_{r,\ residual}$) from first heat cycle and percent monomer conversion for 25 mol% NBI_4 post-FROMP at varied loadings.

Monomer (equiv)	Initiator (equiv)	Inhibitor (equiv)	Direction	$H_{r,\ residual}$ (J/g)	error	Calculated Conversion (%)	error
4000	1	1	downward	7	4	98	1
2000	1	1	downward	7	3	98	1
1000	1	1	downward	9	4	97	1
500	1	1	downward	6	3	98.3	0.8
200	1	1	downward	0	0	100	0

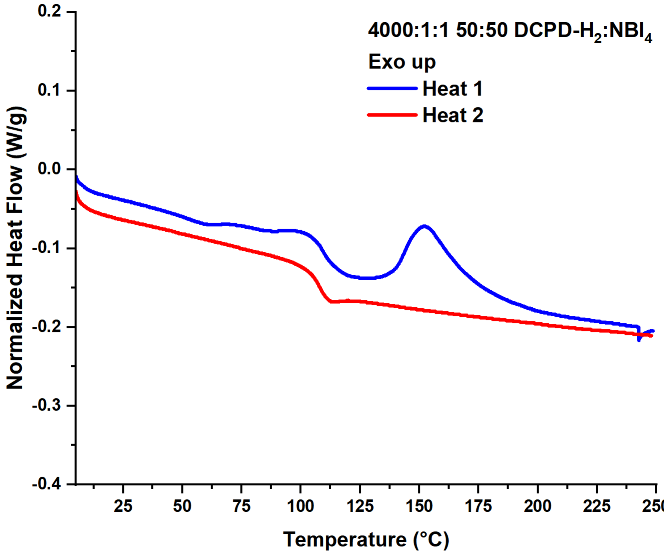

Figure S246: Representative DSC of 50 mol% NBI4 post-FROMP for 4000:1:1 ($T_{g,\text{ Heat 1}}$ = 110 ± 1 °C, $H_{r,\text{ residual}}$ 9 ± 2= J/g, $T_{g,\text{ Heat 2}}$ = 107 ± 1 °C). Exo up, first (blue) and second heat (red) cycle.

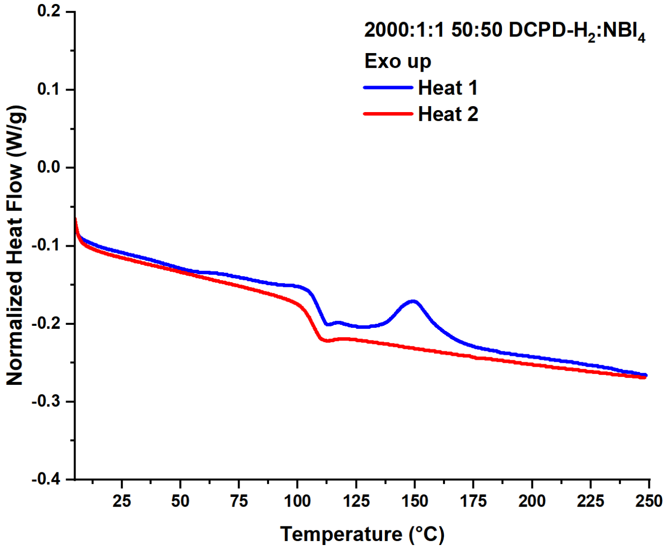

Figure S247: Representative DSC of 50 mol% NBI4 post-FROMP for 2000:1:1 ($T_{g,\text{ Heat 1}}$ = 108 ± 1 °C, $H_{r,\text{ residual}}$ = 4 ± 1 J/g, $T_{g,\text{ Heat 2}}$ = 106 ± 1 °C). Exo up, first (blue) and second heat (red) cycle.

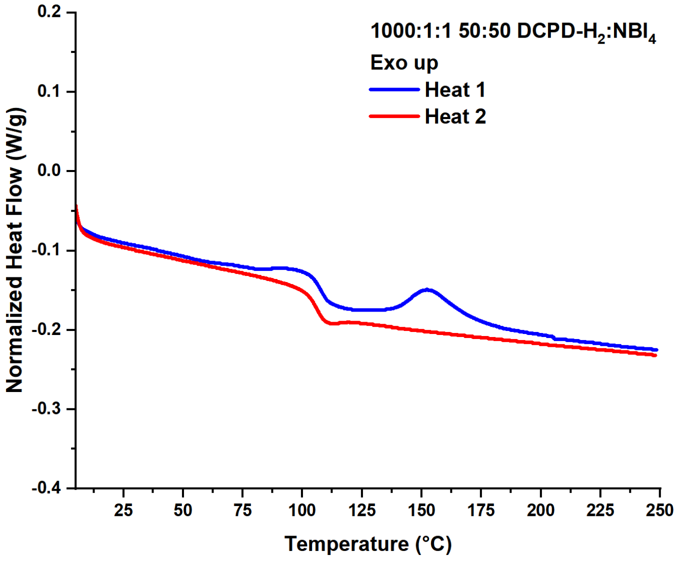

Figure S248: Representative DSC of 50 mol% NBI_4 post-FROMP for 1000:1:1 ($T_{g,\text{ Heat 1}}$ = 109 ± 1 °C, $H_{r,\text{ residual}}$ = 5 ± 1 J/g, $T_{g,\text{ Heat 2}}$ = 105 ± 1 °C). Exo up, first (blue) and second heat (red) cycle.

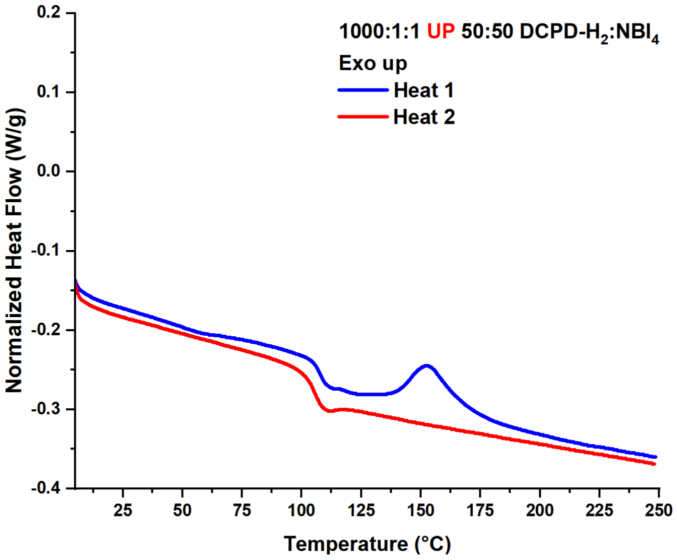

Figure S249: Representative DSC of 50 mol% NBI_4 post-FROMP for 1000:1:1 **bottom up** ($T_{g,\text{ Heat 1}}$ = 109 ± 2 °C, $H_{r,\text{ residual}}$ = 8 ± 1 J/g, $T_{g,\text{ Heat 2}}$ = 106 ± 2 °C). Exo up, first (blue) and second heat (red) cycle.

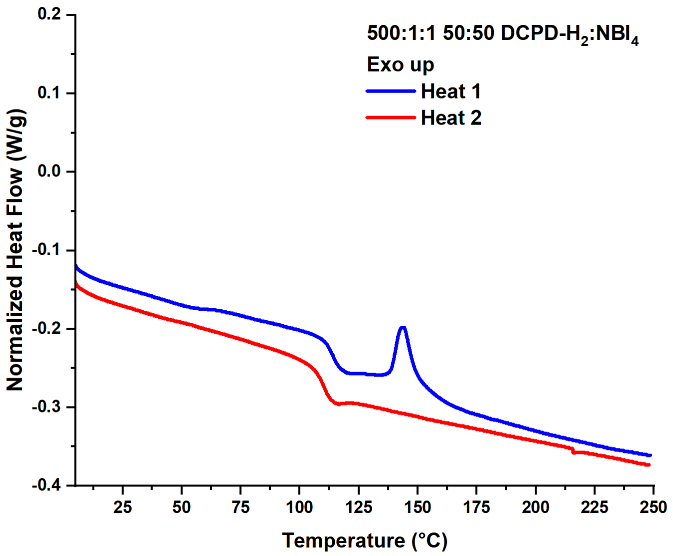

Figure S250: Representative DSC of 50 mol% NBI4 post-FROMP for 500:1:1 ($T_{g,\text{ Heat 1}}$ = 108± 1 °C, $H_{r,\text{ residual}}$ = 5 ± 2 J/g, $T_{g,\text{ Heat 2}}$ = 103 ± 1 °C). Exo up, first (blue) and second heat (red) cycle.

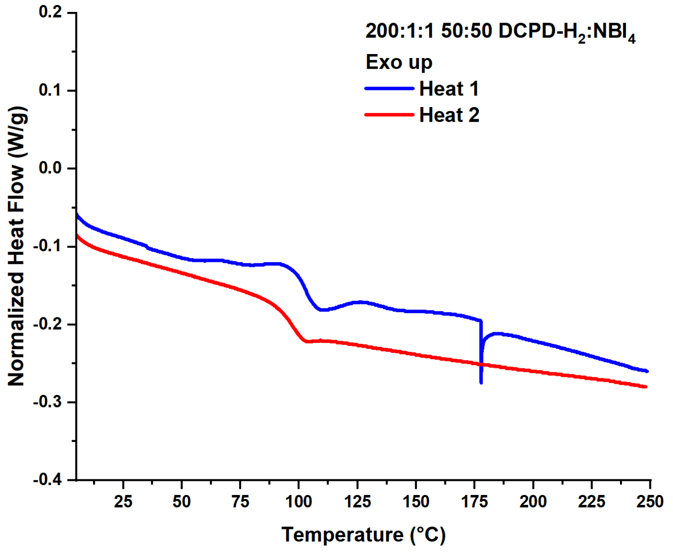

Figure S251: Representative DSC of 50 mol% NBI4 post-FROMP for 200:1:1 ($T_{g,\text{ Heat 1}}$ = 104 ± 1 °C, $H_{r,\text{ residual}}$ = 0 J/g, $T_{g,\text{ Heat 2}}$ = 98 ± 1 °C). Exo up, first (blue) and second heat (red) cycle.

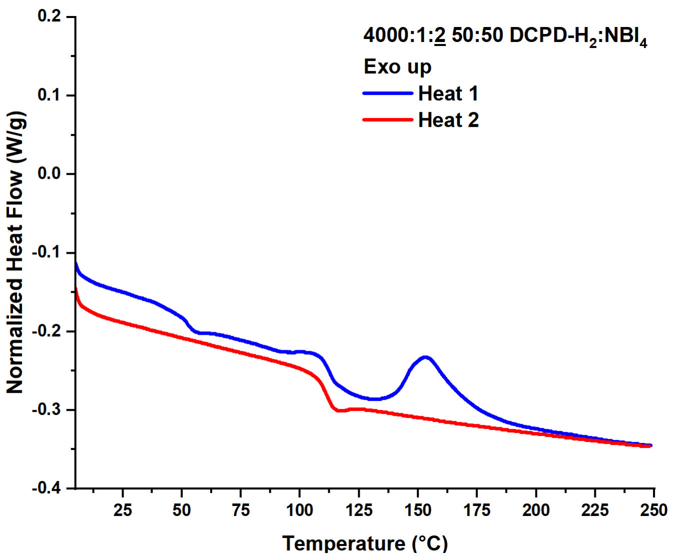

Figure S252: Representative DSC of 50 mol% NBI4 post-FROMP for 4000:1:2 ($T_{g,\text{ Heat 1}}$ = 111 ± 2 °C, $H_{r,\text{ residual}}$ = 4 ± 3 J/g, $T_{g,\text{ Heat 2}}$ = 110 ± 3 °C). Exo up, first (blue) and second heat (red) cycle.

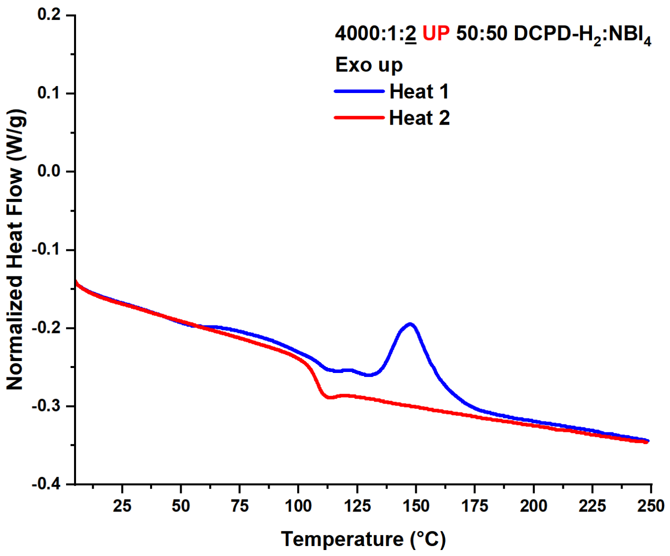

Figure S253: Representative DSC of 50 mol% NBI4 post-FROMP for 4000:1:2 **bottom up** ($T_{g,\text{ Heat 1}}$ = 111 ± 1 °C, $H_{r,\text{ residual}}$ = 8 ± 2 J/g, $T_{g,\text{ Heat 2}}$ = 108 ± 1 °C). Exo up, first (blue) and second heat (red) cycle.

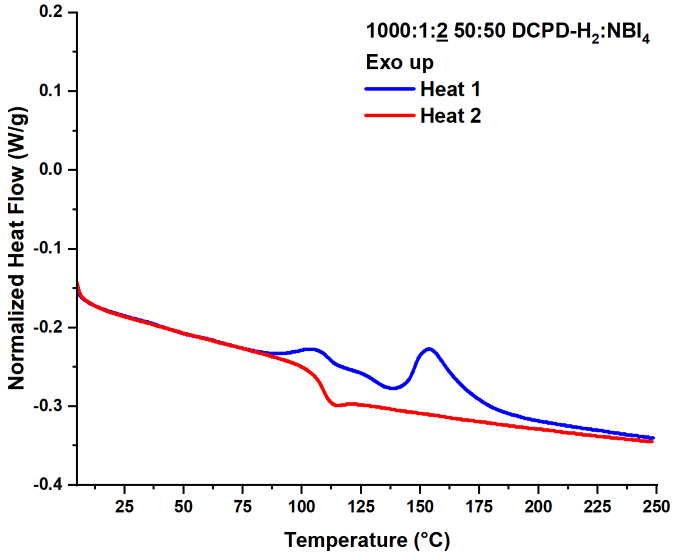

Figure S254: Representative DSC of 50 mol% NBI_4 post-FROMP for 1000:1:2 ($T_{g,\ Heat\ 1}$ = 111 ± 2 °C, $H_{r,\ residual}$ = 6 ± 1 J/g, $T_{g,\ Heat\ 2}$ = 107 ± 2 °C). Exo up, first (blue) and second heat (red) cycle.

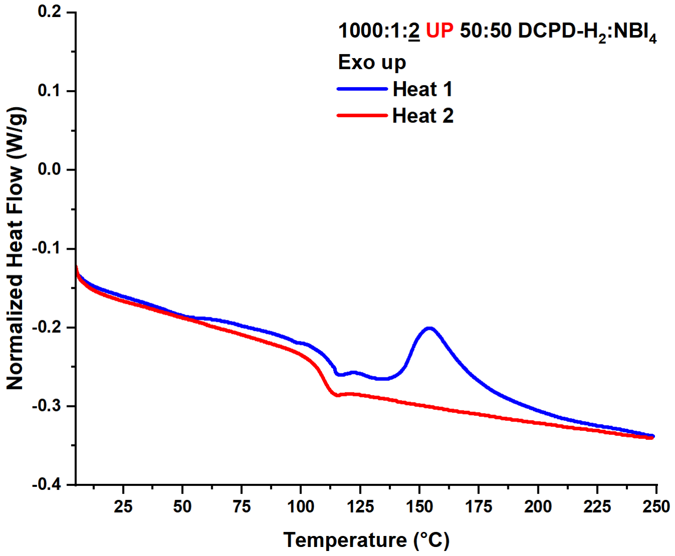

Figure S255: Representative DSC of 50 mol% NBI_4 post-FROMP for 1000:1:2 **bottom up** ($T_{g,\ Heat\ 1}$ = 113 ± 3 °C, $H_{r,\ residual}$ = 7 ± 5 J/g, $T_{g,\ Heat\ 2}$ = 111 ± 3 °C). Exo up, first (blue) and second heat (red) cycle.

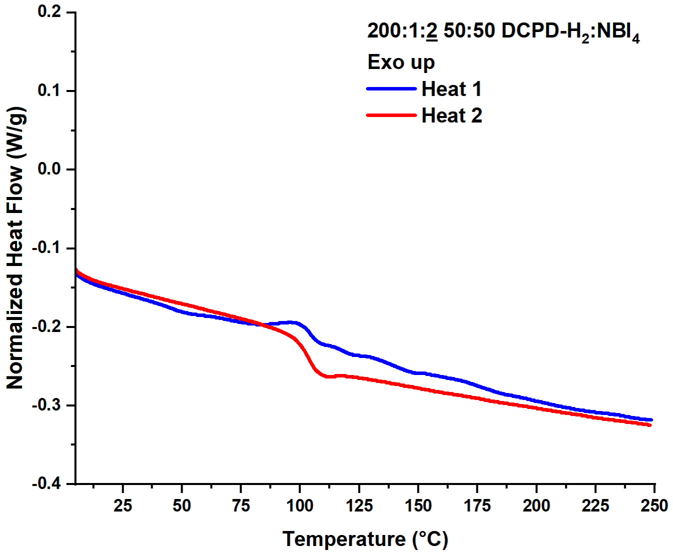

Figure S256: Representative DSC of 50 mol% NBI_4 post-FROMP for 200:1:2 ($T_{g,\text{ Heat 1}}$ = 69 °C (1/3 runs) 105 ± 1 °C (3/3 runs) & 131 ± 5 °C (2/3 runs), $H_{r,\text{ residual}}$ = 0 J/g, $T_{g,\text{ Heat 2}}$ = 103 ± 4 °C). Exo up, first (blue) and second heat (red) cycle.

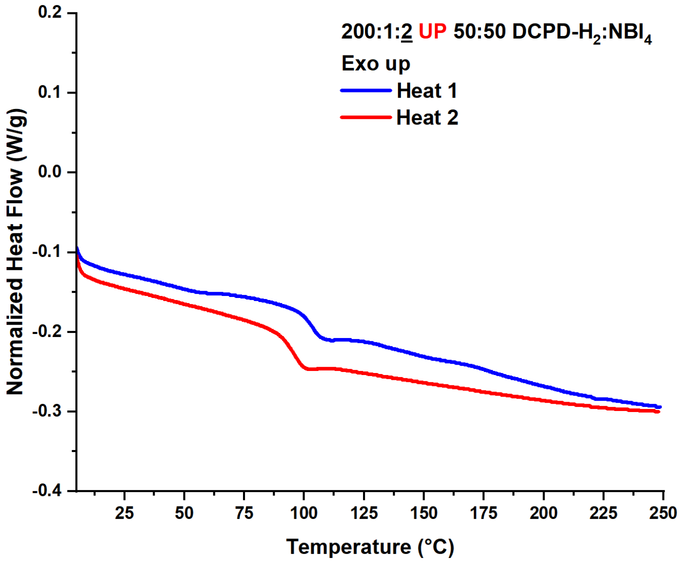

Figure S257: Representative DSC of 50 mol% NBI_4 post-FROMP for 200:1:2 **bottom up** ($T_{g,\text{ Heat 1}}$ = 102 ± 2 °C, $H_{r,\text{ residual}}$ =1 ± 2 J/g, $T_{g,\text{ Heat 2}}$ = 97 ± 1 °C). Exo up, first (blue) and second heat (red) cycle.

Table S14: Glass Transition (T_g) for first and second heat for 50 mol% NBI_4 post-FROMP at varied loadings.

Monomer (equiv)	Initiator (equiv)	Inhibitor (equiv)	Direction	$T_{g, Heat 1}$ (°C)	error	$T_{g, Heat 2}$ (°C)	error
4000	1	1	downward	109.8	0.2	107	1
2000	1	1	downward	108.1	0.3	106.0	0.7
1000	1	1	downward	109	1	104.6	0.5
1000	1	1	upward	109	2	106	2
500	1	1	downward	108	1	102.5	0.3
200	1	1	downward	104	1	98.0	0.8
4000	1	2	downward	111	2	110	3
4000	1	2	upward	111	1	108	1
1000	1	2	downward	111	2	107	2
1000	1	2	upward	113	3	111	3
200	1	2	downward	104	1	103	4
200	1	2	upward	102	2	97	1

Table S15: Residual heat of reaction ($H_{r,\ residual}$) from first heat cycle and percent monomer conversion for 50 mol% NBI_4 post-FROMP at varied loadings.

Monomer (equiv)	Initiator (equiv)	Inhibitor (equiv)	Direction	$H_{r, residual}$ (J/g)	error	Calculated Conversion (%)	error
4000	1	1	downward	9	2	97.0	0.9
2000	1	1	downward	4	1	98.5	0.3
1000	1	1	downward	5	1	98.1	0.2
1000	1	1	upward	8	1	97.0	0.4
500	1	1	downward	5	2	98.1	0.7
200	1	1	downward	1	2	99.5	0.6
4000	1	2	downward	4	3	99	1
4000	1	2	upward	8	2	97.3	0.6
1000	1	2	downward	6	1	97.8	0.4
1000	1	2	upward	7	5	98	2
200	1	2	downward	0	0	100	0
200	1	2	upward	1	2	99.5	0.7

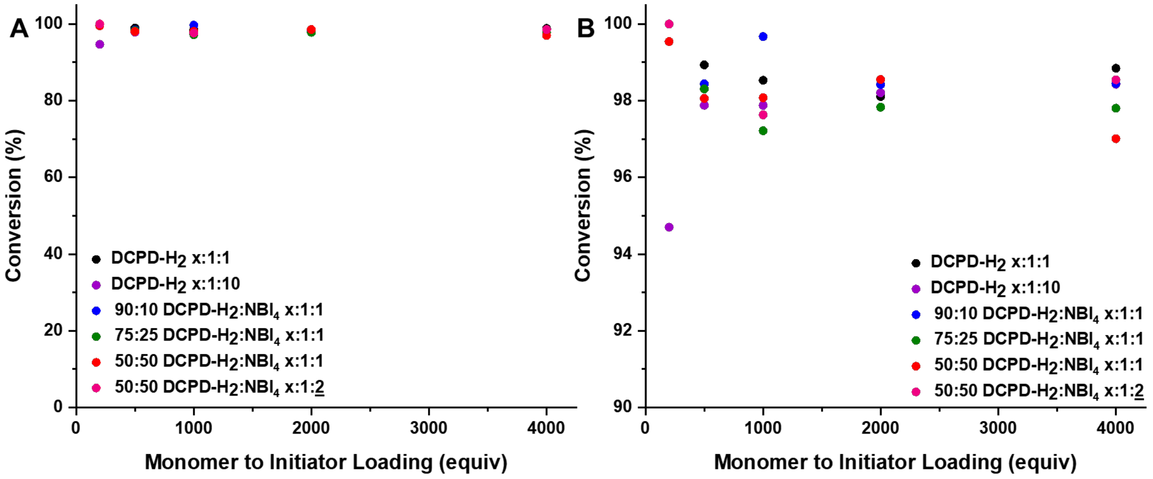

Figure S258: Percent conversion from DSC post cure residual heat of reaction ($H_{r,\ residual}$) for all loadings (A – 0-100% and B – 90-100% y-axis scale).

NMR of Copolymers:

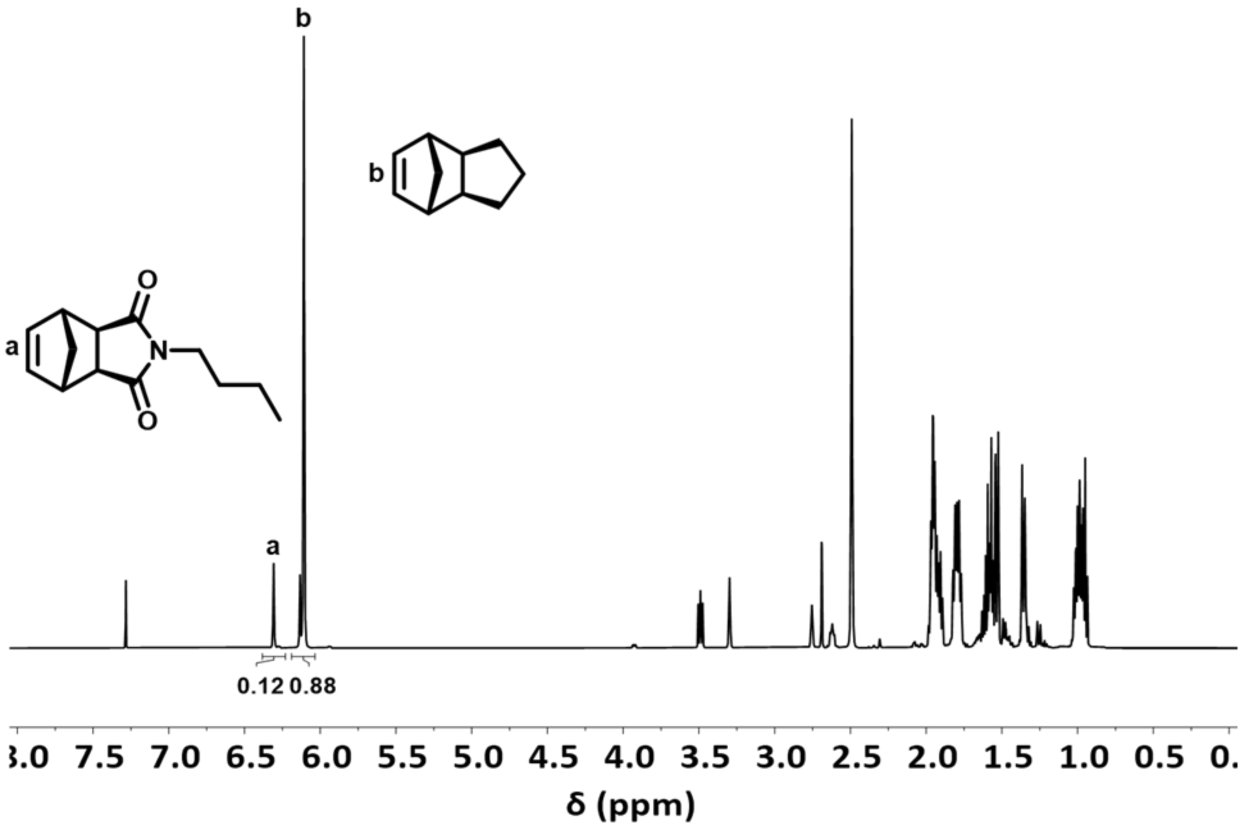

Figure S259: Representative NMR of 10 mol% NBI_4 monomer mixture.

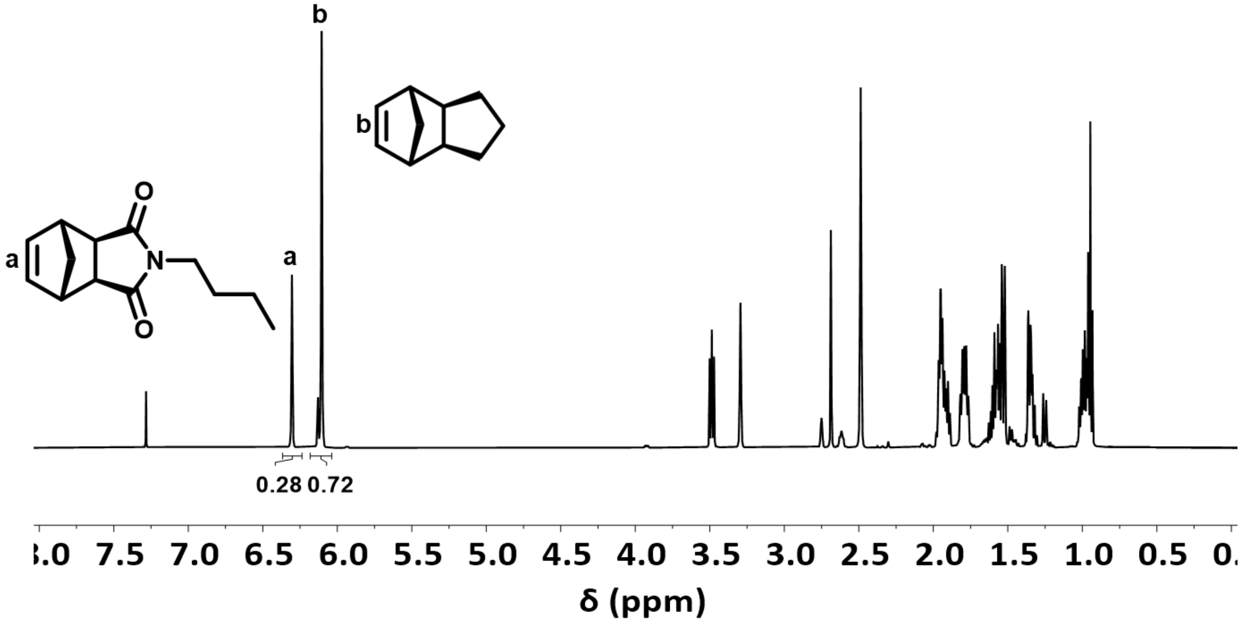

Figure S260: Representative NMR of 25 mol% monomer mixture.

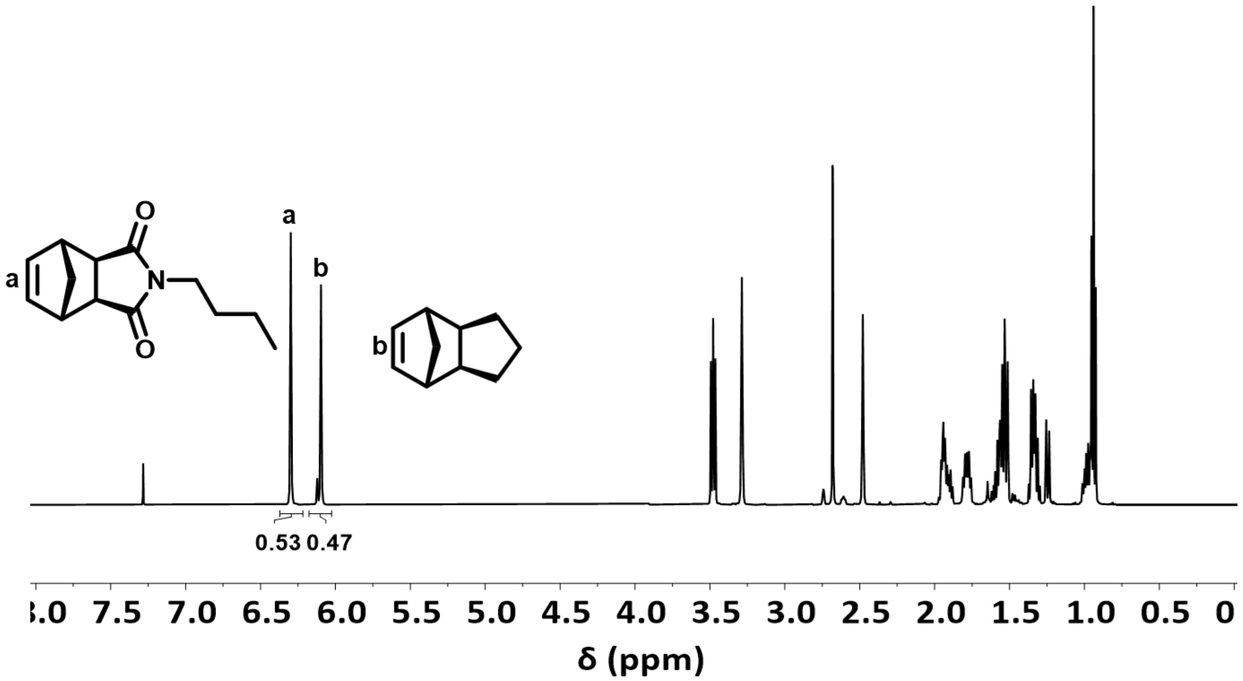

Figure S261: Representative NMR of 50 mol% NBI_4 monomer mixture.

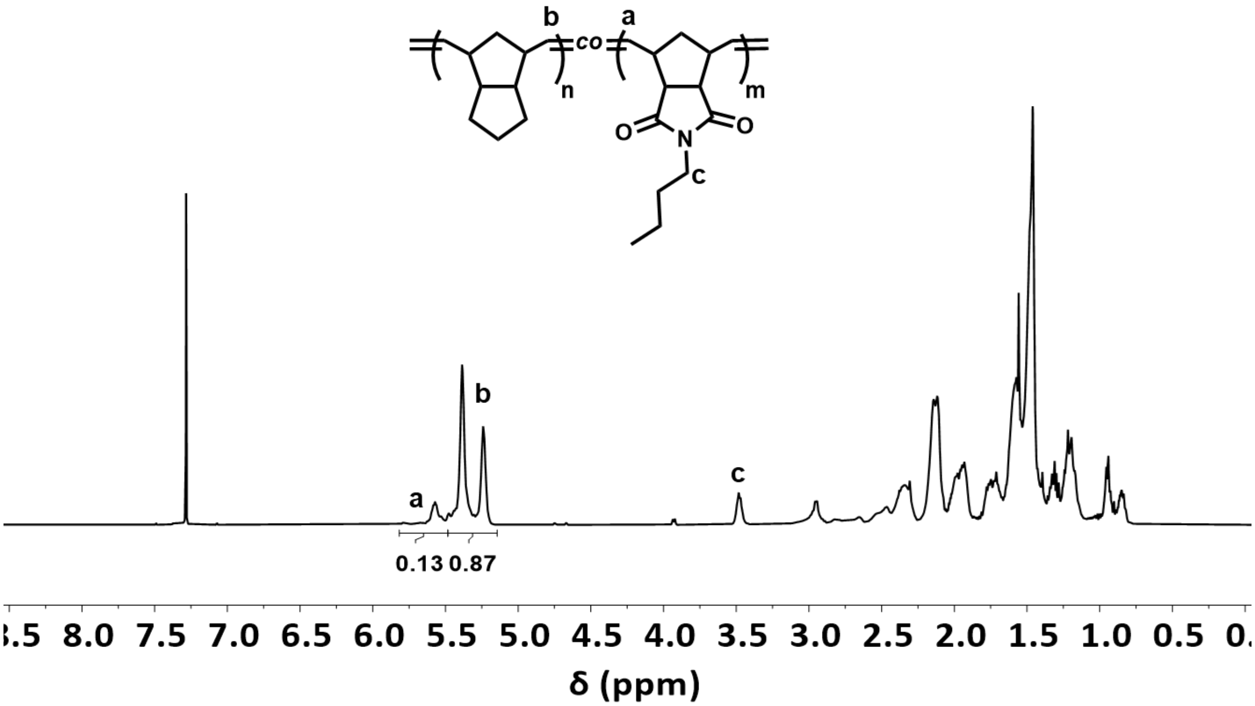

Figure S262: Representative NMR of 10 mol% NBI_4 post-FROMP for 4000:1:1.

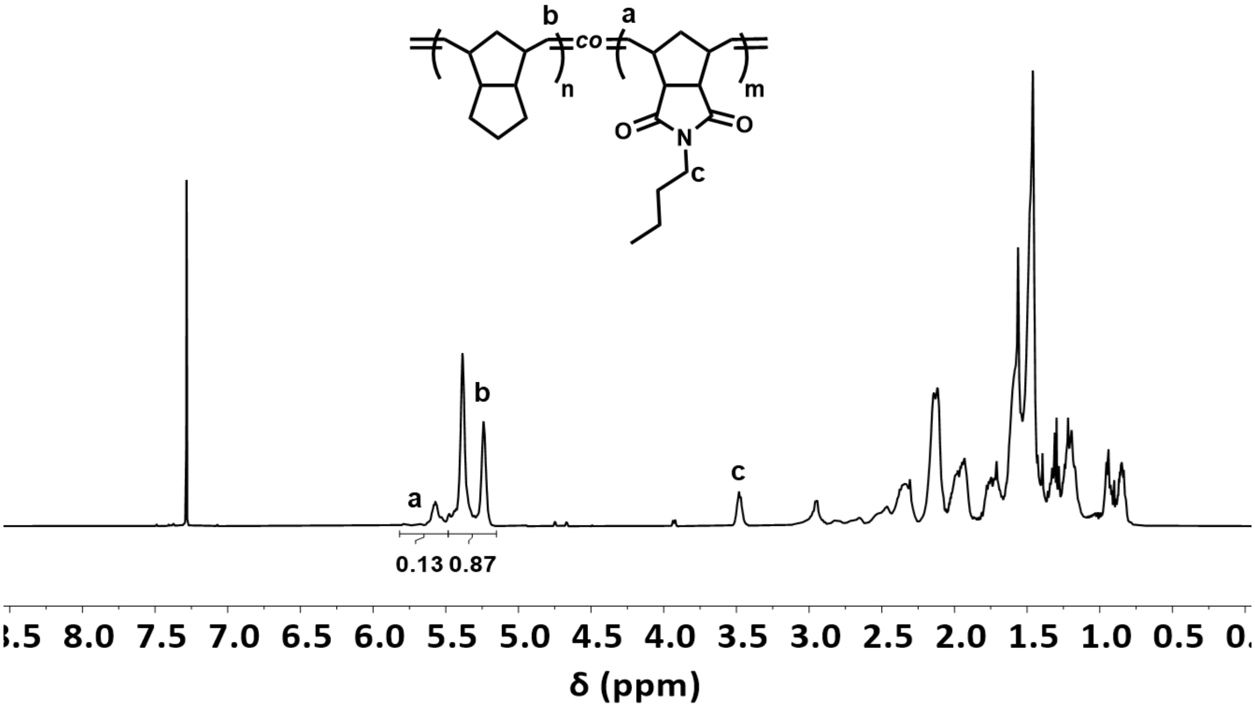

Figure S263: Representative NMR of 10 mol% NBI_4 post-FROMP for 2000:1:1.

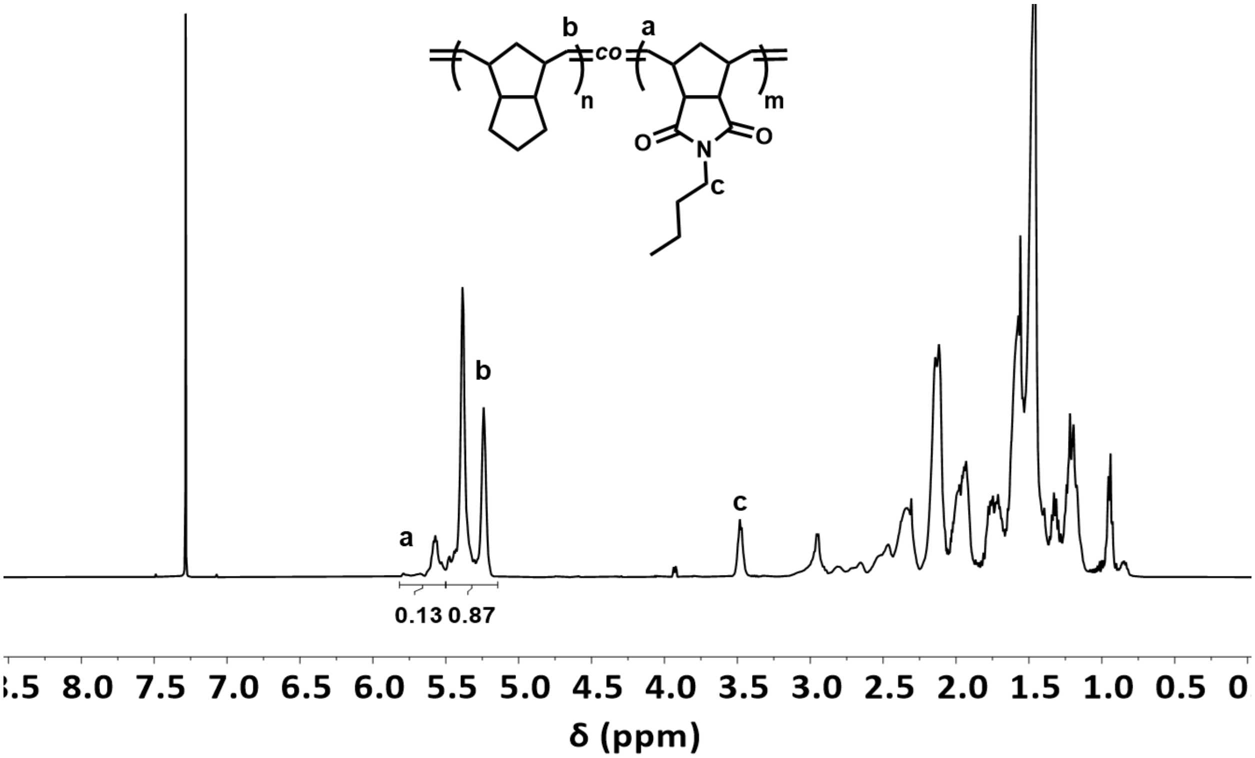

Figure S264: Representative NMR of 10 mol% NBI_4 post-FROMP for 1000:1:1.

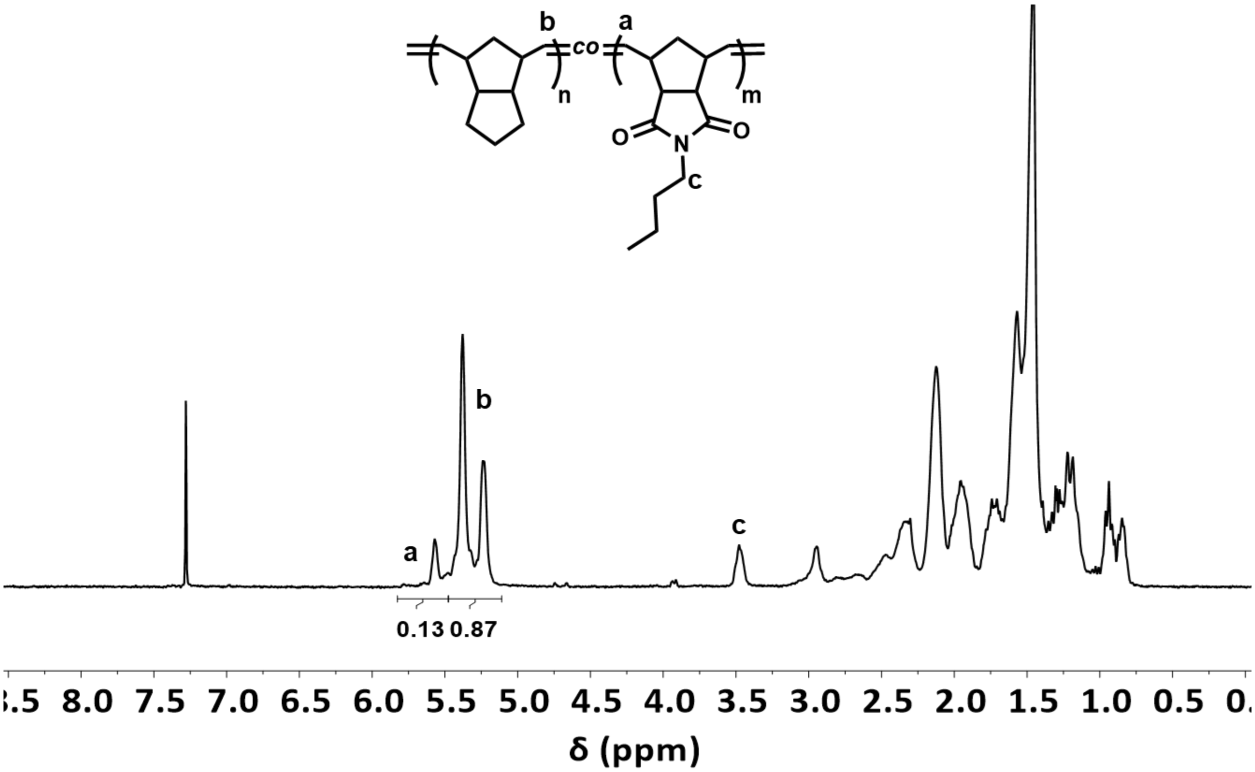

Figure S265: Representative NMR of 10 mol% NBI_4 post-FROMP for 500:1:1.

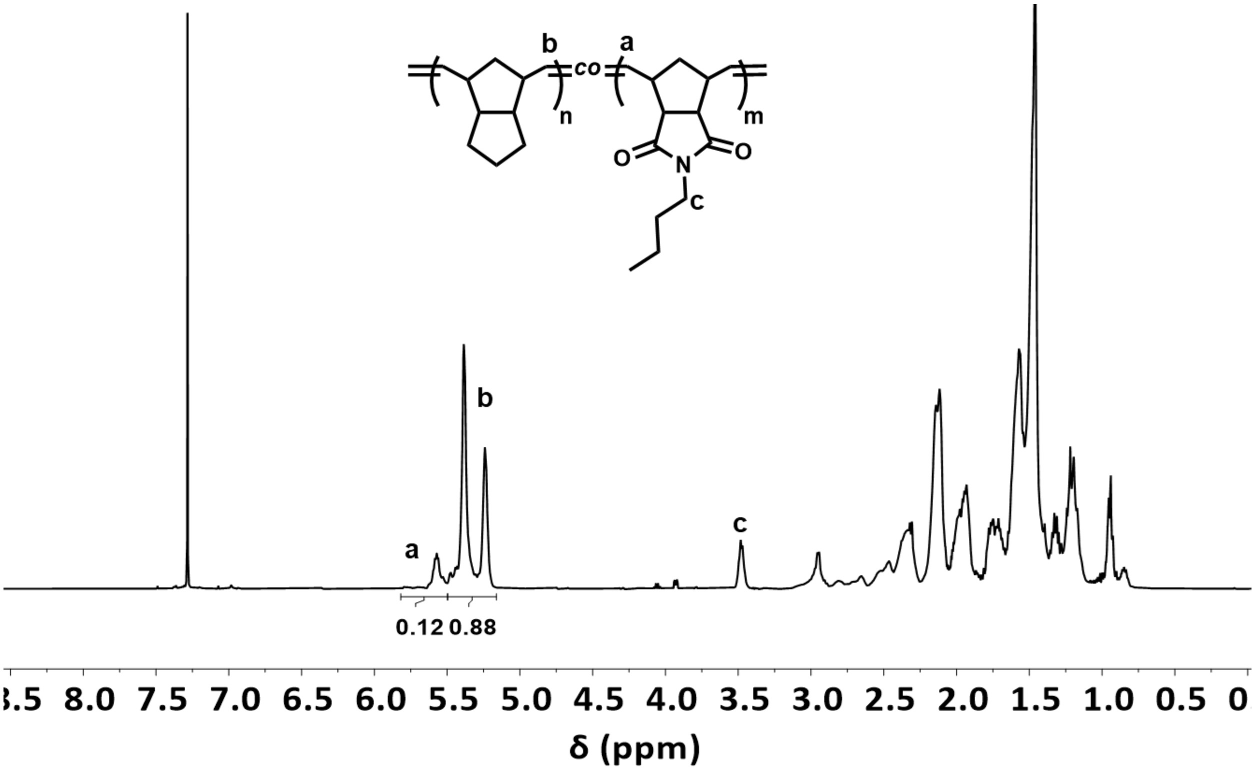

Figure S266: Representative NMR of 10 mol% NBI_4 post-FROMP for 200:1:1.

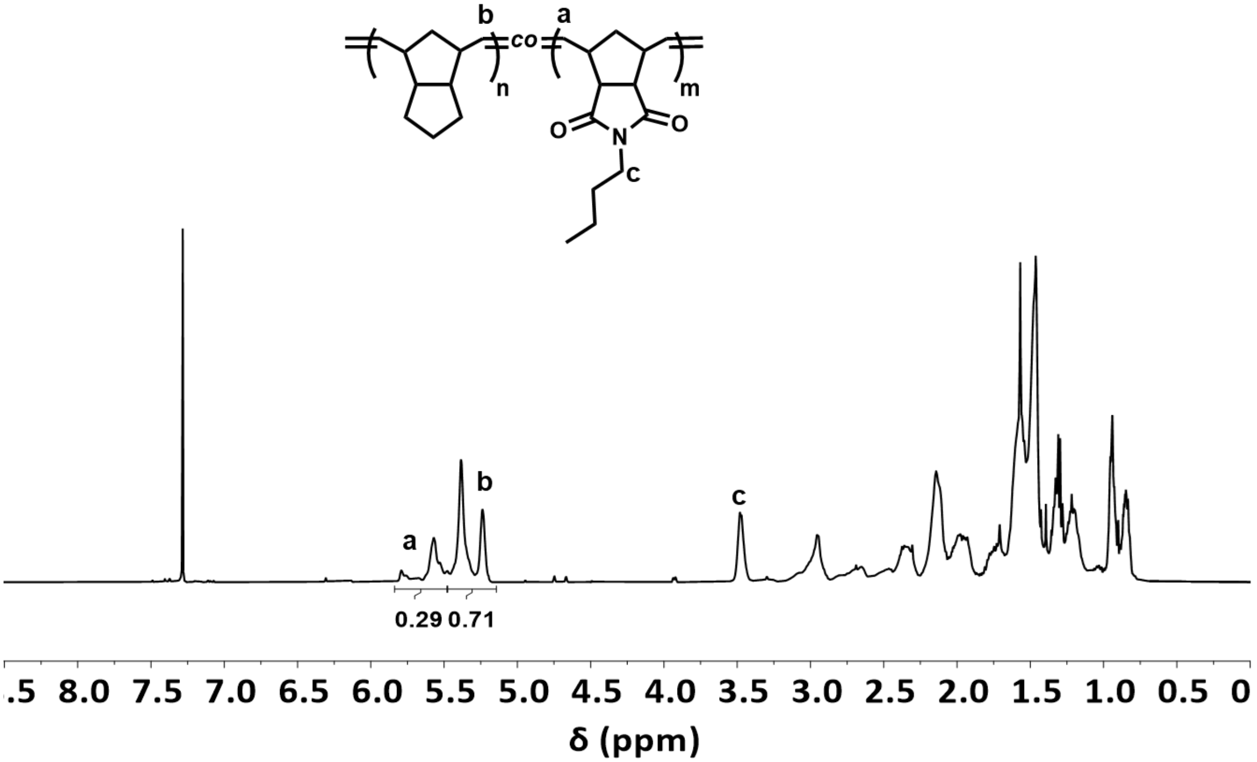

Figure S267: Representative NMR of 25 mol% NBI_4 post-FROMP for 4000:1:1.

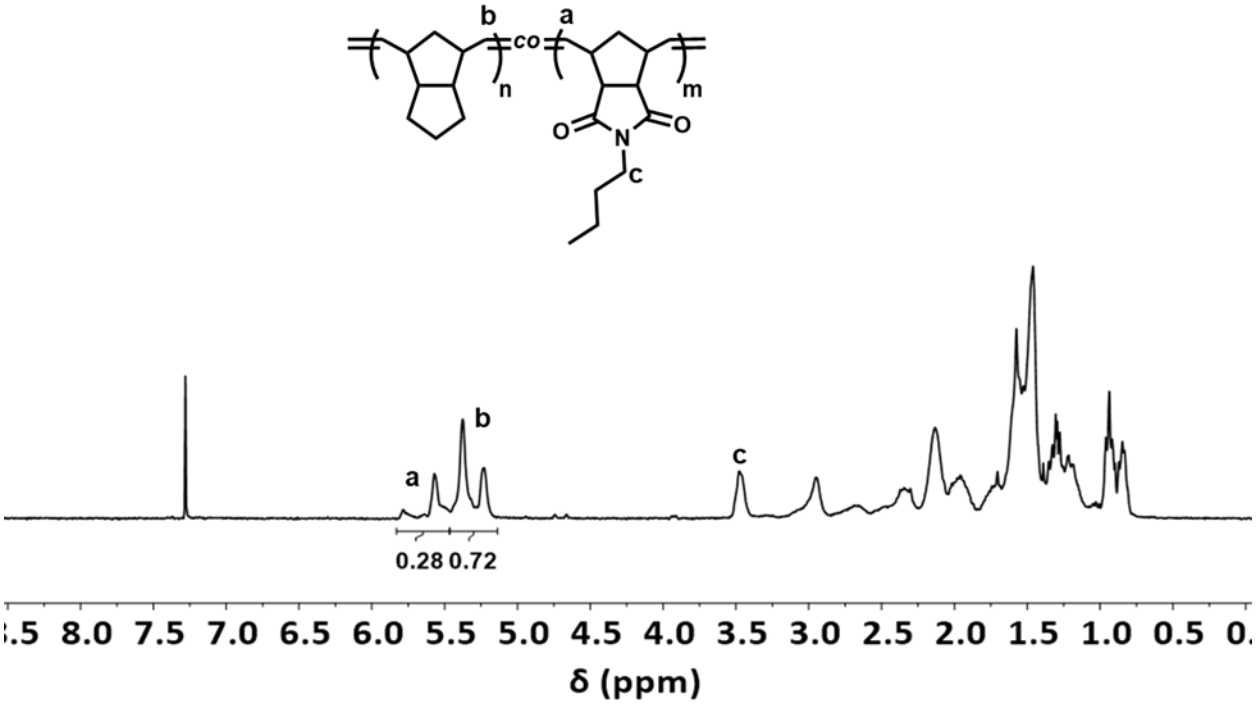

Figure S268: Representative NMR of 25 mol% NBI_4 post-FROMP for 2000:1:1.

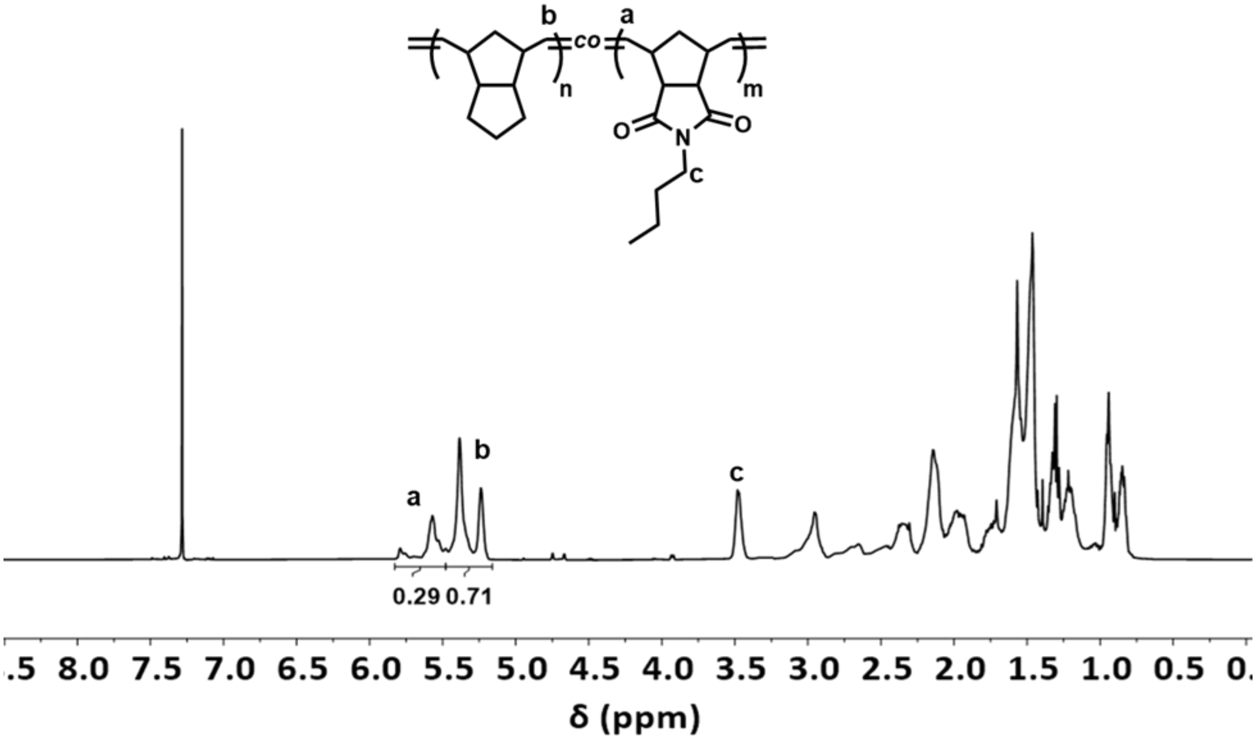

Figure S269: Representative NMR of 25 mol% NBI_4 post-FROMP for 1000:1:1.

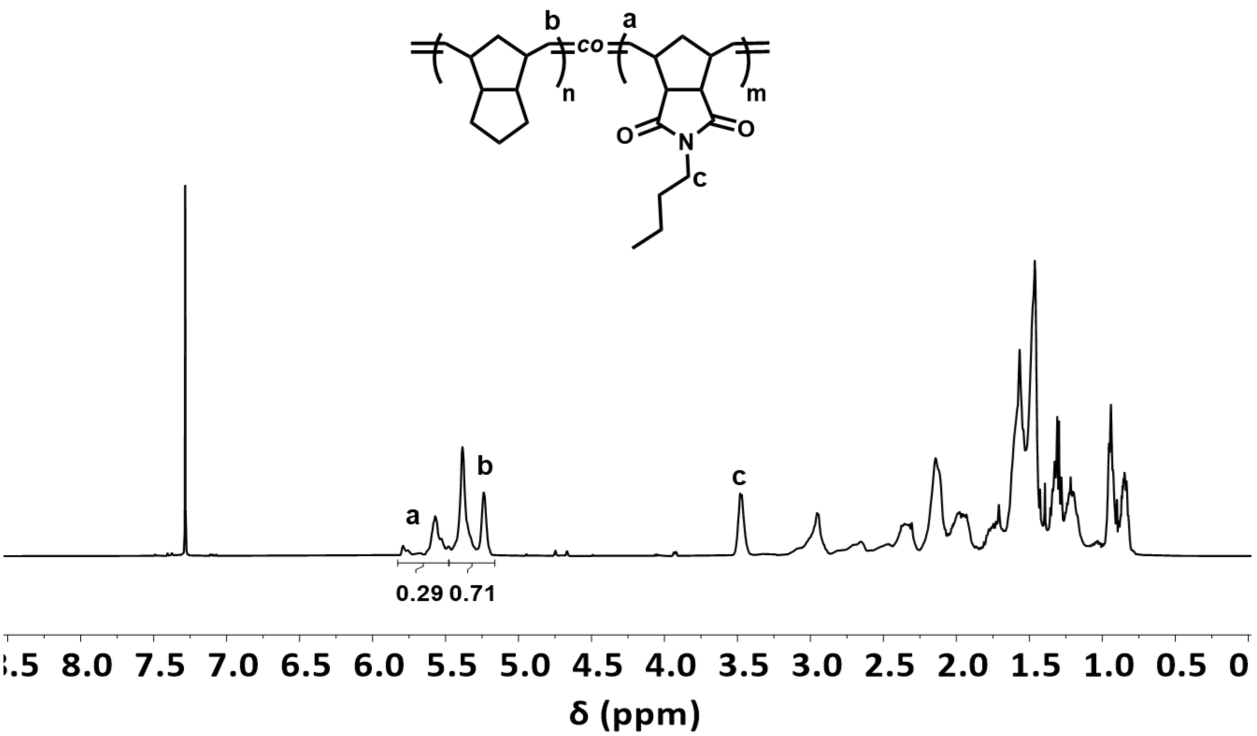

Figure S270: Representative NMR of 25 mol% NBI_4 post-FROMP for 500:1:1.

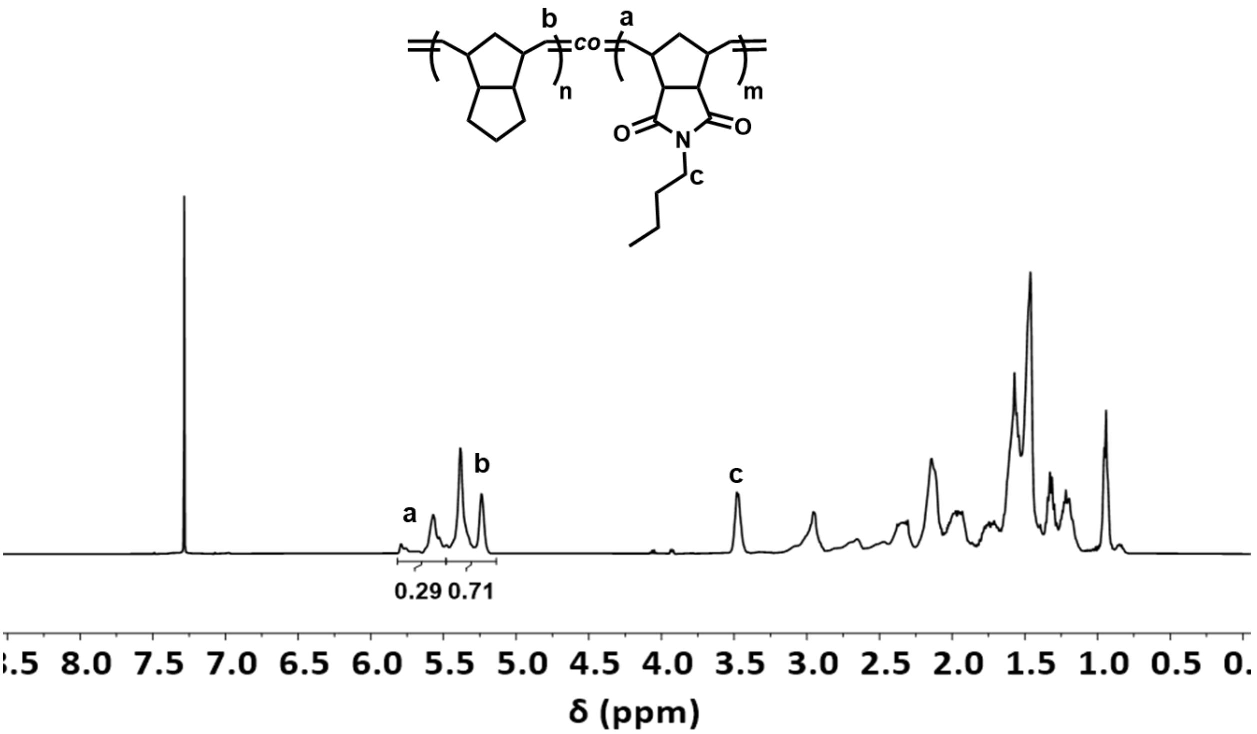

Figure S271: Representative NMR of 25 mol% NBI_4 post-FROMP for 200:1:1.

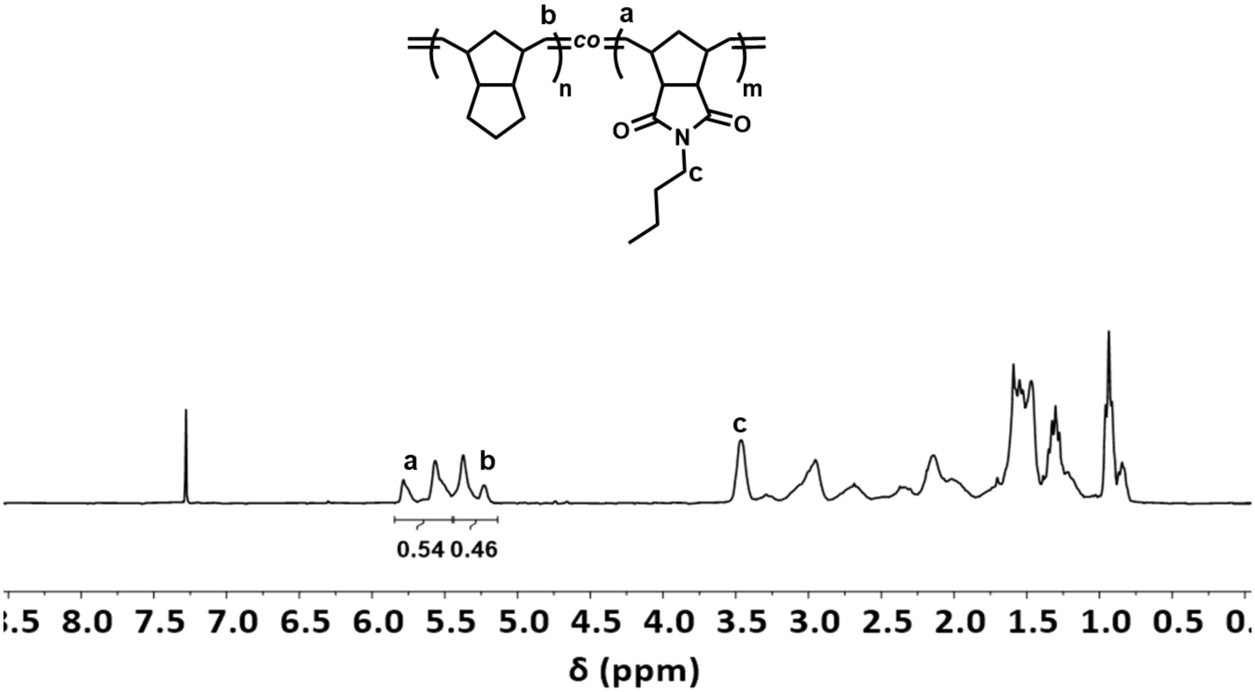

Figure S272: Representative NMR of 50 mol% NBI_4 post-FROMP for 4000:1:1.

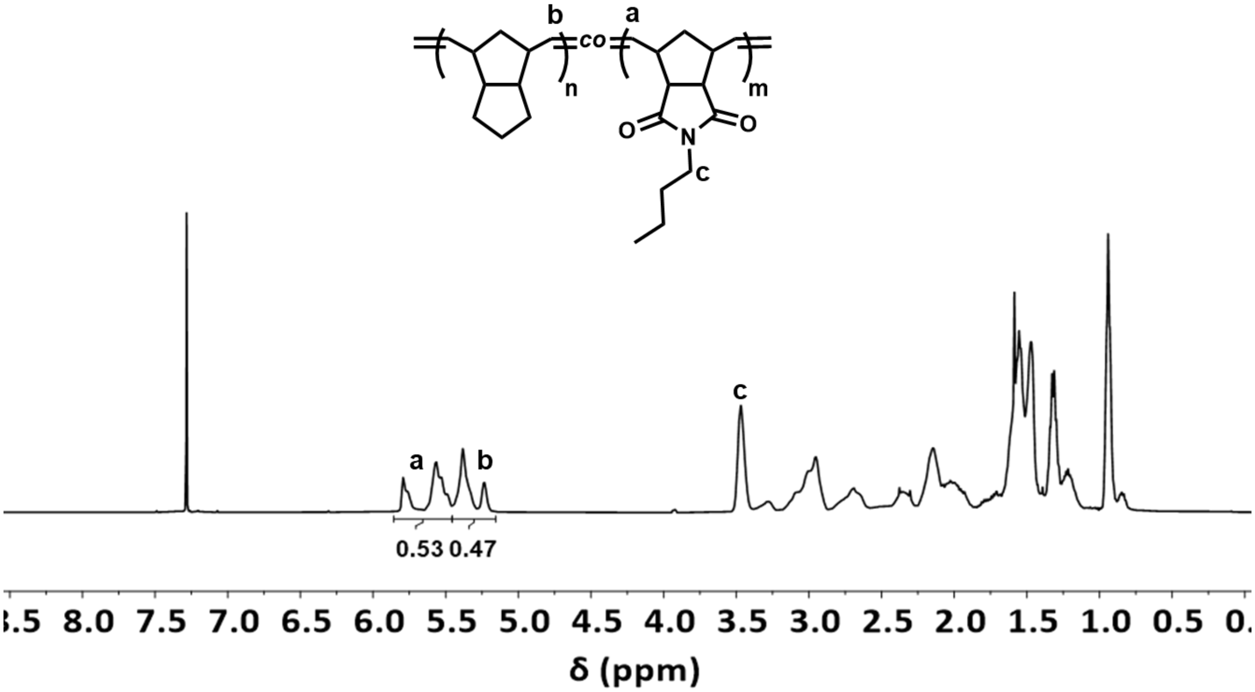

Figure S273: Representative NMR of 50 mol% NBI_4 post-FROMP for 2000:1:1.

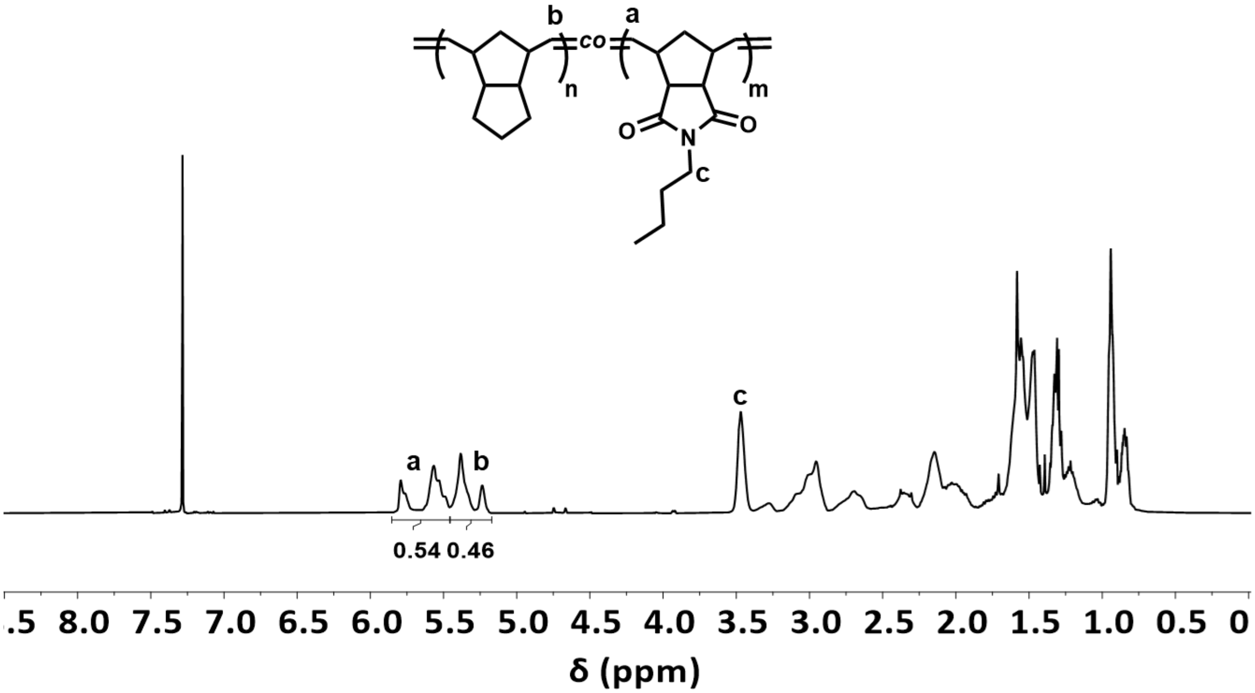

Figure S274: Representative NMR of 50 mol% NBI_4 post-FROMP for 1000:1:1.

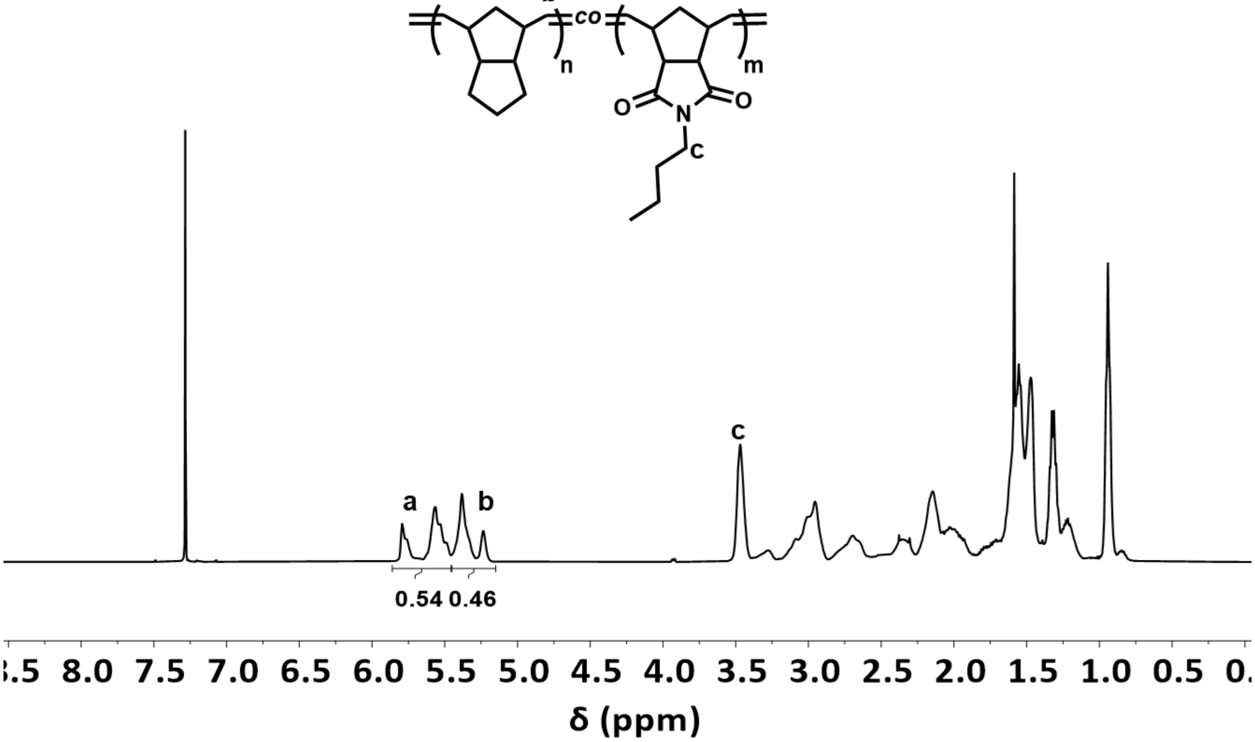

Figure S275: Representative NMR of 50 mol% NBI_4 post-FROMP for 1000:1:1 **bottom up**.

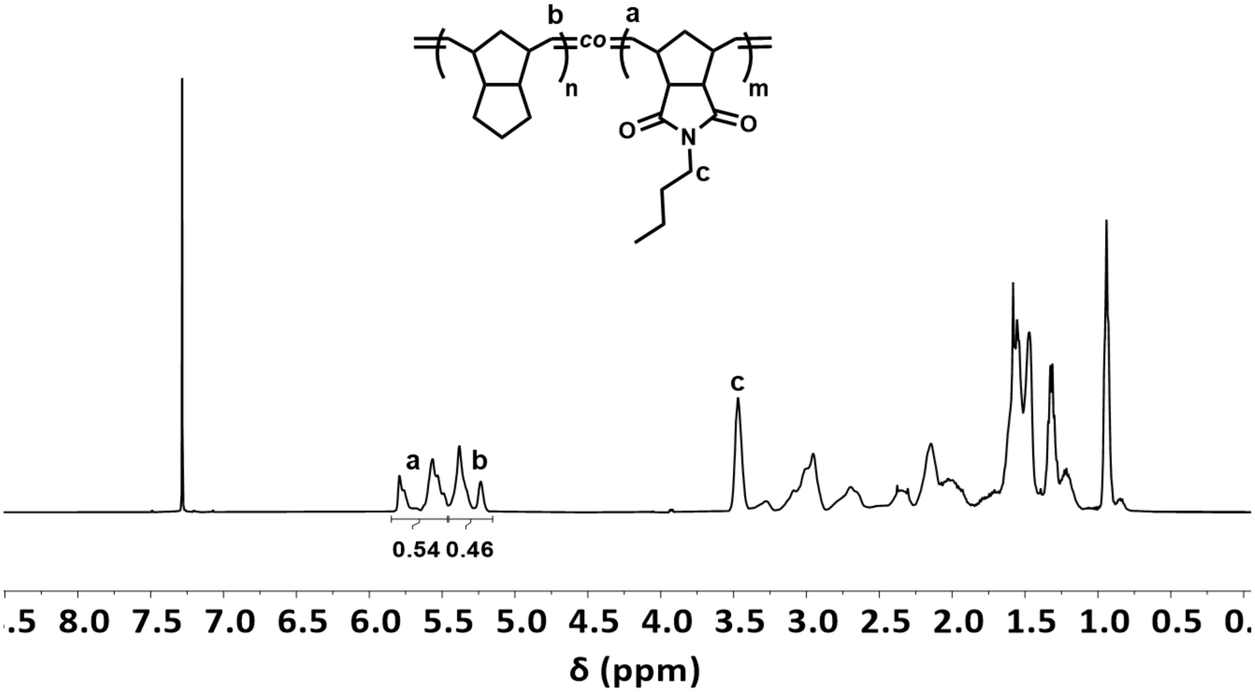

Figure S276: Representative NMR of 50 mol% NBI_4 post-FROMP for 500:1:1.

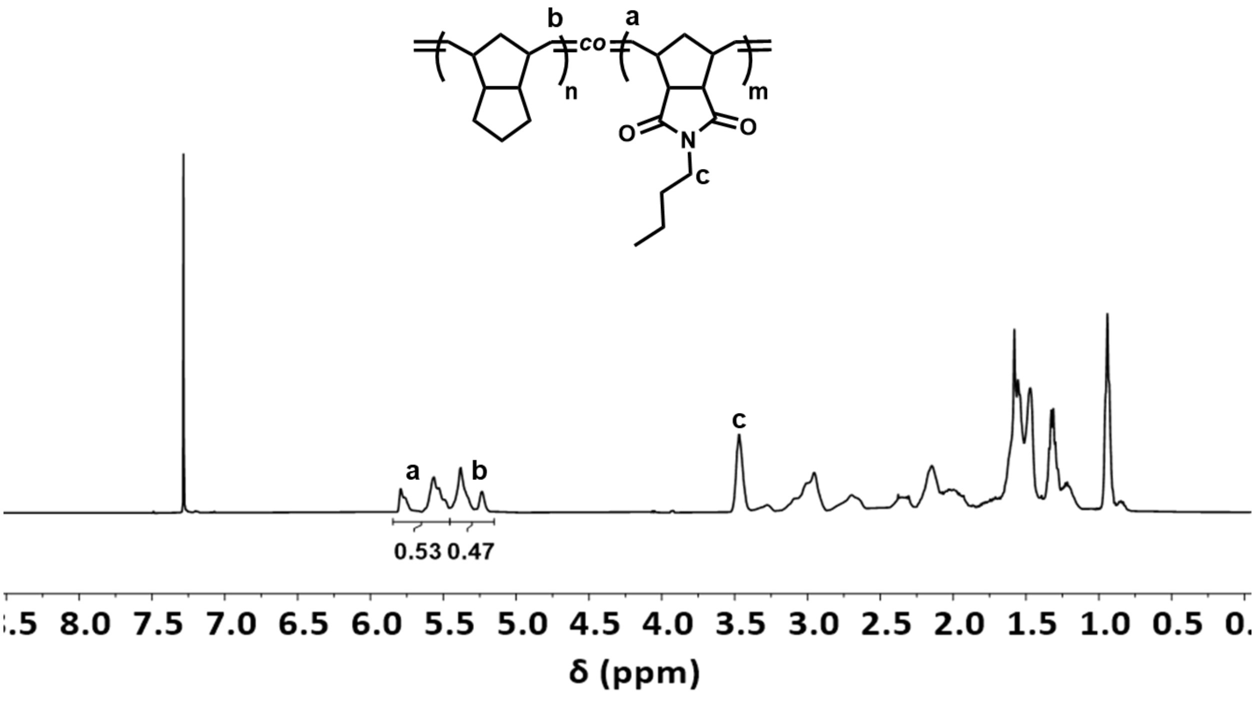

Figure S277: Representative NMR of 50 mol% NBI_4 post-FROMP for 200:1:1.

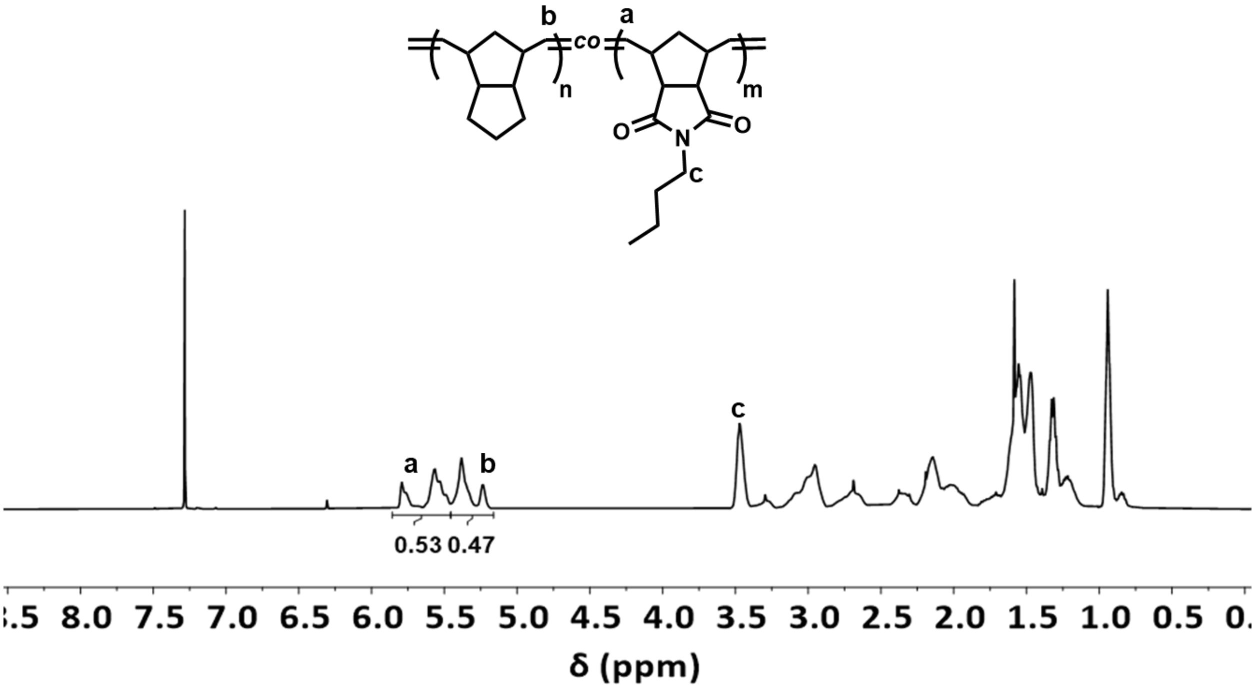

Figure S278: Representative NMR of 50 mol% NBI_4 post-FROMP for 4000:1:2.

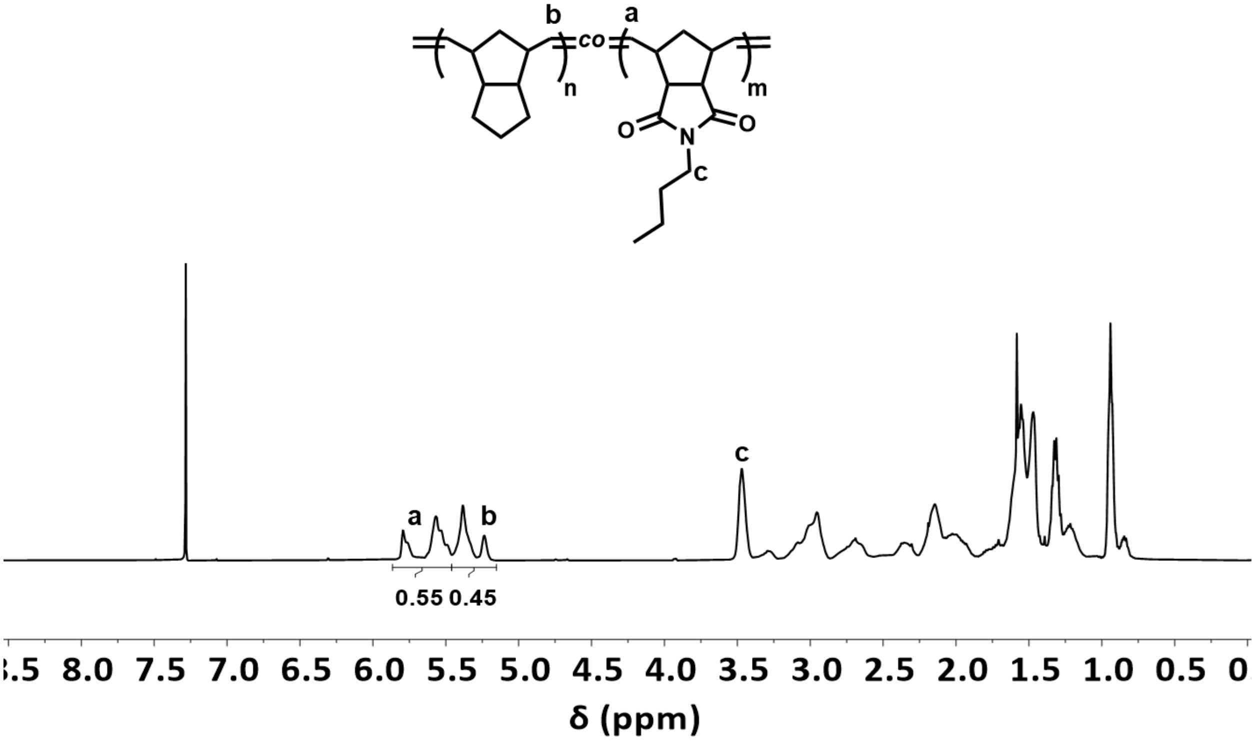

Figure S279: Representative NMR of 50 mol% NBI_4 post-FROMP for 4000:1:2 **bottom up**.

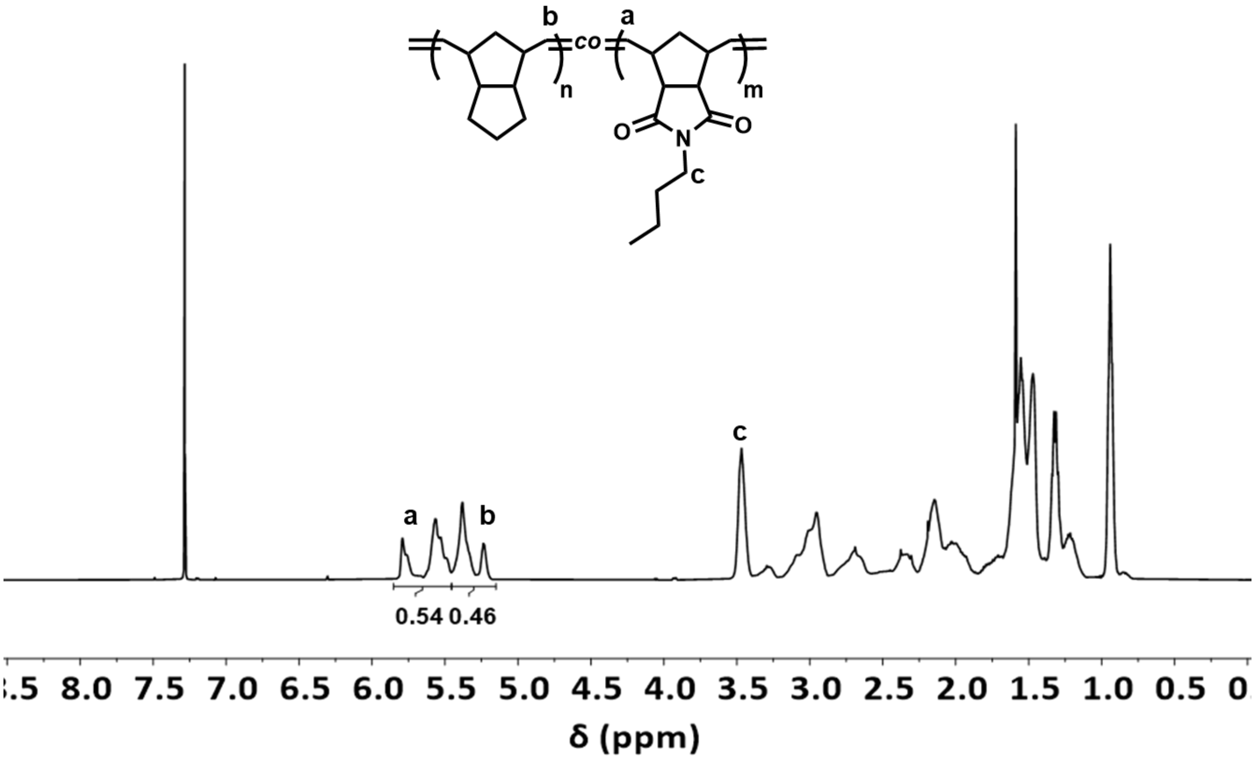

Figure S280: Representative NMR of 50 mol% NBI_4 post-FROMP for 1000:1:2.

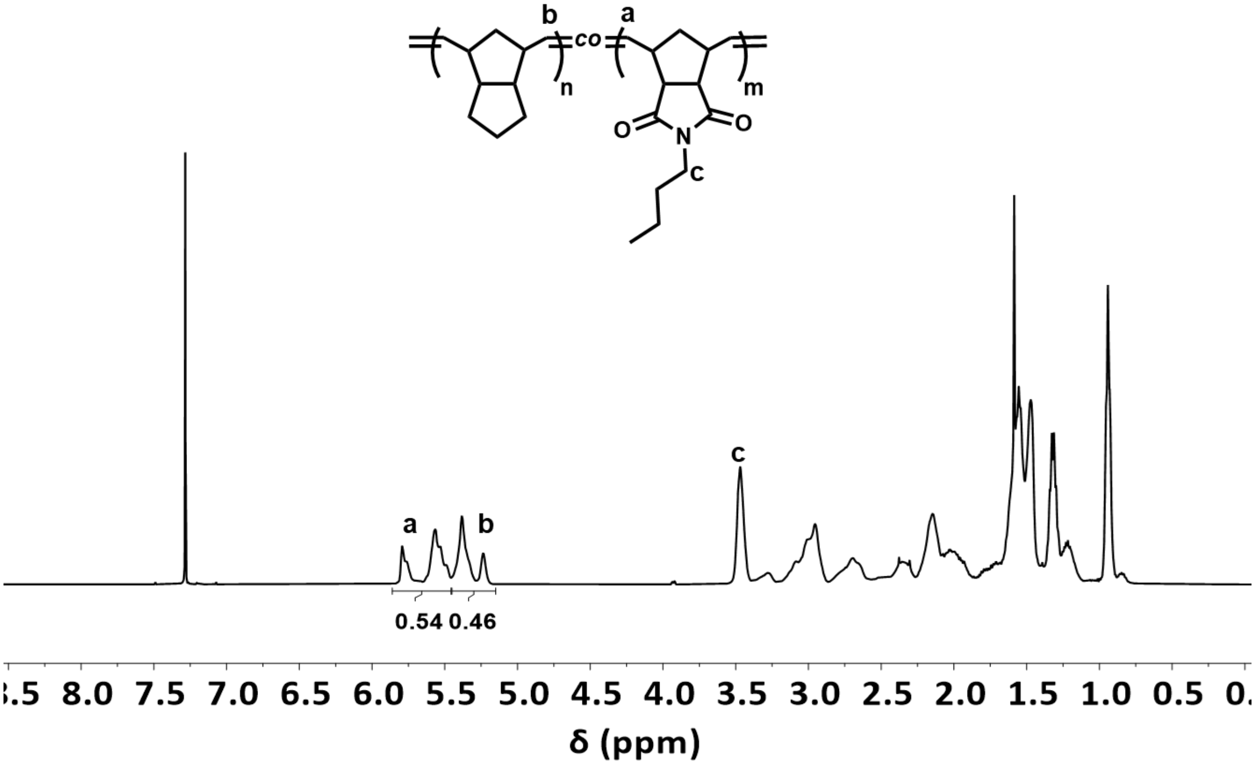

Figure S281: Representative NMR of 50 mol% NBI_4 post-FROMP for 1000:1:2 **bottom up**.

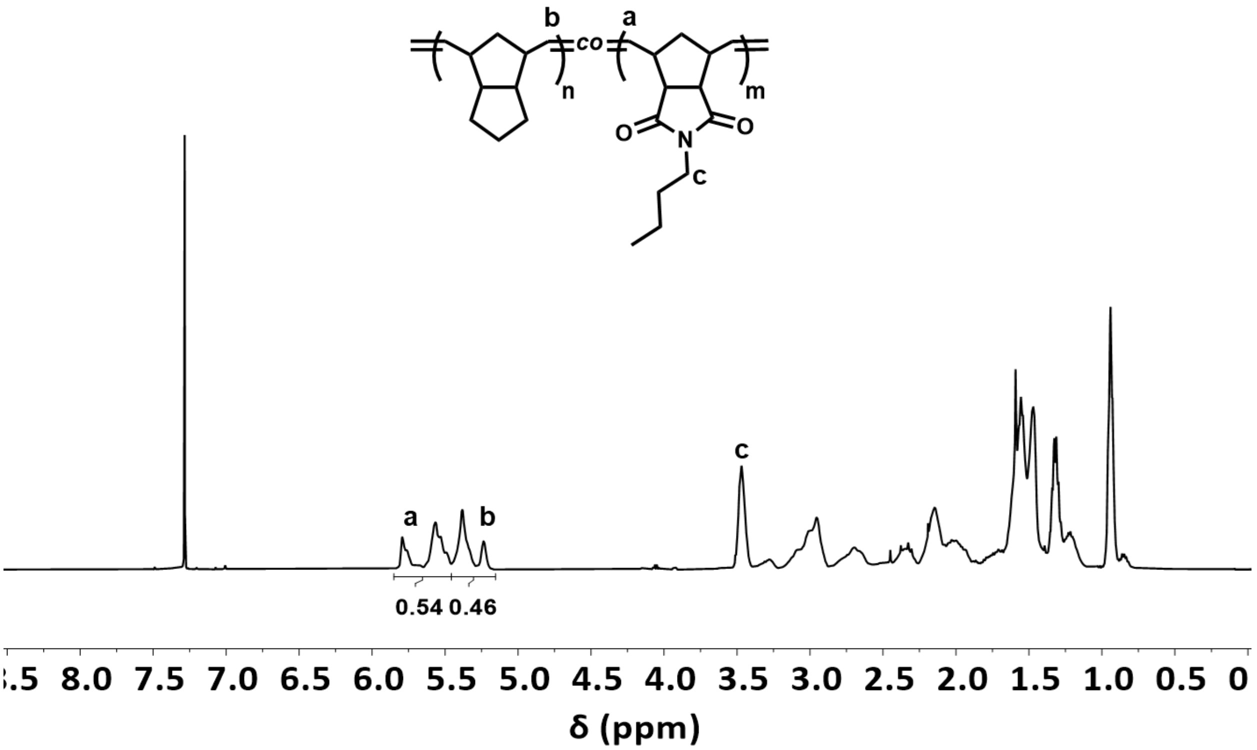

Figure S282: Representative NMR of 50 mol% NBI_4 post-FROMP for 200:1:2.

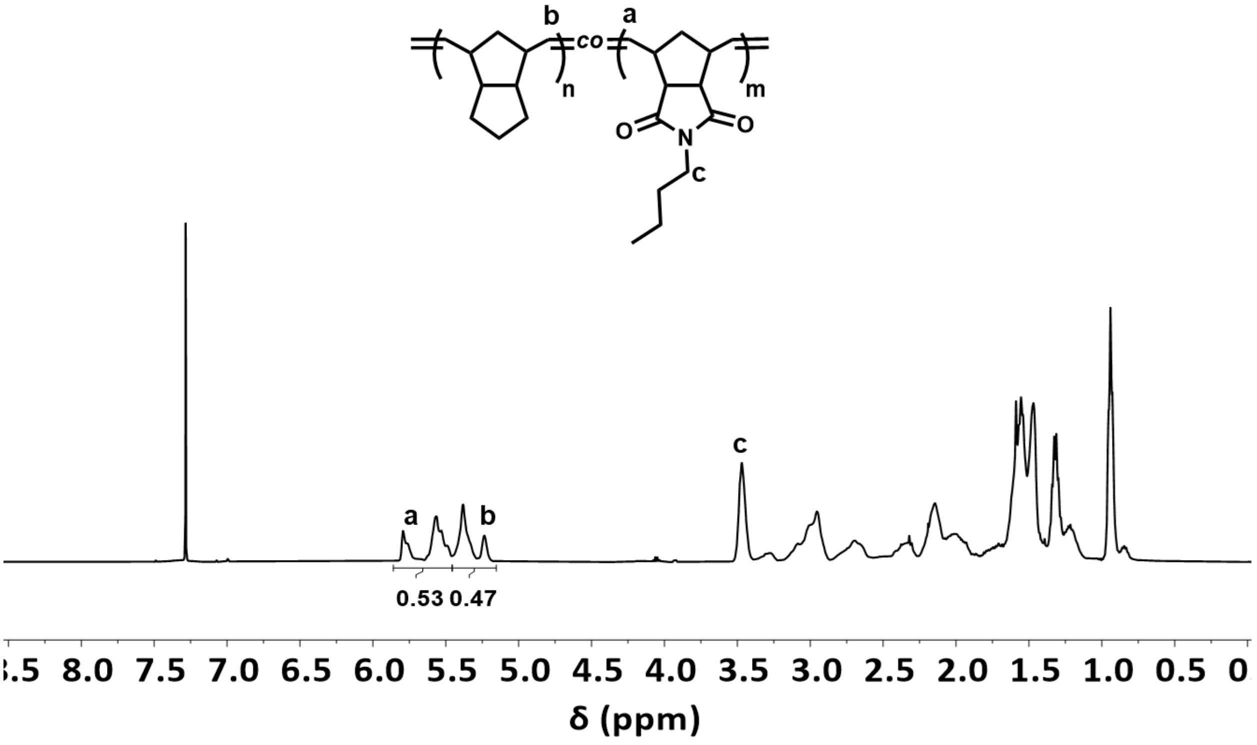

Figure S283: Representative NMR of 50 mol% NBI_4 post-FROMP for 200:1:2 **bottom up**.

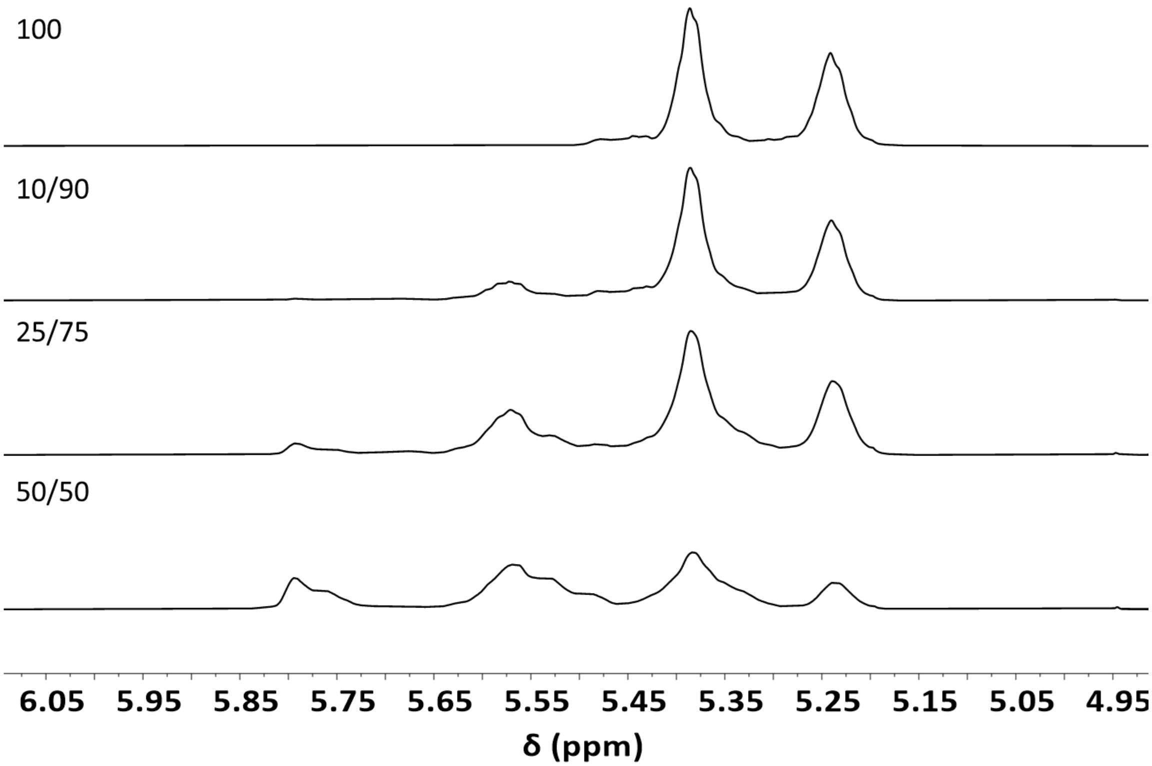

Figure S284: Representative NMR of resulting monomers at each monomer loading post-FROMP for 1000:1:1 monomer:initiator:inhibitor suggesting statistical copolymerization and random monomer distribution due to peak broadening.

Table S16: NMR integrations (n = 3) for varying mol% NBI_4 post-FROMP at varying loadings.

Mixture	Monomer (equiv)	Initiator (equiv)	Inhibitor (equiv)	Direction	NBI_4 Content (%)	Error (%)
90:10	*Pre-polymerization Monomer Mixture*				*12*	--
90:10	4000	1	1	downward	13	1
90:10	2000	1	1	downward	13	1
90:10	1000	1	1	downward	13	1
90:10	500	1	1	downward	13	1
90:10	200	1	1	downward	12	1
75:25	*Pre-polymerization Monomer Mixture*				*28*	--
75:25	4000	1	1	downward	29	1
75:25	2000	1	1	downward	28	1
75:25	1000	1	1	downward	29	0
75:25	500	1	1	downward	29	1
75:25	200	1	1	downward	30	1
50:50	*Pre-polymerization Monomer Mixture*				*53*	--
50:50	4000	1	1	downward	54	1
50:50	2000	1	1	downward	54	1
50:50	1000	1	1	downward	55	1
50:50	1000	1	1	upward	54	0
50:50	500	1	1	downward	54	1
50:50	200	1	1	downward	53	1
50:50	4000	1	2	downward	53	1
50:50	4000	1	2	upward	54	1
50:50	1000	1	2	downward	53	1
50:50	1000	1	2	upward	54	1
50:50	200	1	2	downward	54	1
50:50	200	1	2	upward	53	1

Gradient Polymer Materials

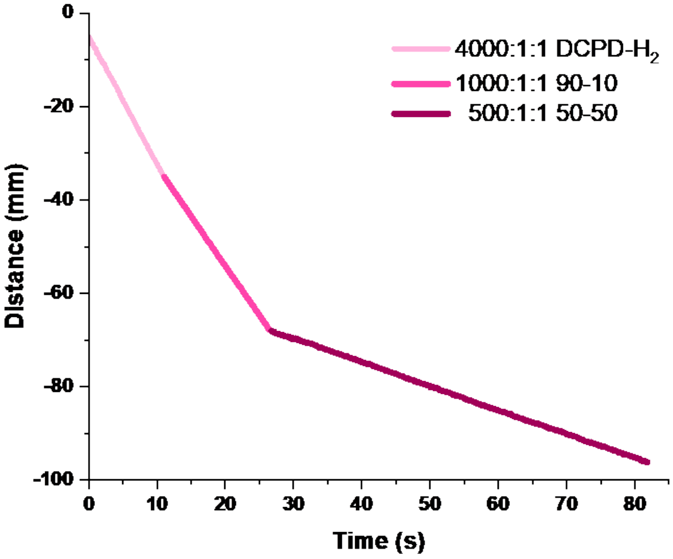

Figure S285: Front velocity of triple stack ($v_{f,\ stack\ 1}$ = 2.7 mm/s, $v_{f,\ stack\ 2}$ = 2.1 mm/s, and $v_{f,\ stack\ 3}$ = 0.51 mm/s).

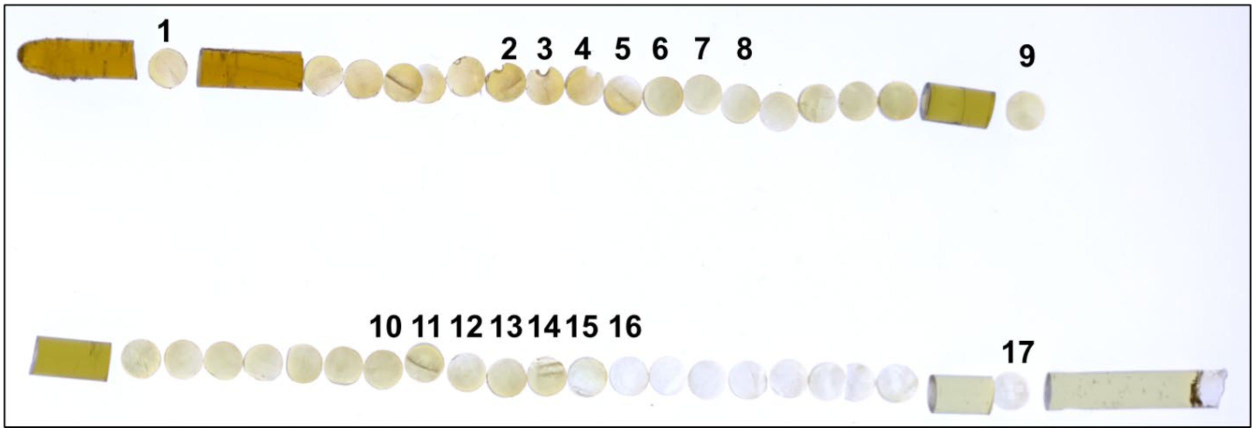

Figure S286: Cutting of triple stacked for SEC analysis.

Table S17: M_n and $Đ$ of each cross section from triple stacked for SEC analysis.

Cross section number	M_n (kg/mol)	$Đ$
**1**	**121**	**1.31**
2	121	1.28
3	121	1.31
4	133	1.41
5	156	1.66
6	157	1.64
7	164	1.6
8	160	1.65
**9**	**166**	**1.65**
10	152	1.62
11	156	1.59
12	165	1.58
13	159	1.62
14	160	1.62
15	315	1.77
16	297	1.8
**17**	**400**	**1.76**

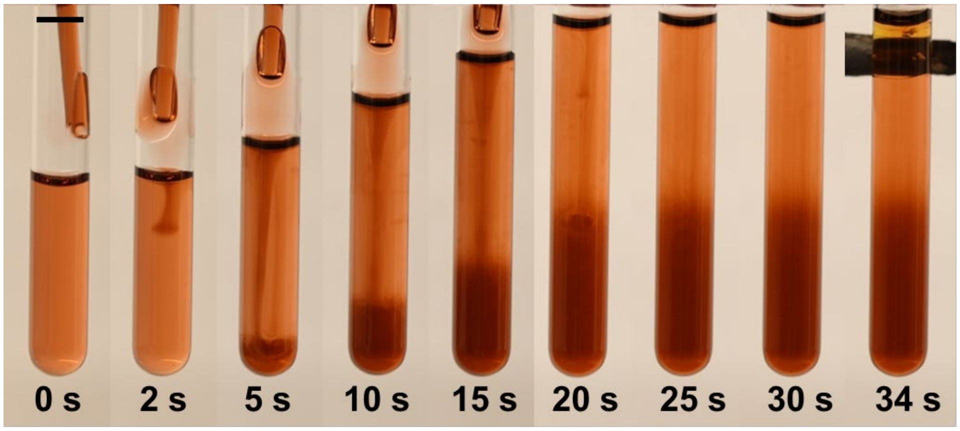

Figure S287: Timelapse of double stack gradient interface formation by adding the denser liquid last promoting mixing (scale bar is 5 mm).

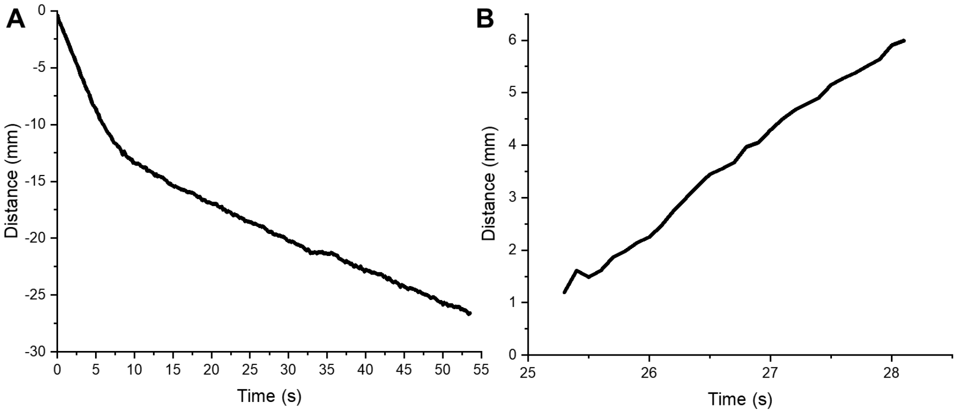

Figure S288: Front velocity of double stack (A) down the tube for both layers and (B) across the tube for the bottom layer ($v_{\text{f, stack 1}}$ = 1.7 mm/s, $v_{\text{f, stack 2}}$ = 0.3 mm/s, and $v_{\text{f, stack 2, across tube}}$ = 1.8 mm/s).

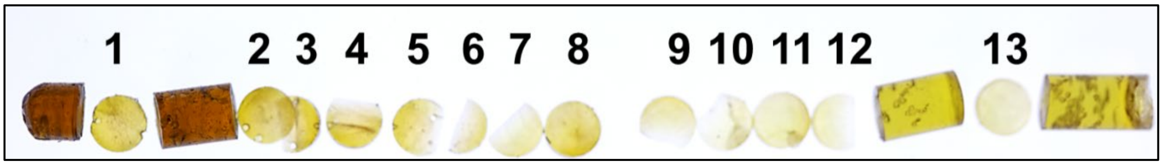

Figure S289: Cross sections of gradient materials from double stacked for SEC analysis.

Table S18: M_n and *Đ* of each cross section from double stacked for SEC analysis.

Cross section number	M_n (kg/mol)	*Đ*
1	57	1.05
2	59	1.08
3	58	1.10
4	58	1.11
5	64	1.10
6	60	1.17
7	81	1.22
8	77	1.16
9	70	1.23
10	97	1.29
11	107	1.31
12	107	1.38
**13**	**111**	**1.55**

Preparation of DMA and Tensile Specimens:

pDCPD-H_2 1000:1:1 monomer:initiator:inhibitor

Grubbs' 2nd generation initiator was massed (G2, 15.81 mg, 1.00 equiv.) in an 8 mL vial prior to the addition of tributyl phosphite (TBP, 5.1 μL, 1.0 equiv.). The mixture was dissolved in monomer (*DCPD-H_2*, 2500 mg, 1000 equiv.) and sonicated for up to 5 minutes. The resulting solution was transferred to a 3 mm gasket U-mold then heated in a 45 °C oven for up to 3 min. Samples were initiated at the top of the resin using a preheated sottering iron creating a descending front.

50 mol% NBI_4 - 1000:1:1 monomer:initiator:inhibitor

Grubbs' 2nd generation initiator was massed (G2, 13.45 mg, 1.00 equiv.) in an 8 mL vial prior to the addition of tributyl phosphite (TBP, 4.3 μL, 1.0 equiv.). The mixture was dissolved in monomer (*DCPD-H_2 & NBI_4*, 2800 mg, 1000 equiv.) and sonicated for up to 5 minutes. The resulting solution was transferred to a 3 mm gasket U-mold then heated in a 45 °C oven for up to 3 min. Samples were initiated at the top of the resin using a preheated sottering iron creating a descending front.

Gradient Mixtures - 1000:1:1 monomer:initiator:inhibitor

Grubbs' 2nd generation initiator was massed (G2, 8.22 mg, 1.00 equiv.) in an 8 mL vial prior to the addition of tributyl phosphite (TBP, 2.6 μL, 1.0 equiv.). The mixture was dissolved in monomer (*DCPD-H_2*, 1300 mg, 1000 equiv.) and sonicated for up to 5 minutes. In a separate vial, Grubbs' 2nd generation initiator was massed (G2, 6.24 mg, 1.00 equiv.) in an 8 mL vial prior to the addition of tributyl phosphite (TBP, 2.0 μL, 1.0 equiv.). The mixture was dissolved in monomer (*DCPD-H_2 & NBI_4*, 1300 mg, 1000 equiv.) and sonicated for up to 5 minutes. The DCPD-H_2 resin was transferred to a 3 mm gasket U-mold prior to the 50 mol% resin. The resulting gradient resin was then heated in a 45 °C oven for up to 3 min. Samples were initiated at the top of the resin using a preheated sottering iron creating a descending front.

Sharp Mixtures - 1000:1:1 monomer:initiator:inhibitor

Grubbs' 2nd generation initiator was massed (G2, 8.22 mg, 1.00 equiv.) in an 8 mL vial prior to the addition of tributyl phosphite (TBP, 2.6 μL, 1.0 equiv.). The mixture was dissolved in monomer (*DCPD-H_2*, 1300 mg, 1000 equiv.) and sonicated for up to 5 minutes. In a separate vial, Grubbs' 2nd generation initiator was massed (G2, 6.24 mg, 1.00 equiv.) in an 8 mL vial prior to the addition of tributyl phosphite (TBP, 2.0 μL, 1.0 equiv.). The mixture was dissolved in monomer (*DCPD-H_2 & NBI_4*, 1300 mg, 1000 equiv.) and sonicated for up to 5 minutes. The 50 mol% resin was transferred to a 3 mm gasket U-mold prior to the DCPD-H_2 resin. The resulting gradient resin was then heated in a 45 °C oven for up to 3 min. Samples were initiated at the top of the resin using a preheated sottering iron creating a descending front.

Dynamic Mechanical Analysis (DMA):

DMA samples were prepared in a U-type mold (3.0 mm thickness) at 45 °C initial temperature as shown in literature.[1]

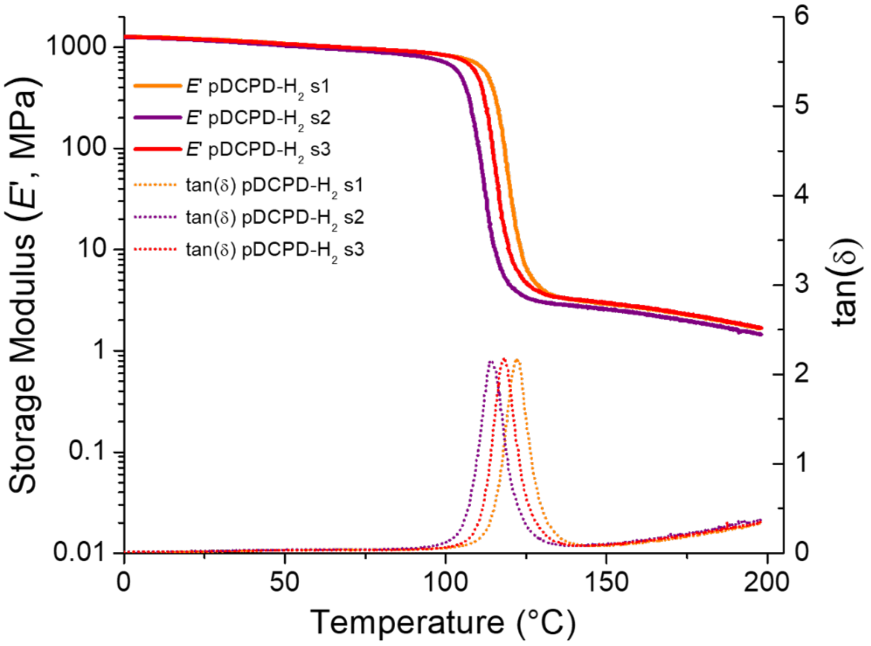

Figure S290: Triplicate DMA temperature ramp of 1000:1:1 monomer:initiator:inhibitor for pDCPD-H_2 post-FROMP.

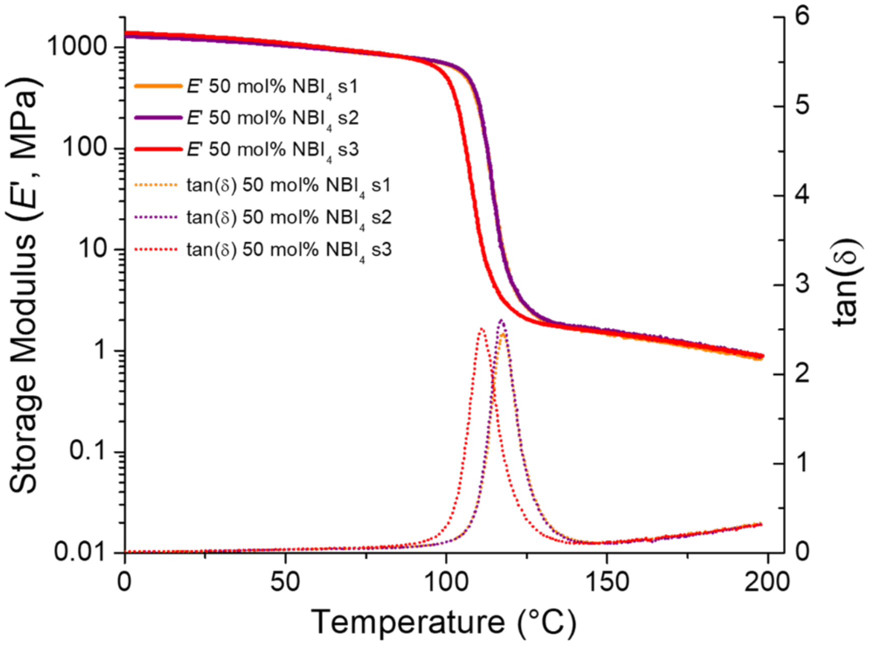

Figure S291: Triplicate DMA temperature ramp of 1000:1:1 monomer:initiator:inhibitor for 50 mol% NBI_4 post-FROMP.

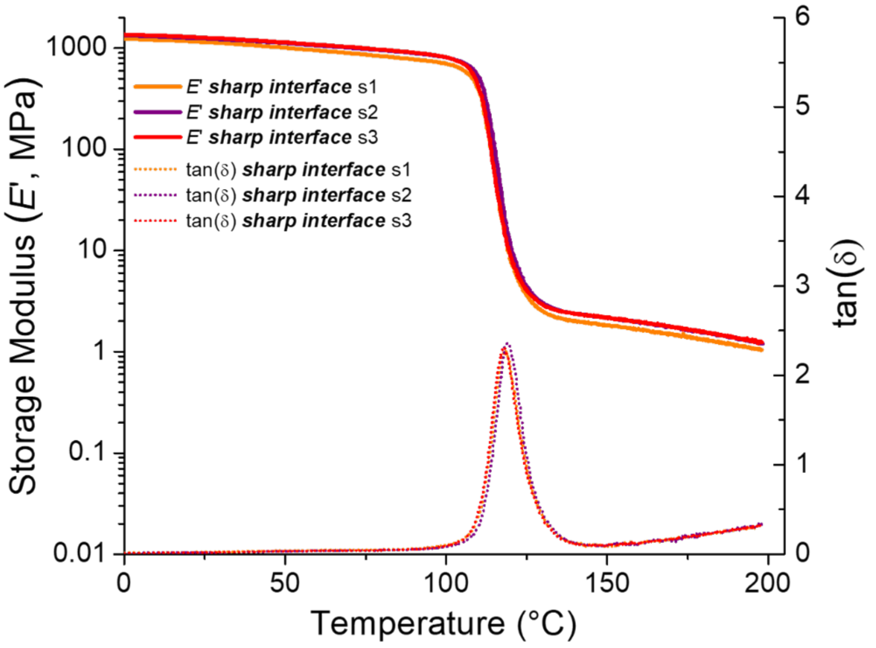

Figure S292: Triplicate DMA temperature ramp of 1000:1:1 monomer:initiator:inhibitor for pDCPD-H_2 and 50 mol% NBI_4 **sharp interface** post-FROMP.

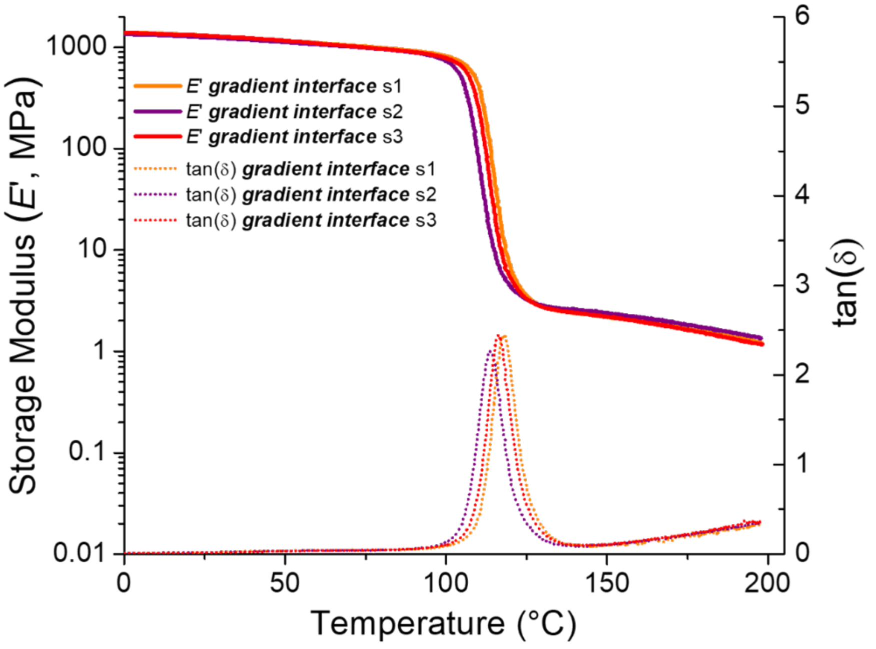

Figure S293: Triplicate DMA temperature ramp of 1000:1:1 monomer:initiator:inhibitor for pDCPD-H_2 and 50 mol% NBI_4 **gradient interface** post-FROMP.

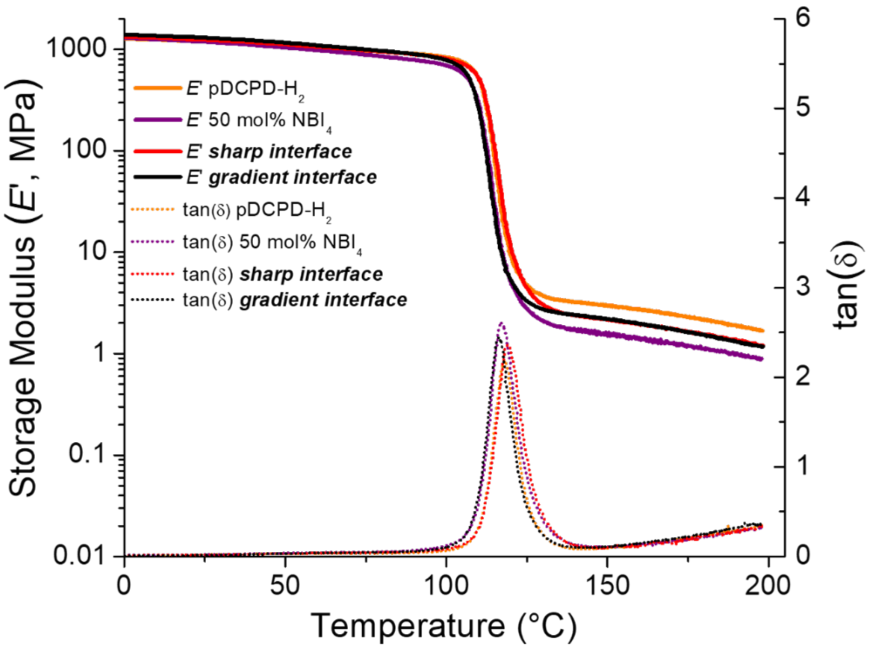

Figure S294: Overlay of DMA temperature ramps of 1000:1:1 monomer:initiator:inhibitor for pDCPD-H_2, 50 mol% NBI_4, **gradient interface**, and **sharp interface** materials post-FROMP.

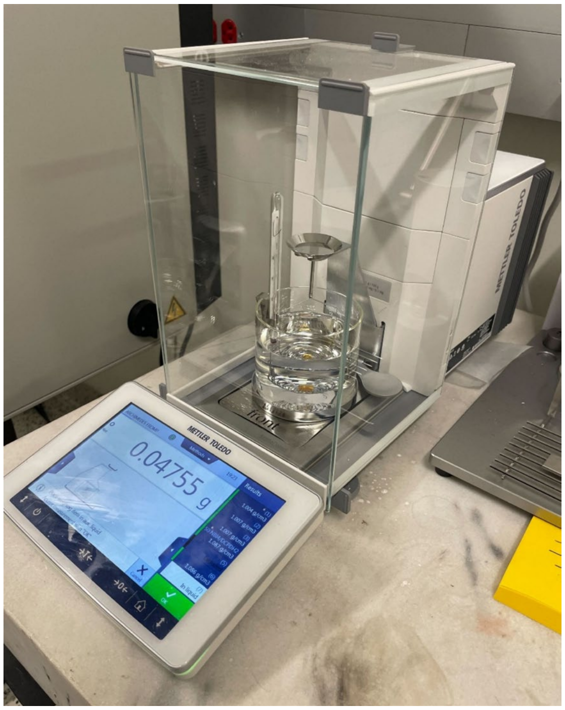

Figure S295: Setup for density calculation for polymer materials.

Table S19: Density determination (n = 3) for 0 and 50 mol% NBI_4 post-FROMP at 1000:1:1 monomer:initiator:inhibitor.

Sample	Loading	Density (g/mL)				
		1	2	3	**average**	**err**
pDCPD-H_2	1000:1:1	1.004	1.007	1.007	**1.006**	**0.001**
50 mol% NBI_4	1000:1:1	1.087	1.086	1.085	**1.086**	**0.001**

The molecular weight between entanglements (M_e) was calculated using the tensile storage modulus (E') from the rubbery plateau.

$$G' = (E')/(2(1+v)) = \rho RT/M_e \qquad \text{[Equation S1]}^{2,3}$$

$$M_e = (2.8(\rho RT))/(E')$$

Using the density of DCPD-H_2 (ρ = 1006 kg/m^3), 50 mol% NBI_4 (ρ = 1086 kg/m^3) or assumed density for gradient/sharp interface (ρ = 1046 kg/m^3) polymer materials, Poisson's ratio of pDCPD (v = 0.4), the ideal gas constant (R = 8.314 J/mol*K), and the storage modulus at the 136 °C, M_e was calculated using Equation S1.

Table S20: T_g, glassy modulus (storage modulus below T_g), and M_e determination (n = 3) for 0 (monomer MW_{ave} = 134.22 g/mol), 50 mol% NBI_4 (monomer MW_{ave} = 176.75 g/mol), and gradient/sharp interface (monomer MW_{ave} = 155.49 g/mol) materials post-FROMP at 1000:1:1 monomer:initiator:inhibitor.

Sample	Loading	$T_{g, DMA}$ (°C)				
		S1	S2	S3	**average**	**error**
pDCPD-H_2	1000:1:1	122.0	114.2	118.2	**118.2**	**3.2**
50 mol% NBI_4	1000:1:1	117.7	116.8	111.4	**115.3**	**2.8**
Sharp Interface	1000:1:1	118.6	119.1	118.0	**118.6**	**0.5**
Gradient Interface	1000:1:1	118.4	113.5	116.1	**116.0**	**2.0**
Sample	Loading	**Storage Modulus (*E*', MPa) at 25 °C**				
		S1	S2	S3	**average**	**Error**
pDCPD-H_2	1000:1:1	1203	1169	1195	**1189**	**15**
50 mol% NBI_4	1000:1:1	1203	1189	1283	**1225**	**41**
Sharp Interface	1000:1:1	1146	1245	1271	**1221**	**54**
Gradient Interface	1000:1:1	1308	1259	1304	**1290**	**22**
Sample	Loading	**Storage Modulus (*E*', MPa) at 150 °C**				
		S1	S2	S3	**average**	**error**
pDCPD-H_2	1000:1:1	2.921	2.599	2.957	**2.826**	**0.161**
50 mol% NBI_4	1000:1:1	1.456	1.506	1.505	**1.489**	**0.023**
Sharp Interface	1000:1:1	1.813	2.148	2.140	**2.034**	**0.156**
Gradient Interface	1000:1:1	2.173	2.391	2.204	**2.256**	**0.096**
Sample	Loading	**M_e (kg/mol)**				
		S1	S2	S3	**average**	**error**
pDCPD-H_2	1000:1:1	3.39	3.81	3.35	**3.52**	**0.21**
50 mol% NBI_4	1000:1:1	7.35	7.10	7.11	**7.19**	**0.12**
Sharp Interface	1000:1:1	5.68	4.80	4.81	**5.10**	**0.41**
Gradient Interface	1000:1:1	4.74	4.31	4.68	**4.58**	**0.19**
Sample	Loading	**DP between crosslinks**				
		S1	S2	S3	**average**	**error**
pDCPD-H_2	1000:1:1	25	28	25	**26**	**2**
50 mol% NBI_4	1000:1:1	42	40	40	**41**	**1**
Sharp Interface	1000:1:1	37	31	31	**33**	**3**
Gradient Interface	1000:1:1	30	28	30	**29**	**1**

Tensile Testing:

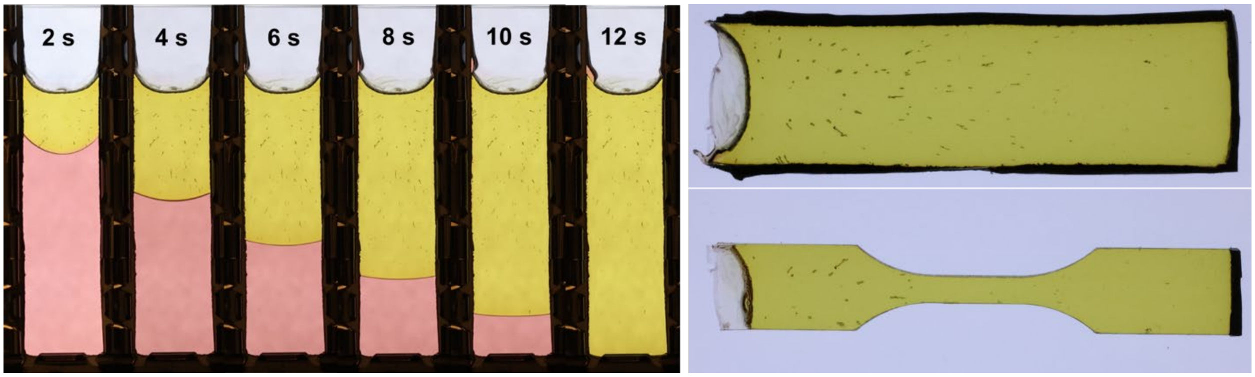

Figure S296: Representative front timelapse and images of 1000:1:1 DCPD-H_2:G2:TBP molar ratio material post-FROMP prepared at 45 °C.

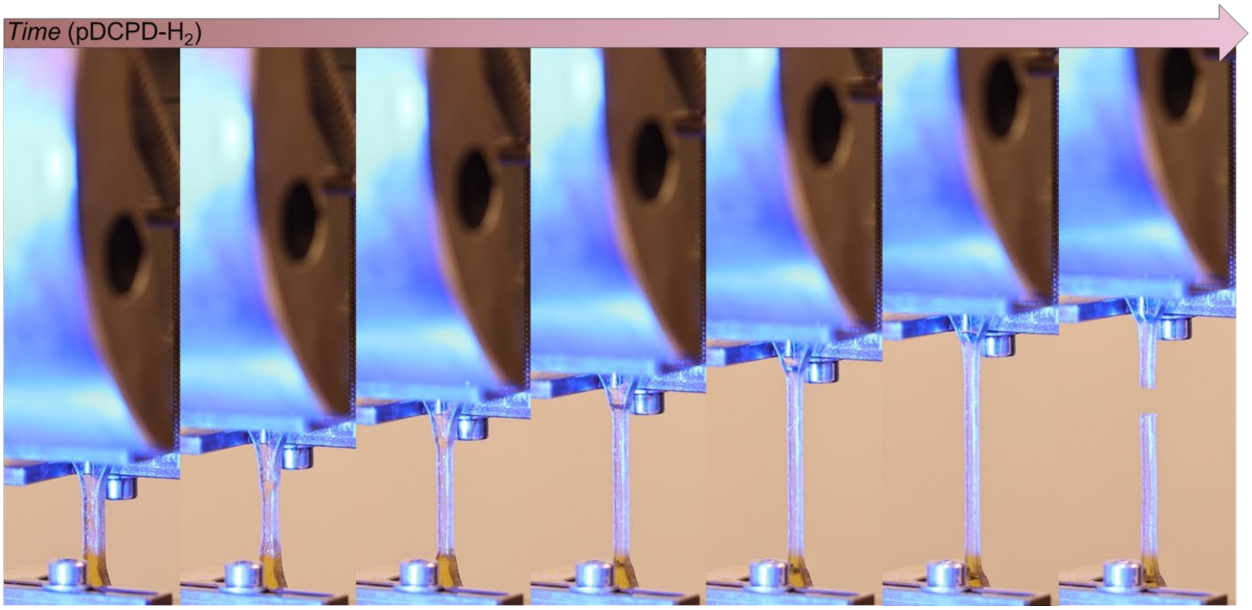

Figure S297: Representative tensile timelapse of 1000:1:1 DCPD-H_2:G2:TBP molar ratio.

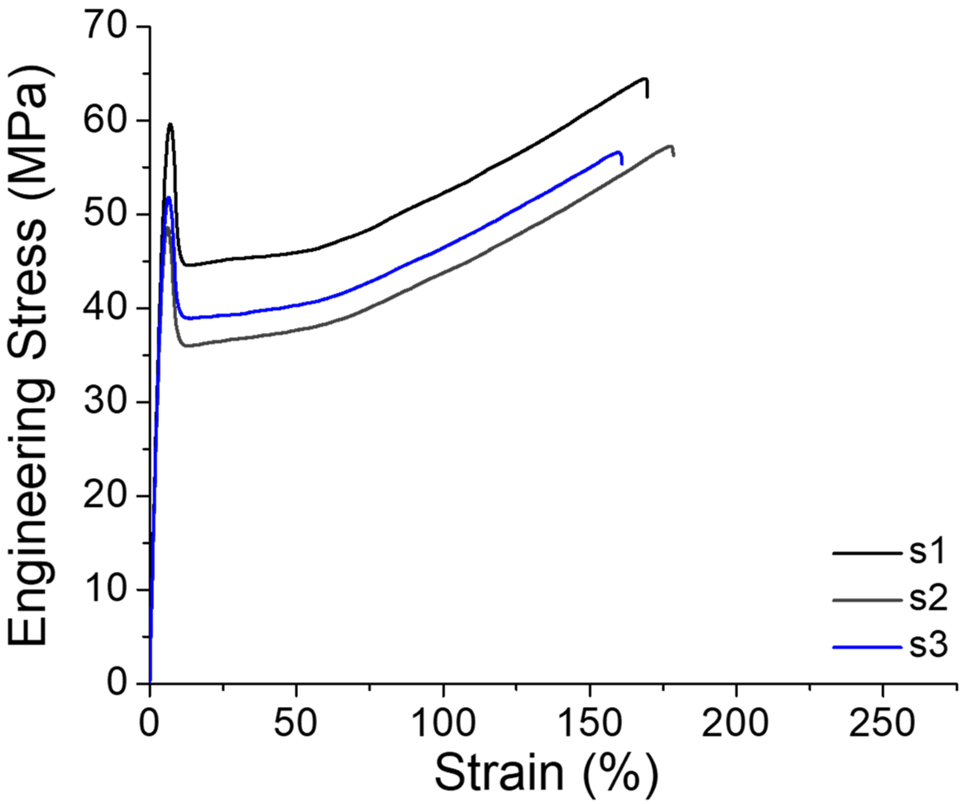

Figure S298: Stress-strain curves of 1000:1:1 DCPD-H_2:G2:TBP molar ratio (n = 3).

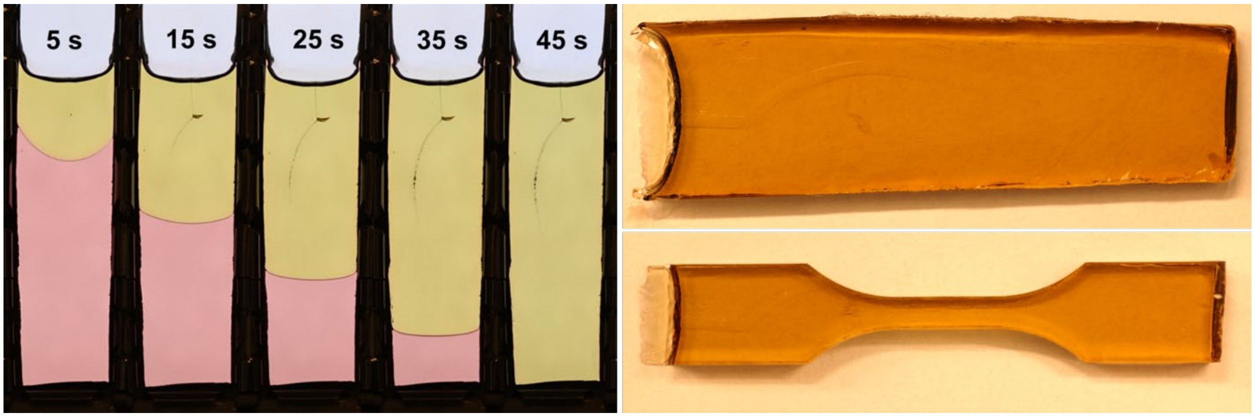

Figure S299: Representative front timelapse and images of 50 mol% NBI4 in DCPD at 1000:1:1 monomer:G2:TBP molar ratio material post-FROMP at 45 °C.

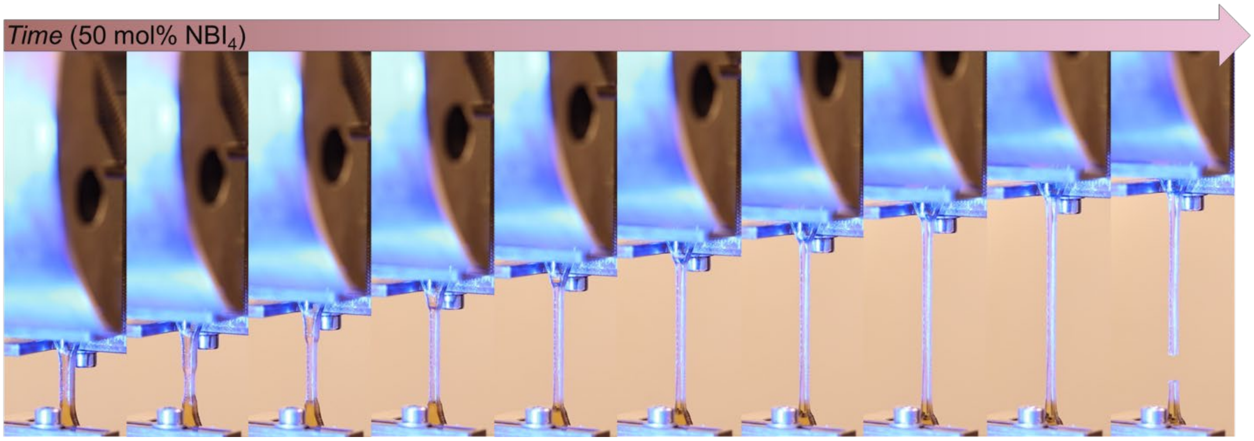

Figure S300: Representative tensile timelapse of 50 mol% NBI4 in DCPD at 1000:1:1 monomer:G2:TBP molar ratio.

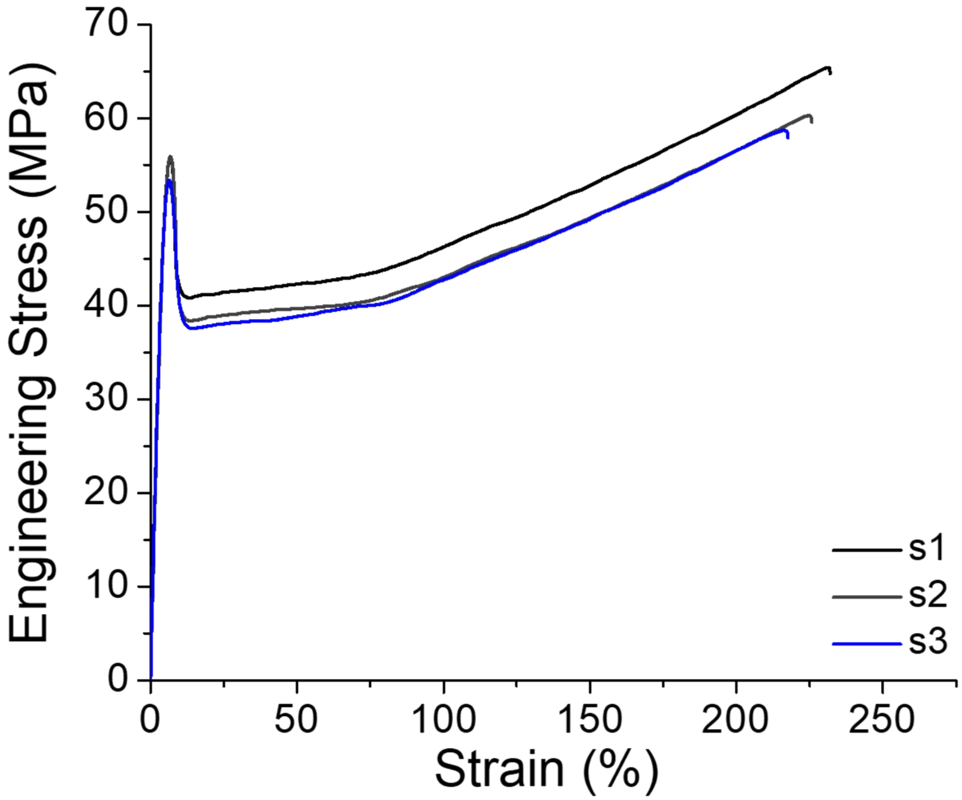

Figure S301: Stress-strain curves of 50 mol% NBI4 in DCPD at 1000:1:1 monomer:G2:TBP molar ratio (n = 3).

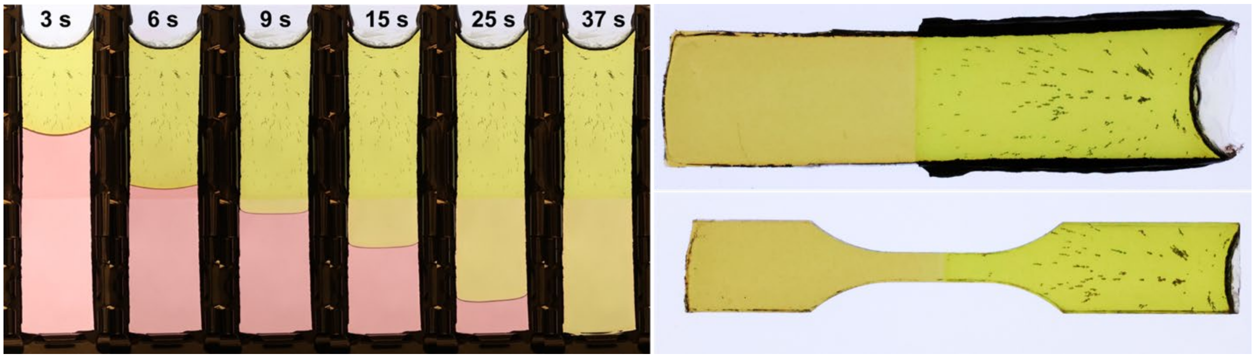

Figure S302: Representative front timelapse, front profile, and image of DCPD at 1000:1:1 (top) and 50 mol% NBI4 in DCPD at 1000:1:1 (bottom) monomer:G2:TBP **sharp interface** materials at 45 °C.

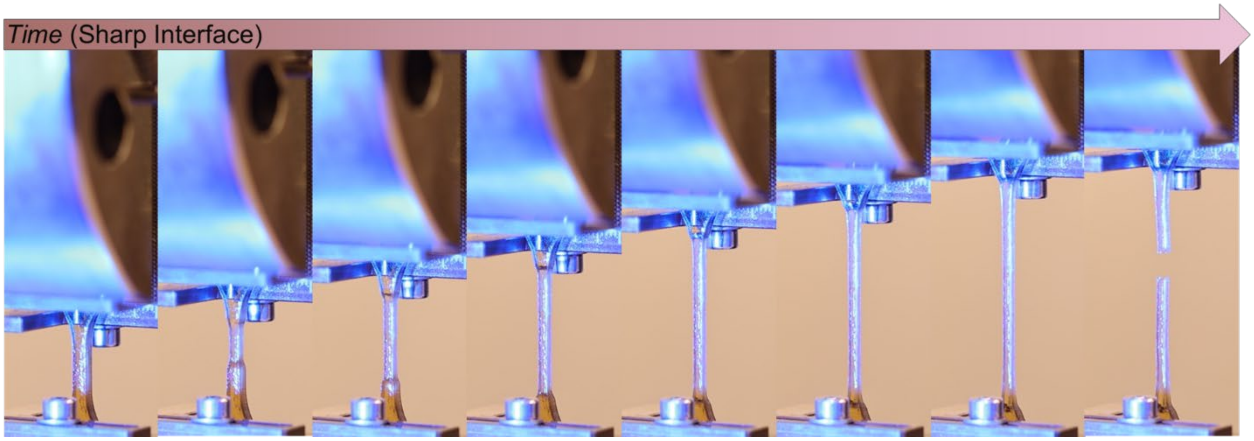

Figure S303: Representative tensile timelapse of DCPD at 1000:1:1 (top) and 50 mol% NBI4 in DCPD at 1000:1:1 (bottom) monomer:G2:TBP **sharp interface** materials.

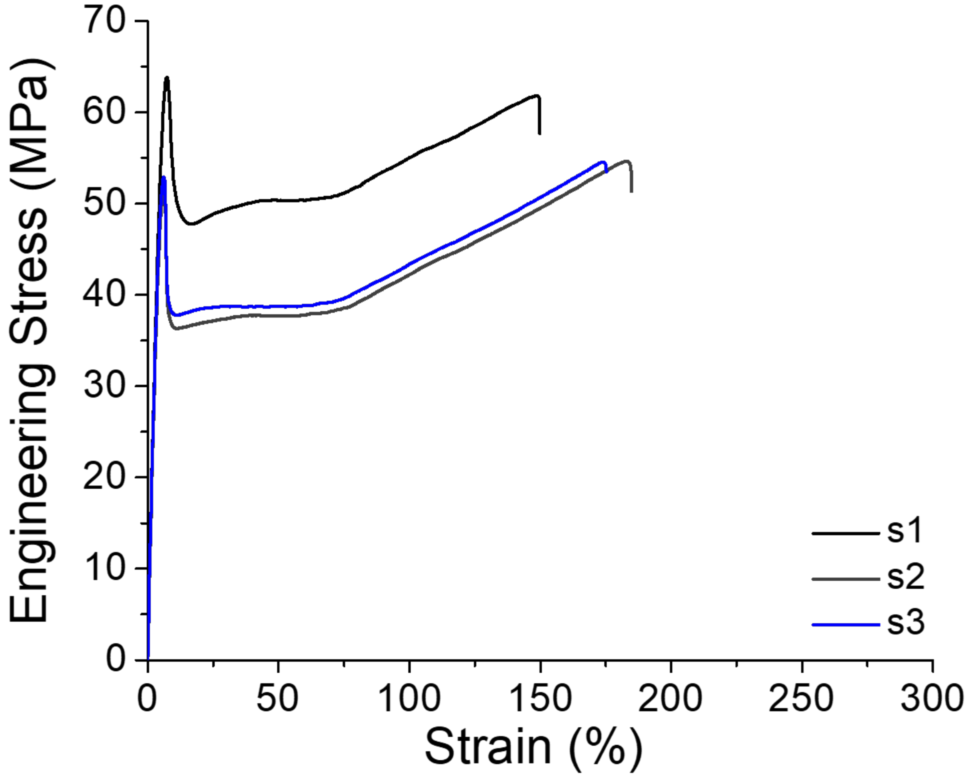

Figure S304: Stress-strain curves of DCPD at 1000:1:1 (top) and 50 mol% NBI4 in DCPD at 1000:1:1 (bottom) monomer:G2:TBP **sharp interface** (n = 3).

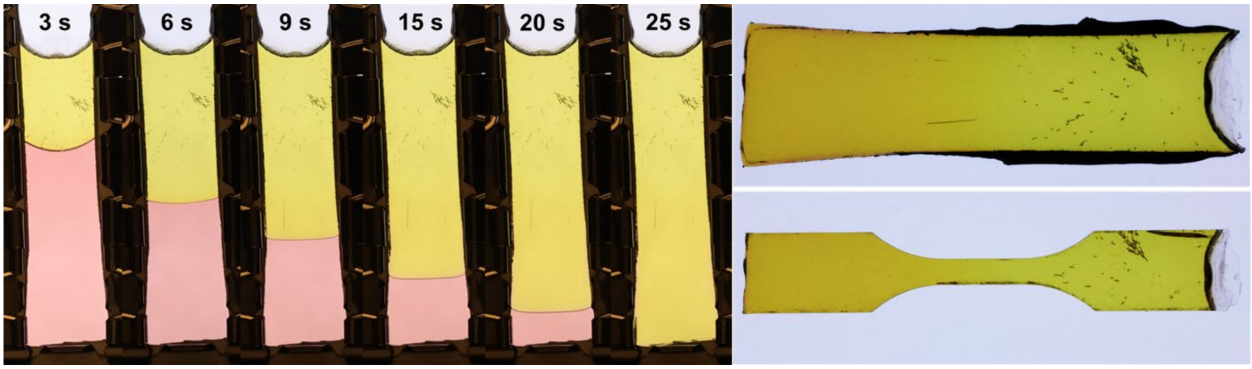

Figure S305: Representative front timelapse, front profile, and image of DCPD at 1000:1:1 (top) and 50 mol% NBI4 in DCPD at 1000:1:1 (bottom) monomer:G2:TBP **gradient interface** materials at 45 °C.

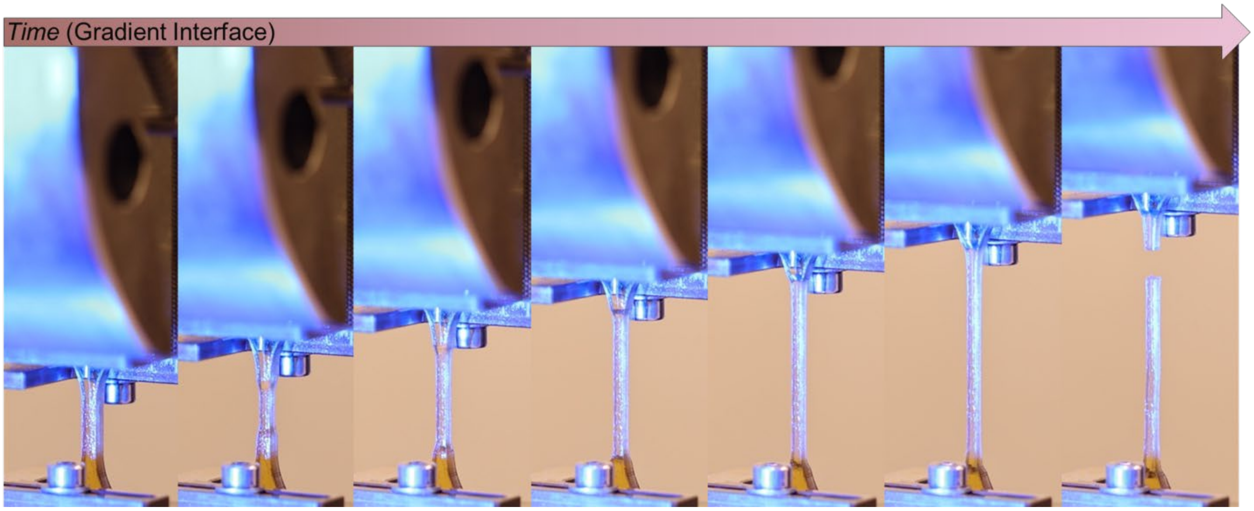

Figure S306: Representative tensile timelapse of DCPD at 1000:1:1 (top) and 50 mol% NBI4 in DCPD at 1000:1:1 (bottom) monomer:G2:TBP **gradient interface** materials.

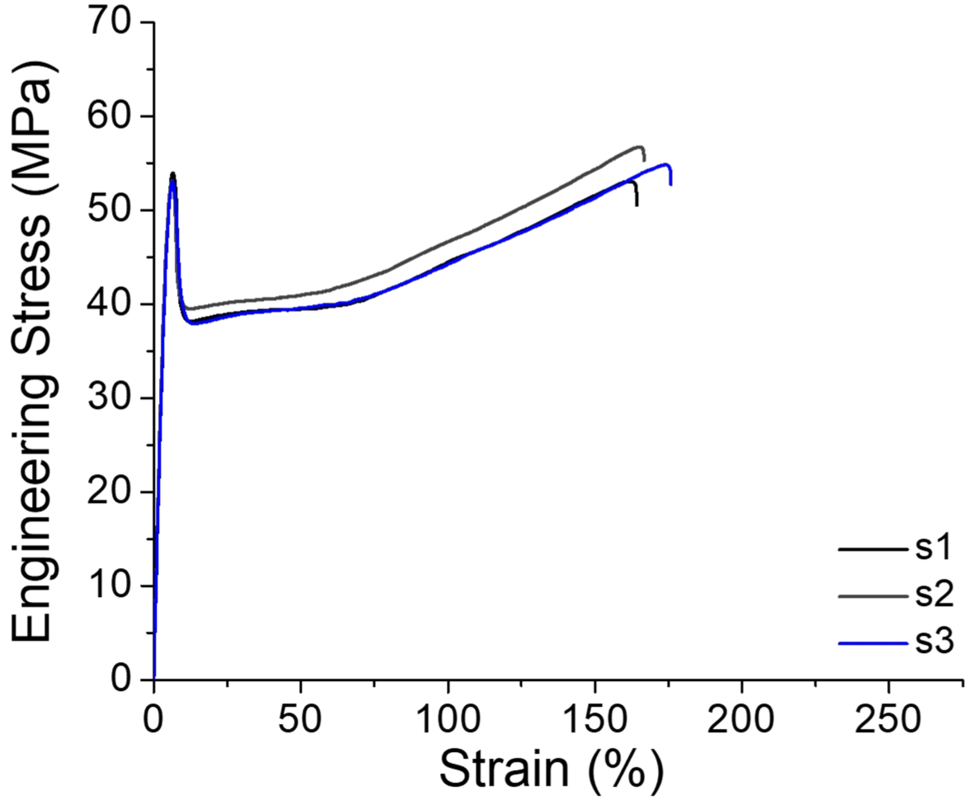

Figure S307: Stress-strain curves of DCPD at 1000:1:1 (top) and 50 mol% NBI4 in DCPD at 1000:1:1 (bottom) monomer:G2:TBP **gradient interface** materials (n = 3).

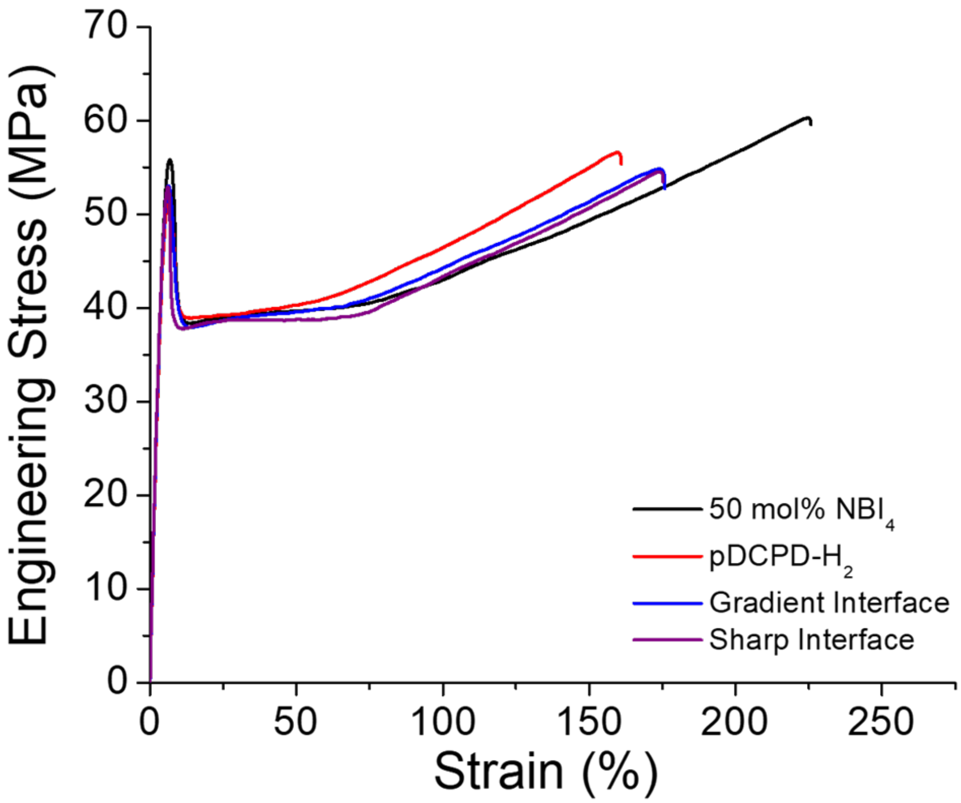

Figure S308: Overlaid representative stress-strain curves of DCPD, 50 mol% NBI4 in DCPD, sharp interface and gradient interface materials at 1000:1:1 (bottom) monomer:G2:TBP molar ratios.

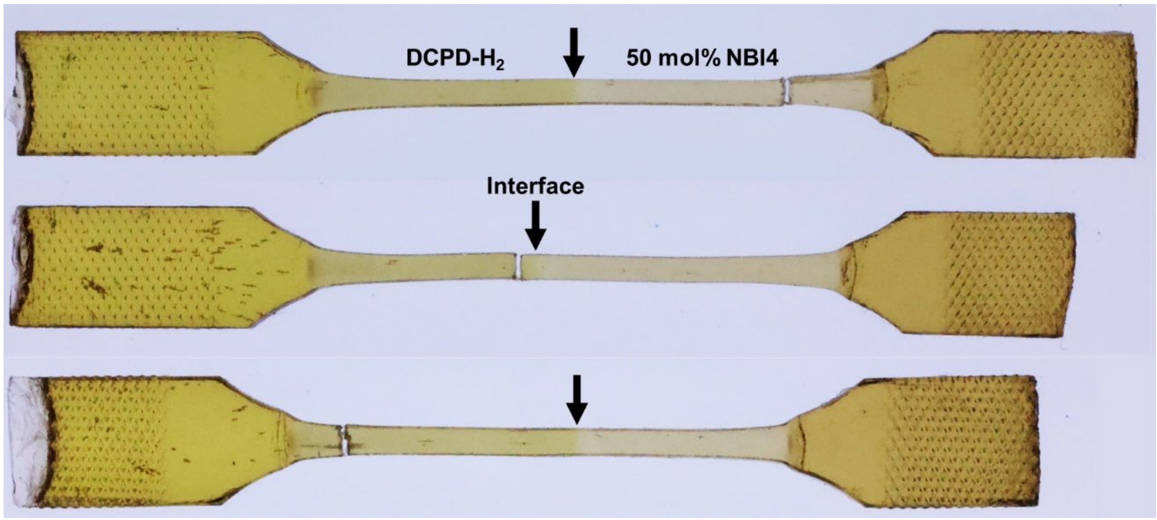

Figure S309: Sharp interface tensile test samples demonstration that the sample did not fail at the interface in any examples.

Table S21: Tensile data for FROMP-derived materials (n = 3) at molar ratios of 1000:1:1 monomer:initiator:inhibitor.

Sample	Elongation at Failure (%)	error	Ultimate Tensile Stress (MPa)	error	Yield Strength (MPa)	error	Young's Modulus (MPa)	error
**DCPD-H_2**								
1000:1:1	170	7	59.4	3.6	53.3	4.6	1347	33
**50 mol% NBI4**								
1000:1:1	225	6	61.5	2.9	56.5	2.9	1425	30
**Sharp Interface**								
1000:1:1	169	14	57.0	3.4	56.1	3.6	1399	4
**Gradient Interface**								
1000:1:1	169	5	55.1	1.2	53.1	0.1	1422	45

Youngs modulus was calculated from 0.1-0.2% strain.

References:

(1) Alzate-Sanchez, D. M.; Yu, C. H.; Lessard, J. J.; Paul, J. E.; Sottos, N. R.; Moore, J. S. Rapid Controlled Synthesis of Large Polymers by Frontal Ring-Opening Metathesis Polymerization. *Macromolecules* **2023**, *56* (4), 1527-1533. DOI: 10.1021/acs.macromol.2c01892.

(2) Mondal, S.; Lessard, J. J.; Meena, C. L.; Sanjayan, G. J.; Sumerlin, B. S. Janus Cross-links in Supramolecular Networks. *Journal of the American Chemical Society* **2022**, *144* (2), 845-853. DOI: 10.1021/jacs.1c10606.

(3) Nishimura, Y.; Chung, J.; Muradyan, H.; Guan, Z. Silyl Ether as a Robust and Thermally Stable Dynamic Covalent Motif for Malleable Polymer Design. *J. Am. Chem. Soc.* **2017**, *139* (42), 14881-14884. DOI: 10.1021/jacs.7b08826.
